# Single-cell transcriptome analyses reveal microglia types associated with proliferative retinopathy

**DOI:** 10.1172/jci.insight.160940

**Published:** 2022-12-08

**Authors:** Zhiping Liu, Huidong Shi, Jiean Xu, Qiuhua Yang, Qian Ma, Xiaoxiao Mao, Zhimin Xu, Yaqi Zhou, Qingen Da, Yongfeng Cai, David J.R. Fulton, Zheng Dong, Akrit Sodhi, Ruth B. Caldwell, Yuqing Huo

**Affiliations:** 1 Vascular Biology Center, Medical College of Georgia, Augusta University, Augusta, Georgia, USA.; 2 Guangdong Province Key Laboratory of Pharmacodynamic Constituents of TCM and New Drugs Research, College of Pharmacy, Jinan University, Guangzhou, China.; 3 Georgia Cancer Center and; 4 Department of Biochemistry and Molecular Biology, Medical College of Georgia, Augusta University, Augusta, Georgia, USA.; 5 Department of Cardiovascular Surgery, Peking University Shenzhen Hospital, Shenzhen, China.; 6 Department of Cellular Biology and Anatomy, Medical College of Georgia, Augusta University, Augusta, Georgia, USA.; 7 Charlie Norwood Veterans Affairs Medical Center, Augusta, Georgia, USA.; 8 Wilmer Eye Institute, Johns Hopkins School of Medicine, Baltimore, Maryland, USA.; 9 James and Jean Culver Vision Discovery Institute, Medical College of Georgia, Augusta University, Augusta, Georgia, USA.

**Keywords:** Angiogenesis, Ophthalmology, Molecular pathology, Mouse models, Retinopathy

## Abstract

Pathological angiogenesis is a major cause of irreversible blindness in individuals of all age groups with proliferative retinopathy (PR). Mononuclear phagocytes (MPs) within neovascular areas contribute to aberrant retinal angiogenesis. Due to their cellular heterogeneity, defining the roles of MP subsets in PR onset and progression has been challenging. Here, we aimed to investigate the heterogeneity of microglia associated with neovascularization and to characterize the transcriptional profiles and metabolic pathways of proangiogenic microglia in a mouse model of oxygen-induced PR (OIR). Using transcriptional single-cell sorting, we comprehensively mapped all microglia populations in retinas of room air (RA) and OIR mice. We have unveiled several unique types of PR-associated microglia (PRAM) and identified markers, signaling pathways, and regulons associated with these cells. Among these microglia subpopulations, we found a highly proliferative microglia subset with high self-renewal capacity and a hypermetabolic microglia subset that expresses high levels of activating microglia markers, glycolytic enzymes, and proangiogenic *Igf1*. IHC staining shows that these PRAM were spatially located within or around neovascular tufts. These unique types of microglia have the potential to promote retinal angiogenesis, which may have important implications for future treatment of PR and other pathological ocular angiogenesis–related diseases.

## Graphical abstract

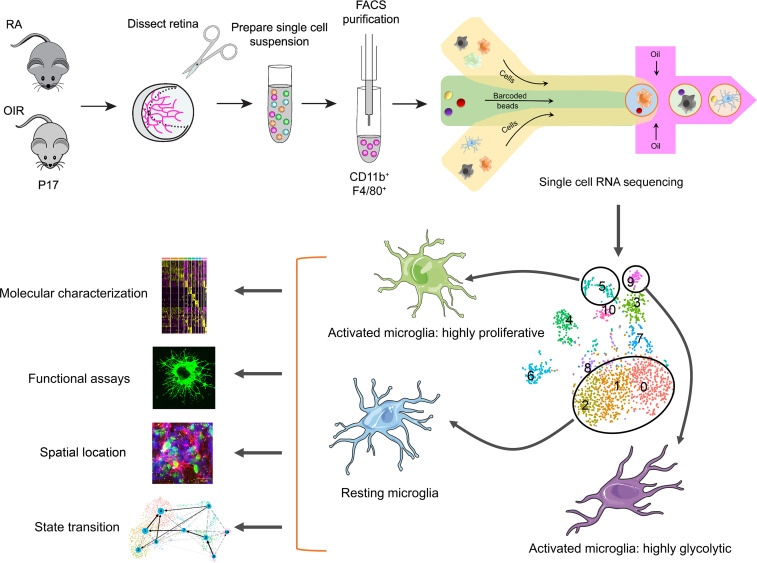

## Introduction

Proliferative retinopathies (PRs), which are primarily characterized as pathological retinal angiogenesis, are the leading cause of irreversible blindness in individuals of all age groups, including retinopathy of prematurity (ROP) in childhood, proliferative diabetic retinopathy (PDR) in middle-age adults, and age-related macular degeneration in the elderly (1–3). Accumulating evidence implicates mononuclear phagocytes (MPs), such as microglia, infiltrated monocytes, and monocyte-derived macrophages, in the regulation of pathological ocular neovascularization (4–7). Among the MPs, microglia are the resident myeloid cells of the CNS, encompassing both brain and retina (8–11). Although the CNS is thought to be immune privileged, it possesses unique capabilities due to the presence of resident microglia and their interplay with infiltrated monocytes and other cell types (12, 13). As the primary and unique immune cell population in the CNS, microglia function as key effectors and regulators of the microenvironment in the CNS (14). In response to a disruption of retinal homeostasis caused by various physiological and pathological factors such as injury, hypoxia, neuroinflammation degeneration, and/or aging, microglia undergo several specific activation processes (15, 16). These include rapid cell division, proliferation, and migration to pathologic sites; endocytosis and phagocytosis of cellular debris; and the generation of cytokines/chemokines or proangiogenic factors that stimulate other retinal cells in their vicinity, thereby promoting pathological retinal neovascularization (RNV) (17, 18). Indeed, we recently identified a metabolic link between microglia/macrophage activation and the stimulation of adjacent endothelial cells in the retinal angiogenic niche (19), emphasizing the critical role of this cell population in PRs.

The state of microglia (either resting or activated) is recognized as a hallmark of health and disease. Numerous studies have demonstrated that myeloid cells, particularly microglia, contribute to the onset and progression of RNV (20, 21). However, those studies relied on analysis of bulk cell populations associated with pathological RNV based on a subset of specific surface markers. Consequently, the heterogeneity, complexity, and diversity of the activated state and the niche specificity of MPs reacting to environmental changes in PRs remain undefined.

In this regard, single-cell genomics has enabled a more detailed and unbiased characterization of diverse immune cell populations at different states in vivo (8, 22, 23). Thus, comprehensive transcriptomic analysis using single-cell RNA-Seq (scRNA-Seq) has become a powerful and efficient tool to systematically define microglial heterogeneity in disease. Here, we combined scRNA-Seq analysis with IHC, genetic perturbation, and ex vivo functional analysis to comprehensively profile microglia populations in pathological RNV. In a mouse model of oxygen-induced retinopathy (OIR), we identified several unique PR-associated microglia (PRAM) subtypes. Further analysis unraveled the spatial location of PRAM adjacent to pathological neovascularization tufts. We elucidated detailed gene signatures, signaling pathways, transcription factor regulons, transcriptional dynamics, and metabolic characteristics in PRAM. Collectively, our study provides a comprehensive perspective that defines the contributions of retinal microglia to PR at the single-cell level.

## Results

### scRNA-Seq analysis of single cells isolated from retinas of OIR and room air (RA) control mice.

Recent studies have highlighted the critical role of immune cells, including microglia, monocytes, and macrophages, in the development of PRs (5, 6). Nonetheless, the transcriptomic and signaling heterogeneity of immune cells associated with disease progression remains poorly defined. Taking an unbiased approach, we therefore set out to better define the heterogeneity of microglia and other retinal myeloid cells in the pathogenesis of PRs. To this end, we used the 10X Genomics platform to perform scRNA-Seq on MPs from the mouse retina of OIR pups at P17 (Supplemental Figure 1A; supplemental material available online with this article; https://doi.org/10.1172/jci.insight.160940DS1). The mouse OIR retina is a well-accepted model for studying the pathological retinal angiogenesis that occurs in infants with ROP and in adults with PDR (24–26). In OIR, neonatal mice are exposed to 75% oxygen from P7 to P12, followed by exposure to RA from P12 to P17, during which mice develop a maximal neovascular response (Supplemental Figure 1B). A previous study has shown that there is a drastic increase (~170%) in the number of CD11b^+^ (a pan-myeloid cell marker) and F4/80^+^ (a marker for macrophage/microglia) cells during maximal neovascularization at P17 in the OIR retina as compared with the control retina exposed to RA only (6). Consistent with previous reports, IHC staining for F4/80 demonstrated a marked increase in the number of F4/80^+^ cells in OIR retinas at P17 (in areas of neovascularization) as compared with non-OIR control (RA) retinas (Supplemental Figure 1C).

Retinas from RA and OIR mice were dissociated, and all live cells expressing both CD11b and F4/80, which include microglia, monocytes, and macrophages, were collected using FACS for subsequent scRNA-Seq (Supplemental Figure 1D). Post hoc analysis of the collected cells showed a significant increase in the number of CD11b^+^F4/80^+^ cells during OIR (Supplemental Figure 1, D and E), consistent with the results of IHC staining of OIR retinas in vivo (Supplemental Figure 1C). scRNA-Seq libraries of CD11b^+^F4/80^+^ cells were prepared using the 10X Genomics platform and sequenced to an average sequencing depth of 89,257 raw reads per cell. Data from a total of 2,708 cells, including 1,449 cells from 16 RA retinas and 1,259 cells from 16 OIR retinas at P17, were obtained. We used graph-based clustering to identify the major subpopulations of cells within the combined RA and OIR single-cell data set, where the relative position of each point reflects the genetic similarity of the cell to others in the population. The majority of cells were found to be CD45^+^ myeloid cells, including microglia, monocytes, and macrophages, while a minority of cells were photoreceptor cells and other cell types (Supplemental Figure 2).

### Identification of myeloid subpopulations in retinas.

**Figure 1:**
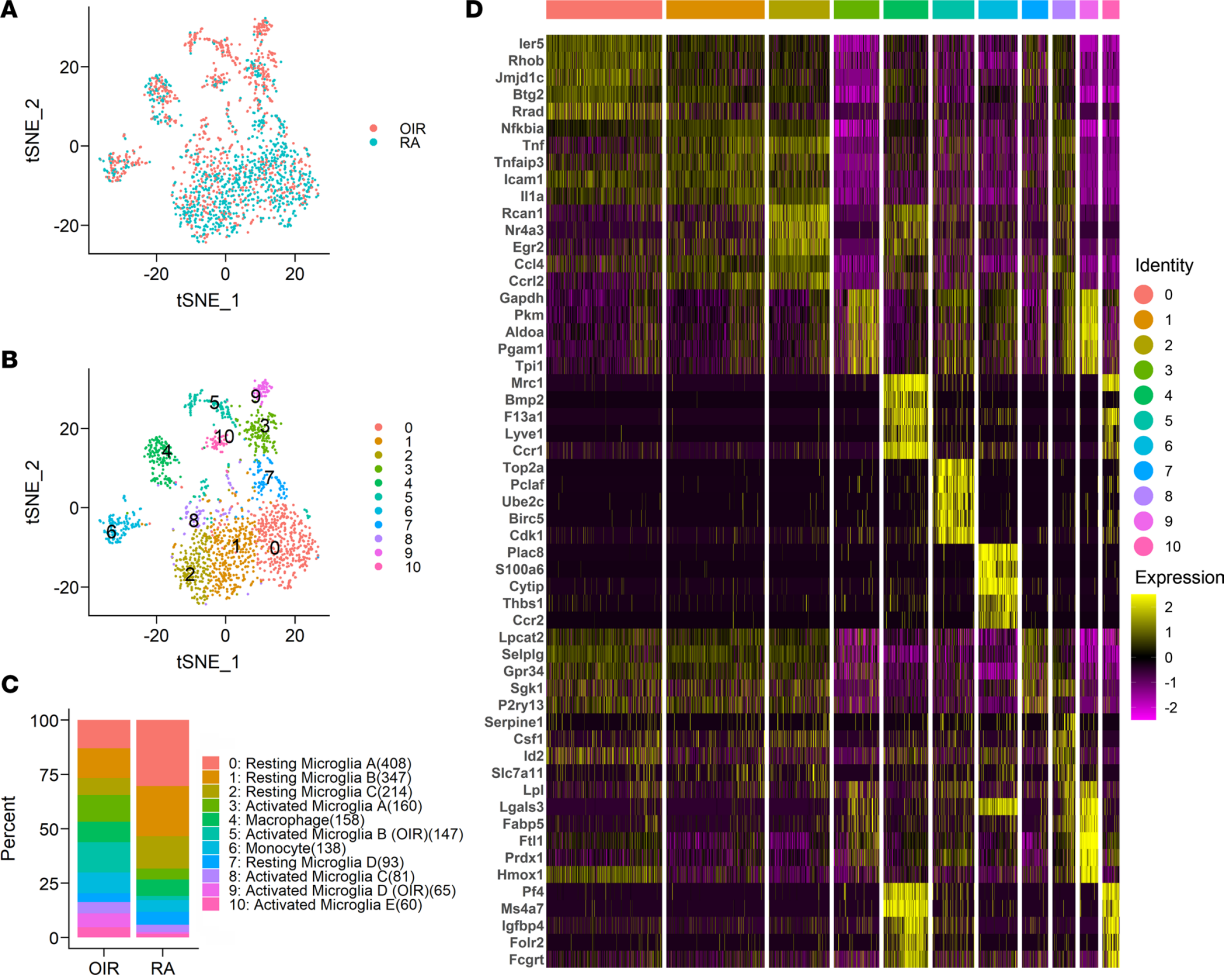
Clustering of retinal immune cells during OIR by scRNA-Seq. (**A**) Identification of immune cells during OIR in tSNE plot. Cells are color coded by sample identity, with mononuclear phagocytes from RA control retinas in blue-green and OIR retinas in orange-red. (**B**) Cells are color coded by cluster and identified by an unsupervised graph-based algorithm. (**C**) Bar graph showing the percentage of RA or OIR cells out of the total CD11b^+^F4/80^+^ cells in each cluster identified in **B**. Assigned cell identity using the relative abundance of established markers. The number in the parentheses is the number of cells in each cluster. (**D**) Heatmap showing the top 5 marker genes (or all marker genes if less than 5) for each of the 11 clusters.

To investigate the transcriptomic heterogeneity of retinal myeloid cells, we filtered out the CD45^–^ cells and performed further analysis on the myeloid cell populations. The t-distributed stochastic neighbor embedding (tSNE) plots revealed RA and OIR myeloid cells (Figure 1A) and displayed 11 transcriptionally distinct clusters (Figure 1, A–C) after data integration and removal of batch effects. Through analysis of differential gene expression, we generated a list of marker genes (Supplemental Tables 1 and 2) for each cluster. Expression of these genes uniquely or in different combinations signified individual cluster identities (Figure 1, C and D). Based on the list of marker genes for each cluster (Supplemental Tables 1 and 2) and known markers for microglia, monocytes, and macrophages, we annotated the 11 clusters into distinct cell types or states. This included 9 microglia clusters, 1 macrophage cluster, and 1 monocyte cluster (Figure 1, B and C, and Supplemental Figure 3).

**Figure 2:**
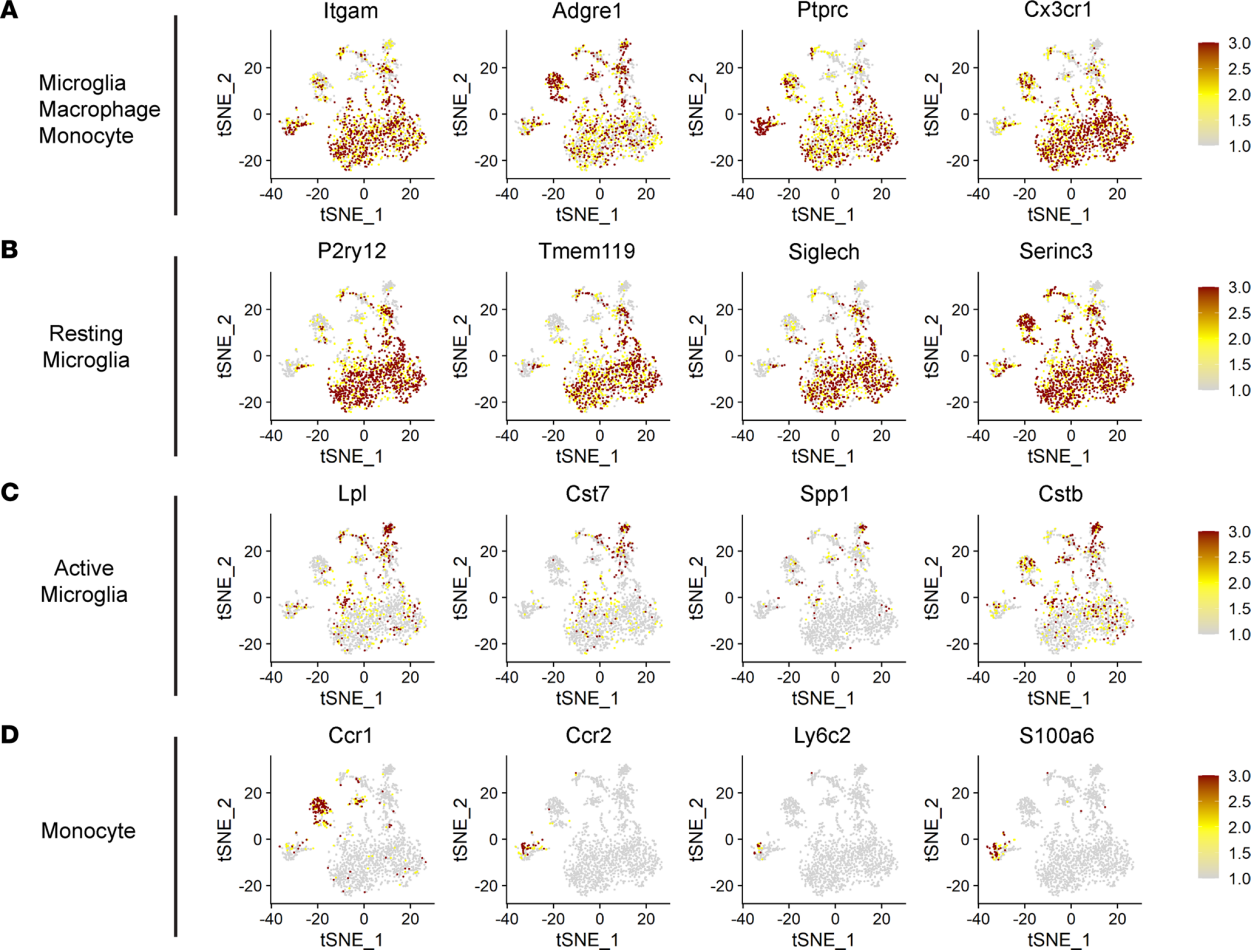
Identification of retinal immune cells during OIR by scRNA-Seq. (**A**) tSNE plots colored for expression of canonical microglial/mononuclear phagocyte genes. (**B**) tSNE plots colored for expression of genes upregulated explicitly in resting microglial clusters. (**C**) tSNE plots colored for expression of genes upregulated explicitly in activated microglial clusters. (**D**) tSNE plots colored for expression of genes upregulated explicitly in monocyte clusters.

All cells in the myeloid cell subset expressed the myeloid cell lineage markers *Ptprc* (CD45), *Itgam* (CD11b), and *Adgre1* (F40/80) (Figure 2A). Projection of cells using tSNE plots separated the monocyte and macrophage groups from the microglia subpopulations (Figure 1B), while the latter contained different subsets of resting and activated microglia. Cells in 4 clusters (0, 1, 2 and 7), mostly from control RA retina (Figure 1, A–C), expressed high levels of resting microglia markers (i.e., homeostatic genes), such as *P2ry12*, *Tmem119*, *Siglech*, and *Serinc3* (11, 22) (Figure 2B). Cells in clusters 5, 9, and 10, which primarily appeared in the OIR retinas, expressed high levels of microglial activation markers, such as *Lpl*, *Cst7*, *Spp1*, and *Cstb* (8, 22) (Figure 2C). Two clusters (3 and 8; Figure 1, A and B) contained intermediate levels of microglial activation markers (Figure 2C), consistent with an intermediate state between resting and activated microglia. Markers of monocyte-derived macrophages, such as *H2-Aa* and *H2-Ab1* (11, 22), were mainly expressed in cells of cluster 4 (Supplemental Figure 3), while monocyte markers such as *Ccr2* (22) and *Ly6c2* (11) were expressed by cells in cluster 6 (Figure 2D).

### Identification of hyperglycolytic microglia associated with retinopathy.

**Figure 3:**
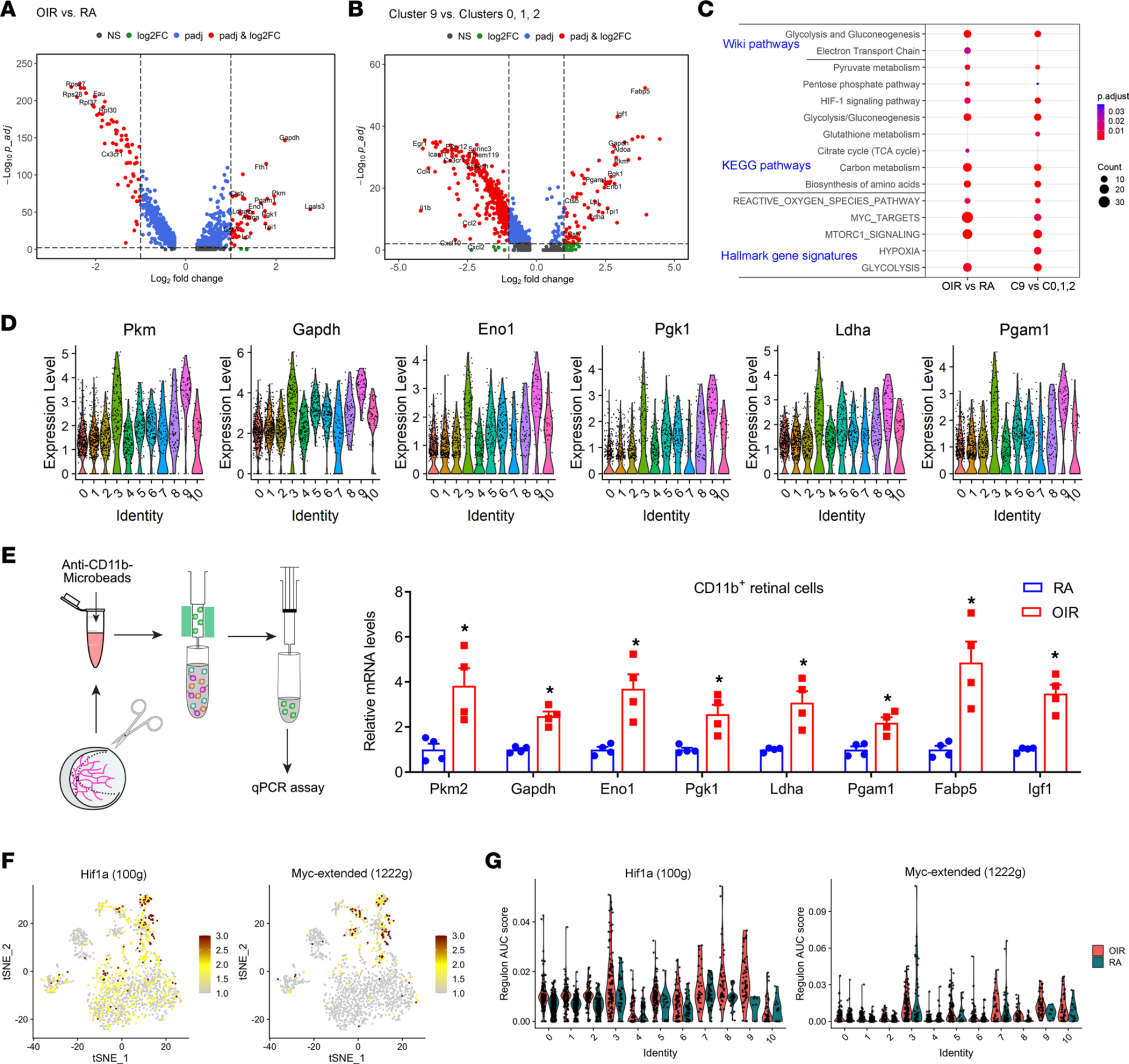
scRNA-Seq revealed elevated metabolic activities in OIR microglia. (**A**) Volcano plot showing differentially expressed genes between OIR and RA microglia. Each dot represents a gene. Genes with absolute average log_2_ fold change > 1 and adjusted *P* < 0.01 are highlighted in red. Representative upregulated and downregulated genes are labeled in each panel. *P* values were determined using the Wilcoxon rank-sum test with Bonferroni correction using all genes in the data set. (**B**) Volcano plot showing differentially expressed genes between cluster 9 and cluster 0, 1, and 2 microglia. (**C**) Dot plot showing pathway enrichment results from 3 independent pathway databases. The lists of upregulated genes in OIR versus RA microglia and cluster 9 versus clusters 0, 1, and 2 were used as inputs for the analysis. The cutoff thresholds for gene inclusion are: average log_2_ fold change > 0.585, adjusted *P* < 0.05, and expression in at least 15% of OIR or cluster 9 cells. (**D**) Violin plots showing differential expression of 6 glycolysis-related genes among the 11 clusters of CD11b^+^F4/80^+^ cells. Each dot represents 1 cell, and the violin shows the distribution of probability density at each value. (**E**) qPCR analysis of gene expression in microglia isolated from RA or OIR retinas in mice at P17 using anti-CD11b MicroBeads (*n* = 4). Data are mean ± SEM. *P < 0.05 versus RA by Student’s *t* test or nonparametric Mann–Whitney *U* test. (**F**) tSNE plot showing regulon activities of Hif1a and Myc extended regulon activity scores among the 11 clusters of CD11b^+^F4/80^+^ cells. The number in the parentheses indicates the number of genes in the regulon. (**G**) Violin plots showing differential activities of Hif1a and Myc extended regulon activity scores among the 11 clusters of CD11b^+^F4/80^+^ cells.

Our next goal was to further characterize the 9 microglial clusters. RA and OIR conditions did not cause a great difference in the numbers of macrophages and monocytes in clusters 4 and 6 (Figure 1, A–C). In contrast, OIR remarkably distinguished the microglia in clusters 3, 5, 9, and 10 (Figure 1, A–C), and we defined the cells in these clusters as PRAM. A comparison of the expression profiles and key marker genes of myeloid cells in RA retinas to those in OIR retinas demonstrated significant upregulation of glycolytic molecules, including *Pgam1*, *Pkm*, *Gapdh*, *Aldoa*, *Tpi1*, *Eno1*, and *Ldha* in OIR groups (Figure 3A). The increased expression of glycolytic genes was even more prominent when comparing cells of clusters 0, 1, and 2 (the major resting clusters) with cells of cluster 9 (the most activated cluster from the OIR retinas) (Figure 3B). Furthermore, pathway enrichment analysis of upregulated genes in OIR microglia compared with RA microglia, and in cluster 9 compared with clusters 0, 1, 2 of OIR microglia using 3 well-annotated databases, revealed significant involvement of genes in pathways of glycolysis, HIF-1 signaling, and MYC targets, lending further support to a hyper-glycolytic phenotype in subsets of OIR microglia (Figure 3C).

The scRNA-Seq data reveal a gradual increase in the expression of critical genes involved in the glycolysis pathway from resting clusters 0, 1, 2, and 7 to OIR intermediate activated microglia (clusters 3, 5, 8, and 10) and OIR most-activated microglia (cluster 9) populations (Figure 3D and Supplemental Figure 4). The increased expression of glycolytic molecules was verified by quantitative PCR (qPCR) in CD11b^+^ cells isolated from OIR retinas (Figure 3E). Of note, the difference in glycolytic gene expression was remarkable among microglia of different OIR clusters. OIR microglia of cluster 9 expressed the highest levels of glycolytic genes among the OIR microglial clusters (Supplemental Figure 4), suggesting that cluster 9 may represent a unique population that is highly enriched with hyperglycolytic microglia.

We next performed a gene regulation network analysis using the single-cell regulatory network inference and clustering (SCENIC) package (27, 28). This analysis revealed differential expression of various transcription-factor networks in the microglia of different subpopulations (Supplemental Figure 5). Since almost all enzymes of the glycolytic cascade are regulated by HIF-1 (29), the principal regulator of the transcriptional response to hypoxia, as well as by MYC (30), a vital regulator of metabolic reprogramming in both normal cells and cancer cells (31), we hypothesized that HIF-1 and MYC might mediate the upregulation of glycolytic genes in OIR microglia. Indeed, we found that regulons driven by both HIF-1 and MYC transcription factors were upregulated in OIR microglia, especially in cluster 9 (Figure 3, F and G). In addition, we performed pathway enrichment analysis at the single-cell level and used the pathway activity score to perform cluster analysis. Metabolic pathway analysis revealed that cluster 9 microglia showed increased metabolism of fatty acids, amino acids, glutathione, and oxidative phosphorylation (OXPHOS), in addition to glycolysis (Supplemental Figure 6), indicating that other regulatory mechanisms beyond HIF-1– and MYC-mediated glycolysis might be involved in the activation of multiple metabolic pathways in cluster 9 cells.

### Identification of a highly proliferative microglia subpopulation associated with retinopathy.

**Figure 4:**
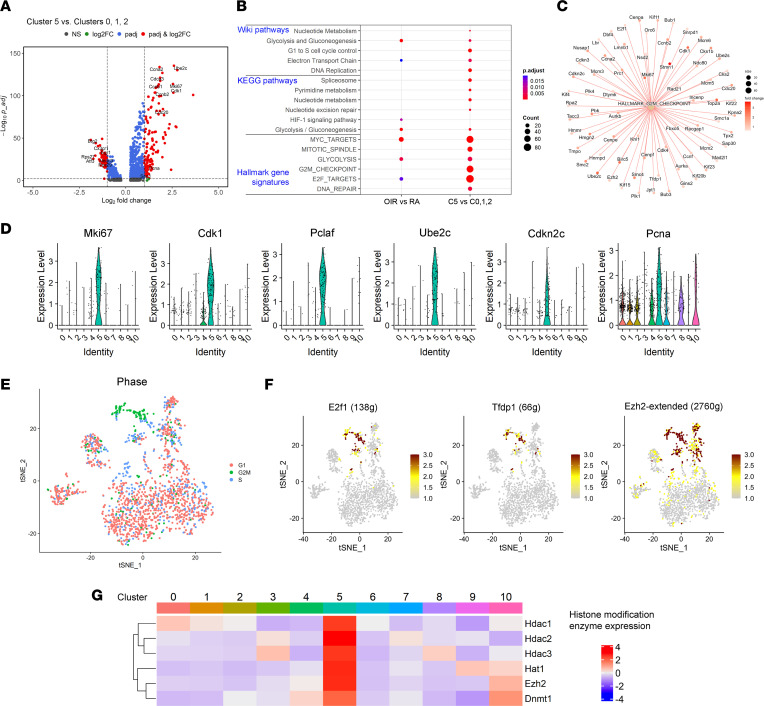
scRNA-Seq reveals a highly proliferative microglia cluster associated with OIR. (**A**) Volcano plot showing differential gene expression between cluster 5 cells to cluster 0, 1, and 2 cells. Each dot represents a gene. Genes with absolute average log_2_ fold change > 1 and adjusted *P* < 0.01 are highlighted in red. Representative upregulated and downregulated genes are labeled in each panel. *P* values were determined using the Wilcoxon rank sum test with Bonferroni correction using all genes in the data set. (**B**) Dot plot showing pathway enrichment results from 3 independent pathway databases. The lists of upregulated genes in cluster 5 versus clusters 0, 1, and 2 are used as inputs for the analysis. The cutoff thresholds for gene inclusion are: average log_2_ fold change > 0.585, adjusted *P* < 0.05, and expression in at least 15% of cluster 5 cells. (**C**) Cnetplot showing a list of genes in Hallmarks G2M checkpoint signatures upregulated in cluster 5 as compared with resting microglia (clusters 0, 1, and 2). (**D**) Violin plots of 6 unique marker genes representing cluster 5. (**E**) tSNE plot of cell cycle scores calculated based on a group of 97 cell cycle–related genes. (**F**) tSNE plot showing E2f1, Tfdp1, and Ezh2 extended regulon activity scores among the 11 clusters of CD11b^+^F4/80^+^ cells. (**G**) Heatmap showing average expression of several histone modification enzymes among the 11 clusters of CD11b^+^F4/80^+^ cells.

We and others have consistently shown a dramatic increase in the number of macrophages/microglia near neovascular tufts (NV tufts) during OIR (6) (Supplemental Figure 1C). Thus, we searched for highly proliferative subpopulations of microglia in OIR groups. When we compared cells of the resting microglia in clusters 0, 1, and 2 to active microglia in cluster 5, we found that proliferation-related genes were among the most highly upregulated genes in cluster 5 and primarily appeared in the OIR retina (Figure 4A). Pathway enrichment analysis of upregulated genes in cluster 5 cells compared with resting microglia (clusters 0, 1, and 2) revealed significant involvement in DNA replication and cell cycle control–related pathways (Figure 4, B and C). These upregulated genes, such as *Mki67*, *Cdk1*, *Pclaf*, *Ube2c*, *Cdhn2c*, and *Pcna*, were uniquely expressed at a high level in cluster 5 but not in any other clusters (Figure 4D). Pathway analysis at the single-cell level showed similar results (Supplemental Figure 6). In addition, cell cycle analysis of the scRNA-Seq data demonstrated that almost all cells of cluster 5 are in the G2M phase (Figure 4E), supporting the notion that cluster 5 is a subpopulation of highly proliferative OIR microglia. In line with the pathway enrichment analysis, cell growth and metabolism-related regulons such as E2f1 (32), Ezh2 (33), and Tfdp1 were upregulated in activated microglia of cluster 5 of OIR retinas (Figure 4F and Supplemental Figure 5). Furthermore, in accordance with recent findings in tumor-associated T cells that genes regulating chromatin modification are coexpressed in proliferative subpopulations of T cells (34), we also observed that gene signatures associated with chromatin modification (i.e., *Ezh2*, *Hdac2*, *Hdac3*, *Hat1*, and *Hdac1*) were predominantly detected in cells of cluster 5 (Figure 4G), suggesting that chromatin modification–mediated epigenetic regulatory mechanisms might underlie the upregulation of proliferative markers and cell cycle–related genes during PR.

### Inflammatory status of various microglia populations.

**Figure 5:**
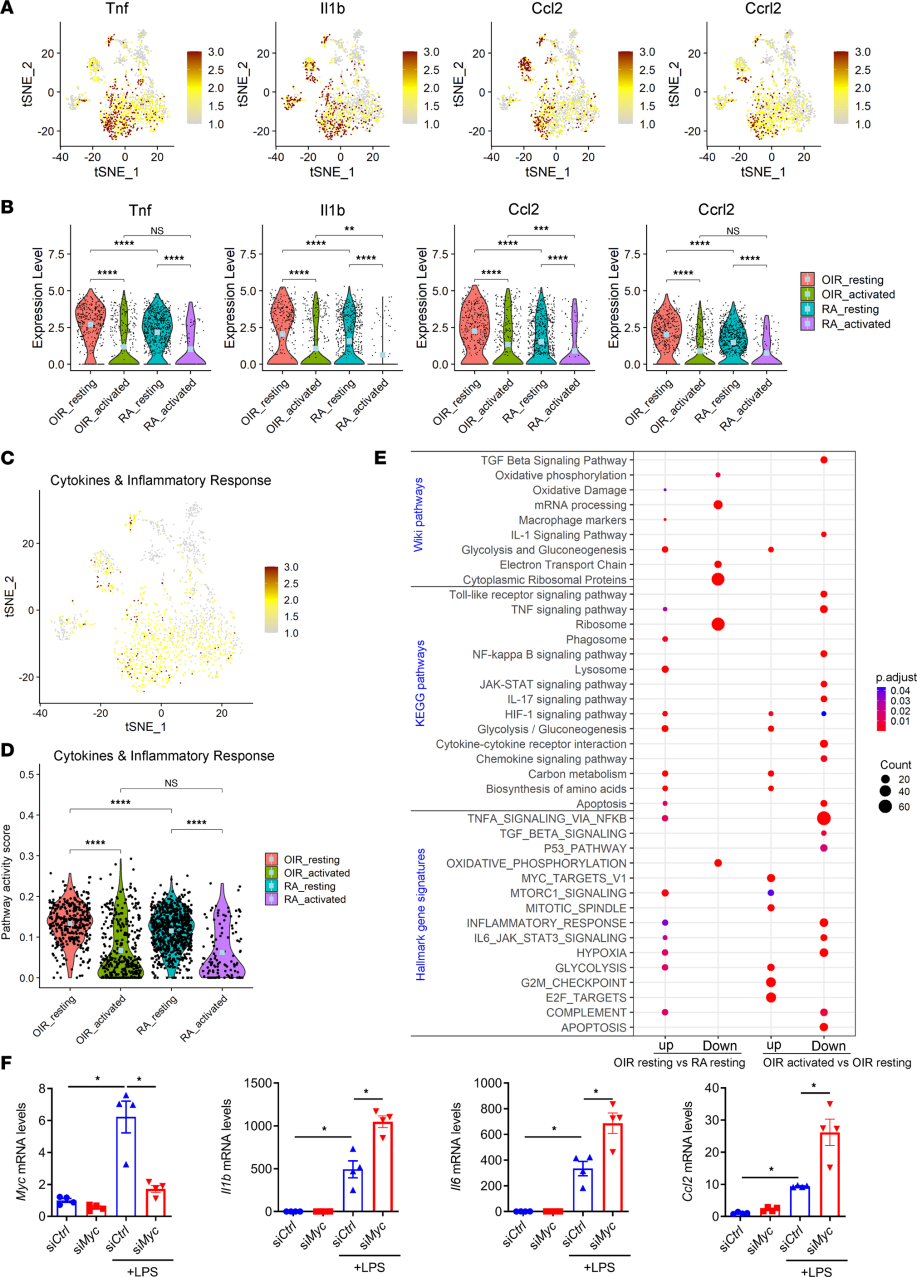
scRNA-Seq revealed heterogeneity of inflammatory gene expression in resting and activated microglia. (**A**) tSNE plots colored for 4 inflammatory genes. (**B**) Violin plots of 4 inflammatory genes. *P* values were determined by the Wilcoxon rank-sum test in R. **P < 0. 01, ***P < 0. 001, ****P < 0.0001. Light blue dots indicate the mean. (**C**) tSNE plots showing cytokine and inflammatory pathway activity scores calculated using the AUCell package. (**D**) Violin plots showing cytokine and inflammatory pathway activity scores. *P* values were determined by the Wilcoxon rank-sum test in R. ****P < 0.0001. Light-blue dots indicate the mean. (**E**) Dot plot showing pathway enrichment results from 3 independent pathway databases. The lists of upregulated and downregulated genes in OIR versus RA resting microglia (clusters 0, 1, 2) and OIR versus RA-activated microglia (clusters 5, 9, and 10) are used as inputs for the analysis. The cutoff thresholds for gene inclusion are: absolute average log_2_ fold change > 0.585, adjusted *P* < 0.05, and expression in at least 15% of OIR or RA microglia. (**F**) qPCR analysis of inflammatory cytokine genes in negative control siRNA and *Myc* siRNA–transfected N9 microglial cells after LPS stimulation (10 ng/mL) for 6 hours (*n* = 4). Data are mean ± SEM. *P < 0.05 by 1-way ANOVA followed by Bonferroni test.

We and others have previously demonstrated the upregulation of inflammatory cytokines in the OIR model. However, scRNA-Seq analysis revealed significant heterogeneity in the expression levels of primary inflammatory cytokines (Figure 5A) in both resting and activated microglia. For instance, among 4 populations of resting microglia, microglia of cluster 2 appeared to express relatively high mRNA levels of *Il1b*, *Tnf*, *Ccl2*, and *Ccrl2*, compared with cells of clusters 0, 1, and 7 (Figure 5A). Similarly, compared with activated OIR microglia in clusters 3, 5, 9, and 10, OIR microglia in cluster 8 produced a significant amount of *Il1b*, *Tnf*, *Ccl2*, and *Ccrl2* mRNA (Figure 5A). When we divided each RA and OIR microglia into 2 groups and performed the statistical analysis, we found that the resting microglia populations of OIR microglia in clusters 0, 1, 2, and 7 expressed statistically higher levels of *Il1b*, *Tnf*, *Ccl2*, and *Ccrl2* than RA resting microglia in clusters 0, 1, 2, and 7 — the major populations of both RA and OIR microglia (Figure 5B). Activated microglia in both OIR and RA retinas displayed reduced expression of *Il1b*, *Tnf*, *Ccl2*, and *Ccrl2* compared with OIR and RA resting microglia, respectively (Figure 5B). There was no significant difference in the expression of *Tnf* and *Ccrl2* between activated OIR and RA microglia. The pathway enrichment score calculated using additional genes in the cytokines and inflammatory response pathways revealed similar heterogeneity and trend (Figure 5, C and D). Furthermore, the results of pathway enrichment analysis suggested that several inflammatory pathways, such as TLR signaling, TNF signaling, NF-κB signaling, and hallmark inflammatory response pathways, were all downregulated in OIR activated microglia (cluster 3, 5, 8, 9, 10) compared with OIR resting microglia (clusters 0, 1, 2, 7) subpopulations (Figure 5E). Surprisingly, these results suggest that cellular metabolism may be disconnected from the inflammatory response in cells of activated microglia clusters in OIR retinas, evidenced by low expression of genes involved in inflammation-related pathways and significant enrichment of genes involved in the cellular metabolism–related pathways, especially glycolysis (Figure 5E and Supplemental Figure 6). Consistent with this notion, a recent study showed that MYC-driven glycolysis negatively regulates proinflammatory responses in inflammatory macrophages (35). A similar effect was observed by us in inflammatory microglia. We found that knockdown of *Myc* by siRNA transfection led to significant upregulation of inflammatory factors *Il1b*, *Il6*, and *Ccl2* in LPS-treated N9 microglial cells (Figure 5F).

### Unique microglia populations near pathological NV tufts.

**Figure 6:**
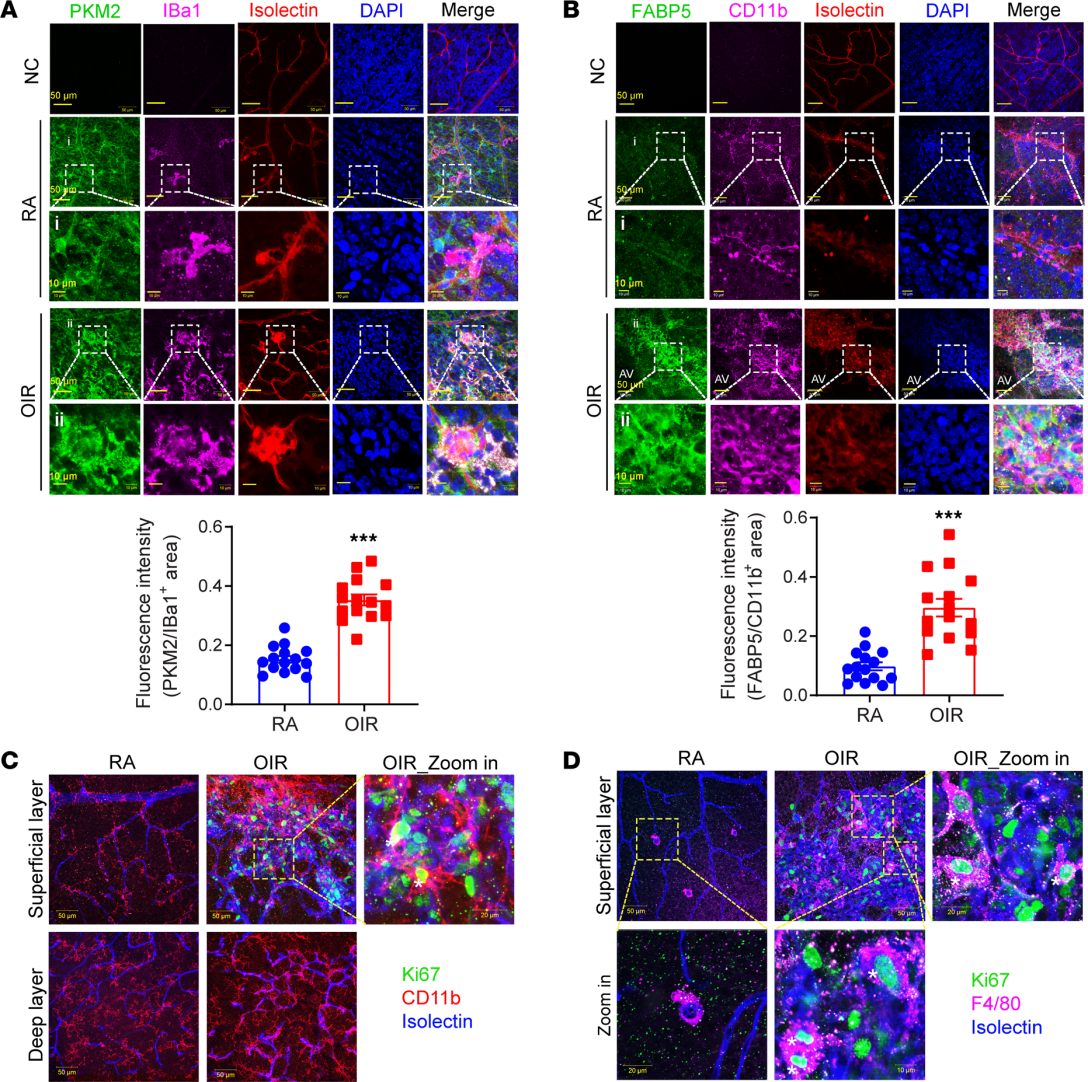
The unique PRAM are localized near the pathological neovascular tufts. (**A**) Representative immunofluorescence images of PKM2 (green), microglia (IBa1, pink), vessel (isolectin B4, red), and nuclei (DAPI, blue) in whole-mount retinas of RA and OIR mice at P17. Scale bar: 50 μm (first, second, and fourth rows) and 10 μm (third and fifth rows). NC, negative controls; stained with isotype IgG of PKM2 and IBa1 antibody. The fluorescence intensity of PKM2 staining was calculated by ImageJ software (NIH) (5 images × 3 regions were obtained from *n* = 5 retinas of each group). Data are mean ± SEM. ***P < 0.001 versus RA by nonparametric Mann–Whitney *U* test. (**B**) FABP5 immunofluorescence staining of retinas from RA and OIR mice at P17. Representative FABP5 (green), CD11b (pink), isolectin B4 (red), DAPI (nuclei, blue), and merged images captured with confocal fluorescence microscopy. Scale bar: 50 μm (first, second, and fourth rows) and 10 μm (third and fifth rows). Stained with isotype IgG of FABP5 and CD11b antibody. The fluorescence intensity of FABP5 staining was calculated by ImageJ software (5 images × 3 regions were obtained from *n* = 5 retinas of each group). Data are mean ± SEM. ***P < 0.001 versus RA by nonparametric Mann–Whitney *U* test. (**C**) Representative immunofluorescence images of nuclear Ki67 (green), myeloid cells (CD11b, red), and vessel (isolectin B4, blue) in whole-mount retinas of RA and OIR mice at P17 (*n* = 4 per group). Scale bar: 50 μm and 20 μm (far right). (**D**) Immunofluorescence imaging of nuclear Ki67^+^ (white asterisk labelled, green), microglia/macrophages (F4/80, pink), and vessel (isolectin B4, blue) in whole-mount retinas of RA and OIR mice at P17 (*n* = 4 per group). Scale bar: 50 μm and 20 μm (far right).

The unique profile of gene expression in the PRAM population prompted us to examine these activated microglia populations in greater detail. To examine the spatial location of these unique microglia populations, we performed multiple immunofluorescence staining assays based on the molecular characteristics of the PRAM population using whole-mounted retina from both RA and OIR mice. We focused on markers of glycolytic and proliferating PRAM and microglial markers to locate clusters 9 and 5. Retinal staining for PKM2, together with IBa1, a classical microglial marker, showed modest expression of PKM2 in the microglia of the RA retina. In contrast, we observed prominent staining for PKM2 in IBa1^+^ microglia in the OIR retina (Figure 6A).

We next examined the distribution of IBa1^+^ and PKM2^+^ cells in OIR retinas stained for NV tufts using isolectin B4. This showed that microglia that coexpress these markers are within or in close proximity to NV tufts (Figure 6A). Similarly, when we probed for the spatial distribution of FABP5 — another PRAM-specific gene in cluster 9 — using immunofluorescence staining, we observed a dramatic upregulation of this protein on microglia near the NV tufts (Figure 6B).

We also characterized the subpopulation of proliferating cells (cluster 5) in the OIR retina identified through scRNA-Seq. Utilizing double immunofluorescence staining with a proliferation marker (Ki67) and microglia marker (F4/80 or CD11b), we confirmed the presence of mitotically active microglia in OIR tissue (Figure 6C). Ki67^+^ nuclei were localized to the pathological NV tufts, partially in microglia/macrophages, as evidenced by its colocalization with F4/80 (Figure 6D). In contrast, Ki67^+^ microglia/macrophages were barely observed in RA retinas, in agreement with the scRNA-Seq data.

### IGF1 is the major growth factor underlying the proangiogenic effects of the highly glycolytic microglia subpopulation.

**Figure 7:**
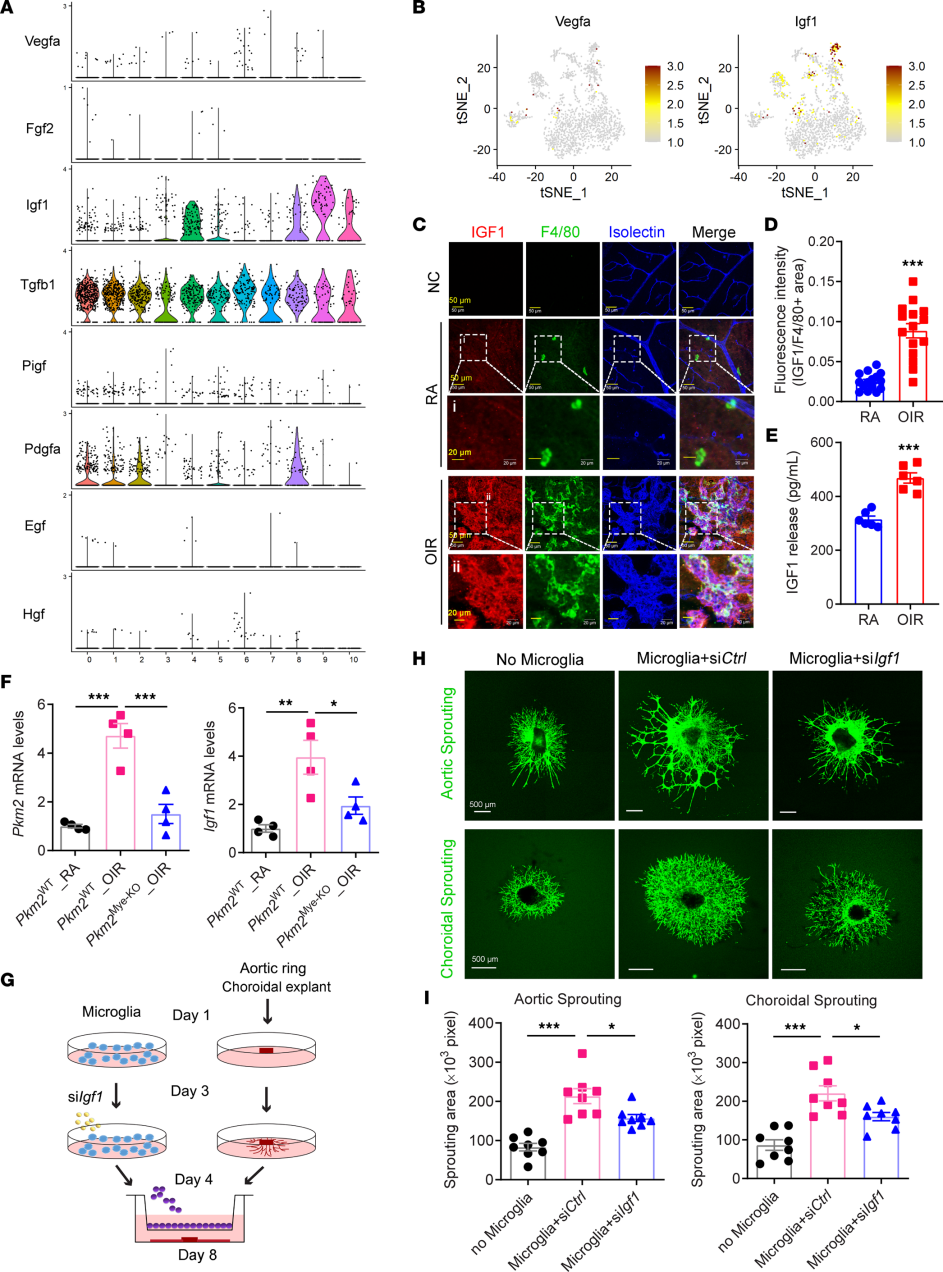
Identification of IGF1 as the major growth factor mediating the angiogenic effects of the highly glycolytic PRAM. (**A**) Violin plots showing the gene expression of growth factors among the 11 clusters of CD11b^+^F4/80^+^ cells. (**B**) tSNE plot showing the gene expression of *Vegfa* and *Igf1* among the 11 clusters of CD11b^+^F4/80^+^ cells. (**C**) Representative immunofluorescence images of IGF1 (red), microglia/macrophages (F4/80, green), and vessel (isolectin B4, blue) in whole-mount retinas of RA and OIR mice at P17. NC, negative controls; stained with isotype IgG of IGF1 and F4/80 antibody. Scale bar: (first, second, and fourth rows) and 20 μm (third and fifth rows). (**D**) The fluorescence intensity of IGF1 staining was calculated by ImageJ software (5 images × 3 regions were obtained from *n* = 5 retinas of each group). Data are mean ± SEM. ***P < 0.001 versus RA by Student’s *t* test. (**E**) Retinal microglia were isolated and cultured from P17 OIR mice or control (RA) mice. Culture supernatants were analyzed for IGF1 protein content by ELISA (*n* = 6). Data are mean ± SEM. ***P < 0.001 versus RA by Student’s *t* test. (**F**) qPCR analysis of mRNA expression of *Pkm2* and *Igf1* in myeloid cells isolated from RA or OIR retinas of *Pkm2*^WT^ or *Pkm2*^Mye-KO^ mice at P17 using CD11b antibody–conjugated magnetic beads (*n* = 4). Data are presented as mean ± SEM. *P < 0.05, **P < 0.01, ***P < 0.001. Statistical significance was determined by 1-way ANOVA followed by the Bonferroni test. (**G**) Schematic illustration of coculture of aortic/choroidal explants and retinal microglia. (**H**) Mouse retinal microglia (MRM) were transfected with siRNAs targeting mouse *Igf1* (si*Igf1*) or with a nontargeting negative control (si*Ctrl*). Forty-eight hours after transfection, MRM were harvested and cocultured with aortic ring and choroidal explants in transwells. Representative images of sprouting aortic rings and choroids were captured with a fluorescence confocal microscope. (**I**) Sprouting areas were quantified in Figure 6H. *n* = 8. *P < 0.05, ***P < 0.001. Data are mean ± SEM. Statistical significance was determined by 1-way ANOVA followed by the Bonferroni test.

Since hyperglycolytic microglia were associated with OIR, we next asked whether glycolytic reprogramming induces microglia to generate specific angiogenic factors that would facilitate pathological retinal angiogenesis. Growth factor expression profiles revealed that *Igf1* and *Tgfb1*, but not *Vegfa*, were highly expressed in retinal microglia/macrophages (Figure 7, A and B). This is consistent with a previous study showing that VEGF is barely detectable in retinal macrophages (36). Intriguingly, further differential gene expression analysis showed that *Igf1* expression was abundant in OIR compared with RA microglia populations, especially in cluster 9. In line with these observations, immunofluorescence staining showed increased expression of IGF1 in retinal microglia/macrophages within the NV tufts of mice with OIR (Figure 7, C and D). Furthermore, the ELISA also revealed that IGF1 secretion by retinal microglia isolated and cultured from OIR mice was increased by 1.5-fold when compared with that of the RA group (Figure 7E). To explore whether the increased glycolysis in cluster 9 microglia underlies the upregulation of *Igf1*, we used F4/80 antibody–conjugated magnetic beads to isolate mouse retinal microglia (MRM)/macrophages from OIR mice deficient in myeloid *Pkm2*, a key regulator of glycolytic reprogramming in macrophages (37, 38), which was enriched in cluster 9 microglia of OIR retinas. Myeloid-specific *Pkm2*-KO mice (*Pkm2*^Mye-KO^) were generated by intercrossing *Lysm*-Cre mice with *Pkm2*^fl/fl^ mice (Supplemental Figure 7A). qPCR analysis showed that the mRNA expression of both *Pkm2* and *Igf1* were markedly reduced in microglia/macrophages from *Pkm2*^Mye-KO^ OIR mice, compared with expression levels in *Pkm2*^WT^ (*Pkm2*^fl/fl^) OIR mice (Figure 7F). These data confirm the effective deletion of *Pkm2* in the microglia/macrophages of *Pkm2^Mye-KO^* mice and imply that PKM2-driven reprogrammed glycolysis in microglia/macrophages is critical for microglia/macrophage activation and subsequent upregulation of IGF1 production, thereby inducing pathological neovascularization. Indeed, by isolectin B4 staining of the retinal blood vessels, we observed a significant reduction in the area of NVs in the retinas of *Pkm2*^Mye-KO^ mice as compared with WT groups (6.72% ± 0.67% versus 12.67% ± 1.33% of total retina area; Supplemental Figure 7B), consistent with the results in our previous study obtained using WT mice of a different genetic background (19).

Functionally, we further evaluated the angiogenic effect of microglia-derived IGF1 using 2 different ex vivo models of angiogenesis. To this end, primary cultured retinal microglia were transfected with small interfering RNA (siRNA) to *Igf1* (si*Igf1*) or control siRNA (si*Ctrl*) for 48 hours and then cocultured with aortic rings or choroid explants in transwells (Figure 7G). Coculture with retinal microglia induced the sprouting of both aortic rings and choroidal explants as compared with the single aortic ring/choroidal explant culture alone. These proangiogenic effects were reduced when retinal microglia were pretreated with si*Igf1* (Figure 7, H and I). These results suggest that microglia-mediated vessel sprouting and angiogenesis are at least partially dependent on the secretion of the proangiogenic factor IGF1.

### Dynamic transcription transition of microglia in OIR.

**Figure 8:**
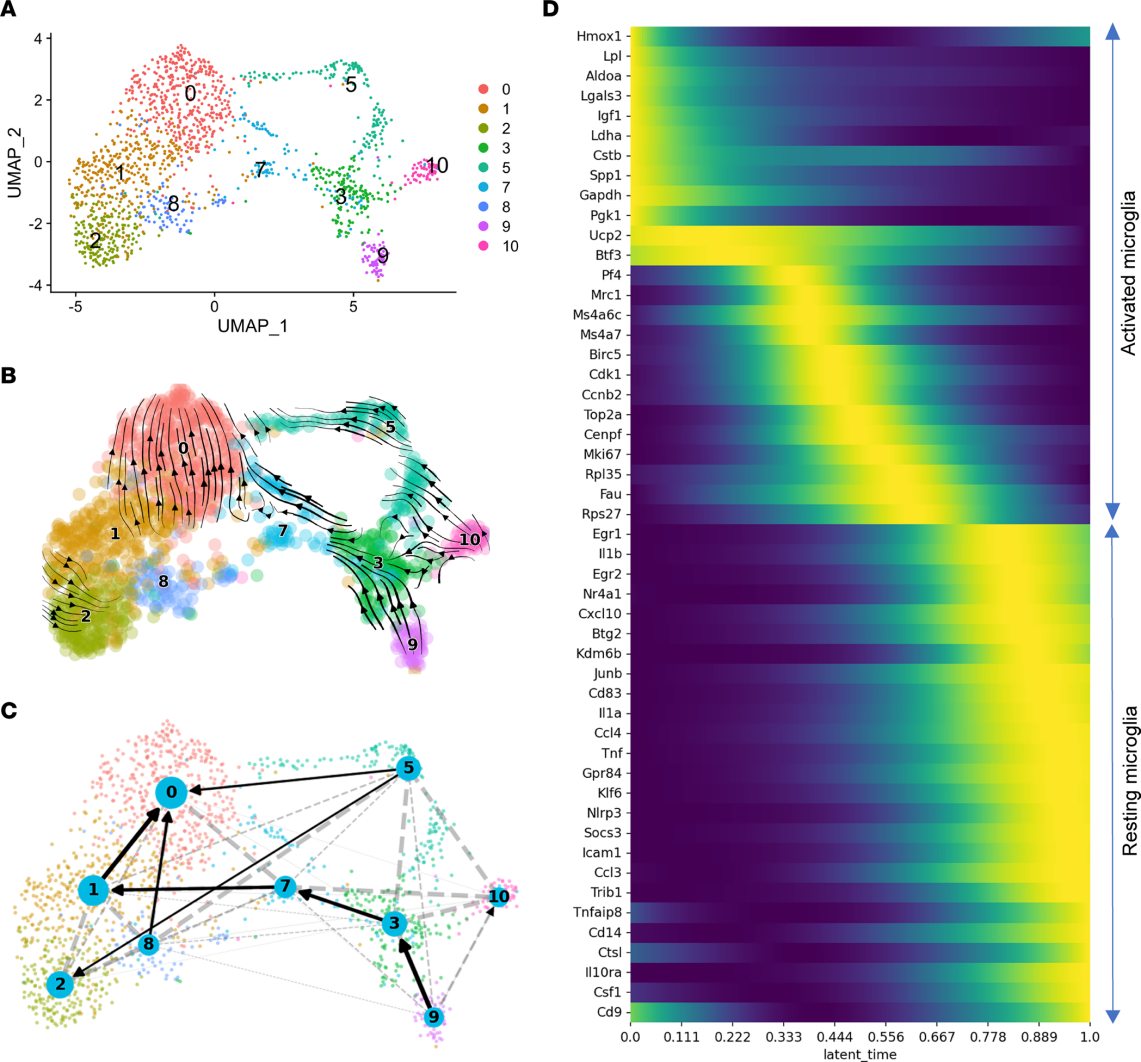
RNA velocity analysis reveals transcriptional dynamics of microglia during OIR. (**A**) The UMAP plot shows 9 distinct clusters among the RA and OIR microglia populations after subsetting and reintegration. Clusters are color coded corresponding to numbers as in Figure 1B. (**B**) The UMAP plot of RNA velocities shows the dynamics and trajectory of mRNA transcription states among RA and OIR microglia. The direction of cell state transition and RNA velocities are projected onto UMAP embedding as streamlines. (**C**) The directed partition-based graph abstraction (PAGA) plot shows the direction of velocity-inferred cell state transitions between clusters. Solid black arrows indicate predicted cell transition directions with high confidence. Dotted lines indicate potential connections between clusters that are suggested by transcriptome similarity but do not have sufficient support by RNA velocity to indicate confident transition. (**D**) Heatmap of 50 latent time-dependent lineage driver genes identified by CellRank shows a trend of gradual gene expression changes along the latent time trajectory. Each column shows gene expression levels at each point in latent time, while each row represents a corresponding gene.

RNA velocity analysis uses the relative abundance of mature (spliced) and newly synthesized (unspliced) transcripts to predict the direction and speed of individual cell transitions between clusters or states in scRNA-Seq data (39). We performed RNA velocity analysis to investigate the heterogenous activation and transition states of microglia in OIR (Figure 8 and Supplemental Figure 8). The RNA velocity vector field was computed using scVelo and overlaid onto the uniform manifold approximation projection (UMAP) (Figure 8, A and B). As shown in Figure 8B, RNA velocity vectors in cluster 9 cells point to cluster 3, while vectors in cluster 3 further point to cluster 7 and eventually toward cluster 0, suggesting a continuous cell state transition trajectory. Conversely, arrows in cluster 10 cells point toward the highly proliferating cluster 5 cells, suggesting that cluster 10 cells could be the source for cycling microglia in OIR. Vectors in clusters 0 and 2 point away from other clusters, indicating their terminal state status. CellRank, a recently developed automatic cell fate mapping algorithm, identified cluster 9 as the initial state or root cells of the differentiation trajectory and discovered only a single lineage (Supplemental Figure 8C). Using the initial and terminal states identified by CellRank, we inferred trajectories between clusters using directed partition-based graph abstraction (PAGA) (Figure 8C). This analysis confirmed that retinal microglia in OIR were ordered along a differentiating trajectory from an activated state returning to a resting state as follows: cluster 9 → cluster 3 → cluster 7 and cluster 1 → cluster 0. The directed PAGA analysis also suggests that cluster 5 proliferating cells probably can directly convert to cluster 0 or cluster 2 resting microglia. These findings support the notion that cluster 9 was the most activated microglia subpopulation, while cluster 3, which expressed intermediate levels of microglial activation markers, may correspond to an intermediate activated state and will eventually restore to resting state if OIR was spontaneously recovered. Understanding the process and adaptive mechanism of retinal blood vessel remodeling and regression after an ischemic injury is critical for the treatment and prognosis of neovascular retinal diseases, since patients with ROP often spontaneously recover (1, 25, 26). By comparing the dynamics of the resting/activating microglia marker genes among the microglia subpopulations corresponding to the different activation states, we noticed that activated microglia markers in clusters 3 are upregulated. However, at the same time, the microglial resting/homeostatic genes, such as *P2ry12*, *Tmem119*, *Siglech*, and *Serinc3*, are still partially preserved (Figure 2). The latent time (dynamic model) and diffusion pseudotime (similarity based) computed by scVelo based on CellRank-identified initial and terminal states demonstrated increased latent time and pseudotime from clusters 3, 5, 9, and 10 toward clusters 0, 1, and 2 (Supplemental Figure 8D). We also performed differential trajectory analysis using the slingshot package (Supplemental Figure 9, A–C). By setting OIR microglia as root, slingshot analysis identified a single trajectory from OIR toward RA microglia (Supplemental Figure 9A). There is a clear difference in the differential distribution of pseudotime between OIR and RA microglia, with a majority of RA microglia having longer pseudotimes (Supplemental Figure 9B). CellRank identified 543 lineage driver genes (FDR < 0.05) whose relative expression levels correlate best with the probability of reaching terminal states. Figure 8D shows a heatmap of 50 linage driver genes whose relative expression levels are ordered along latent time, including cell proliferation–related genes (i.e., *Cdk1* and *Mki67*), glycolytic genes (i.e., *Gapdh*, Ldha, and *Pgk1*), ribosome genes (i.e., *Rpl35* and *Rps27*), as well as transcription factors (i.e., *Btf3*, *Nr4a1*, *Egr1*, and *Egr2*) that might play an essential role in the activation process of microglia in OIR. As shown in Supplemental Figure 8E, the gene trend plots predicted the expression trajectory of cluster-specific marker genes for cluster 9 (*Gapdh* and *Aldoa*), cluster 5 *(Birc5* and *Top2a*), cluster 10 (*Mrc1*), and clusters 0, 1, and 2 (*Ccl4*, *Icam1*, and *Klf6*).

**Figure 9:**
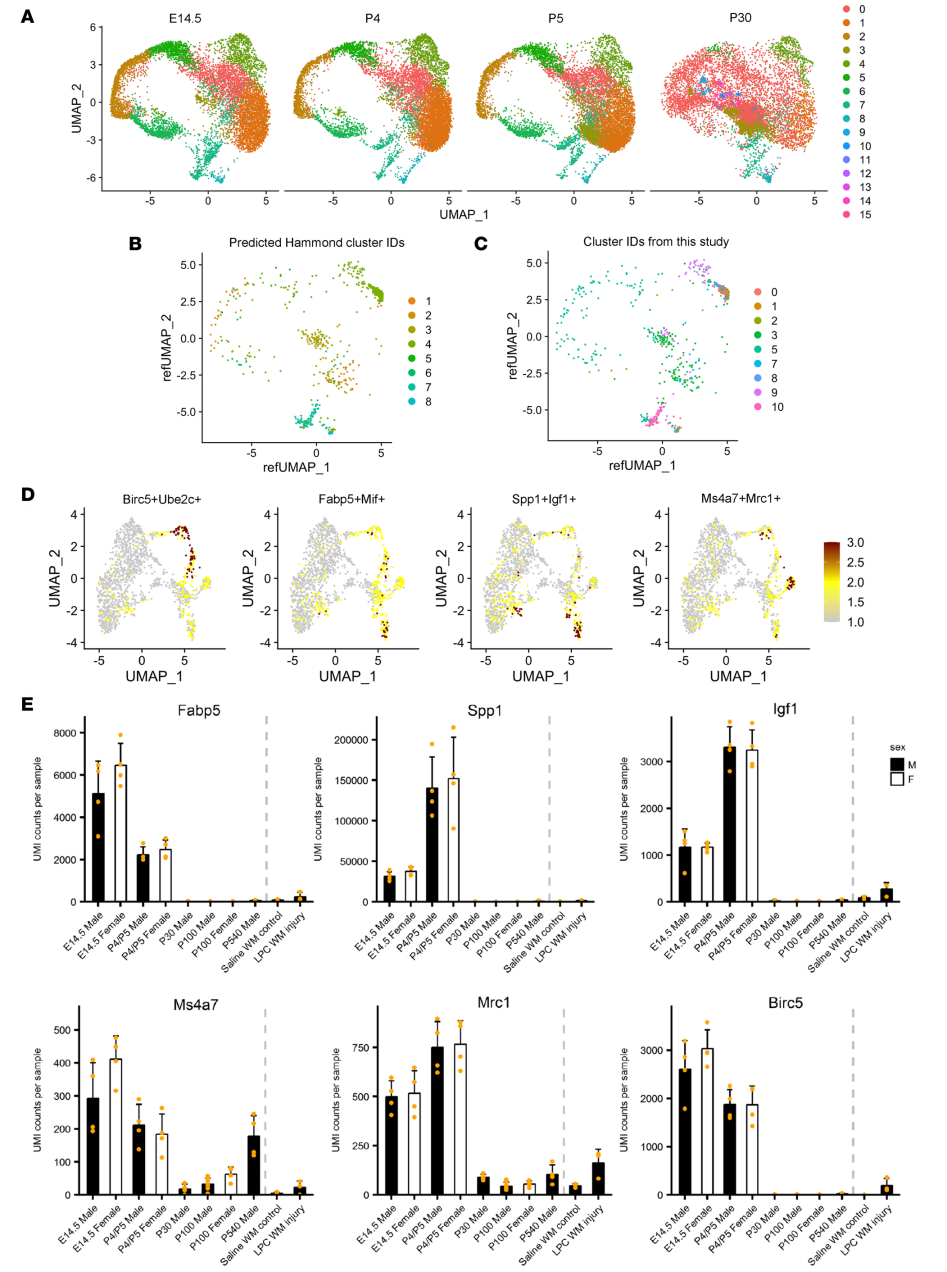
OIR and RA microglia subtypes can be identified in microglia populations from E14.5, P4, P5, and P30 mouse brain. (**A**) UMAP plot of microglia from E14.5, P4, P5, and P30 mouse brain (22). (**B**) UMAP plot of OIR and RA microglia after reference mapping and label transferring. The OIR and RA microglia were used as query data, and microglia from Hammond et al. (22) were used as the reference data set. Seurat V4 reference mapping function was used. Cells are labeled by predicted cluster names in **A** and colored accordingly. (**C**) UMAP plot of OIR and RA microglia after reference mapping and label transferring. Cells are labeled by the cluster names as in Figure 1B and colored accordingly. (**D**) UMAP plots showing gene signature AUC scores of several mouse brain microglia subpopulations from ref. 22 in OIR and RA microglia. (**E**) Bar plots showing the gene expression of subpopulation-specific markers in mouse brain microglia at different ages and upon injury from ref. 22. UMI counts, which represent expression levels of corresponding genes in microglia from embryonic (E14.5), postnatal early development (P4/P5), and young (P30), adult (P100), and aged (P540) mouse brain are shown. Data for control and injury microglia isolated at day 7 from saline-injected and lysolecithin-injected mouse white matter (P100) are shown to the left of the dashed gray lines. Plots were generated from https://www.microgliasinglecell.com.

Together, the RNA velocity and pseudotime analyses provide insight into the activation and maturation trajectories of microglia in OIR. To determine if the predicted differentiation trajectories are biologically meaningful, we compared the retinal microglia data from our study with a scRNA-Seq data set of more than 76,000 microglia throughout a mouse’s lifespan (22). Using the reference mapping approach in the Seurat package, we compared the OIR and RA microglia with brain microglia from E14.5, P4, P5, and P30 (Figure 9, A and B, and Supplemental Figure 10). Interestingly, clusters 9, 10, 3, and 5 were mapped to distinct populations in different stages of brain development, while clusters 7, 8, 0, 1, and 2 were clumped together to form a tight cluster. Using the cluster-specific marker genes identified by Hammond et al. (22), we calculated the gene signature enrichment AUC score using the AUCell package (Figure 9D). Both reference mapping (Figure 9, B and C) and gene signature enrichment analysis (Figure 9D) suggest that cluster 10 microglia belong to the *Ms4a*-expressing microglia predominantly from E14.5 compared with those from any other age (Figure 9E). This subpopulation of microglia is also defined by *Mrc1* and *Ccr1* expression. Cluster 9 microglia are similar to 2 metabolically active microglia subpopulations that are more abundant in the brain of E14.5 and P4/P5 mice: the *Fabp5*^+^*Mif*^+^ microglia almost exclusively found in E14.5 and the *Spp1*^+^ axon tract–associated microglia (ATM) mainly found in P4/P5 mice (Figure 9, D and E). The *Fabp5*^+^*Mif*^+^ and *Spp1*^+^*Igf1*^+^ subpopulations of brain microglia share many marker genes such as *Fabp5*, *Mif*, *Spp1*, *Igf1*, *Gapdh*, *Ldha*, *Tpi1* and *Pkm*; however, they are expressed at different levels (22). Pathway analysis showed that the metabolic profile of *Fabp5*^+^*Mif*^+^ microglia tilted more significantly toward glycolysis (22), which is similar to the OIR retinal microglia in cluster 9. Cluster 5 is similar to the proliferating microglia that are abundant in E14.5 but gradually decrease in frequency during brain development and become a minor microglia population by age P30 and beyond (Figure 9, D and E). Hammond et al. suggest that the expansion of microglia is fueled by these metabolically active and proliferative microglia populations in early brain development (22). The preactivated and resting retinal microglia (clusters 7, 8, 1, 2, and 0) are similar to the *Ccl4*^+^*Ccl3*^+^ brain microglia, which are present in P4/P5 mice but significantly increased in numbers in the older brain (Figure 9, B and C). Cluster 3 microglia are similar to a population that increases in its frequency from early development to P30 mice (Figure 9, B and C). Overall, these comparative analyses, thus, support the velocity and peusdotime analysis results that those activated microglia in clusters 10, 9, and 5 are the microglia precursor cells that drive the expansion and metabolic activation of inflammatory microglia in OIR.

## Discussion

The transitional state and functional changes of immune cells are frequently associated with the onset and progression of numerous neurological diseases, including retinal disorders. Nonetheless, the differential roles of tissue-resident microglia, as well as infiltrating blood-derived immune cells, have been debated. Accumulating evidence has revealed that the embryonic origin of microglia, infiltrated monocytes, and macrophages are different in the CNS of adult mice (9, 40, 41). Moreover, recent studies have demonstrated that resident microglia constitute the predominant retinal MP population in both normal and OIR retinas (7, 10, 11). The specific role of resident microglia in retinal diseases has, therefore, attracted considerable attention. Microglia have been reported to contribute to various diseases of the CNS, including Alzheimer’s disease (AD) (8), other degenerative diseases of the brain (23), retinal degenerative disease (42), and RNV (7). However, defining the specific role of microglia in the retina has proven to be difficult, as it remains technically challenging, in part due to the phenotypic overlap between microglia and macrophages. For example, both microglia and macrophages express the cell surface markers CD11b, CD45, F4/80, and IBa1. A standard method to target microglia is to utilize cell-specific reporter mice such as *Cx3cr1*^GFP-CreERT2^, *Cx3cr1*^YFP-CreERT2^, or *Cx3cr1*^CreERT2^ Rosa26-tdTomato mice. Nonetheless, this method is not without its critics, since retinal macrophages have also been reported to express considerable levels of *Cx3cr1* (43, 44), as we also demonstrate in this study. Surprisingly, our data show that the mRNA expression level of *Cx3cr1* correlated negatively with microglia activation, and its expression was barely detectable in the most activated microglia, indicating that the use of *Cx3cr1* as a microglia marker and proxy for the activation status of microglia under certain pathologies should be evaluated with caution. Most importantly, the traditional techniques (marker-based approaches) of combining microglial cellular surface markers with FACS at the bulk cell level are incapable of accurately analyzing distinct microglial subpopulations implicated in retinal homeostasis and disease. The introduction of scRNA-Seq technologies, particularly the 10X Genomics platform, has revolutionized our ability to study microglia. Individual cells are characterized by their transcriptome rather than cell-surface markers, resulting in unprecedented resolution for identifying novel and sometimes rare cell populations.

In the current study, we utilized high-throughput scRNA-Seq to investigate cellular diversity in the OIR model, a well-recognized mouse model for PR. We found that retinal microglia were highly heterogeneous and composed of diverse subtypes during OIR. Moreover, our study uncovered a variety of unique microglia subpopulations associated with OIR, termed PRAM, and defined their molecular, metabolic, and functional characteristics, as well as their transcriptional dynamics, during activation. Using immunofluorescence staining, we revealed that PRAM are spatially localized near the sites of RNV, indicating their involvement in PR. Further analysis of the genetic signature of PRAM uncovered the upregulation of several metabolic pathways, in particular glycolysis, which may reflect higher demand for energy to support their activation and renewal during OIR. Among the PRAM subpopulations, the combination of volcano plot, tSNE, and clustering with metabolic pathway activities revealed that cluster 9 is a unique subset of PRAM that expresses the highest level of activated microglia marker genes, as well as glycolysis-related genes. We, therefore, anticipated that these highly metabolic and activated PRAM might generate proangiogenic factors that facilitate the abnormal growth of retinal vessels during OIR. In line with this hypothesis, IGF1 was markedly upregulated at both the mRNA and protein levels. Conversely, significant upregulation of other proangiogenic growth factors, such as *Vegfa* and *Hgf*, which have been proposed as potential mediators of RNV and choroidal neovascularization (CNV) (45), did not occur in these PRAM. Importantly, ex vivo functional analysis demonstrated the robust angiogenic effects of retinal microglia-derived IGF1 on both aortic and choroidal vessel sprouting. These findings support the recent observation that IGF1 is upregulated following hypoxia in microglia and plays a vital proangiogenic role in Basigin-2–mediated choroid-retinal endothelial cell tube formation in vitro (46), as well as in the OIR mouse model in vivo (47). The induction of IGF1 in OIR is dependent on PKM2-mediated glycolysis. *Pkm2* is one of the top metabolic gene markers in cluster 9. Retinal microglia isolated from *Pkm2*^Mye-KO^ mice expressed a lower level of *Igf1* mRNA, compared with WT microglia. Congruent with these observations, we have previously demonstrated in a mouse model of OIR that pathological neovascularization was dramatically decreased in *Pkm2*^Mye-KO^ mice compared with control mice (19).

We also discovered a highly proliferative microglia subset, cluster 5, mainly found in the pathological NV tufts of the OIR retina. This subset shares a characteristic molecular signature supporting microglial activation, cell cycle, DNA replication, purine and glucose metabolism, and chromatin modifications. Using FACS and IHC, we assessed the number of CD11b^+^F4/80^+^ cells in the RA and OIR retinas and detected a substantial increase of MPs in the OIR retina, particularly within the areas of RNV tufts at P17. Our findings strongly suggest that the origin of the increased CD11b^+^F4/80^+^ cells in the OIR retina is at least partly attributed to increased cell proliferation. Although the association between retinal microglia proliferation and RNV is disputed (48, 49), our results show a correlation between the number of PRAM and the severity of PR. Thus, it remains to be determined whether inhibition of microglia proliferation could attenuate disease progression and whether targeting the microglia-proliferation pathway can be adopted as a preventive or therapeutic strategy against the pathology of PR and other pathological angiogenesis–related diseases.

It is generally accepted that activation of immune cells is associated with metabolic reprogramming, which is required for energy generation and biosynthesis. For example, macrophages and DCs activated with proinflammatory stimuli undergo a metabolic switch from OXPHOS to glycolysis (50), similar to the Warburg effect in cancer cells. In line with this concept, we indeed noticed the enhancement of both glycolysis and the inflammatory response in the OIR microglia of the major subpopulations (clusters 0, 1, and 2) compared with RA microglia of those clusters. Nevertheless, in contrast to the prevailing notion postulated in the literature that proinflammatory immune cells are hyperglycolytic, an important observation in our study was that, under in vivo conditions, activated microglia from OIR retinas exhibit an imbalance between the gene expression profiles of proinflammatory factors and the upregulation of the glycolysis pathway. These data reflect the metabolic heterogeneity and complexity of different microglia subtypes in the context of OIR. This concept expands our knowledge of the complexity of retinal immunometabolism in disease onset and progression and provides an impetus for further investigation into the relationship between a proinflammatory response and metabolic reprogramming in immune cells.

RNA velocity and cell fate mapping analyses discovered a single lineage transition trajectory from activated microglia to resting microglia and identified the hyperglycolytic cluster 9 as the root cells of the trajectory. Both latent time calculated by RNA velocity and pseudotime calculated by cell-to-cell similarity indicated that clusters 5, 9, and 10 have shorter differentiation times than the rest of the clusters, suggesting that these microglia are precursors. The results from a comparative analysis with published scRNA-Seq data of microglia in the embryonic, early postnatal, and aged mouse brains supported the computational predictions. Reference mapping and label-transfer techniques identified distinct brain microglia subpopulations corresponding to clusters 5, 9, and 10. The corresponding *Fabp5*^+^*Mif*^+^ (cluster 9), *Ms4a7*^+^*Mrc1*^+^ (cluster 10), and *Birc5*^+^*Ube2c*^+^ (cluster 5) brain microglia populations are significantly more abundant in the E14.5 sample, suggesting their early lineage status. Although the frequency of these brain microglia precursors decreases as mice age, they frequently reemerge under pathological conditions. For example, embryonic *Mrc1*^+^ microglia precursors (cluster 10) preferentially expand during pathological states in development (51). *Birc5*^+^ proliferative microglia are frequently present in activated microglia called disease-associated microglia (DAM) (22), while *Spp1* and *Lpl*, which are both markers of cluster 9, are frequently upregulated in DAM. Therefore, it is likely that, in the OIR model, activated retinal microglia such as cluster 9 cells were reprogrammed to an immature state or that residential immature microglia such as cluster 10 were expanded in OIR.

In summary, our results demonstrate a heterogeneity of retinal microglia in response to the microenvironment of pathological angiogenesis. We identify and characterize microglia types and identify genetic markers, spatial localization, signaling pathways, and transcriptional dynamics associated with these types of cells. Suppression of specific PRAM activation/populations represents a potential therapeutic strategy for the treatment of ROP and other ocular disorders associated with microglia-driven RNV (e.g., PDR) or CNV (e.g., age-related macular degeneration).

## Methods

### Animals.

Floxed *Pkm2* (*Pkm2*^fl/fl^) mice from The Jackson Laboratory (stock no. 024048) were crossed with *Lysm*-Cre transgenic mice to obtain *Pkm2*^flox/+^;*Lysm*-Cre heterozygous breeders and, ultimately, *Pkm2*^fl/fl^;*Lysm*-Cre homozygous KO mice (*Pkm2*^Mye-KO^) and littermate control mice (*Pkm2*^fl/fl^, *Pkm2*^WT^). All mice were on a C57BL/6J background.

### Mouse model of OIR.

The OIR model was described previously (24). Briefly, 7-day-old (P7) mouse pups along with their foster/nursing mothers were exposed to 75% oxygen for 5 days to induce vaso-obliteration. At P12, the mice were returned to RA (21% oxygen) to induce RNV, which was maximal at P17. Age-matched mice kept in RA served as the RA controls. Underdeveloped neonatal mice with very low body weight (less than 5 grams at P17) were excluded.

### FACS.

FACS was performed according to protocols described previously with some modifications (11). Whole retinal cells dissected from the retinas of RA and OIR mice were resuspended in HBSS (Thermo Fisher Scientific), 5% FBS (MilliporeSigma), 10 mM HEPES (MilliporeSigma), 0.7 mg/mL calcium chloride (CaCl_2_; MilliporeSigma), 1.5 mg/mL Collagenase A (10103586001, Roche), and 0.1 mg/mL DNase I (10104159001, Roche) for 30 minutes at 37°C. Following incubation, each retina was gently dissociated and processed into a single-cell suspension and then resuspended in PBS (Thermo Fisher Scientific) containing 5% normal mouse serum (Thermo Fisher Scientific) and 5% normal rat serum (Thermo Fisher Scientific). Cells were stained for viability (7-AAD, 420403, BioLegend), incubated with Fc block (553141, BD Biosciences), and then stained with PerCP-Cy5.5 anti–mouse CD11b (2 μg/mL, 561114, BD Biosciences), PE anti–mouse F4/80 (3 μg/mL, 565410, BD Biosciences), and the matching control isotype IgGs (BD Biosciences) for 30 minutes at 4°C. The cells were then washed with FACS buffer (138 mM NaCl, 2.67 mM KCl, 8.06 mM Na_2_HPO_4_, 1.47 mM KH_2_PO_4_, 0.1% BSA, 0.02% NaN_3_, pH 7.4), centrifuged at 400*g* for 5 minutes at 4°C, and resuspended in FACS buffer. Cells were sorted into 50 μL of ice-cold FACS buffer supplemented with 5% FBS (Thermo Fisher Scientific) using the FACSAria II SORP (BD Biosciences) at the Georgia Cancer Center — Flow Cytometry Core Facility, gating for live CD11b^+^F4/80^+^ cells. Further analysis was performed using FACSDiva 8.0 (BD Biosciences).

### scRNA-Seq and analysis.

Donor CD11b^+^F4/80^+^ cells sorted by FACS were processed for scRNA-Seq libraries using the Chromium Controller (10X Genomics) and the Chromium 3′ Single Cell mRNA-Seq V3 reagents. The scRNA-Seq libraries were sequenced using an Illumina NextSeq500 sequencer. The raw data were processed using the Cell Ranger package (10X Genomics). The Cell Ranger pipeline outputs were analyzed using Seurat v4.0 (52). Quality control measures were implemented in Seurat to filter out cells expressing a high number of genes (top 2 percentile, assuming a 2% doublet rate) and/or with a higher percentage of mitochondrial genes (>20%). OIR and RA data sets were then integrated using the canonical correlation analysis–based (CCA-based) integration approach in Seurat to remove batch effects. In total, 3,000 anchor genes were identified using Seurat FindIntegrationAnchors function after normalization. The IntegrateData function merged the 2 data sets. Subsequent analyses such as PCA, UMAP, tSNE, and clustering were performed in the integrated assay using the 3,000 anchor genes. Differential expression analysis was performed using the Seurat FindMarkers or FindAllMarkers functions using log_2_ normalized and scaled data before integration. Cell type was assigned to each cluster based on the top makers identified in each cluster. Several clusters of nonimmune cells that do not express CD45, which likely belong to nerve, endothelial, blood vessel, and photoreceptor cells, were excluded from downstream analysis. Cell cycle scores were calculated using a gene signature containing 97 cell cycle–related genes (34, 53).

### RNA velocity analysis and cell fate mapping.

RNA velocity analysis was performed using the Python package scVelo (54), which uses a likelihood-based dynamical model to relate pre-mRNA abundance to the abundance of mature mRNA. The BAM files were preprocessed using the velocyto command line tool with default parameters specific to the 10X Genomics scRNA-Seq data (39). RNA velocity of microglia (excluding clusters 5 and 6, which represent circulating monocytes and macrophages) was estimated using scVelo, and the velocity vector fields were visualized on UMAP embedding from the Seurat object. The automatic cell fate mapping was performed using CellRank (55). First, the terminal and initial states were determined using the cr.tl.terminal_states and cr.tl.initial_states functions, respectively. The fate maps were computed to define the probability that each cell would transition toward the identified terminal states. The latent time and diffusion map pseudotime was calculated using scVelo based on the CellRank-identified initial and terminal states. Subsequently, the individual fate maps were aggregated to cluster-level fate maps by the directed PAGA (55). The lineage driver genes, whose expression levels correlate with lineage probability, were identified using the cr.tl.lineage_drivers function. Gene expression trends along latent time were calculated using a general additive model (GAM) with the cr.pl.gene_trends function. Heatmaps of the 50 lineage driver genes whose expression levels correlated with the lineage were plotted using the cr.pl.heatmap function.

### Slingshot pseudotime trajectory analysis.

OIR and RA microglia were analyzed for differential pseudotime trajectory inference using the slingshot package (56). The postprocessing Seurat integrated object was saved as a single-cell experiment object and analyzed using slingshot directly with the sample names serving as the cluster identifier. The OIR microglia were selected as the start cluster when ordering cells along the pseudotime trajectory.

### Pathway and gene signature enrichment analysis.

The pathway enrichment analysis of differentially expressed genes between clusters or groups was performed using clusterProfiler v4.0 R package (57). The single-cell level pathway analysis was performed using AUCell v1.16.0 R package (27). Metabolic pathway gene sets were downloaded from the WikiPathway collection and used to calculate the pathway activity score, represented as the AUC score, indicating whether a critical subset of the input gene set is enriched within the expressed genes for each cell. The AUC score matrix was imported into Seurat to generate violin, tSNE, and UMAP plots. Heatmaps of metabolic activities of single cells were generated by the ComplexHeatmap package. The cnetplot, which depicts the linkages of genes and biological concepts was generated using clusterProfiler.

### Gene regulatory network analysis.

To carry out transcription factor network inference analysis, the scRNA-Seq data were analyzed using the SCENIC work-flow (SCENIC v1.2.4, R package) as described (27). After filtering genes either expressed at deficient levels or expressed in too few cells, 9,408 genes in the RcisTarget mm9 motif database were used to derive the coexpression networks with GENIE3 v1.16.0. The gene regulatory networks (regulons) were constructed and scored using RcisTarget v1.14.0 and AUCell v1.16.0, respectively. Regulons annotated as ‘‘extended’’ include target genes harboring motifs linked to the respective transcription factor by lower confidence annotations (27); when a pass-filter standard regulon is available, the extended regulon is not used. Regulon-specific scores were calculated for each cluster using the regulon_specificity_socre function. The regulon scoring matrix was imported into Seurat to generate violin, tSNE, and UMAP plots. ComplexHeatmap was used to generate heatmaps of regulon activities.

### Analysis of brain microglia data sets.

The scRNA-Seq data sets of microglia of E14.5, P4/P5, and P30 consisting of 22 individual samples from Hammond et al. (22) were downloaded from Gene Expression Omnibus (GEO; GSE121654). The raw count matrix data were first processed using a code published by Green et al. (51) ( https://github.com/michael-r-odea/Green_ODea_2022/commit/a9b732d2bf40a8527ac2d1800f53876bb9be1a24). A cleaned and filtered Seurat object consisting of only microglia was downsampled so that each age group had the same number of cells as the P30 group, which had the least number of cells. Each sample was normalized using SCTransform function and integrated using Seurat IntegrateData. Then, the integrated brain microglia object was used as a reference data set. The OIR/RA retinal microglia Seurat object was used as a query data set for projecting the query onto the reference UMAP embedding. The anchor genes were identified by FindTranferAnchors function. The predicted cluster names and reference UMAP projection was computed using MapQuery function. The cluster-specific gene signatures were derived by filtering the marker gene list published by Hammond et al. (cutoff values: adjusted *P* < 0.05, log_2_ fold change >1, and percentage of cells [pct] >0.15; ref. 22). The gene signature enrichment analysis was performed using the AUCell package described above. The UMAP plots of gene signature AUC scores were generated in Seurat after importing AUC scores into the Seurat object. The bar plot of UMI count in brain microglia of different age groups and upon injury was generated using the web tool developed by Hammond et al. (22) (https://www.microgliasinglecell.com).

### Isolation and culture of MRM.

MRM were isolated and cultured according to the protocol described previously by us (19).

### ELISA analysis.

MRM were isolated and cultured from RA and OIR mice at P17. The secretion of IGF1 from cultured retinal microglia was detected using Mouse/Rat IGF-I/IGF-1 Quantikine ELISA Kit (R&D systems, MG100) according to the manufacturer’s instructions.

### RNA interference.

MRM or N9 microglial cells (Otwo Biotech, Shenzhen, HTX1877) were transfected with control nontargeting siRNA, specific mouse *Igf1* siRNAs (Santa Cruz Biotechnology, sc-37194), or specific mouse *Myc* siRNA (Ribobio, Guangzhou, siG13821152313) using Lipofectamine RNAiMax reagent (Invitrogen, 13778-150) or HiPerFect transfection reagent (Qiagen) according to the manufacturer’s instructions. Briefly, siRNAs and Lipofectamine RNAiMax /HiPerFect reagents were dissolved in Opti-MEM and incubated for 20 minutes at room temperature. The siRNA transfection reagent complexes were added to microglia and incubated at 37°C for 6 hours. The complexes were then removed, and fresh complete media were added to the cells. The cells were cultured for an additional 48 hours before use in experiments.

### Aortic ring and choroidal sprouting assays.

Aortic ring and choroidal sprouting assays were performed as described previously (19). MRM were transfected with si*Ctrl* or si*Igf1* for 48 hours. Coculture was performed with a transwell system, with the aortic/choroidal explant cultured at the bottom of the well before adding pretreated MRM to the top of the transwells. Aortic/choroidal explant sprouting was imaged using a Zeiss LSM 780 Inverted Confocal Microscope (Carl Zeiss). The sprouting area was quantified with Adobe Photoshop.

### Fluorescence immunostaining in whole-mount retinas.

Eyes were enucleated and fixed in 4% paraformaldehyde for 2 hours at room temperature. The intact retinas were collected, blocked, and permeabilized in PBS containing 10% goat serum, 3% BSA, 1% Triton X-100, and 0.2% Tween 20 for 1 hour. Samples were then incubated with primary antibodies against rabbit PKM2 (1:100, 4053, Cell Signaling Technology), rabbit Ki67 (1:200, RM-9106, Thermo Scientific), rabbit Fabp5 (1:200, 2.5 μg/mL, RD181060100, BioVendo), rabbit Igf1 (10 μg/mL, AF791, R&D Systems), rat F4/80 (1:100, ab6640, Abcam), rabbit IBa1 (1:400, 019-19741, Sakura Finetek), goat IBa1 (1:400, ab48004, Abcam), rat CD11b (5 μg/mL, 561114, BD Biosciences), and Alexa Fluor 488–, Alexa Fluor 594–, or Alexa Fluor 647–labeled *Griffonia simplicifolia* isolectin B4 (10 μg/mL; catalogs 121411, 121413, and I32450, respectively; Invitrogen) overnight at 4°C, followed by incubation with fluorescence-conjugated cross-adsorbed secondary antibody (1:500, Molecular Probes, Invitrogen) for 1 hour, and then counterstained with DAPI (Invitrogen). Retinas were ﬂat mounted on microscope slides in a mounting medium (Vectashield; Vector Laboratories) and examined by confocal microscopy (Zeiss 780; Carl Zeiss). For all immunoﬂuorescence experiments, parallel groups of tissues were stained with IgG isotype and secondary antibody as negative controls.

### qPCR analysis.

Total RNA of cells was extracted using Trizol Reagent (Invitrogen). The resultant sample of total RNA was utilized as a template for reverse transcription using the iScript cDNA synthesis kit (Bio-Rad, 170-8891). qPCR was performed on a StepOne Plus System (Applied Biosystems) using Power SYBR GreenMaster Mix (Invitrogen, 4367659) with the respective gene-specific primers listed in Supplemental Table 3. Quantification of relative gene expression was calculated with the efficiency-corrected 2^–ΔΔCT^ method using *18S* ribosomal RNA as the internal control. Data were presented as fold change relative to control groups.

### Data availability.

The scRNA-Seq data generated in this study have been deposited to the GEO and assigned the identifier GSE199792.

### Statistics.

Data are presented as mean ± SEM unless otherwise specified. Statistical analysis was performed using GraphPad Prism 8.0. Statistical comparisons were made using a 2-tailed unpaired Student’s *t* test, nonparametric Mann–Whitney *U* test, the Wilcoxon rank-sum test in R, or 1-way ANOVA followed by Bonferroni’s post hoc tests when appropriate. Two-sided *P* values were calculated, and *P* < 0.05 denoted significance.

### Study approval.

Animals were used in this study according to the NIH *Guide for the Care and Use of Laboratory Animals* (National Academies Press, 2011) and in accordance with the protocol approved by the IACUC at Augusta University.

## Author contributions

ZL, RBC, HS, and YH designed the research; ZL, JX, QY, QM, XM, ZX, YZ, QD, and YC performed the experiments; ZL, HS, and YH analyzed the data; ZL, HS, and YH wrote the manuscript; ZL, ZD, DF, AS, RBC, HS, and YH participated in the experimental design and revised the manuscript; and ZL, RBC, and YH provided financial support.

## Supplementary Material

Supplemental data

Supplemental table 1

Supplemental table 2

## Version 1 10/20/2022

In-Press Preview

## Version 2 12/08/2022

Electronic publication
