# Supplementary material for: Single-cell transcriptome analyses reveal microglia types associated with proliferative retinopathy
Source: JCI Insight. 2022 Dec 8;7(23):e160940. doi: 10.1172/jci.insight.160940 (PMC9746914; doi:10.1172/jci.insight.160940)
Supplement: Supplemental data [file jciinsight-7-160940-s221.pdf]

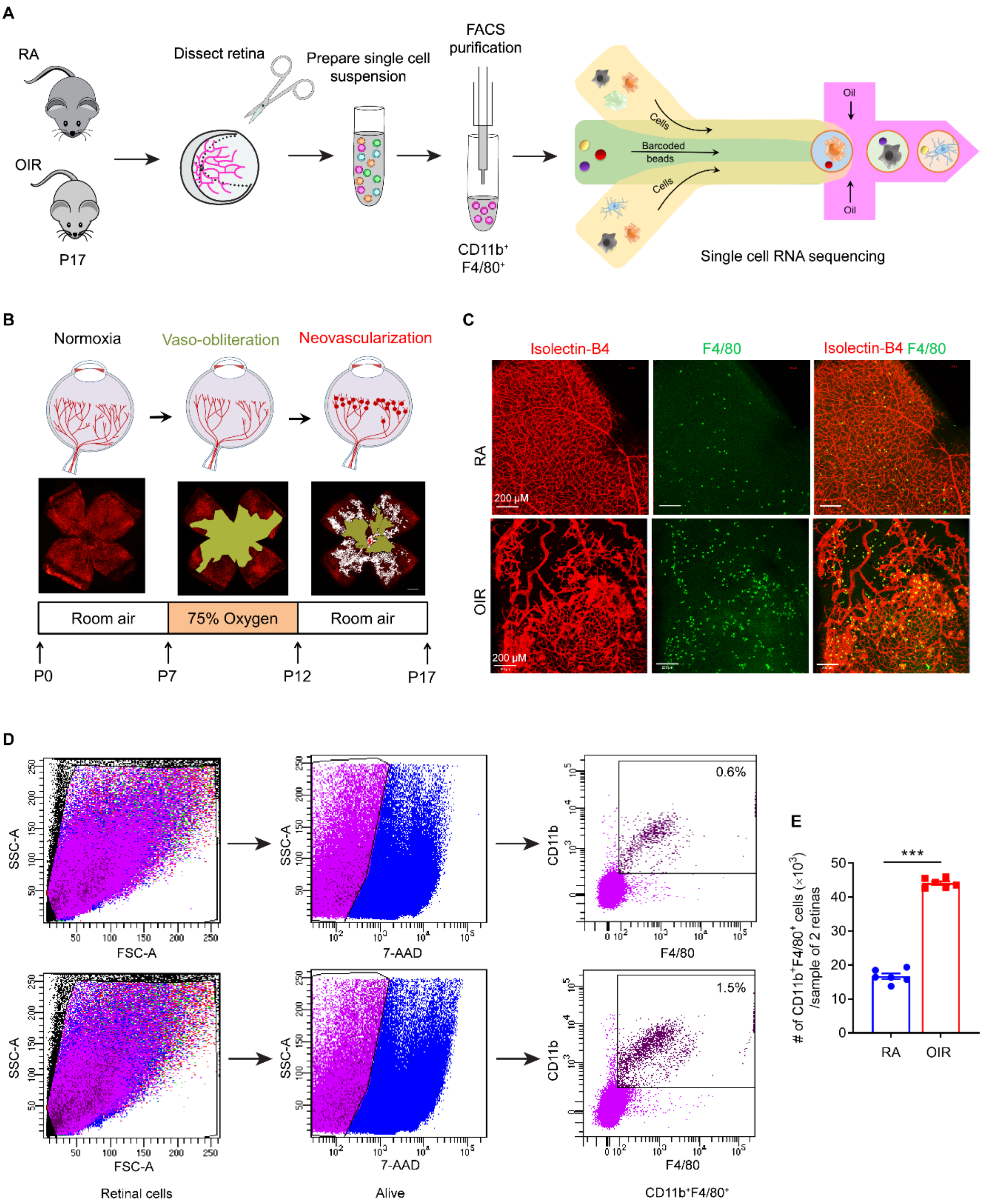

Supplementary Figure 1. Isolation, purification, and single-cell mRNA sequencing of retinal mononuclear phagocytes from RA and OIR mice

(A) Schematic diagram showing the experimental design. Whole retinal single cells were isolated from the whole retinas of RA and OIR mice. $CD11b^{+}F4/80^{+}$ cells were captured by FACS for scRNA-seq analysis.

(B) Schematic illustration of mouse OIR model. Neonatal mice with nursing mothers were exposed to 75% O_2 from postnatal day 7 (P7) to P12, followed by RA with maximum neovascularization (NV) at P17.

(C) Double immunohistochemistry staining of isolectin B4 and F4/80 in retinas of RA and OIR mice at P17. Scale bars, 200 μm. n = 6.

(D) Representative FACS plots showing the cells captured for scRNA-seq.

(E) RA and OIR retinas were collected at P17 for FACS analysis. The number of $CD11b^{+}F4/80^{+}$ cells was calculated according to the proportion measured by FACS analysis. n = 6 (total of 12 retinas per condition; each "n" comprises 2 retinas). ***P < 0.001. Data are mean ± SEM. Statistical significance was determined by Student's *t*-test.

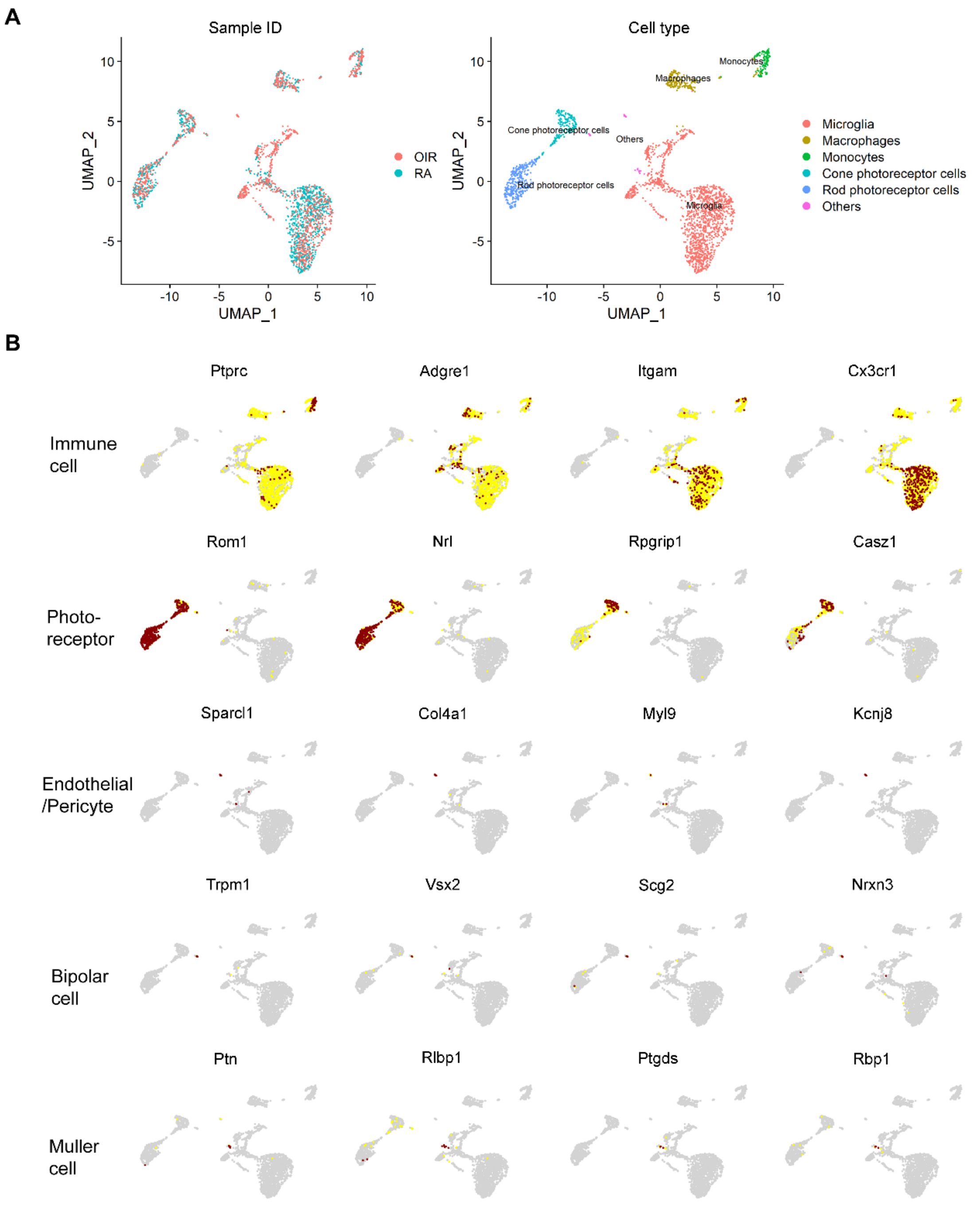

Supplementary Figure 2. Identification of immune cells in the scRNA-seq data of RA and OIR retinas.

(A) UMAP plots for all 2708 cells from RA and OIR retinas with sample name (left panel) and cell type (right panel) indicated.

(B) UMAP plots showing the expression levels of cell type-specific marker genes for immune cells, cone/rod photoreceptor cells, and other types of cells.

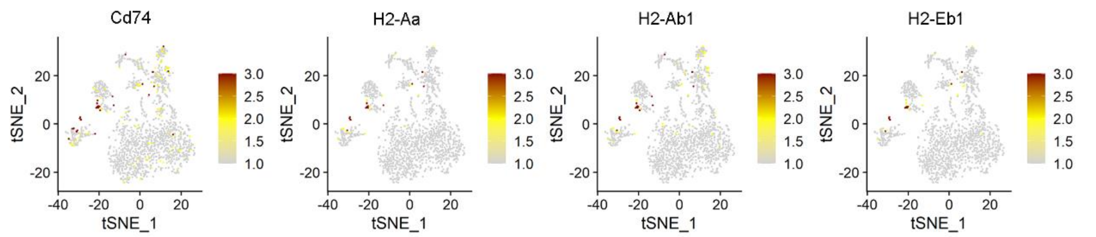

Supplementary Figure 3. Identification of monocyte-derived macrophages during OIR by scRNA-seq. tSNE plots showing the expression of canonical marker genes of monocyte-derived macrophages among the 11 clusters of CD11b$^+$F4/80$^+$ cells.

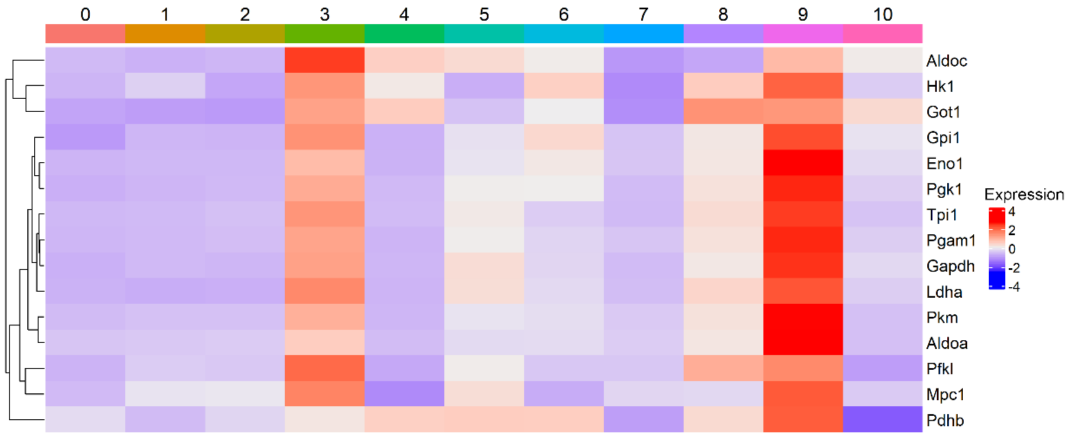

Supplementary Figure 4. Heatmap showing the average expression of genes in the glycolysis and glycogenesis pathways in each cluster.

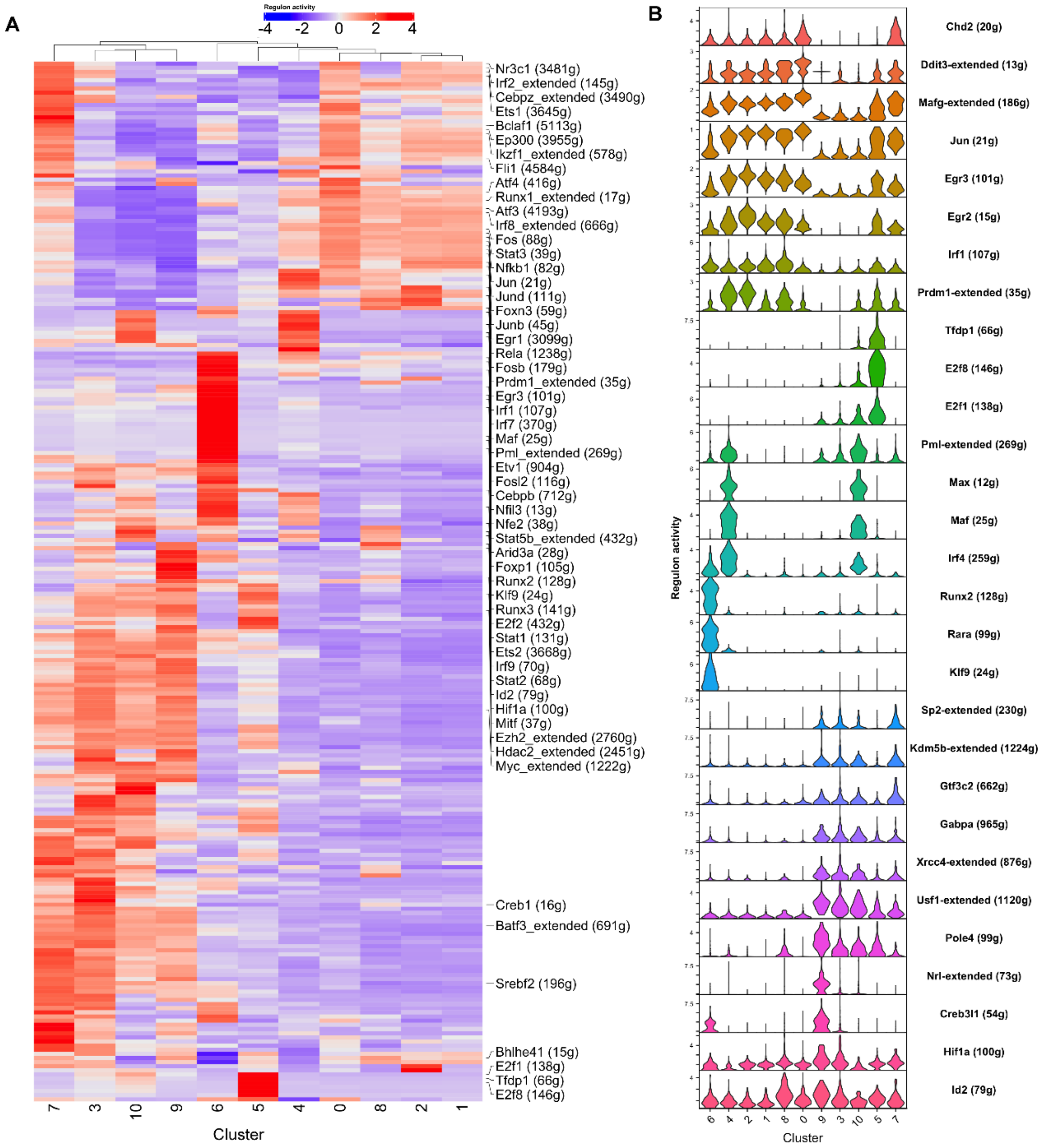

Supplementary Figure 5. Single-cell transcriptional regulatory network analysis in RA and OIR retinas.

(A) Heatmap of average transcription factor regulon activities in each cluster. The columns represent clusters, and the rows represent regulons. The transcription factor regulon activity scores are determined by Single-Cell rEgulatory Network Inference and Clustering (SCENIC) package.

(B) Violin plots showing the regulon activities of top 3 cluster-specific transcription factors of each cluster in $CD11b^{+}F4/80^{+}$ cells.

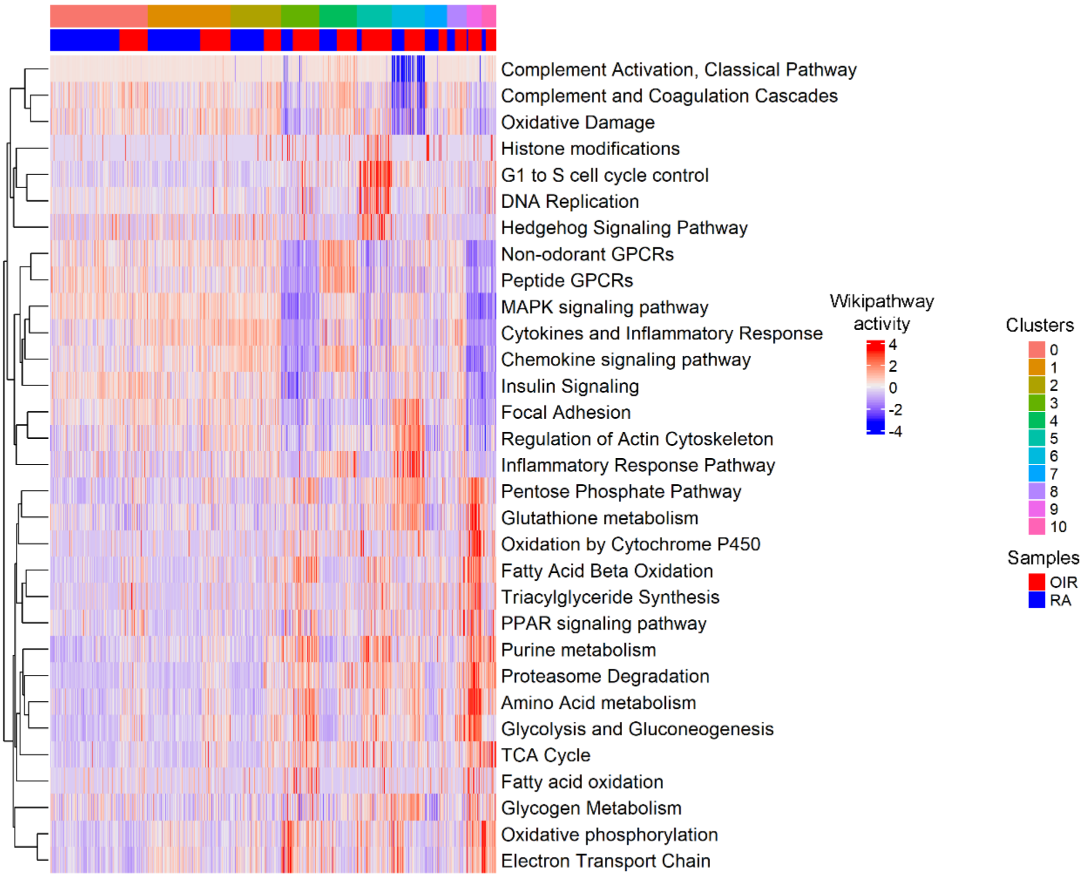

Supplementary Figure 6. Single-cell pathway enrichment analysis in RA and OIR retinas. Heatmap showing WikiPathways activity scores calculated by AUCell package in single cells. The columns represent single cells and the rows represent pathway AUC scores.

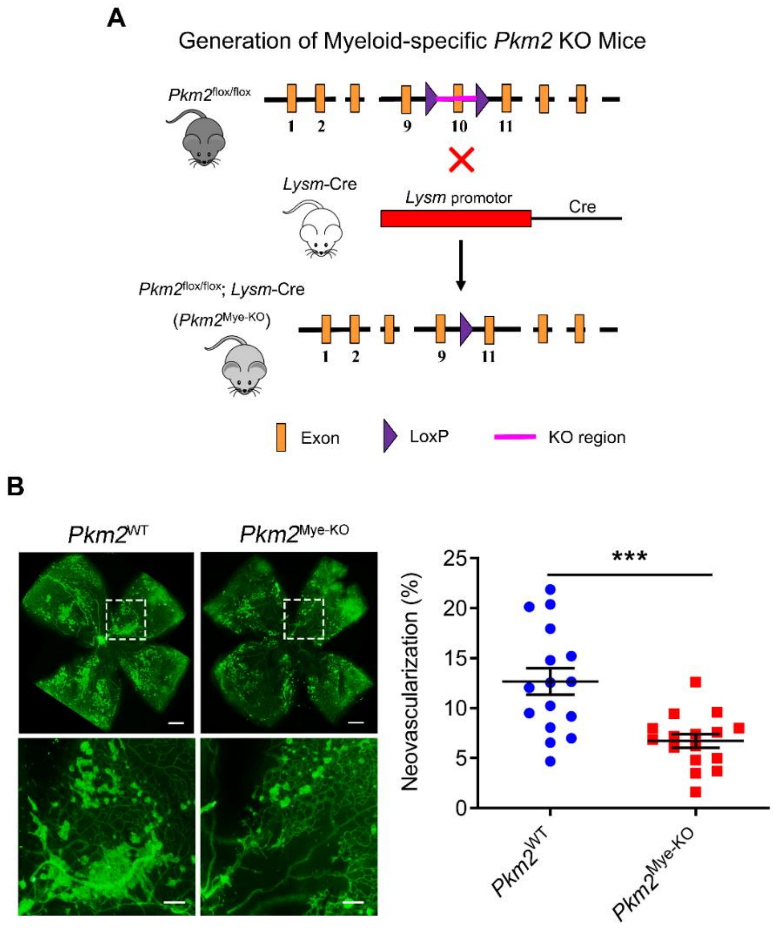

Supplementary Figure 7. Myeloid *Pkm2* deficiency significantly decreases formation of pathological neovascularization in OIR retinas.

(A) Schematic illustration of the generation of myeloid-specific *Pkm2* knock-out (KO) mice.

(B) Representative retinal whole mounts from postnatal day (P)17-OIR retinas of $Pkm2^{+/+}Lysm^{cre}$ ($Pkm2^{WT}$) and $Pkm2^{flox/flox}Lysm^{cre}$ ($Pkm2^{Mye\text{-}KO}$) mice stained with isolectin B4 (green). Two selected retinal areas (white box) were enlarged to show pathological neovessels. Area of pathological neovascularization is quantified as percentage of total retinal area. n = 16 retinas; Scale bars, 500 µm for original images and 200 µm for enlarged images. Data are means ± SEM. ***$P < 0.001$ by Student's *t* test.

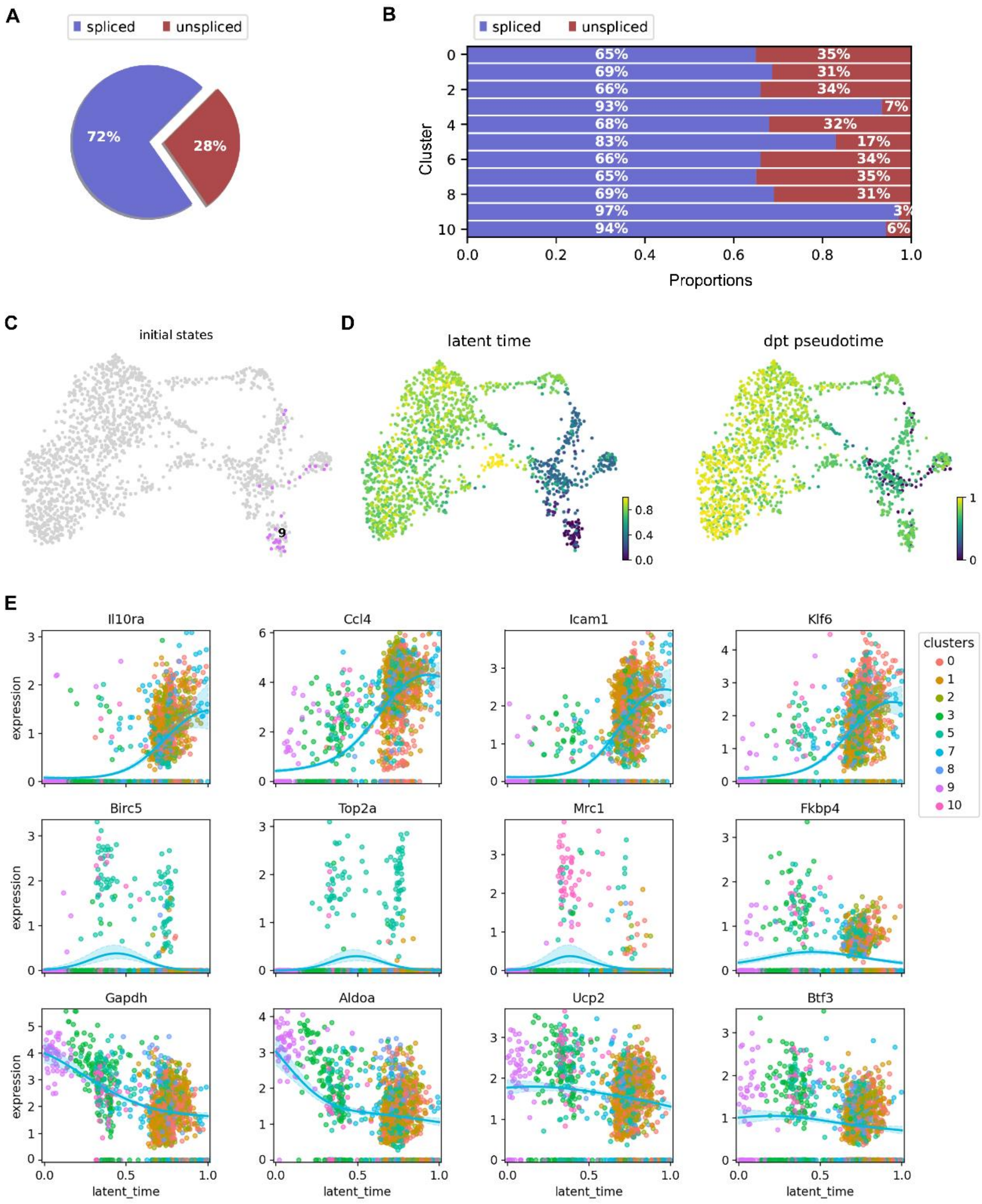

Supplementary Figure 8. RNA velocity analysis reveals transcriptional dynamics of OIR and RA microglia.

(A) Pie chart showing the proportions of spliced and unspliced (intron-containing) sequencing reads in the whole dataset.

(B) Bar chart showing proportions of spliced and unspliced (intron-containing) sequencing reads in each of the 11 clusters of the CD11b$^+$F4/80$^+$ cells.

(C) The initial state identified by the CellRank package. A majority of cells in cluster 9 and a portion of cells in cluster 10 were identified as in the initial state of differentiation trajectory.

(D) UMAP plot of latent time and diffusion map pseudotime calculated by scVelo based on the initial and terminal states identified by CellRank.

(E) Gene trend plots showing the smoothed gene expression toward the terminal populations for a group of lineage driver genes.

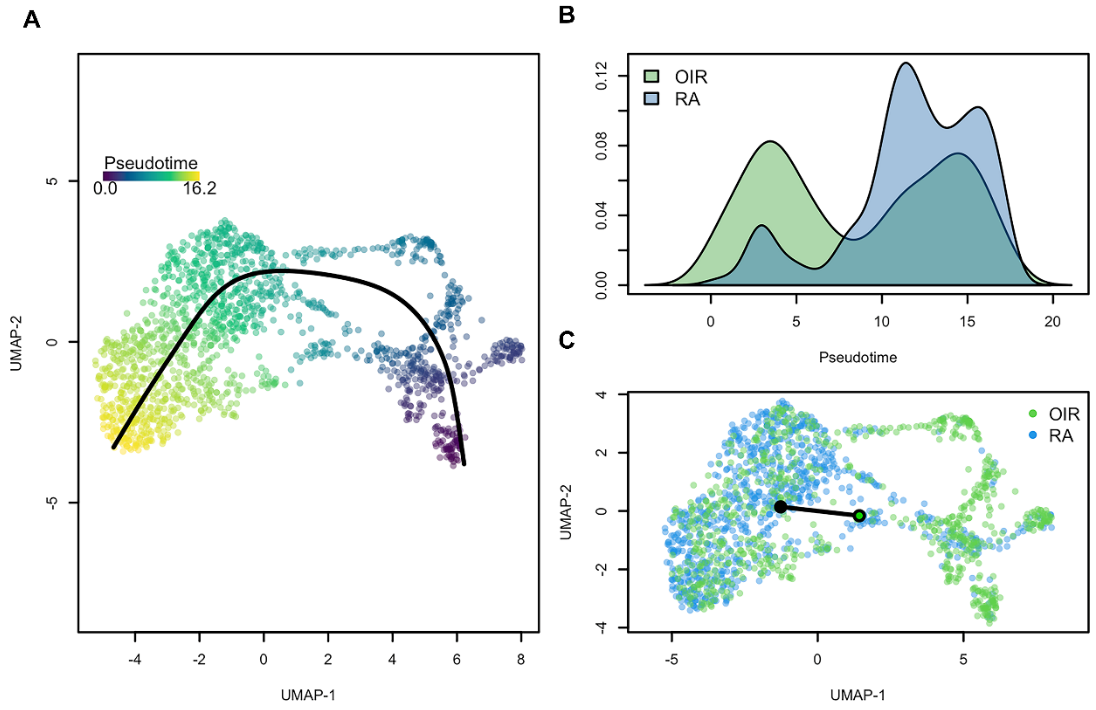

Supplementary Figure 9. Pseudotime trajectory inferred by slingshot package.

(A) UMAP plot of slingshot pseudotime. Each point is a cell and is colored by pseudotime ordering. The fitted principal curve is shown in black.

(B) Comparison between the distribution of slingshot pseudotime between OIR and RA microglia.

(C) The minimal spanning tree generated by slingshot, where the OIR was selected as root cells.

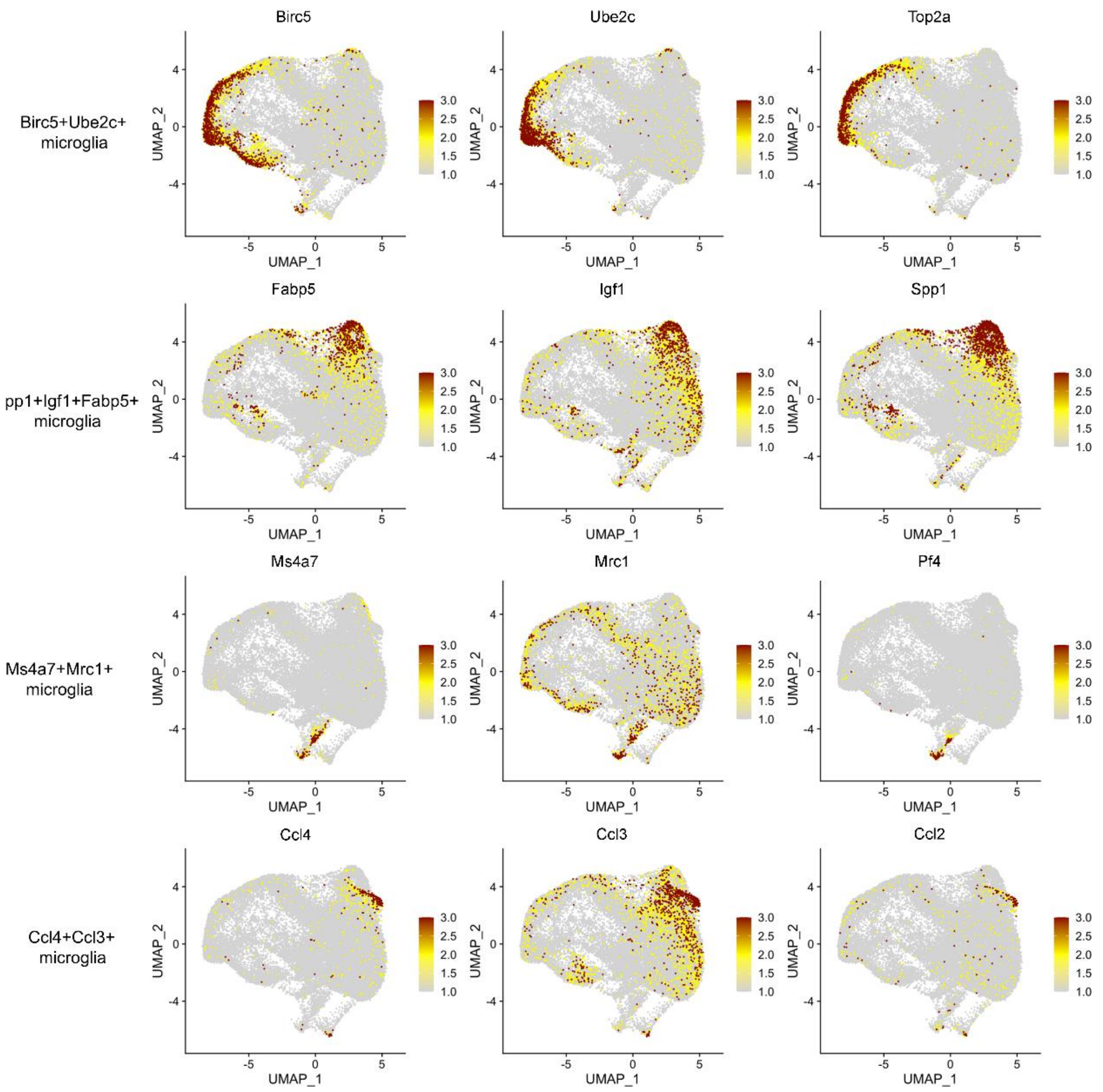

Supplementary Figure 10. OIR and RA microglia subtypes can be identified in microglia populations from E14.5, P4, P5 and P30 mouse brain. UMAP plots showing gene expression patterns of representative marker genes for several indicated microglia subpopulations in E14.5, P4, P5 and P30 mouse brains from Hammond et al.

[See uploaded file]

Supplementary Table 1. List of enriched marker genes of the 11 clusters of $CD11b^{+}F4/80^{+}$ cells.

[See uploaded file]

Supplementary Table 2. List of the top 10 enriched marker genes of the 11 clusters of $CD11b^{+}F4/80^{+}$ cells.

Target gene	Sequence
mouse*18S ribosomal RNA*	F: 5'- CTTAGAGGGACAAGTGGCG -3'
	R: 5'- ACGCTGAGCCAGTCAGTGTA -3'
mouse *Gapdh*	F:5'- AGGTCGGTGTGAACGGATTTG -3'
	R:5'- GGGGTCGTTGATGGCAACA -3'
mouse *Pgk1*	F:5'- ATGTCGCTTTCCAACAAGCTG -3'
	R:5'- GCTCCATTGTCCAAGCAGAAT -3'
mouse *Eno1*	F:5'- TGCGTCCACTGGCATCTAC -3'
	R:5'- CAGAGCAGGCGCAATAGTTTTA -3'
mouse *Ldha*	F:5'- ACATTGTCAAGTACAGTCCACAC -3'
	R:5'- TTCCAATTACTCGGTTTTTGGGA -3'
mouse *Pkm2*	F:5'- AGGATGCCGTGCTGAATG -3'
	R:5'- TAGAAGAGGGGCTCCAGAGG -3'
mouse *Pgam1*	F:5'- TCTGTGCAGAAGAGAGCAATCC -3'
	R:5'- CTGTCAGACCGCCATAGTGT -3'
mouse *Igf1*	F:5'- CTGGACCAGAGACCCTTTGC -3'
	R:5'- GGACGGGGACTTCTGAGTCTT -3'
mouse *Fabp5*	F:5'- TGAAAGAGCTAGGAGTAGGACTG -3'
	R:5'- CTCTCGGTTTTGACCGTGATG -3'
mouse *Myc*	F:5'- GCCGATCAGCTGGAGATGA -3'
	R:5'- GTCGTCAGGATCGCAGATGAAG -3'

mouse *Ccl2*	F:5'- CAGGTCCCTGTCATGCTTCT -3'
	R:5'- GTGGGGCGTTAACTGCATCT -3'
mouse *Il6*	F:5'- TCTTGGGACTGATGCTG GTGA -3'
	R:5'- GCAAGTGCATCATCGTT GTTCA -3'
mouse *Il1b*	F:5'- TGTCTTGGCCGAGGACTAAGG -3'
	R:5'- TGGGCTGGACTGTTTCTAATGC -3'
mouse *Tnfa*	F:5'- ACGGCATGGATCTCAAAGAC -3'
	R:5'- AGATAGCAAATCGGCTGACG -3'

Supplementary Table 3. Specific primer sequences for qRT-PCR analyses (F: forward primer; R: reverse primer)
