# Supplementary material for: Single-cell transcriptome analyses reveal microglia types associated with proliferative retinopathy
Source: JCI Insight. 2022 Dec 8;7(23):e160940. doi: 10.1172/jci.insight.160940 (PMC9746914; doi:10.1172/jci.insight.160940)
Supplement: Supplemental table 1 [file jciinsight-7-160940-s222.pdf]

"","p_val","avg_log2FC","pct.1","pct.2","p_val_adj","cluster","gene"
"Ier5",5.31946386757398e-105,1.25549729116785,0.998,0.771,8.9696799735
0324e-101,"0","Ier5"
"Rhob",1.98607106193287e-99,1.40466588793594,0.995,0.763,3.34891302463
121e-95,"0","Rhob"
"Ddx5",9.74315925987136e-92,0.879198847980367,0.995,0.923,1.6428915143
9951e-87,"0","Ddx5"
"Jund",1.67046564594163e-91,1.09584813841618,0.998,0.876,2.81673917218
677e-87,"0","Jund"
"Sertad1",1.28548335297366e-83,1.11734178469083,0.824,0.396,2.16758202
978419e-79,"0","Sertad1"
"Jmjd1c",1.31122732220766e-83,1.19482265632404,0.963,0.66,2.2109915107
0656e-79,"0","Jmjd1c"
"Btg2",6.4041287355696e-81,1.14427944800629,0.998,0.796,1.079864187391
75e-76,"0","Btg2"
"Ccnl1",3.43566952212195e-76,1.02500144597659,0.983,0.711,5.7932259482
0202e-72,"0","Ccnl1"
"Junb",1.88723150020698e-74,0.880039340517231,0.998,0.859,3.1822497556
4901e-70,"0","Junb"
"Hmox1",1.02986994891003e-71,0.568322058502597,0.961,0.673,1.736566707
85209e-67,"0","Hmox1"
"Ifrd1",2.62663392186684e-70,1.14827473258941,0.983,0.704,4.4290301190
5186e-66,"0","Ifrd1"
"Fos",1.39532168308993e-67,1.0039284884438,0.993,0.817,2.3527914220262
4e-63,"0","Fos"
"Rrad",6.1706411611102e-66,1.61624694743704,0.745,0.357,1.040493512586
4e-61,"0","Rrad"
"Ubc",2.23284831030313e-65,0.891595489493831,1,0.925,3.76502882083314e
-61,"0","Ubc"
"Wsb1",1.64341879752141e-64,0.941068958261532,0.924,0.618,2.7711327763
806e-60,"0","Wsb1"
"Atf4",6.1714157902912e-64,1.0039130221127,0.902,0.599,1.0406241305589
e-59,"0","Atf4"
"Eif1",3.08560603449179e-62,0.647231213606203,1,0.943,5.20294889536005
e-58,"0","Eif1"
"Atf3",4.15660263768674e-62,1.32123052068005,0.956,0.699,7.00886336766
739e-58,"0","Atf3"
"Eif5",1.50705344221015e-61,0.910749594159987,0.953,0.72,2.54119351425
475e-57,"0","Eif5"
"Jun",6.23262502029701e-59,1.04514537931351,0.995,0.789,1.050945230922
48e-54,"0","Jun"
"Mat2a",1.5439408083842e-58,0.961761270915983,0.951,0.668,2.6033929910
9743e-54,"0","Mat2a"
"Klf6",2.35414972085368e-57,1.05025160590839,0.985,0.716,3.96956725930
348e-53,"0","Klf6"
"Mcl1",1.22239544754339e-56,0.793865689376037,1,0.84,2.06120320364767e
-52,"0","Mcl1"
"Neurl3",1.8355856368553e-54,1.03729944897731,0.897,0.605,3.0951645008
6541e-50,"0","Neurl3"
"Ddit3",6.36179892091768e-53,0.911792754019454,0.608,0.247,1.072726534

04514e-48,"0","Ddit3"
"Tra2b",1.34341343849846e-52,0.920021900321397,0.887,0.619,2.265263739
99611e-48,"0","Tra2b"
"Sqstm1",3.02212115536265e-51,0.923178725442587,0.963,0.697,5.09590069
21725e-47,"0","Sqstm1"
"Srsf5",1.02225139069173e-49,0.747824316721675,0.966,0.73,1.7237202949
8439e-45,"0","Srsf5"
"Egr1",1.2351385151262e-49,0.874641308386815,0.998,0.741,2.08269056420
58e-45,"0","Egr1"
"Kdm6b",1.80729478568031e-49,0.842719083527495,0.968,0.689,3.047460467
61415e-45,"0","Kdm6b"
"Neat1",2.25651515979293e-49,0.921102233385613,0.978,0.706,3.804935862
44284e-45,"0","Neat1"
"Ppp1r10",7.62408871349035e-49,0.837640949677708,0.924,0.619,1.2855738
3886874e-44,"0","Ppp1r10"
"Malat1",1.72831087056952e-47,0.750461102213346,1,0.781,2.914277789954
32e-43,"0","Malat1"
"Dusp8",1.79178895182974e-47,0.760939320658782,0.635,0.301,3.021314530
5753e-43,"0","Dusp8"
"Brd2",2.74167695780689e-47,0.917409384322184,0.853,0.552,4.6230156862
5398e-43,"0","Brd2"
"Irf2bpl",6.55558423430966e-46,0.882981944543408,0.777,0.46,1.10540261
358929e-41,"0","Irf2bpl"
"Zfand5",6.69055012571402e-46,0.770791859237688,0.949,0.728,1.12816056
21979e-41,"0","Zfand5"
"Adgrg1",8.58324294316982e-46,0.655952680695147,0.853,0.487,1.44730642
507729e-41,"0","Adgrg1"
"Rbbp6",1.34841527236699e-45,0.777503691461141,0.797,0.502,2.273697832
26522e-41,"0","Rbbp6"
"Slco2b1",4.07875443839309e-45,0.705487721101746,0.944,0.667,6.8775957
3401844e-41,"0","Slco2b1"
"Pcf11",1.22177348421536e-44,0.708752774617728,0.824,0.5,2.06015444908
394e-40,"0","Pcf11"
"Herpud1",1.88453438971556e-44,0.810518642783834,0.902,0.602,3.1777018
8793838e-40,"0","Herpud1"
"C3ar1",2.10453895930956e-44,0.784233221634625,0.922,0.687,3.548673593
18778e-40,"0","C3ar1"
"Pnrc1",2.94593086210866e-44,0.693390208009891,0.971,0.768,4.967428619
68762e-40,"0","Pnrc1"
"Klf2",5.66775348576552e-43,1.31138410104506,0.887,0.602,9.55696592769
782e-39,"0","Klf2"
"Nav3",6.70681254921042e-43,0.708326326500682,0.855,0.489,1.1309027320
4786e-38,"0","Nav3"
"Tfe3",9.48417259252082e-43,0.767639025724144,0.789,0.491,1.5992211825
5086e-38,"0","Tfe3"
"Stat3",1.10819094650775e-42,0.745324971274588,0.914,0.63,1.8686315740
0137e-38,"0","Stat3"
"Tsc22d3",2.06770069647967e-42,0.982951510276845,0.674,0.349,3.4865569
1440402e-38,"0","Tsc22d3"
"Rela",3.74428918092087e-42,0.679541039021673,0.779,0.478,6.3136204168

6878e-38,"0","Rela"
"Rbm39",6.45742208318513e-42,0.592389875718696,0.975,0.76,1.0888505116
6668e-37,"0","Rbm39"
"Skil",1.95408995427987e-41,0.600756419759848,0.998,0.745,3.2949864809
0672e-37,"0","Skil"
"Sfpq",2.15134653283418e-41,0.661790024232864,0.949,0.721,3.6276005236
65e-37,"0","Sfpq"
"Icam1",3.34890664293105e-41,0.749246605543436,0.971,0.681,5.646926381
31033e-37,"0","Icam1"
"Gm17056",3.76607633047144e-41,0.855218778388635,0.708,0.373,6.3503579
0844094e-37,"0","Gm17056"
"Tnfrsf12a",1.62547482287462e-40,0.578908693254658,0.488,0.182,2.74087
564633118e-36,"0","Tnfrsf12a"
"Tmem119",1.68408919921861e-40,0.690137126030891,0.956,0.654,2.8397112
0772242e-36,"0","Tmem119"
"Dusp1",1.97215756261389e-40,0.790776183432897,0.985,0.744,3.325452082
07954e-36,"0","Dusp1"
"Arid5a",2.14264606969356e-40,0.701152877466683,0.696,0.392,3.61292980
271728e-36,"0","Arid5a"
"Eif1a",3.65649731280091e-40,0.778466630744845,0.721,0.431,6.165585768
84489e-36,"0","Eif1a"
"Rsrp1",4.47995964802141e-40,0.651538912116757,0.958,0.683,7.554107958
49371e-36,"0","Rsrp1"
"Nufip2",5.49800306854033e-40,0.703907765609305,0.76,0.454,9.270732774
1727e-36,"0","Nufip2"
"Sbno2",7.4817973570002e-40,0.747527687119179,0.841,0.552,1.2615806703
3737e-35,"0","Sbno2"
"Srrm2",1.21930047646109e-39,0.607780838687444,0.963,0.768,2.055984463
4087e-35,"0","Srrm2"
"Adap2",2.67393574560077e-39,0.603006559915121,0.873,0.571,4.508790454
23202e-35,"0","Adap2"
"Clk1",5.17619715844106e-39,0.667713247133094,0.924,0.664,8.7281036485
6331e-35,"0","Clk1"
"Siglech",1.56879004987789e-38,0.623035358123229,0.951,0.638,2.6452937
8210409e-34,"0","Siglech"
"P2ry12",2.40574283951027e-38,0.636938755245466,0.98,0.759,4.056563575
98221e-34,"0","P2ry12"
"Son",3.51082471146552e-38,0.649637842851878,0.946,0.74,5.919952628473
17e-34,"0","Son"
"Cx3cr1",4.93495066123422e-38,0.670247518613979,0.985,0.823,8.32131380
497314e-34,"0","Cx3cr1"
"Usp2",5.2506458905261e-38,0.615718809891525,0.718,0.408,8.85363910060
511e-34,"0","Usp2"
"Zbtb7a",5.44002761848965e-38,0.763870423488735,0.777,0.496,9.17297457
029725e-34,"0","Zbtb7a"
"Dnajb1",7.23268085142793e-38,0.944765423902772,0.755,0.457,1.21957464
516778e-33,"0","Dnajb1"
"Rlim",8.77828006348195e-38,0.679568310297846,0.691,0.403,1.4801935843
0433e-33,"0","Rlim"
"Vmp1",3.03255538737859e-37,0.650192488002237,0.904,0.663,5.1134948941

9777e-33,"0","Vmp1"
"Selplg",8.13366186948678e-37,0.602970937485244,0.978,0.823,1.37149806443286e-32,"0","Selplg"
"Zfhx3",1.13631426985816e-36,0.57399710837854,0.941,0.66,1.91605312183483e-32,"0","Zfhx3"
"Cebpa",1.7032131360639e-36,0.859953621940291,0.833,0.552,2.87195799003095e-32,"0","Cebpa"
"Il6ra",3.99864941598312e-36,0.628588848416853,0.887,0.604,6.74252264523073e-32,"0","Il6ra"
"Trib1",8.11441585431775e-36,0.784701472080694,0.828,0.548,1.36825280135506e-31,"0","Trib1"
"Ier2",1.02980204119177e-35,0.715137022477333,0.995,0.807,1.73645220185756e-31,"0","Ier2"
"Ccr5",2.66661543780856e-35,0.661254185517027,0.858,0.561,4.49644695123279e-31,"0","Ccr5"
"Fam214b",3.06330905035785e-35,0.570707263432047,0.515,0.232,5.16535172071342e-31,"0","Fam214b"
"Srgap2",5.5919425336595e-35,0.582492836853656,0.902,0.623,9.42913350025664e-31,"0","Srgap2"
"Fus",2.74020362446592e-34,0.618360290678391,0.912,0.692,4.62053135157444e-30,"0","Fus"
"Taf7",3.01665904160061e-34,0.635074208804523,0.623,0.332,5.08669047594695e-30,"0","Taf7"
"Mafg",3.3055892401923e-34,0.619189505564258,0.848,0.586,5.57388457681225e-30,"0","Mafg"
"H3f3b",7.12196791506742e-34,0.540458207248542,0.998,0.971,1.20090622983867e-29,"0","H3f3b"
"Il10ra",9.25377929837799e-34,0.669923369294811,0.87,0.618,1.5603722652925e-29,"0","Il10ra"
"Epb41l2",1.02307126204658e-33,0.578162747203204,0.973,0.725,1.72510276206294e-29,"0","Epb41l2"
"Tanc2",1.4889617015874e-33,0.545728089801232,0.865,0.55,2.51068722121667e-29,"0","Tanc2"
"Midn",2.51816502824753e-33,0.714993443976397,0.696,0.44,4.24612987063099e-29,"0","Midn"
"Senp2",2.72469223518549e-33,0.616552377931475,0.755,0.488,4.59437604696978e-29,"0","Senp2"
"Csnk1e",5.64192379461277e-33,0.569743962845495,0.909,0.658,9.51341190247605e-29,"0","Csnk1e"
"Col27a1",1.86096108702115e-32,0.581294951358211,0.596,0.299,3.13795258493507e-28,"0","Col27a1"
"Gls",2.57068491067262e-32,0.599683413278465,0.76,0.477,4.33468889637617e-28,"0","Gls"
"Cyth4",1.51470082140522e-31,0.557963013637282,0.956,0.743,2.55408852505349e-27,"0","Cyth4"
"Frmd4a",2.07423810102715e-31,0.517586398407018,0.846,0.55,3.49758028595199e-27,"0","Frmd4a"
"Tmem88",2.50993049346168e-31,0.661775886158731,0.659,0.373,4.23224479807509e-27,"0","Tmem88"
"Gmip",2.93685970923085e-31,0.590595583271642,0.74,0.494,4.95213284170

505e-27,"0","Gmip"
"Slc15a3",4.99162994383012e-31,0.653756567384236,0.9,0.643,8.416886411
28635e-27,"0","Slc15a3"
"Rsrc2",5.75864271226045e-31,0.614953587617567,0.816,0.556,9.710223341
41358e-27,"0","Rsrc2"
"Nktr",1.26231934376775e-30,0.573375353572352,0.855,0.584,2.1285228774
6117e-26,"0","Nktr"
"Rbm25",1.71608218472306e-30,0.558255277172756,0.907,0.674,2.893657779
88002e-26,"0","Rbm25"
"Srsf2",3.71113183512393e-30,0.608582215193876,0.939,0.735,6.257710500
38597e-26,"0","Srsf2"
"Zfp36",4.14879132907577e-30,0.573389659048921,0.993,0.805,6.995691939
08757e-26,"0","Zfp36"
"Mertk",6.2833213082048e-30,0.518069134451725,0.811,0.543,1.0594936389
8949e-25,"0","Mertk"
"Txnrd1",6.9838081186758e-30,0.639871966107213,0.752,0.468,1.177609724
97111e-25,"0","Txnrd1"
"Trim47",7.51070505111608e-30,0.567215632971542,0.623,0.339,1.26645508
571919e-25,"0","Trim47"
"Zc3h12a",8.67025855981818e-30,0.593249792022497,0.738,0.487,1.4619789
9835654e-25,"0","Zc3h12a"
"Gm26532",1.53314413047069e-29,0.615202441111878,0.652,0.374,2.5851876
3279967e-25,"0","Gm26532"
"Sun2",1.55759604366643e-29,0.568098767384177,0.627,0.356,2.6264184488
3034e-25,"0","Sun2"
"Bcl10",4.48756787024609e-29,0.560241521546576,0.877,0.649,7.566936942
80896e-25,"0","Bcl10"
"Nlrp3",6.49164963537232e-29,0.740392855186294,0.892,0.642,1.094621961
51648e-24,"0","Nlrp3"
"Fosb",9.30933916722622e-29,0.888083856521918,0.789,0.507,1.5697407703
7769e-24,"0","Fosb"
"Slc38a2",1.19898839903997e-28,0.540559091373629,0.853,0.599,2.0217342
384612e-24,"0","Slc38a2"
"Rassf1",2.97559241784726e-28,0.610150570481527,0.637,0.371,5.01744393
497405e-24,"0","Rassf1"
"BC005537",3.26975105692372e-28,0.566080847382751,0.824,0.569,5.513454
23218478e-24,"0","BC005537"
"Mef2c",3.29204026735913e-28,0.524748407548447,0.968,0.775,5.551038298
82097e-24,"0","Mef2c"
"Actg1",3.75317945006862e-28,0.38428926028541,1,0.989,6.32861118870571
e-24,"0","Actg1"
"Arhgap17",4.02896578245494e-28,0.536839171536121,0.745,0.491,6.793642
10237552e-24,"0","Arhgap17"
"Gm46224",4.5256617604492e-28,0.729598151060802,0.404,0.173,7.63117086
046944e-24,"0","Gm46224"
"Mef2a",6.43303598352933e-28,0.486838197953688,0.951,0.727,1.084738527
54271e-23,"0","Mef2a"
"Nfkb1",7.60410708562794e-28,0.518616164963938,0.909,0.675,1.282204536
77858e-23,"0","Nfkb1"
"Cggbp1",7.96831478241564e-28,0.511347363775285,0.733,0.474,1.34361723

861092e-23,"0","Cggbp1"
"Vps37b",9.00805775344456e-28,0.552603413446998,0.642,0.381,1.51893869
838582e-23,"0","Vps37b"
"Gpr34",1.28946218611061e-27,0.547430517703901,0.973,0.779,2.174291138
21972e-23,"0","Gpr34"
"Rasgrp3",2.0086491215254e-27,0.49134245993134,0.725,0.438,3.386984148
71612e-23,"0","Rasgrp3"
"Csf1r",4.95479125372501e-27,0.371788544513096,1,0.931,8.3547690120311
1e-23,"0","Csf1r"
"Vsir",8.05221605949611e-27,0.417831713724658,0.971,0.783,1.3577646719
5223e-22,"0","Vsir"
"Ddx6",1.08062248753472e-26,0.495627275994908,0.865,0.623,1.8221456384
8104e-22,"0","Ddx6"
"Plekho2",1.17525751006065e-26,0.561189302742074,0.828,0.588,1.9817192
1346426e-22,"0","Plekho2"
"Rtn4rl1",2.10499843222003e-26,0.395166001504817,0.613,0.334,3.5494483
5640942e-22,"0","Rtn4rl1"
"Chd4",2.83621395864332e-26,0.584319684526601,0.885,0.671,4.7824239770
6436e-22,"0","Chd4"
"Tsc22d2",3.35596894296046e-26,0.469075399232152,0.846,0.554,5.6588348
3161992e-22,"0","Tsc22d2"
"Rel",3.50579411964021e-26,0.506854816705077,0.931,0.659,5.91147004453
732e-22,"0","Rel"
"Tnpo3",4.92001172323141e-26,0.513966308178005,0.669,0.416,8.296123767
7128e-22,"0","Tnpo3"
"Arhgap5",6.05216451904284e-26,0.47032099443282,0.914,0.642,1.02051598
1201e-21,"0","Arhgap5"
"Ddx3x",8.03371308982333e-26,0.564515665829068,0.855,0.64,1.3546447012
0601e-21,"0","Ddx3x"
"Elf1",9.30758156300832e-26,0.489999002394037,0.757,0.498,1.5694444031
5446e-21,"0","Elf1"
"Dennd4a",9.31889676700464e-26,0.503782966051489,0.949,0.692,1.5713523
7285232e-21,"0","Dennd4a"
"Etf1",1.1522316451189e-25,0.43130338912418,0.843,0.585,1.942892999999
49e-21,"0","Etf1"
"Lair1",1.56042846994872e-25,0.464076709896871,0.975,0.771,2.631194486
02753e-21,"0","Lair1"
"Pfkfb3",1.85404611430543e-25,0.568688812698732,0.637,0.384,3.12629255
794182e-21,"0","Pfkfb3"
"Ubash3b",2.3151063219931e-25,0.469297814727124,0.799,0.537,3.90373228
014476e-21,"0","Ubash3b"
"Slc16a6",3.55993426102125e-25,0.491390676323368,0.787,0.519,6.0027611
5093403e-21,"0","Slc16a6"
"Ndel1",4.78405734650068e-25,0.499165648487636,0.809,0.558,8.066877497
66944e-21,"0","Ndel1"
"Ddx50",7.20611130728984e-25,0.52988072975438,0.713,0.465,1.2150944886
3521e-20,"0","Ddx50"
"Mknk2",7.94552870175912e-25,0.490515089131127,0.721,0.475,1.339775049
69062e-20,"0","Mknk2"
"Mafk",2.01354736829924e-24,0.421318117635723,0.414,0.189,3.3952435724

2618e-20,"0","Mafk"
"Ubn1",3.1158470276013e-24,0.437204943453414,0.809,0.548,5.25394125794131e-20,"0","Ubn1"
"Ssh2",3.12762705196056e-24,0.465642213985849,0.89,0.642,5.27380473501589e-20,"0","Ssh2"
"Csf3r",3.16574882635352e-24,0.411752312541602,0.762,0.507,5.3380856709973e-20,"0","Csf3r"
"Map2k3",3.34855505318261e-24,0.506135765869205,0.755,0.524,5.64633353067651e-20,"0","Map2k3"
"Bmp2k",1.43449736357275e-23,0.438283696341786,0.89,0.656,2.41884945445638e-19,"0","Bmp2k"
"Plxdc2",3.14803283128056e-23,0.435210511018567,0.958,0.727,5.30821296010528e-19,"0","Plxdc2"
"Spag9",3.22197602282792e-23,0.527710434507552,0.855,0.618,5.43289596969244e-19,"0","Spag9"
"Itga5",5.26368015414634e-23,0.463585170434172,0.409,0.194,8.87561747592156e-19,"0","Itga5"
"Fbrsl1",5.45692308023418e-23,0.494666261608868,0.657,0.414,9.20146369789088e-19,"0","Fbrsl1"
"Hps4",1.13941547082146e-22,0.419161157423157,0.6,0.357,1.92128236689914e-18,"0","Hps4"
"Ppp1r11",1.28957337002256e-22,0.542970533733524,0.676,0.463,2.17447861653204e-18,"0","Ppp1r11"
"Rnf19b",1.42427683570901e-22,0.619298152809147,0.728,0.51,2.40161560037254e-18,"0","Rnf19b"
"Qk",1.5660470472377e-22,0.429263520230188,0.973,0.803,2.64066853105221e-18,"0","Qk"
"Slc29a3",1.87636214501227e-22,0.444295712311033,0.846,0.595,3.16392184891969e-18,"0","Slc29a3"
"Tmem41b",1.95551118706675e-22,0.430529279525476,0.409,0.2,3.29738296363196e-18,"0","Tmem41b"
"Ywhaz",2.04112428912869e-22,0.448100841904828,0.87,0.636,3.44174377632879e-18,"0","Ywhaz"
"Ube2f",2.31716270936229e-22,0.464976411435514,0.735,0.5,3.90719976052669e-18,"0","Ube2f"
"Aff4",2.43212159871241e-22,0.466546153415462,0.674,0.455,4.10104343974887e-18,"0","Aff4"
"Top1",3.9621720190646e-22,0.472722967610196,0.855,0.645,6.68101445854673e-18,"0","Top1"
"Ascc3",4.17333944732282e-22,0.334731924042788,0.561,0.322,7.03708497607574e-18,"0","Ascc3"
"Hexim1",4.68545460599473e-22,0.475442194047167,0.559,0.338,7.90061355662831e-18,"0","Hexim1"
"Chd2",4.92130841050453e-22,0.514825541851304,0.635,0.42,8.29831024179274e-18,"0","Chd2"
"Ints6",4.94622335264003e-22,0.624536964477176,0.586,0.365,8.34032181722161e-18,"0","Ints6"
"Adap2os",5.22964547989073e-22,0.394310897454033,0.517,0.285,8.81822820819175e-18,"0","Adap2os"
"Fgd2",5.91445895381509e-22,0.426922400204995,0.816,0.592,9.9729606879

2301e-18,"0","Fgd2"
"Slbp",5.98205976929735e-22,0.482574168197139,0.63,0.401,1.00869491829892e-17,"0","Slbp"
"Zcchc6",6.78535346623796e-22,0.473375867482779,0.838,0.62,1.14414630147704e-17,"0","Zcchc6"
"Lpin2",9.93308062674688e-22,0.492941365345295,0.654,0.42,1.67491605528206e-17,"0","Lpin2"
"Il4ra",1.00316751488955e-21,0.46477601037841,0.723,0.488,1.69154106360676e-17,"0","Il4ra"
"Srxn1",1.04901421329198e-21,0.697692235665932,0.522,0.285,1.76884776645294e-17,"0","Srxn1"
"Serinc3",1.10428041303e-21,0.355865389101651,0.998,0.916,1.86203763245118e-17,"0","Serinc3"
"Mknk1",1.11319707182857e-21,0.433851029036077,0.623,0.41,1.87707290251733e-17,"0","Mknk1"
"Map3k8",1.14887723929016e-21,0.512937643220923,0.701,0.463,1.93723680089107e-17,"0","Map3k8"
"Erf",1.53639787827762e-21,0.468350277612402,0.527,0.306,2.59067410235173e-17,"0","Erf"
"Tpm3",2.04407384069261e-21,0.364246830741409,0.983,0.889,3.44671731017589e-17,"0","Tpm3"
"Dnaja1",2.82553887511334e-21,0.50921440798362,0.877,0.694,4.76442365121612e-17,"0","Dnaja1"
"Tob2",2.91049614394614e-21,0.53779717253586,0.738,0.516,4.90767859792198e-17,"0","Tob2"
"Whrn",2.96017307535738e-21,0.349200876189677,0.397,0.185,4.99144383966761e-17,"0","Whrn"
"Cd37",3.04121018653594e-21,0.402414592734926,0.934,0.727,5.1280886165369e-17,"0","Cd37"
"Cebpg",3.46636253673162e-21,0.433771819440963,0.728,0.506,5.84498050943685e-17,"0","Cebpg"
"Tox4",4.17306399890863e-21,0.445180291177838,0.64,0.416,7.03662051495973e-17,"0","Tox4"
"Nfic",5.67294556683859e-21,0.446924766837838,0.703,0.476,9.56572081480322e-17,"0","Nfic"
"Arid3b",5.93545234835873e-21,0.363387488744502,0.348,0.153,1.00083597498025e-16,"0","Arid3b"
"St3gal6",6.53734208498585e-21,0.439861956267757,0.806,0.573,1.10232662237031e-16,"0","St3gal6"
"Tubb2a",7.14045862934642e-21,0.39716933820515,0.743,0.51,1.20402413408039e-16,"0","Tubb2a"
"Scamp2",8.97283873670506e-21,0.404319299016203,0.951,0.772,1.51300006778321e-16,"0","Scamp2"
"Hspa1a",1.12160797443072e-20,0.717817539967611,0.529,0.301,1.89125536648508e-16,"0","Hspa1a"
"Ppp1r15a",1.15465449491293e-20,0.343028678904498,0.971,0.695,1.94697840932218e-16,"0","Ppp1r15a"
"Pla2g15",1.29295824694327e-20,0.3958994644509,0.792,0.549,2.18018619599574e-16,"0","Pla2g15"
"Mafb",1.83508349586227e-20,0.666988362945796,0.924,0.761,3.0943177907

2297e-16,"0","Mafb"
"Hnrnph1",1.90331997558183e-20,0.402975216274155,0.765,0.537,3.2093781
4282607e-16,"0","Hnrnph1"
"Ptpro",2.25480620020461e-20,0.375466048027309,0.542,0.312,3.802054214
78501e-16,"0","Ptpro"
"Cd33",2.26236481491787e-20,0.392373117021736,0.855,0.616,3.8147995509
1452e-16,"0","Cd33"
"Eif4a1",2.35011766231211e-20,0.333524547494562,0.99,0.923,3.962768402
19068e-16,"0","Eif4a1"
"Eid3",2.38847152578162e-20,0.550536728578022,0.417,0.217,4.0274406867
7297e-16,"0","Eid3"
"March7",2.76449255569686e-20,0.406870085289256,0.711,0.491,4.66148734
741604e-16,"0","March7"
"Lrif1",2.98798956233168e-20,0.403569627664217,0.395,0.192,5.038348000
00367e-16,"0","Lrif1"
"Etv3",2.9925936136636e-20,0.514717318937459,0.534,0.319,5.04611135135
956e-16,"0","Etv3"
"Samd4b",3.02459932247964e-20,0.376078674369414,0.542,0.316,5.10007937
756517e-16,"0","Samd4b"
"P2ry13",3.19104897727816e-20,0.396822866960455,0.836,0.595,5.38074678
548643e-16,"0","P2ry13"
"Ctc1",3.43744307745331e-20,0.382098343754465,0.647,0.427,5.7962165172
0177e-16,"0","Ctc1"
"Lpcat2",3.67976760874292e-20,0.35594817049862,0.978,0.831,6.204824141
86232e-16,"0","Lpcat2"
"Errfi1",3.84962697976091e-20,0.612604496563278,0.431,0.223,6.49124101
327285e-16,"0","Errfi1"
"2810403A07Rik",4.18306987875022e-20,0.392762698752788,0.453,0.247,7.0
5349242954862e-16,"0","2810403A07Rik"
"F11r",4.66083332509686e-20,0.394066613156555,0.75,0.503,7.85909715277
833e-16,"0","F11r"
"Purb",5.35951605121559e-20,0.39084389857895,0.919,0.72,9.037215965559
73e-16,"0","Purb"
"Hexb",6.52323984823523e-20,0.345285521202548,1,0.945,1.09994870320942
e-15,"0","Hexb"
"Sirpa",7.28746863199866e-20,0.382857084277067,0.973,0.83,1.2288129607
2761e-15,"0","Sirpa"
"Tmcc3",7.46422497259208e-20,0.358798572617133,0.816,0.563,1.258617614
87848e-15,"0","Tmcc3"
"Blnk",8.29135625203261e-20,0.427876198659213,0.701,0.472,1.3980884912
1774e-15,"0","Blnk"
"Sik2",8.32059410598506e-20,0.429824446339333,0.439,0.23,1.40301857815
12e-15,"0","Sik2"
"Pou2f2",8.80846854236636e-20,0.400386823306935,0.949,0.722,1.48528396
561382e-15,"0","Pou2f2"
"Siah2",1.42450663499348e-19,0.375395834663604,0.392,0.193,2.402003087
926e-15,"0","Siah2"
"Ski",1.91498941304739e-19,0.38691238715783,0.826,0.598,3.229055148280
51e-15,"0","Ski"
"Luc7l2",1.95742220058882e-19,0.398247469330124,0.897,0.686,3.30060531

463287e-15,"0","Luc7l2"
"Fmnl3",1.97115344140643e-19,0.372349870453605,0.564,0.351,3.323758932
89951e-15,"0","Fmnl3"
"AC149090.1",3.73040583393798e-19,0.381151899693282,0.647,0.422,6.2902
1031718623e-15,"0","AC149090.1"
"Csnk1a1",4.07888240304788e-19,0.345415503434413,0.887,0.697,6.8778115
0801934e-15,"0","Csnk1a1"
"Ccnt1",4.097435443874e-19,0.40873390364032,0.647,0.418,6.909095645460
33e-15,"0","Ccnt1"
"Foxn3",4.26116327924835e-19,0.396704918832253,0.833,0.599,7.185173521
46856e-15,"0","Foxn3"
"Tgfbr2",4.60074370564985e-19,0.400425341581151,0.848,0.617,7.75777403
646678e-15,"0","Tgfbr2"
"Slc2a5",6.77841563463377e-19,0.325066121515873,0.517,0.285,1.14297644
431195e-14,"0","Slc2a5"
"Hnrnpa2b1",7.03208627069013e-19,0.409708987197826,0.975,0.813,1.18575
038696377e-14,"0","Hnrnpa2b1"
"Marcksl1",8.39996522920084e-19,0.50017597693679,0.904,0.69,1.41640213
694784e-14,"0","Marcksl1"
"Safb2",8.47233885214363e-19,0.349815247134627,0.657,0.43,1.4286057772
4846e-14,"0","Safb2"
"Kcnk13",1.07310433506331e-18,0.381078219943151,0.586,0.376,1.80946852
978375e-14,"0","Kcnk13"
"Numb",1.14236911042126e-18,0.324146603180362,0.561,0.332,1.9262627939
9233e-14,"0","Numb"
"Kcnq1ot1",1.18160630542433e-18,0.761650255170668,0.574,0.37,1.9924245
522065e-14,"0","Kcnq1ot1"
"Plxdc1",1.29695780655526e-18,0.322581850995913,0.441,0.238,2.18693025
341348e-14,"0","Plxdc1"
"Wdr45b",1.52137377820637e-18,0.358580077140682,0.569,0.361,2.56534046
481159e-14,"0","Wdr45b"
"Tpst2",1.59118143661076e-18,0.353077421306884,0.877,0.656,2.683050138
41306e-14,"0","Tpst2"
"Kmt2e",1.70313200858243e-18,0.401446377114336,0.755,0.542,2.871821192
8717e-14,"0","Kmt2e"
"Jmjd6",1.79761686960223e-18,0.395897176653227,0.49,0.284,3.0311415655
2327e-14,"0","Jmjd6"
"Prkx",1.98810764500556e-18,0.436158142152437,0.475,0.271,3.3523471110
0838e-14,"0","Prkx"
"Casp4",1.9902960615093e-18,0.512633031120473,0.757,0.548,3.3560372189
1698e-14,"0","Casp4"
"Zbtb11",1.99821123554578e-18,0.43688829397446,0.414,0.222,3.369383785
37729e-14,"0","Zbtb11"
"Fam102b",2.23316288506473e-18,0.393533116644202,0.718,0.494,3.7655592
5679615e-14,"0","Fam102b"
"Glul",2.24950160558725e-18,0.449470378181369,0.961,0.807,3.7931096073
4123e-14,"0","Glul"
"Arhgap45",2.68082725039641e-18,0.323737421480463,0.789,0.573,4.520410
90961843e-14,"0","Arhgap45"
"Crk",2.71811844134215e-18,0.307444375793387,0.691,0.476,4.58329131579

113e-14,"0","Crk"
"Kdm2a",3.36410308626461e-18,0.32405439850733,0.603,0.385,5.6725506240
5939e-14,"0","Kdm2a"
"Acox3",4.57106539344262e-18,0.319029046091748,0.583,0.373,7.707730466
42294e-14,"0","Acox3"
"Sipa1",5.01088257034788e-18,0.355392848467535,0.772,0.565,8.449350190
1206e-14,"0","Sipa1"
"Fubp1",5.50146505172442e-18,0.387645960744381,0.75,0.546,9.2765703702
1772e-14,"0","Fubp1"
"Plekhm2",5.72857038165184e-18,0.37508565835709,0.441,0.244,9.65951537
754133e-14,"0","Plekhm2"
"Arf6",5.83847363485838e-18,0.387209375429848,0.627,0.424,9.8448342430
982e-14,"0","Arf6"
"Hnrnpab",6.46761803895783e-18,0.430863490038388,0.794,0.596,1.0905697
5372907e-13,"0","Hnrnpab"
"Daglb",7.10964272132005e-18,0.356172181282724,0.892,0.685,1.198827955
66899e-13,"0","Daglb"
"Clk4",7.2609763683744e-18,0.308802294321676,0.525,0.308,1.22434583523
529e-13,"0","Clk4"
"Golm1",7.69024480916367e-18,0.342193284111458,0.873,0.619,1.296729079
72118e-13,"0","Golm1"
"Prpf38b",7.80748693113576e-18,0.360558372451598,0.689,0.476,1.3164984
4632811e-13,"0","Prpf38b"
"Hspa5",8.76330640788983e-18,0.436825357426809,0.988,0.867,1.477668726
49838e-13,"0","Hspa5"
"Phf23",9.0796807589619e-18,0.345946974233406,0.424,0.228,1.5310157695
7615e-13,"0","Phf23"
"Tgif2",9.2290333121026e-18,0.412154660290637,0.38,0.193,1.55619959708
674e-13,"0","Tgif2"
"Crlf3",1.04153574158656e-17,0.334259997989666,0.662,0.452,1.756237567
46326e-13,"0","Crlf3"
"Hpgds",1.04900591463631e-17,0.400302947976828,0.944,0.729,1.768833773
25975e-13,"0","Hpgds"
"Plk3",1.07654410963375e-17,0.445868222152528,0.767,0.532,1.8152686776
6442e-13,"0","Plk3"
"Pde3b",1.18414345047517e-17,0.384168729314307,0.848,0.621,1.996702686
19123e-13,"0","Pde3b"
"Itgb5",1.25324133857482e-17,0.343037523820816,0.99,0.836,2.1132155451
0486e-13,"0","Itgb5"
"Rapgef5",1.41555796787419e-17,0.394775115316879,0.561,0.356,2.3869138
4542945e-13,"0","Rapgef5"
"Tgfbr1",1.47345863212226e-17,0.367016695370762,0.919,0.706,2.48454594
548455e-13,"0","Tgfbr1"
"Pwwp2a",1.52011234954806e-17,0.360767964353807,0.551,0.348,2.56321344
380794e-13,"0","Pwwp2a"
"Frmd4b",1.61517378415381e-17,0.301841730295984,0.706,0.472,2.72350603
484015e-13,"0","Frmd4b"
"Rasal3",2.47846255510745e-17,0.320369924627165,0.669,0.461,4.17918356
042218e-13,"0","Rasal3"
"Tbpl1",2.48447716129964e-17,0.451261945791837,0.635,0.427,4.189325389

38345e-13,"0","Tbpl1"
"Ccnl2",2.94068263130311e-17,0.356876241159095,0.667,0.466,4.958579052
90331e-13,"0","Ccnl2"
"Ggnbp2",3.14118117308347e-17,0.337978696376215,0.674,0.452,5.29665969
405334e-13,"0","Ggnbp2"
"Cables1",3.53796139327323e-17,0.339619984860556,0.458,0.264,5.9657105
0133731e-13,"0","Cables1"
"Tsc22d4",3.74990911664992e-17,0.356726186601518,0.775,0.584,6.3230967
524951e-13,"0","Tsc22d4"
"Bcl6",3.99209267256059e-17,0.407756466860345,0.559,0.363,6.7314666644
7167e-13,"0","Bcl6"
"Mylip",4.09006589662865e-17,0.457602672742316,0.816,0.601,6.896669114
89523e-13,"0","Mylip"
"Sowahc",4.2138615040903e-17,0.393357818297177,0.713,0.482,7.105413268
19706e-13,"0","Sowahc"
"Sall1",4.21830070905405e-17,0.340816091296458,0.669,0.437,7.112898655
60694e-13,"0","Sall1"
"Hnrnpu",6.6489682155904e-17,0.385776030760216,0.951,0.793,1.121149020
51285e-12,"0","Hnrnpu"
"Id2",7.34601990420919e-17,0.888685166935967,0.672,0.494,1.23868587624
775e-12,"0","Id2"
"Elmo1",8.02612329864234e-17,0.365223459373372,0.804,0.572,1.353364910
61707e-12,"0","Elmo1"
"BC037034",8.51836446125542e-17,0.386421476959155,0.485,0.295,1.436366
61545689e-12,"0","BC037034"
"Ube2d3",9.68346775695308e-17,0.316937113582135,0.975,0.861,1.63282633
317743e-12,"0","Ube2d3"
"Tmx4",9.80810608762687e-17,0.52611109769383,0.517,0.322,1.65384284849
564e-12,"0","Tmx4"
"Ldb1",1.07375171901737e-16,0.321006466204382,0.522,0.327,1.8105601486
0709e-12,"0","Ldb1"
"Dnajb9",1.19926473423468e-16,0.393932364380308,0.591,0.37,2.022200194
86652e-12,"0","Dnajb9"
"Pag1",1.23342799524125e-16,0.333711996969799,0.694,0.469,2.0798062855
758e-12,"0","Pag1"
"Eif4g2",1.2853333570605e-16,0.346944786413802,0.936,0.782,2.167329106
67542e-12,"0","Eif4g2"
"Nabp1",1.34130749774774e-16,0.370324584737364,0.385,0.2,2.26171270270
224e-12,"0","Nabp1"
"Pdgfb",1.40361009701537e-16,0.534499926678354,0.699,0.496,2.366767345
58731e-12,"0","Pdgfb"
"Gtf2h2",1.81687392084299e-16,0.276736804206761,0.684,0.465,3.06361280
532544e-12,"0","Gtf2h2"
"Hnrnpdl",1.83135400779374e-16,0.378743145649187,0.716,0.524,3.0880291
2794181e-12,"0","Hnrnpdl"
"Zeb2",1.95342784355874e-16,0.350663352095476,0.966,0.746,3.2938700298
0875e-12,"0","Zeb2"
"Ube2h",2.02358947278722e-16,0.349909341026803,0.618,0.419,3.412176569
0138e-12,"0","Ube2h"
"Wdr26",2.07884832256545e-16,0.310464481845566,0.86,0.665,3.5053540415

0987e-12,"0","Wdr26"
"Nectin2",2.27481783221537e-16,0.344594806523525,0.483,0.278,3.8357978
2868155e-12,"0","Nectin2"
"Ranbp2",3.03175234754226e-16,0.394165644476531,0.689,0.467,5.11214080
842575e-12,"0","Ranbp2"
"Btg1",3.49398232695375e-16,0.327581183836017,0.98,0.806,5.89155299970
941e-12,"0","Btg1"
"Ythdc1",3.7269328985263e-16,0.389899934238057,0.605,0.419,6.284354253
49505e-12,"0","Ythdc1"
"Fbrs",3.85997577200388e-16,0.385437166740611,0.51,0.322,6.50869114675
295e-12,"0","Fbrs"
"Slc12a9",3.90556958945405e-16,0.337597913718268,0.637,0.442,6.5855714
4173741e-12,"0","Slc12a9"
"Ptbp3",4.47156923699948e-16,0.336650041111666,0.855,0.661,7.539960047
42853e-12,"0","Ptbp3"
"Tbc1d9",4.60810761162335e-16,0.349149804165979,0.564,0.365,7.77019105
471929e-12,"0","Tbc1d9"
"1810013L24Rik",4.81821837695418e-16,0.360786941656379,0.414,0.239,8.1
2447982722013e-12,"0","1810013L24Rik"
"Dst",5.32441290919582e-16,0.3461614625347,0.767,0.548,8.9780250474859
9e-12,"0","Dst"
"Kctd12",5.71040724923697e-16,0.481487994228223,0.98,0.804,9.628888703
66338e-12,"0","Kctd12"
"Ptpn1",5.92237711187996e-16,0.322680953214513,0.941,0.761,9.986312286
05199e-12,"0","Ptpn1"
"Dnase2a",5.9792698085688e-16,0.406001580994052,0.775,0.58,1.008224475
12087e-11,"0","Dnase2a"
"Nfkbiz",6.46262826167655e-16,0.402573445306482,0.975,0.733,1.08972837
74839e-11,"0","Nfkbiz"
"Pmepa1",7.14487829444515e-16,0.351248404569378,0.936,0.719,1.20476937
800934e-11,"0","Pmepa1"
"Usp16",7.50130214251423e-16,0.322095059586458,0.502,0.309,1.264869567
27075e-11,"0","Usp16"
"Klf4",8.31507468094798e-16,0.600536600096731,0.574,0.367,1.4020878927
0145e-11,"0","Klf4"
"Zranb1",9.05843407032351e-16,0.396339239650748,0.483,0.299,1.52743315
293795e-11,"0","Zranb1"
"Unc93b1",9.23423334590102e-16,0.27247181055012,0.98,0.901,1.557076426
78583e-11,"0","Unc93b1"
"Atp6v0a2",9.31001123219173e-16,0.34162168272624,0.603,0.4,1.569854093
97217e-11,"0","Atp6v0a2"
"Olfml3",9.6973114321958e-16,0.371531391383186,0.983,0.769,1.635160653
69686e-11,"0","Olfml3"
"Bptf",1.05547820057493e-15,0.306288833057792,0.725,0.531,1.7797473418
0945e-11,"0","Bptf"
"Zfp36l1",1.08372114170162e-15,0.489514982455547,0.89,0.684,1.82737058
913728e-11,"0","Zfp36l1"
"Lag3",1.11342941473707e-15,0.303338465192932,0.909,0.65,1.87746467912
965e-11,"0","Lag3"
"Irf5",1.14583071433839e-15,0.348483535746929,0.865,0.675,1.9320997505

1739e-11,"0","Irf5"
"Ptbp1",1.2387710689906e-15,0.388665676515415,0.74,0.527,2.08881577653195e-11,"0","Ptbp1"
"Ticam1",1.28512177435752e-15,0.297366523140389,0.294,0.139,2.16697233592165e-11,"0","Ticam1"
"Fam110a",1.29799462131384e-15,0.46019209076366,0.463,0.271,2.18867853045939e-11,"0","Fam110a"
"Orai1",1.53814539653661e-15,0.342551240037709,0.816,0.612,2.59362076764003e-11,"0","Orai1"
"Peli1",1.5605497161317e-15,0.37426240914458,0.733,0.54,2.63139893134127e-11,"0","Peli1"
"Gt(ROSA)26Sor",1.66382926319331e-15,0.316654578884624,0.424,0.241,2.80554890359655e-11,"0","Gt(ROSA)26Sor"
"Ep300",1.87918700234425e-15,0.365775118429267,0.61,0.42,3.16868512335287e-11,"0","Ep300"
"Pafah1b1",1.88958451318522e-15,0.346838243496874,0.838,0.667,3.18621740613292e-11,"0","Pafah1b1"
"Ppfia4",2.06393805109625e-15,0.32283271351694,0.902,0.673,3.4802123417585e-11,"0","Ppfia4"
"Zrsr2",2.12770357735299e-15,0.370642940975538,0.51,0.33,3.58773377213261e-11,"0","Zrsr2"
"Arid4a",2.17707529563897e-15,0.347139189848287,0.645,0.448,3.67098436350643e-11,"0","Arid4a"
"Sde2",2.75391992491666e-15,0.365752701952568,0.547,0.348,4.64365977739447e-11,"0","Sde2"
"Tuba1c",2.96521350316282e-15,0.262919171782356,0.75,0.539,4.99994300903314e-11,"0","Tuba1c"
"Arhgef2",3.05926659629138e-15,0.306455993958431,0.593,0.407,5.15853533466653e-11,"0","Arhgef2"
"Wnk1",3.30174801063017e-15,0.413292544671987,0.953,0.768,5.56740749552459e-11,"0","Wnk1"
"Akirin2",3.68906786398777e-15,0.310320529200086,0.701,0.501,6.22050623225618e-11,"0","Akirin2"
"Sall3",3.93924232335672e-15,0.314609240474683,0.505,0.303,6.6423504056441e-11,"0","Sall3"
"9930111J21Rik2",4.06659769577117e-15,0.330611496962615,0.593,0.403,6.85709703460935e-11,"0","9930111J21Rik2"
"Evi2a",4.27187437867218e-15,0.354613334670082,0.951,0.8,7.20323457731703e-11,"0","Evi2a"
"Iffo2",4.46017008997282e-15,0.296280911794462,0.277,0.125,7.52073880571217e-11,"0","Iffo2"
"Phf14",5.01861192165542e-15,0.313671725932981,0.645,0.442,8.46238342229536e-11,"0","Phf14"
"Ttll3",5.1890234140553e-15,0.303240981573268,0.426,0.241,8.74973128078004e-11,"0","Ttll3"
"Socs3",5.66385310355799e-15,0.422867191929802,0.939,0.73,9.55038910321948e-11,"0","Socs3"
"Acp2",5.94455668297176e-15,0.283700890969457,0.681,0.48,1.0023711478827e-10,"0","Acp2"
"Arglu1",6.06173906873617e-15,0.32953760647291,0.706,0.527,1.022130441

77029e-10,"0","Arglu1"
"Tbc1d16",6.6076952293266e-15,0.291187299535579,0.596,0.393,1.11418956956905e-10,"0","Tbc1d16"
"Csrnp1",6.9786367232056e-15,0.348075113756046,0.86,0.642,1.17673772426693e-10,"0","Csrnp1"
"St3gal5",8.73821535273213e-15,0.290209333720967,0.596,0.396,1.47343787277769e-10,"0","St3gal5"
"Il1a",8.89801721663488e-15,0.407109188702438,0.958,0.709,1.50038366306897e-10,"0","Il1a"
"Sf1",9.14345762378241e-15,0.333938073414221,0.625,0.437,1.54176982452219e-10,"0","Sf1"
"Ptgs1",9.25736047614358e-15,0.340425488029477,0.941,0.744,1.56097612348733e-10,"0","Ptgs1"
"Rybp",1.1163514067263e-14,0.25256790054503,0.341,0.174,1.88239174202189e-10,"0","Rybp"
"Eng",1.22360710039682e-14,0.323114991117202,0.429,0.256,2.06324629268911e-10,"0","Eng"
"Paqr7",1.43356770071112e-14,0.299872544995104,0.456,0.273,2.41728185693909e-10,"0","Paqr7"
"Tcirg1",1.44846440669237e-14,0.266207924498012,0.77,0.551,2.44240068256468e-10,"0","Tcirg1"
"Brd1",1.45478382472415e-14,0.369254882527823,0.537,0.366,2.45305648524986e-10,"0","Brd1"
"Csad",1.49770983406951e-14,0.290914953485728,0.377,0.206,2.52543832220801e-10,"0","Csad"
"Xbp1",1.53391001259687e-14,0.348998151731087,0.723,0.528,2.58647906324084e-10,"0","Xbp1"
"Rnf215",1.55909496796546e-14,0.27711601193396,0.475,0.3,2.62894593498336e-10,"0","Rnf215"
"Marcks",1.58007230925068e-14,0.320749992364276,0.993,0.903,2.6643179278585e-10,"0","Marcks"
"Fbxo33",1.67764126657583e-14,0.368446710510868,0.424,0.254,2.82883870370017e-10,"0","Fbxo33"
"Dip2b",1.79931465285434e-14,0.308404922368184,0.608,0.414,3.03400436764298e-10,"0","Dip2b"
"Stk40",2.60808557313448e-14,0.291742953144374,0.468,0.289,4.39775389341936e-10,"0","Stk40"
"Irf2bp2",2.64004168644147e-14,0.353362778873058,0.811,0.605,4.4516382916776e-10,"0","Irf2bp2"
"Kdm6bos",2.67518109393889e-14,0.263705886800114,0.25,0.11,4.51089036059976e-10,"0","Kdm6bos"
"Creb5",2.71063602437878e-14,0.594349081872175,0.368,0.203,4.5706744643075e-10,"0","Creb5"
"Itpkc",2.71869128010433e-14,0.330491401787794,0.333,0.172,4.58425723651193e-10,"0","Itpkc"
"Susd6",2.7404358465309e-14,0.366310262314902,0.539,0.357,4.62092292442041e-10,"0","Susd6"
"Mtdh",2.7967041915782e-14,0.299521997847668,0.941,0.789,4.71580260783916e-10,"0","Mtdh"
"Setd5",2.83698002748176e-14,0.33822950285881,0.574,0.389,4.7837157223

3974e-10,"0","Setd5"
"Mob3c",2.96046924327589e-14,0.326532484604064,0.627,0.435,4.991943238
01181e-10,"0","Mob3c"
"Stard3",3.03425872093702e-14,0.313400957449698,0.686,0.508,5.11636705
5244e-10,"0","Stard3"
"Ripk1",3.12465522230146e-14,0.287894792364702,0.529,0.346,5.268793635
84473e-10,"0","Ripk1"
"Nckap5l",3.1313984381115e-14,0.259482712402871,0.304,0.147,5.28016404
634362e-10,"0","Nckap5l"
"Snrnp70",3.1756901246879e-14,0.322302696195607,0.821,0.645,5.35484868
824873e-10,"0","Snrnp70"
"Inpp5d",3.24763925700569e-14,0.348632370817802,0.826,0.634,5.47616931
516299e-10,"0","Inpp5d"
"P2ry6",3.263433429464e-14,0.407552260727106,0.777,0.611,5.50280144876
22e-10,"0","P2ry6"
"Gtf2b",3.54600287847462e-14,0.381817606230607,0.586,0.411,5.979270053
6839e-10,"0","Gtf2b"
"Rps6ka1",3.5596949151918e-14,0.284961698397616,0.703,0.486,6.00235756
599641e-10,"0","Rps6ka1"
"Retreg1",3.61156659805534e-14,0.2948896418432,0.848,0.642,6.089823597
64092e-10,"0","Retreg1"
"Slc7a8",3.63230617875248e-14,0.317152568421507,0.706,0.502,6.12479467
861244e-10,"0","Slc7a8"
"Sft2d1",4.12557996726266e-14,0.294005592552394,0.912,0.733,6.95655294
07983e-10,"0","Sft2d1"
"Rtn4",4.55878622314526e-14,0.308354575990931,0.951,0.815,7.6870253294
6754e-10,"0","Rtn4"
"Cttnbp2nl",4.74753022631919e-14,0.292430876104605,0.728,0.541,8.00528
546761941e-10,"0","Cttnbp2nl"
"Zfp36l2",4.84609893343737e-14,0.410251977857925,0.846,0.657,8.1714920
215621e-10,"0","Zfp36l2"
"Fli1",4.95932081105356e-14,0.260147392647035,0.662,0.469,8.3624067515
9851e-10,"0","Fli1"
"Serpine2",5.34700331408985e-14,0.313683485850756,0.941,0.69,9.0161169
882183e-10,"0","Serpine2"
"Myd88",6.25871530580066e-14,0.349369023736759,0.549,0.362,1.055344574
86411e-09,"0","Myd88"
"Rabgef1",6.3848780685899e-14,0.293153939241054,0.569,0.377,1.07661813
992563e-09,"0","Rabgef1"
"Senp6",6.42352844659462e-14,0.309802523647265,0.625,0.436,1.083135366
66478e-09,"0","Senp6"
"Nrip1",6.75778621274241e-14,0.279966888908973,0.767,0.556,1.139497911
19262e-09,"0","Nrip1"
"Dnajb4",6.93235020211353e-14,0.355265010829595,0.478,0.303,1.16893289
108038e-09,"0","Dnajb4"
"Il6st",7.30217148238095e-14,0.315788280298574,0.505,0.31,1.2312921553
5908e-09,"0","Il6st"
"Tmem63a",7.38797913041398e-14,0.273715376800003,0.561,0.361,1.2457610
4097041e-09,"0","Tmem63a"
"Zdhhc20",7.48219798083598e-14,0.310004708460912,0.613,0.429,1.2616482

2352856e-09,"0","Zdhhc20"
"Mgat1",7.63338139464326e-14,0.346422929646335,0.556,0.379,1.287140770
76475e-09,"0","Mgat1"
"Bin2",8.25907199561995e-14,0.292006344279054,0.929,0.738,1.3926447199
0144e-09,"0","Bin2"
"Klf7",8.4887509357243e-14,0.291741943797411,0.583,0.414,1.43137318278
183e-09,"0","Klf7"
"Ash1l",8.84652746227831e-14,0.389042611264399,0.654,0.487,1.491701460
68937e-09,"0","Ash1l"
"Kin",9.10548069492674e-14,0.292986312760319,0.358,0.197,1.53536615477
855e-09,"0","Kin"
"Azin1",9.11384063581958e-14,0.35036643500402,0.605,0.434,1.5367758080
119e-09,"0","Azin1"
"Otud1",9.37581594252052e-14,0.2966639247673,0.292,0.138,1.58095008422
781e-09,"0","Otud1"
"Mta2",9.6911412379987e-14,0.299506880571051,0.485,0.303,1.63412023555
134e-09,"0","Mta2"
"Zfp513",9.72542718400437e-14,0.26734733133649,0.348,0.187,1.639901531
76682e-09,"0","Zfp513"
"Slc15a4",9.85903232267045e-14,0.302445923071275,0.632,0.446,1.6624300
3024869e-09,"0","Slc15a4"
"Ecscr",1.05705837164875e-13,0.263713331345939,0.806,0.556,1.782411826
27412e-09,"0","Ecscr"
"Apbb1ip",1.06025337646907e-13,0.278688484819827,0.949,0.79,1.78779924
340214e-09,"0","Apbb1ip"
"Ppp1r9a",1.06335370127401e-13,0.329005563327696,0.515,0.332,1.7930270
1108824e-09,"0","Ppp1r9a"
"Eif3j1",1.09945321183091e-13,0.289322696714297,0.532,0.354,1.85389800
578927e-09,"0","Eif3j1"
"Saraf",1.15467384569131e-13,0.253296561817584,0.9,0.718,1.94701103860
469e-09,"0","Saraf"
"Rgl2",1.17021217681336e-13,0.260007216587211,0.468,0.295,1.9732117725
4268e-09,"0","Rgl2"
"Colgalt1",1.28075429132536e-13,0.288013951269711,0.728,0.56,2.1596078
8603283e-09,"0","Colgalt1"
"Rbm26",1.3132616836856e-13,0.321039800213171,0.569,0.386,2.2144218510
3066e-09,"0","Rbm26"
"Srf",1.41632072795885e-13,0.268635910783872,0.338,0.18,2.388200011484
21e-09,"0","Srf"
"Kansl1l",1.58330006920864e-13,0.287072214739032,0.578,0.383,2.6697605
7669961e-09,"0","Kansl1l"
"Mgat4a",1.58633548460189e-13,0.334458116661454,0.679,0.496,2.67487889
41357e-09,"0","Mgat4a"
"Spint1",1.6159053169786e-13,0.298130142256807,0.743,0.526,2.724739545
48932e-09,"0","Spint1"
"Irf8",1.70169945045114e-13,0.305775344778875,0.968,0.798,2.8694056133
5071e-09,"0","Irf8"
"Nasp",1.7325442259188e-13,0.265911279350845,0.554,0.363,2.92141607374
428e-09,"0","Nasp"
"Vgll4",1.74151891198407e-13,0.289790161351414,0.544,0.364,2.936549189

38753e-09,"0","Vgll4"
"B4galt3",1.77747494742052e-13,0.2596150165671,0.321,0.172,2.997178256
34048e-09,"0","B4galt3"
"Nav2",1.8164110610243e-13,0.348339919484421,0.397,0.23,3.062832331099
18e-09,"0","Nav2"
"H2-
Q4",1.91274730066494e-13,0.28103554984224,0.507,0.332,3.22527449838122
e-09,"0","H2-Q4"
"Tubb2b",2.22417018950206e-13,0.275964228671441,0.368,0.201,3.75039577
353838e-09,"0","Tubb2b"
"Liph",2.23566035235613e-13,0.290927730086051,0.353,0.191,3.7697704861
4291e-09,"0","Liph"
"Tbc1d22a",2.48812804421877e-13,0.275988470895438,0.431,0.263,4.195481
50816169e-09,"0","Tbc1d22a"
"Armc5",2.52730060279095e-13,0.271503413939866,0.392,0.227,4.261534276
42611e-09,"0","Armc5"
"Hnrnpl",2.54539209183015e-13,0.296301685732605,0.672,0.494,4.29204014
5244e-09,"0","Hnrnpl"
"Pald1",3.01971646664808e-13,0.303636430827188,0.576,0.386,5.091845906
062e-09,"0","Pald1"
"Lyl1",3.04679770205328e-13,0.264457432446833,0.632,0.444,5.1375102852
0224e-09,"0","Lyl1"
"Mepce",3.12021238726935e-13,0.275054899951747,0.38,0.22,5.26130212741
358e-09,"0","Mepce"
"Rrbp1",3.27115333802399e-13,0.319151208405677,0.975,0.824,5.515818758
57605e-09,"0","Rrbp1"
"Cyth2",3.3013988597288e-13,0.257594424796935,0.505,0.329,5.5668187572
747e-09,"0","Cyth2"
"Ttc28",3.32610881194833e-13,0.340020963635928,0.559,0.386,5.608484678
70728e-09,"0","Ttc28"
"Tug1",3.44805927647294e-13,0.35147892606433,0.456,0.293,5.81411755198
867e-09,"0","Tug1"
"Mdm2",3.85666041660928e-13,0.305167876789903,0.554,0.38,6.50310079448
656e-09,"0","Mdm2"
"Aim2",3.95888441776654e-13,0.273035783065971,0.618,0.421,6.6754709052
3795e-09,"0","Aim2"
"Snhg12",3.97222700733817e-13,0.354366648756417,0.36,0.21,6.6979691797
7361e-09,"0","Snhg12"
"Prpf4b",4.78333130827535e-13,0.334288028578192,0.586,0.416,8.06565325
20139e-09,"0","Prpf4b"
"Nab2",5.03840477095798e-13,0.313167446238729,0.412,0.248,8.4957581247
8935e-09,"0","Nab2"
"Nmt1",5.20516634815198e-13,0.292300231063737,0.828,0.643,8.7769514962
5387e-09,"0","Nmt1"
"Asb2",5.73299610073273e-13,0.266423460835638,0.395,0.23,9.66697802505
553e-09,"0","Asb2"
"Entpd1",5.96979082124757e-13,0.329825777690679,0.713,0.531,1.00662612
827877e-08,"0","Entpd1"
"Dusp6",6.03566499353662e-13,0.325766345594291,0.522,0.347,1.017733831
21014e-08,"0","Dusp6"

"Fscn1",6.0919583001853e-13,0.268576375329893,0.684,0.472,1.0272260085
7724e-08,"0","Fscn1"
"Plau",6.26192651895608e-13,0.422407601246366,0.331,0.179,1.0558860496
2637e-08,"0","Plau"
"Jup",6.4020959056534e-13,0.26184175486722,0.358,0.199,1.0795214116112
8e-08,"0","Jup"
"Vamp3",7.06897497912566e-13,0.264818849099407,0.716,0.539,1.191970560
98017e-08,"0","Vamp3"
"Nfya",7.65335723178458e-13,0.346968534769069,0.51,0.334,1.29050909642
352e-08,"0","Nfya"
"Slc25a33",7.68312188621335e-13,0.295541747432689,0.385,0.223,1.295528
01245329e-08,"0","Slc25a33"
"Gem",8.31935352744311e-13,0.494968129729609,0.591,0.402,1.40280939179
746e-08,"0","Gem"
"Rock2",8.77779187295456e-13,0.261823631634843,0.755,0.573,1.480111265
6176e-08,"0","Rock2"
"Tra2a",8.81462735341074e-13,0.329552571626337,0.75,0.567,1.4863224643
3212e-08,"0","Tra2a"
"Poldip3",9.74259013706122e-13,0.320948091337438,0.407,0.251,1.6427955
4891126e-08,"0","Poldip3"
"Arid1a",9.91010069456678e-13,0.330828793261073,0.662,0.481,1.67104117
911785e-08,"0","Arid1a"
"Chn2",1.02394789290623e-12,0.291369064719429,0.404,0.243,1.7265809370
1849e-08,"0","Chn2"
"Sh2b3",1.07458462510782e-12,0.298580940649002,0.547,0.377,1.811964594
85681e-08,"0","Sh2b3"
"Slc25a25",1.1669260329333e-12,0.349057457767223,0.39,0.231,1.96767067
673214e-08,"0","Slc25a25"
"Med7",1.17035380393542e-12,0.302230284178148,0.505,0.336,1.9734505841
9591e-08,"0","Med7"
"Zbtb4",1.22516051667367e-12,0.311618852150241,0.466,0.297,2.065865663
21514e-08,"0","Zbtb4"
"Pitpnc1",1.23308203650003e-12,0.329110466881968,0.453,0.29,2.07922292
994635e-08,"0","Pitpnc1"
"Ankrd17",1.24199741161315e-12,0.306205847582652,0.667,0.474,2.0942560
3546209e-08,"0","Ankrd17"
"Sh2b2",1.24596438391244e-12,0.30483093370746,0.475,0.297,2.1009451441
5316e-08,"0","Sh2b2"
"Rbm5",1.30441585335478e-12,0.326736142253389,0.679,0.506,2.1995060119
2682e-08,"0","Rbm5"
"Arhgap25",1.38186209999786e-12,0.308046775451576,0.598,0.437,2.330095
87301639e-08,"0","Arhgap25"
"Atxn2l",1.57472349610858e-12,0.270659180081016,0.51,0.341,2.655298759
13829e-08,"0","Atxn2l"
"Hnrnpa0",1.81640468534411e-12,0.257756510790768,0.973,0.821,3.0628215
8042724e-08,"0","Hnrnpa0"
"N4bp2l2",1.82029071055873e-12,0.269022400934532,0.495,0.335,3.0693741
9614413e-08,"0","N4bp2l2"
"Itpr2",2.00430227295087e-12,0.276133180001471,0.588,0.412,3.379654492
64976e-08,"0","Itpr2"

"2610037D02Rik",2.0300362349632e-12,0.265454234344709,0.35,0.198,3.42304709939494e-08,"0","2610037D02Rik"
"Ifitm10",2.12373799534896e-12,0.318725935579569,0.333,0.183,3.58104700775742e-08,"0","Ifitm10"
"Bclaf1",2.14089130394568e-12,0.310236440464071,0.711,0.529,3.6099709167132e-08,"0","Bclaf1"
"Gatad2b",2.41986544158209e-12,0.306741809252198,0.6,0.422,4.08037710759573e-08,"0","Gatad2b"
"Neu1",2.45878847865665e-12,0.283414888521763,0.627,0.455,4.14600913271084e-08,"0","Neu1"
"Ctsf",2.46217795351251e-12,0.254595097709577,0.824,0.624,4.1517244652128e-08,"0","Ctsf"
"Sla",2.61461613771402e-12,0.272254474768238,0.544,0.373,4.40876573141337e-08,"0","Sla"
"Chst1",2.69866476001412e-12,0.288954390020202,0.551,0.367,4.55048851833581e-08,"0","Chst1"
"Phf20l1",2.72032335527348e-12,0.26134986944458,0.581,0.414,4.58700924166215e-08,"0","Phf20l1"
"Akirin1",2.73506513567391e-12,0.319360518577341,0.721,0.543,4.61186683177335e-08,"0","Akirin1"
"Gm26740",2.90941126949527e-12,0.271059083646248,0.529,0.351,4.90584928262293e-08,"0","Gm26740"
"Npc1",3.28271568965407e-12,0.304164237201329,0.574,0.403,5.53531519589469e-08,"0","Npc1"
"Tgif1",3.91051773499516e-12,0.307828283399372,0.897,0.667,6.59391500474884e-08,"0","Tgif1"
"Clip1",3.93369890856914e-12,0.398923632696194,0.672,0.519,6.63300309962928e-08,"0","Clip1"
"Batf3",4.38813811740902e-12,0.28344955479561,0.324,0.177,7.3992784935751e-08,"0","Batf3"
"Lbh",4.71995559621875e-12,0.35251160529791,0.402,0.243,7.95878912634405e-08,"0","Lbh"
"Nfat5",4.90472094320628e-12,0.314279979077533,0.61,0.432,8.27034045443443e-08,"0","Nfat5"
"Tpp2",5.39617686120675e-12,0.333148878730938,0.598,0.433,9.09903342336683e-08,"0","Tpp2"
"Taf1d",5.40288273940929e-12,0.351749361116509,0.529,0.371,9.11034087519194e-08,"0","Taf1d"
"Mfng",5.40796521919048e-12,0.271750210854729,0.566,0.388,9.11891095259898e-08,"0","Mfng"
"Susd3",5.42512243087021e-12,0.278128264846921,0.73,0.556,9.14784144293334e-08,"0","Susd3"
"Dock4",5.54286923579101e-12,0.27075266003903,0.728,0.537,9.34638610539079e-08,"0","Dock4"
"Ankrd11",6.07191080878191e-12,0.250265314528589,0.873,0.668,1.02384560057681e-07,"0","Ankrd11"
"Nuak1",6.14081280712031e-12,0.324050529070474,0.436,0.276,1.03546385553663e-07,"0","Nuak1"
"Gtf2a1",6.41839975856855e-12,0.278261756120544,0.417,0.259,1.08227056728983e-07,"0","Gtf2a1"

"Tmem251",6.67393180169405e-12,0.365873741502867,0.475,0.319,1.1253583
8040165e-07,"0","Tmem251"
"Dmtf1",7.25805642258992e-12,0.293325676128672,0.377,0.226,1.223853473
97711e-07,"0","Dmtf1"
"Chka",8.79115318316323e-12,0.460816250977372,0.471,0.314,1.4823642497
4498e-07,"0","Chka"
"Lysmd3",9.0156700455333e-12,0.317261215598388,0.304,0.168,1.520222283
07783e-07,"0","Lysmd3"
"Ubxn4",9.70378193270882e-12,0.273464000282969,0.696,0.53,1.6362517094
9336e-07,"0","Ubxn4"
"Vav2",1.04403630469665e-11,0.266865738513871,0.48,0.316,1.76045401697
949e-07,"0","Vav2"
"Vps4b",1.04555855297692e-11,0.251707044403048,0.493,0.338,1.763020832
02969e-07,"0","Vps4b"
"Rnf111",1.54331092458473e-11,0.255292688827669,0.444,0.286,2.60233088
103478e-07,"0","Rnf111"
"Gnl3",1.55770706522746e-11,0.302539196801042,0.471,0.308,2.6266056533
8655e-07,"0","Gnl3"
"Eef2k",1.62314557779695e-11,0.261393510159505,0.49,0.329,2.7369480732
8122e-07,"0","Eef2k"
"Akap8",1.67424730985219e-11,0.260879865028261,0.414,0.264,2.823115813
87276e-07,"0","Akap8"
"Wasf2",1.71216976182321e-11,0.27627614923095,0.863,0.689,2.8870606523
863e-07,"0","Wasf2"
"Tbc1d10a",2.04139303837955e-11,0.254857145958536,0.434,0.277,3.442196
9413156e-07,"0","Tbc1d10a"
"Tor1aip1",2.09126048274474e-11,0.258937857665646,0.814,0.634,3.526283
42600419e-07,"0","Tor1aip1"
"Gxylt1",2.10053766711938e-11,0.304034142366129,0.453,0.295,3.54192661
42967e-07,"0","Gxylt1"
"Pik3cd",2.15070001775931e-11,0.315815218622562,0.473,0.333,3.62651036
994576e-07,"0","Pik3cd"
"Zfp710",2.20119171453098e-11,0.280276859020185,0.564,0.405,3.71164946
904215e-07,"0","Zfp710"
"Nars",2.36100688446114e-11,0.259121055773744,0.696,0.528,3.9811298085
7837e-07,"0","Nars"
"2810474O19Rik",2.41196199210672e-11,0.32831188044098,0.581,0.419,4.06
705031109035e-07,"0","2810474O19Rik"
"Ppp1r15b",2.51814341818579e-11,0.260366279646758,0.453,0.296,4.246093
43174489e-07,"0","Ppp1r15b"
"Akap8l",2.5349501959999e-11,0.265838215794531,0.412,0.269,4.274433020
49503e-07,"0","Akap8l"
"Srrm1",2.57787977421696e-11,0.285993964583605,0.745,0.574,4.346820875
28464e-07,"0","Srrm1"
"Sertad2",2.89052112844364e-11,0.276556768901945,0.39,0.241,4.87399672
678167e-07,"0","Sertad2"
"Stard9",2.89826599607421e-11,0.259366763328857,0.461,0.297,4.88705612
258034e-07,"0","Stard9"
"Epc1",3.00147251494115e-11,0.266696594777278,0.363,0.215,5.0610829546
9377e-07,"0","Epc1"

"Gripap1",3.29471231174363e-11,0.289809732762381,0.48,0.326,5.55554390
00621e-07,"0","Gripap1"
"Rhoh",3.41404562318874e-11,0.279830443464279,0.667,0.496,5.7567637298
2086e-07,"0","Rhoh"
"Rnf2",3.66736775978548e-11,0.258940469246753,0.52,0.364,6.18391551655
028e-07,"0","Rnf2"
"Zbtb2",3.8279405434347e-11,0.298965219306107,0.304,0.165,6.4546733443
3959e-07,"0","Zbtb2"
"Mex3c",4.09982861783501e-11,0.277096925984502,0.446,0.284,6.913131015
3934e-07,"0","Mex3c"
"Dgkd",4.18237391613845e-11,0.293024380091074,0.583,0.414,7.0523188973
9266e-07,"0","Dgkd"
"Zmiz1",4.43743827837882e-11,0.278260055663214,0.699,0.53,7.4824084250
0236e-07,"0","Zmiz1"
"Itgam",4.45132500683041e-11,0.31138119978168,0.946,0.77,7.50582422651
744e-07,"0","Itgam"
"Arsb",4.55796610913973e-11,0.259359293593195,0.725,0.515,7.6856424532
3142e-07,"0","Arsb"
"Cdk9",5.07191363313859e-11,0.289038403366591,0.652,0.496,8.5522607681
9829e-07,"0","Cdk9"
"Gna15",5.43275340693458e-11,0.251323067227351,0.613,0.45,9.1607087947
731e-07,"0","Gna15"
"Bcl3",5.59361857612276e-11,0.305373453777292,0.348,0.208,9.4319596430
5819e-07,"0","Bcl3"
"Bud31",5.7302295104673e-11,0.303268623667392,0.561,0.416,9.6623130005
4997e-07,"0","Bud31"
"Srek1",6.48540876939083e-11,0.273226555186444,0.463,0.314,1.093569626
69468e-06,"0","Srek1"
"Arl4c",6.62132545602528e-11,0.339598537795018,0.836,0.652,1.116487898
39498e-06,"0","Arl4c"
"Dock10",7.30527030413819e-11,0.254184043969484,0.789,0.599,1.23181467
868378e-06,"0","Dock10"
"Gnaq",7.46996417129834e-11,0.298424193500587,0.566,0.398,1.2595853585
6433e-06,"0","Gnaq"
"Cbx4",7.9460235910658e-11,0.331246975243211,0.407,0.262,1.33985849792
552e-06,"0","Cbx4"
"Ifnar1",9.20124508191146e-11,0.290420371142175,0.466,0.314,1.55151394
571191e-06,"0","Ifnar1"
"Ppp2r2a",9.83614582326634e-11,0.320945642365681,0.529,0.386,1.6585709
0871917e-06,"0","Ppp2r2a"
"Cdk12",9.91316582156048e-11,0.312652561466593,0.561,0.421,1.671558020
83153e-06,"0","Cdk12"
"Gpr146",1.09028617473991e-10,0.252207793133346,0.358,0.208,1.83844054
784644e-06,"0","Gpr146"
"Trpc4ap",1.15243693900938e-10,0.257790992581671,0.586,0.437,1.9432391
6655761e-06,"0","Trpc4ap"
"Pi4k2a",1.4284484609309e-10,0.260068208692427,0.441,0.295,2.408649794
82169e-06,"0","Pi4k2a"
"Flcn",1.42894476565917e-10,0.275720098945328,0.338,0.203,2.4094866638
5449e-06,"0","Flcn"

"Lncpint",1.45494946376657e-10,0.324983239024967,0.4,0.252,2.45333578580319e-06,"0","Lncpint"
"Lrba",1.50033001527838e-10,0.256260476026418,0.321,0.181,2.5298564717624e-06,"0","Lrba"
"Cebpd",1.6268337118966e-10,0.434755972899923,0.571,0.411,2.74316700500005e-06,"0","Cebpd"
"Oser1",1.68336675416606e-10,0.260810633271858,0.451,0.299,2.8384930208748e-06,"0","Oser1"
"Btaf1",1.68506317065607e-10,0.26842158594365,0.409,0.269,2.84135351836026e-06,"0","Btaf1"
"Itga6",1.69707874815746e-10,0.27903767356372,0.75,0.569,2.8616141851431e-06,"0","Itga6"
"Man1a2",1.70850732767162e-10,0.262440877829638,0.699,0.542,2.88088505591988e-06,"0","Man1a2"
"Plekho1",1.7400466040843e-10,0.260815258748584,0.877,0.725,2.93406658380695e-06,"0","Plekho1"
"Nfkbib",1.98798572563003e-10,0.257331037606876,0.676,0.521,3.35214153055735e-06,"0","Nfkbib"
"Sbno1",2.15040212207616e-10,0.300033178677297,0.578,0.433,3.62600805824482e-06,"0","Sbno1"
"Cd84",2.68678459228509e-10,0.290149764060055,0.809,0.626,4.53045617951113e-06,"0","Cd84"
"Ip6k1",2.79160061952218e-10,0.256680336360756,0.4,0.257,4.70719696463829e-06,"0","Ip6k1"
"Traf5",3.04023855668531e-10,0.250877048897053,0.328,0.191,5.12645025428276e-06,"0","Traf5"
"Mier1",3.12075541023948e-10,0.255457345339566,0.566,0.407,5.26221777274581e-06,"0","Mier1"
"Wwc2",3.30265389611517e-10,0.256905968177774,0.407,0.263,5.5689349996294e-06,"0","Wwc2"
"Cul1",3.32047449492519e-10,0.269306344836507,0.642,0.491,5.59898409334286e-06,"0","Cul1"
"Cdip1",3.63801709457638e-10,0.251529094933429,0.324,0.191,6.13442442487469e-06,"0","Cdip1"
"Sgk1",3.80842058113451e-10,0.321513541759832,0.703,0.52,6.421758783909e-06,"0","Sgk1"
"Maml3",4.08987647891843e-10,0.297279779540258,0.407,0.269,6.89634971875226e-06,"0","Maml3"
"Sparc",4.44627570021722e-10,0.284099212688077,0.995,0.8,7.49731008570628e-06,"0","Sparc"
"Tmem245",6.18861869826454e-10,0.251376418527976,0.392,0.249,1.04352488490137e-05,"0","Tmem245"
"Ophn1",8.27260430625377e-10,0.270527236348269,0.645,0.489,1.39492653812051e-05,"0","Ophn1"
"Zfp131",8.65327136518359e-10,0.284364184745227,0.412,0.271,1.45911461759726e-05,"0","Zfp131"
"Adam10",8.71800649379245e-10,0.270499499809457,0.691,0.536,1.47003025498328e-05,"0","Adam10"
"Fam46a",1.52897549333614e-09,0.283093315802787,0.537,0.383,2.5781584768634e-05,"0","Fam46a"

"BC017643",2.05314134624621e-09,0.255086667104028,0.439,0.299,3.46200693804036e-05,"0","BC017643"
"Kcnk6",2.09929357774349e-09,0.265930360132677,0.48,0.337,3.53982883079107e-05,"0","Kcnk6"
"Vav1",2.25050318864188e-09,0.253015366649659,0.77,0.619,3.79479847668795e-05,"0","Vav1"
"Arl5b",2.30536340433561e-09,0.25203589435648,0.429,0.293,3.88730377239071e-05,"0","Arl5b"
"G3bp1",2.30691875069192e-09,0.255431129561813,0.77,0.598,3.88992639741671e-05,"0","G3bp1"
"BC005561",2.55039813396543e-09,0.316219099199701,0.449,0.307,4.30048133349251e-05,"0","BC005561"
"Tnrc6b",2.78731076631338e-09,0.280121862699245,0.561,0.42,4.69996341415762e-05,"0","Tnrc6b"
"Macf1",3.05158983072302e-09,0.262212414777037,0.875,0.685,5.14559077256516e-05,"0","Macf1"
"Bbc3",3.31840757926704e-09,0.283878383995269,0.309,0.183,5.59549886016008e-05,"0","Bbc3"
"Yrdc",3.64613936149362e-09,0.250522023617398,0.429,0.299,6.14812019135054e-05,"0","Yrdc"
"Pnisr",3.85324194015437e-09,0.252884366617558,0.691,0.557,6.49733655948831e-05,"0","Pnisr"
"Cdk6",4.34950502991155e-09,0.252964471870524,0.444,0.297,7.33413538143685e-05,"0","Cdk6"
"Wac",4.40998160263263e-09,0.25767008969384,0.544,0.416,7.43611097835913e-05,"0","Wac"
"Gcnt1",4.51620461823987e-09,0.279403455778958,0.417,0.277,7.61522422727608e-05,"0","Gcnt1"
"Fam133b",4.54527311320899e-09,0.255779283495063,0.426,0.286,7.66423952349301e-05,"0","Fam133b"
"Vps18",5.33270361898266e-09,0.335251133586922,0.453,0.329,8.99200484232857e-05,"0","Vps18"
"Hpgd",5.50139515051877e-09,0.260866180314646,0.824,0.645,9.27645250280475e-05,"0","Hpgd"
"Ddx3y",5.78668372865388e-09,0.31775152319848,0.461,0.331,9.75750610325618e-05,"0","Ddx3y"
"Setd2",1.01583270313919e-08,0.250510873667409,0.507,0.366,0.00017128971040333,"0","Setd2"
"Spty2d1",1.27477067638581e-08,0.289031297402901,0.498,0.37,0.000214951831452175,"0","Spty2d1"
"Lpcat1",1.37967683190744e-08,0.250599092971479,0.424,0.292,0.000232641107396233,"0","Lpcat1"
"Prdm2",1.40486003441573e-08,0.270314122175607,0.392,0.279,0.000236887499003181,"0","Prdm2"
"Slc26a2",1.68843041144296e-08,0.282866916524844,0.328,0.213,0.000284703135977512,"0","Slc26a2"
"Rgs2",2.08223429222969e-08,0.31847793202146,0.657,0.495,0.000351106346355771,"0","Rgs2"
"Mef2d",2.32919158440003e-08,0.265768011585813,0.485,0.357,0.000392748284961532,"0","Mef2d"

"Arih2",2.72033070412793e-08,0.276933585615994,0.507,0.383,0.000458702
163330052,"0","Arih2"
"Cebpz",2.9219496378079e-08,0.261080038864992,0.583,0.445,0.0004926991
47927168,"0","Cebpz"
"Pknox1",3.18275716265391e-08,0.269628021152967,0.316,0.202,0.00053667
6512766702,"0","Pknox1"
"Dusp3",3.24073810602207e-08,0.28750109574067,0.556,0.444,0.0005464532
59437441,"0","Dusp3"
"Hsp90aa1",3.71790746120415e-08,0.309142023910604,0.919,0.8,0.00062691
3556108244,"0","Hsp90aa1"
"Sbds",3.96803651685021e-08,0.280367099293346,0.512,0.391,0.0006690903
17471282,"0","Sbds"
"Polr2a",4.51226441599759e-08,0.289265103187811,0.444,0.332,0.00076085
8025825514,"0","Polr2a"
"Abca9",5.30998925497421e-08,0.259366207151366,0.544,0.416,0.000895370
388173752,"0","Abca9"
"Gclc",5.57245184016469e-08,0.261990204963454,0.392,0.272,0.0009396268
2928857,"0","Gclc"
"Esco1",5.69072377227819e-08,0.252306941150714,0.319,0.204,0.000959569
842481548,"0","Esco1"
"Dnmt3a",7.04363698595601e-08,0.2822652960871,0.554,0.423,0.0011876980
685719,"0","Dnmt3a"
"Arl13b",7.64007655061694e-08,0.290634424195601,0.321,0.21,0.001288269
70796503,"0","Arl13b"
"B4galt1",9.02258466636008e-08,0.264851759333281,0.48,0.355,0.00152138
822644164,"0","B4galt1"
"Rnf167",1.36844657900427e-07,0.259937671658514,0.392,0.28,0.002307474
62151699,"0","Rnf167"
"Gadd45g",4.45002159930803e-07,0.358683044230961,0.27,0.166,0.00750362
64207532,"0","Gadd45g"
"Luzp1",1.77659377691944e-06,0.251466462340336,0.385,0.286,0.029956924
2664156,"0","Luzp1"
"Impact",1.22229681091513e-05,0.309032283618028,0.304,0.219,0.20610368
825651,"0","Impact"
"Adrb2",1.80111734731403e-05,0.31047429566875,0.505,0.401,0.3037044071
04091,"0","Adrb2"
"Klf10",4.7830294147303e-05,0.290988821447501,0.306,0.223,0.8065144199
11823,"0","Klf10"
"Lmna",0.000396287702350794,0.26344420167624,0.324,0.244,1,"0","Lmna"
"Mt1",0.000846259032747782,0.320972625367584,0.647,0.588,1,"0","Mt1"
"Xist",0.00201273144039878,0.31864165500492,0.346,0.28,1,"0","Xist"
"Nfkbia",2.65533840644532e-60,0.912572990494412,1,0.827,4.477431620948
09e-56,"1","Nfkbia"
"Gpr84",7.85138426136222e-54,1.0852089612253,0.988,0.679,1.32390041415
09e-49,"1","Gpr84"
"Gadd45b",5.67678882474295e-50,0.94064134490969,0.96,0.615,9.572201316
28156e-46,"1","Gadd45b"
"Arl5c",8.2166155735808e-49,1.3160250767422,0.839,0.49,1.3854857180172
e-44,"1","Arl5c"
"Cst3",7.35513557687333e-45,0.626320604448808,1,0.991,1.24022296097238

e-40,"1","Cst3"
"Sparc1",1.67677632400122e-42,0.705263280520871,0.997,0.807,2.82738023
753085e-38,"1","Sparc"
"Ppp1r15a1",2.48376059817047e-39,0.762219086997975,0.965,0.707,4.18811
712063505e-35,"1","Ppp1r15a"
"Tnf",6.34377166380242e-39,0.907106964869106,0.963,0.652,1.06968677795
036e-34,"1","Tnf"
"Tnfaip3",9.5266117599547e-39,0.774087894735045,0.974,0.659,1.60637727
496356e-34,"1","Tnfaip3"
"Bcl2a1b",1.63721561815449e-38,0.835362330942275,0.991,0.835,2.7606729
753321e-34,"1","Bcl2a1b"
"Icam11",3.23536869434388e-38,0.797857586676466,0.977,0.692,5.45547869
240265e-34,"1","Icam1"
"Cd83",3.35738238600526e-38,0.738781537207247,0.997,0.802,5.6612181792
8208e-34,"1","Cd83"
"Ctss",8.39304201516906e-37,0.451482237400375,1,0.98,1.41523474459781e
-32,"1","Ctss"
"Il1a1",7.6166654736479e-36,0.767885964170282,0.991,0.711,1.2843221321
6651e-31,"1","Il1a"
"Itgb51",1.64333680429203e-34,0.523880845347182,0.988,0.843,2.77099451
939721e-30,"1","Itgb5"
"P2ry121",2.3789235486295e-32,0.601924203873768,0.988,0.766,4.01134088
769906e-28,"1","P2ry12"
"Siglech1",2.60322639822056e-31,0.586291858475017,0.942,0.652,4.389560
35267951e-27,"1","Siglech"
"Nfkbiz1",3.36331285780484e-31,0.63467164977642,0.994,0.738,5.67121814
083053e-27,"1","Nfkbiz"
"Dusp2",4.98099455880313e-31,1.01221532613403,0.804,0.521,8.3989530250
5385e-27,"1","Dusp2"
"Arhgap51",5.36112198382388e-31,0.53894468295014,0.916,0.652,9.0399238
8912382e-27,"1","Arhgap5"
"Junb1",9.89252680629847e-30,0.508115429961798,1,0.864,1.6680778700780
5e-25,"1","Junb"
"Selplg1",2.28911228533015e-29,0.543211967297951,0.986,0.827,3.8599011
3552369e-25,"1","Selplg"
"Ppp1r101",4.71167620725658e-29,0.601055041524457,0.902,0.636,7.944828
42067604e-25,"1","Ppp1r10"
"Ier21",5.10291874663671e-29,0.620071709780353,0.994,0.814,8.604541590
57883e-25,"1","Ier2"
"Egr11",1.02609524336986e-27,0.565089342803441,1,0.751,1.7302017993702
6e-23,"1","Egr1"
"Ccrl2",2.52580542060785e-27,0.567076778878439,0.934,0.625,4.259013100
22896e-23,"1","Ccrl2"
"Clcf1",3.23238001792402e-27,0.705759948473065,0.478,0.214,5.450439186
22348e-23,"1","Clcf1"
"Lag31",2.12601967818614e-25,0.509569210670588,0.942,0.653,3.584894381
35747e-21,"1","Lag3"
"Socs31",2.62393896582803e-25,0.646411820498867,0.965,0.732,4.42448588
417923e-21,"1","Socs3"
"Marcks1",3.62219588274374e-25,0.41715503214218,0.997,0.906,6.10774669

74825e-21,"1","Marcks"
"Rpl18a",7.83194396585219e-25,0.48682030125938,0.991,0.944,1.320622391
522e-20,"1","Rpl18a"
"Cx3cr11",1.20083582068113e-24,0.496807966258949,0.977,0.831,2.0248493
6083252e-20,"1","Cx3cr1"
"Hexb1",2.83642297628791e-24,0.403621679514249,1,0.948,4.7827764226166
7e-20,"1","Hexb"
"Ncf1",3.60540025268485e-24,0.581379481250702,0.945,0.76,6.07942590607
719e-20,"1","Ncf1"
"Cd81",5.3200900718867e-24,0.44188549601837,1,0.865,8.97073587921534e-
20,"1","Cd81"
"Tgfbr11",5.51175708141639e-24,0.441421498106687,0.954,0.707,9.2939247
9068431e-20,"1","Tgfbr1"
"Nav31",6.28478155802872e-24,0.497150680264124,0.836,0.508,1.059739866
3148e-19,"1","Nav3"
"Sowahc1",2.35892346056599e-23,0.595450031694654,0.744,0.484,3.9776167
3920638e-19,"1","Sowahc"
"Plxdc21",9.64906888260737e-23,0.450408820063694,0.963,0.736,1.6270259
9498525e-18,"1","Plxdc2"
"Zfp622",1.54083166046608e-22,0.509131393017346,0.775,0.522,2.59815034
58779e-18,"1","Zfp622"
"Eef1a1",1.94900189794016e-22,0.256342272896311,1,0.991,3.286407000306
7e-18,"1","Eef1a1"
"Tmem1191",2.26451859625468e-22,0.446210945061353,0.939,0.67,3.8184312
5700464e-18,"1","Tmem119"
"Nlrp31",2.42797675237965e-22,0.443689280031119,0.942,0.64,4.094054399
86256e-18,"1","Nlrp3"
"Rps9",2.54122801937159e-22,0.359316452638604,0.994,0.968,4.2850186862
6438e-18,"1","Rps9"
"Epb41l21",3.94025050518546e-22,0.425014634014278,0.968,0.736,6.644050
40184372e-18,"1","Epb41l2"
"Rps21",5.97917042386414e-22,0.602485524391276,0.96,0.879,1.0082077168
7197e-17,"1","Rps21"
"Skil1",7.19193449399018e-22,0.487004375526225,0.977,0.76,1.2127039943
7662e-17,"1","Skil"
"Ly86",7.78813046975439e-22,0.357472887868459,0.991,0.948,1.3132345598
0998e-17,"1","Ly86"
"Rpl27a",1.31298089481871e-21,0.440622769366899,0.991,0.951,2.21394838
48433e-17,"1","Rpl27a"
"Rpl21",1.38551571795738e-21,0.518428460373384,0.988,0.936,2.336256603
61974e-17,"1","Rpl21"
"Rps11",4.3875086892534e-21,0.460250715121218,0.994,0.946,7.3982171518
1908e-17,"1","Rps11"
"Bcl2a1a",6.08028673443581e-21,0.592294939904949,0.608,0.349,1.0252579
4916057e-16,"1","Bcl2a1a"
"Ppfia41",8.68867803512042e-21,0.418631566208464,0.925,0.677,1.4650848
90282e-16,"1","Ppfia4"
"Fau",1.05633853136855e-20,0.557343575545963,0.997,0.964,1.78119803159
365e-16,"1","Fau"
"Chd41",1.2914503035064e-20,0.512554611417471,0.914,0.673,2.1776435017

7249e-16,"1","Chd4"
"Rpl6",1.50968315481435e-20,0.285860775496841,0.994,0.964,2.5456277356
4796e-16,"1","Rpl6"
"Fam102b1",2.11077183324337e-20,0.436433779105658,0.749,0.496,3.559183
46521496e-16,"1","Fam102b"
"Zfp361",2.52760457344853e-20,0.460113175390624,1,0.811,4.262046831748
92e-16,"1","Zfp36"
"Rps27",2.62741702230542e-20,0.604238935720958,0.942,0.861,4.430350583
01139e-16,"1","Rps27"
"Srgap21",4.47799875809237e-20,0.401577461344556,0.89,0.637,7.55080150
589535e-16,"1","Srgap2"
"Tgif11",1.05394060101485e-19,0.438532001130551,0.937,0.667,1.77715464
143124e-15,"1","Tgif1"
"Rgs10",1.67067403308909e-19,0.333272466816463,0.986,0.898,2.817090554
59483e-15,"1","Rgs10"
"Frmd4a1",2.49939434052605e-19,0.424980436868408,0.85,0.56,4.214478736
99503e-15,"1","Frmd4a"
"Rpl23",3.13022117813368e-19,0.469859556526757,0.986,0.947,5.278178950
56901e-15,"1","Rpl23"
"Marcksl11",3.68783268088886e-19,0.497781240716198,0.948,0.688,6.21842
34665148e-15,"1","Marcksl1"
"P2ry131",3.74604906006196e-19,0.396760117741764,0.839,0.604,6.3165879
2507648e-15,"1","P2ry13"
"Mob3c1",4.59050493781822e-19,0.460633526931937,0.686,0.429,7.74050942
614907e-15,"1","Mob3c"
"Zfhx31",6.08631367575416e-19,0.419756977255179,0.911,0.678,1.02627421
200567e-14,"1","Zfhx3"
"Ldhb",8.5351003013582e-19,0.423072036597272,0.974,0.775,1.43918861281
502e-14,"1","Ldhb"
"Sall11",1.3035555022141e-18,0.396636684311334,0.712,0.437,2.198055287
83342e-14,"1","Sall1"
"Rps7",1.49914009609778e-18,0.321189919870209,0.986,0.935,2.5278500300
4008e-14,"1","Rps7"
"Rpl39",2.06515903516085e-18,0.542180908441543,0.965,0.902,3.482271165
08823e-14,"1","Rpl39"
"Rplp1",2.13553885755324e-18,0.487959801070318,0.997,0.949,3.600945621
60627e-14,"1","Rplp1"
"Rps12",2.6232911726523e-18,0.480258897189867,0.98,0.944,4.42339357532
63e-14,"1","Rps12"
"Rpl30",2.84014984551223e-18,0.510054179787621,0.968,0.911,4.789060669
50273e-14,"1","Rpl30"
"Rps10",3.07418042920948e-18,0.389879544475428,0.988,0.947,5.183683039
73303e-14,"1","Rps10"
"Dennd4a1",3.661338058582e-18,0.418155840034768,0.942,0.704,6.17374823
438097e-14,"1","Dennd4a"
"Rpl37",3.91348327303799e-18,0.556788570408697,0.954,0.873,6.598915494
99666e-14,"1","Rpl37"
"Rps15",5.63915398562934e-18,0.496497342329805,0.939,0.84,9.5087414505
682e-14,"1","Rps15"
"Rps29",7.24774789551996e-18,0.52008881760685,0.994,0.943,1.2221152501

4258e-13,"1","Rps29"
"Rps25",7.29184938182258e-18,0.487481062516811,0.931,0.849,1.229551642
76292e-13,"1","Rps25"
"Rpl12",8.76933314644452e-18,0.338522172100751,0.98,0.929,1.4786849551
5347e-13,"1","Rpl12"
"Rpl34",9.93435371609197e-18,0.47879520871806,0.968,0.888,1.6751307236
0743e-13,"1","Rpl34"
"Kctd121",1.21342699631055e-17,0.458574472418843,0.991,0.808,2.0460806
0117885e-13,"1","Kctd12"
"Gem1",1.31634056764081e-17,0.552070000983455,0.64,0.398,2.21961346515
594e-13,"1","Gem"
"Adgrg11",1.38094762762737e-17,0.349012369389935,0.81,0.512,2.32855388
970528e-13,"1","Adgrg1"
"Rpl17",1.67176303659155e-17,0.369085722622643,0.997,0.942,2.818926832
30068e-13,"1","Rpl17"
"Rpl35a",1.7848262800193e-17,0.507276774018449,0.974,0.913,3.009574073
36854e-13,"1","Rpl35a"
"Btg21",2.07110952291681e-17,0.347937317078812,0.994,0.805,3.492304877
54233e-13,"1","Btg2"
"Serpine21",3.20356598764209e-17,0.362954521912668,0.957,0.696,5.40185
29683621e-13,"1","Serpine2"
"Rpl32",3.83853704502012e-17,0.419605863669139,0.986,0.941,6.472541165
31293e-13,"1","Rpl32"
"Ier3",3.93720550259035e-17,0.37404346260045,0.988,0.766,6.63891591846
784e-13,"1","Ier3"
"Rab6b",5.48255197953313e-17,0.298013159923688,0.429,0.217,9.244679147
88876e-13,"1","Rab6b"
"Pde3b1",6.45135652609499e-17,0.359051040787707,0.862,0.627,1.08782773
743014e-12,"1","Pde3b"
"Fscn11",6.53399581523327e-17,0.41016154725594,0.726,0.47,1.1017623743
6463e-12,"1","Fscn1"
"Rps15a",8.09680494363817e-17,0.468578779824753,0.983,0.913,1.36528324
959627e-12,"1","Rps15a"
"Rpl11",8.33370456289643e-17,0.33692527971739,0.988,0.942,1.4052292633
956e-12,"1","Rpl11"
"Irf1",1.94173427706223e-16,0.569787424661868,0.605,0.383,3.2741523379
8234e-12,"1","Irf1"
"Csf1r1",2.27853868977833e-16,0.302564743886956,1,0.934,3.842071938704
22e-12,"1","Csf1r"
"Smad7",2.42560102573674e-16,0.357478833126726,0.735,0.466,4.090048449
59728e-12,"1","Smad7"
"Cyfip1",2.49889830904978e-16,0.334011030061131,0.983,0.852,4.21364232
871975e-12,"1","Cyfip1"
"Tanc21",2.94235175031294e-16,0.423806472143038,0.824,0.572,4.96139352
137768e-12,"1","Tanc2"
"Herc2",3.43312955823117e-16,0.340232105133502,0.746,0.499,5.788943061
08939e-12,"1","Herc2"
"Golm11",3.51428159461249e-16,0.367466287579254,0.87,0.629,5.925781624
83558e-12,"1","Golm1"
"Pmepa11",4.49433305981376e-16,0.371114934632776,0.939,0.727,7.5783444

0545797e-12,"1","Pmepa1"
"Eef2",4.6363547374189e-16,0.274509248189096,0.997,0.946,7.81782135823
575e-12,"1","Eef2"
"Rplp2",4.67653254952641e-16,0.477942966571451,0.945,0.853,7.885569185
01143e-12,"1","Rplp2"
"Rpl10",4.75009754012033e-16,0.268682869730337,0.997,0.957,8.009614472
1509e-12,"1","Rpl10"
"Rpl22",5.62309574812306e-16,0.416685982231412,0.925,0.825,9.481664050
4851e-12,"1","Rpl22"
"Rpl26",6.32511135674902e-16,0.405964497499337,0.98,0.927,1.0665402769
7502e-11,"1","Rpl26"
"Tmcc31",7.2047087488545e-16,0.399706892655852,0.818,0.572,1.214857989
23185e-11,"1","Tmcc3"
"Rps14",7.92607998231811e-16,0.373776045145038,0.988,0.934,1.336495606
61848e-11,"1","Rps14"
"Ddx51",1.00481091674631e-15,0.280973805294917,1,0.925,1.6943121678176
3e-11,"1","Ddx5"
"Man2b1",1.04540083636198e-15,0.359972177036135,1,0.886,1.762754890273
57e-11,"1","Man2b1"
"Vsir1",1.25567338883604e-15,0.30319863064099,0.968,0.791,2.1173164682
5533e-11,"1","Vsir"
"Sdc4",1.3772053811109e-15,0.645924575258641,0.594,0.368,2.32224371362
919e-11,"1","Sdc4"
"Rpl37a",1.38907240415761e-15,0.499607721043918,0.963,0.887,2.34225388
789056e-11,"1","Rpl37a"
"Rpl36a",1.86577279211609e-15,0.508636556291954,0.85,0.745,3.146066082
06614e-11,"1","Rpl36a"
"Rcsd1",2.09777129525186e-15,0.465017426985722,0.643,0.428,3.537261958
05368e-11,"1","Rcsd1"
"Rps18",2.26702272627349e-15,0.351092167037375,0.965,0.911,3.822653721
04237e-11,"1","Rps18"
"Cmtm6",3.18225309209621e-15,0.30612170662503,0.905,0.718,5.3659151638
9262e-11,"1","Cmtm6"
"Wnk11",3.48022760866647e-15,0.348594872320574,0.957,0.774,5.868359793
73341e-11,"1","Wnk1"
"Rps23",4.0222787123373e-15,0.437709237081716,0.974,0.925,6.7823663647
4316e-11,"1","Rps23"
"Bcl2a1d",4.2368143090027e-15,0.412269055709625,0.573,0.36,7.144116287
84036e-11,"1","Bcl2a1d"
"Mrpl52",5.61371143394905e-15,0.35499212293478,0.677,0.469,9.465840219
92489e-11,"1","Mrpl52"
"Pag11",6.85332905352955e-15,0.296025788482664,0.712,0.474,1.155608345
00615e-10,"1","Pag1"
"Herpud11",6.93458826180004e-15,0.387062312083062,0.862,0.623,1.169310
27270472e-10,"1","Herpud1"
"Cd34",8.32686435261598e-15,0.317792569921,0.79,0.533,1.40407586713811
e-10,"1","Cd34"
"Rps20",1.07760523311723e-14,0.390370381827821,0.974,0.914,1.817057944
08228e-10,"1","Rps20"
"Dnajb41",1.12921471704704e-14,0.357671697414394,0.513,0.302,1.9040818

5588471e-10,"1","Dnajb4"
"Ecscr1",1.28322376156861e-14,0.311611087529229,0.827,0.561,2.16377190675699e-10,"1","Ecscr"
"Rrbp11",1.33700765879642e-14,0.344050902733194,0.963,0.833,2.25446231426253e-10,"1","Rrbp1"
"F11r1",1.35832506509131e-14,0.286124776730547,0.767,0.509,2.29040772475696e-10,"1","F11r"
"Rps13",1.46822296276019e-14,0.384266748152193,0.974,0.925,2.47571755980623e-10,"1","Rps13"
"Rps26",1.60641338536964e-14,0.453089965148649,0.931,0.846,2.70873425041029e-10,"1","Rps26"
"Tnfaip2",1.66493688575005e-14,0.56006382457033,0.576,0.36,2.80741657675173e-10,"1","Tnfaip2"
"Rps27a",2.80174822931446e-14,0.32543907388072,0.988,0.953,4.72430786427005e-10,"1","Rps27a"
"Rps8",2.82796435017814e-14,0.322389870349358,0.991,0.966,4.76851348727037e-10,"1","Rps8"
"Ccni",2.97254763322964e-14,0.294478945311498,0.784,0.561,5.01230981915183e-10,"1","Ccni"
"Rpl36",3.56099028146728e-14,0.404561064341141,0.916,0.816,6.00454181261013e-10,"1","Rpl36"
"Rps28",3.63512281110913e-14,0.486943614921079,0.89,0.79,6.12954408409222e-10,"1","Rps28"
"Ubash3b1",4.8245172095388e-14,0.320108564729031,0.772,0.553,8.13510091872432e-10,"1","Ubash3b"
"Tsc22d21",6.99564660177124e-14,0.341107468545037,0.807,0.575,1.17960592999067e-09,"1","Tsc22d2"
"Rps19",8.45227510246211e-14,0.341473619488637,0.971,0.926,1.42522262777716e-09,"1","Rps19"
"Macf11",1.16505941828697e-13,0.322621757941586,0.899,0.687,1.96452319111549e-09,"1","Macf1"
"Tmem63a1",1.42400317839009e-13,0.312813218588521,0.565,0.368,2.40115415940137e-09,"1","Tmem63a"
"Rps24",1.54769176001945e-13,0.344655808392932,0.988,0.949,2.60971784574479e-09,"1","Rps24"
"Pold4",1.58566074864456e-13,0.281706249620666,0.844,0.614,2.67374115436446e-09,"1","Pold4"
"Ccl4",1.66909841002076e-13,0.328992928888456,0.98,0.752,2.814433738977e-09,"1","Ccl4"
"Ski1",1.68036675104765e-13,0.259912247789331,0.833,0.606,2.83343441561654e-09,"1","Ski"
"Tnfaip8",1.68615867969624e-13,0.35915642570679,0.862,0.67,2.84320076570379e-09,"1","Tnfaip8"
"Rpl28",1.68776648114294e-13,0.358202608967929,0.957,0.882,2.84591184050323e-09,"1","Rpl28"
"Rpl29",1.77292140880524e-13,0.294822428706528,0.974,0.906,2.98950007952739e-09,"1","Rpl29"
"Rpl23a",1.86310365184685e-13,0.385975924815516,0.905,0.789,3.14156537774417e-09,"1","Rpl23a"
"Rpl35",2.19389786980069e-13,0.40633540149384,0.876,0.752,3.6993505880

5792e-09,"1","Rpl35"
"Ints61",2.20542136275213e-13,0.357220349423965,0.585,0.374,3.71878150
187265e-09,"1","Ints6"
"Rps16",2.25005794373158e-13,0.344445033331596,0.994,0.95,3.7940477047
2018e-09,"1","Rps16"
"Lpcat21",2.25201173814388e-13,0.28485434450665,0.983,0.836,3.79734219
285821e-09,"1","Lpcat2"
"Kdm6b1",2.95833836493089e-13,0.294763232510375,0.945,0.705,4.98835015
094646e-09,"1","Kdm6b"
"Itgam1",2.96471900106666e-13,0.326692868800097,0.942,0.778,4.99910917
95986e-09,"1","Itgam"
"Hk2",3.24028570836949e-13,0.371409740098302,0.738,0.549,5.46376976145
264e-09,"1","Hk2"
"Egr3",3.87229500845324e-13,0.399760550226772,0.573,0.351,6.5294638432
5386e-09,"1","Egr3"
"Tssc4",4.3241628838172e-13,0.340436146775843,0.507,0.316,7.2914034546
9256e-09,"1","Tssc4"
"Bmp2k1",4.36658074081494e-13,0.298852249440853,0.873,0.669,7.36292844
516214e-09,"1","Bmp2k"
"Rpl13a",4.6815006340688e-13,0.322767073690932,0.833,0.68,7.8939463691
6681e-09,"1","Rpl13a"
"Commd8",5.6649307477189e-13,0.256504010529246,0.801,0.603,9.552206226
80361e-09,"1","Commd8"
"Pabpc1",6.33350208353538e-13,0.256549433605172,0.991,0.909,1.06795512
132574e-08,"1","Pabpc1"
"Rps17",7.40937889650503e-13,0.318385956976105,0.873,0.73,1.2493694695
2868e-08,"1","Rps17"
"Mtdh1",8.94460147496216e-13,0.32255705345044,0.968,0.789,1.5082387007
0812e-08,"1","Mtdh"
"Dock41",1.01323156292655e-12,0.299574743659996,0.749,0.54,1.708511061
40675e-08,"1","Dock4"
"Dhx40",1.02403055873865e-12,0.472457077295002,0.476,0.293,1.726720328
14511e-08,"1","Dhx40"
"Rpl41",1.2314325244604e-12,0.394343137325017,0.954,0.889,2.0764415227
4512e-08,"1","Rpl41"
"Ptgs11",1.23389004580587e-12,0.321827604761301,0.945,0.751,2.08058539
523786e-08,"1","Ptgs1"
"Mef2a1",1.26284338069546e-12,0.259068688665417,0.948,0.737,2.12940650
852868e-08,"1","Mef2a"
"Dst1",1.48773533464644e-12,0.36616875231624,0.772,0.556,2.50861932128
083e-08,"1","Dst"
"Kcnk61",1.64595015357534e-12,0.300618221747076,0.536,0.33,2.775401148
95875e-08,"1","Kcnk6"
"Gm11808",1.71376038254586e-12,0.29840628240378,0.481,0.303,2.88974275
704883e-08,"1","Gm11808"
"Ttll31",1.90920939817068e-12,0.337816322498252,0.427,0.248,3.21930888
719539e-08,"1","Ttll3"
"Csrnp11",2.08328113925462e-12,0.327972240113822,0.879,0.646,3.5128286
5701114e-08,"1","Csrnp1"
"Tob21",2.17336282955476e-12,0.308172500492182,0.741,0.524,3.664724403

19523e-08,"1","Tob2"
"Ppp1r111",3.23980568025993e-12,0.357643384090714,0.66,0.475,5.4629603
3805429e-08,"1","Ppp1r11"
"Rpl38",5.586853669976e-12,0.371840704918813,0.902,0.811,9.42055265831
354e-08,"1","Rpl38"
"Ier51",7.46648726699885e-12,0.250671826548944,0.991,0.781,1.258999082
96135e-07,"1","Ier5"
"Rpl24",8.38734651970866e-12,0.321379090678135,0.96,0.89,1.41427437015
327e-07,"1","Rpl24"
"Tgfbr21",9.21176694862118e-12,0.305611110628514,0.824,0.632,1.5532881
428765e-07,"1","Tgfbr2"
"Bin21",9.63527190328595e-12,0.261432596356532,0.914,0.749,1.624699548
33208e-07,"1","Bin2"
"Cttnbp2nl1",9.68623478147131e-12,0.268372413789065,0.758,0.541,1.6332
9290885169e-07,"1","Cttnbp2nl"
"Klk8",1.19065944231437e-11,0.290969205097078,0.45,0.26,2.007689951630
48e-07,"1","Klk8"
"H2-
DMa",1.34767685789549e-11,0.285958639131779,0.847,0.646,2.272452717783
37e-07,"1","H2-DMa"
"Tnpo31",1.37924433999339e-11,0.264140031140483,0.631,0.435,2.32568180
609685e-07,"1","Tnpo3"
"Abca91",1.60982789881791e-11,0.279899283750213,0.62,0.404,2.714491802
98677e-07,"1","Abca9"
"Ctsf1",1.99774537825675e-11,0.273997773918581,0.839,0.629,3.368598256
81653e-07,"1","Ctsf"
"Rel1",2.04243120335155e-11,0.31875736420055,0.888,0.68,3.443947495091
38e-07,"1","Rel"
"Capn3",2.16108721450132e-11,0.260031638496893,0.51,0.322,3.6440252610
9213e-07,"1","Capn3"
"Rpl27",2.32799847239169e-11,0.321669938891192,0.818,0.65,3.9254710241
4687e-07,"1","Rpl27"
"Cks2",2.49712391321563e-11,0.306643096687241,0.476,0.293,4.2106503424
6419e-07,"1","Cks2"
"Lair11",2.72286851090763e-11,0.272344784828264,0.963,0.782,4.59130088
309244e-07,"1","Lair1"
"Mylip1",3.16309858781242e-11,0.284550014174775,0.833,0.606,5.33361683
87693e-07,"1","Mylip"
"Pbxip1",3.23016962093401e-11,0.307546566311132,0.697,0.505,5.44671201
481893e-07,"1","Pbxip1"
"Slc35b2",3.23983707571288e-11,0.310805253005579,0.729,0.532,5.4630132
7706705e-07,"1","Slc35b2"
"Pdgfb1",3.60112348123564e-11,0.28431261701379,0.718,0.499,6.072214414
05954e-07,"1","Pdgfb"
"Sft2d11",5.03430351121469e-11,0.271290459228941,0.922,0.738,8.4888425
8061021e-07,"1","Sft2d1"
"Wsb11",5.23549026044976e-11,0.253633435712402,0.859,0.645,8.828083677
17039e-07,"1","Wsb1"
"Qk1",5.35466837353148e-11,0.290539579413298,0.965,0.812,9.02904181144
878e-07,"1","Qk"

"Nuak11",5.39412277388445e-11,0.254226904359313,0.455,0.278,9.09556982
132396e-07,"1","Nuak1"
"Cflar",5.41574590470611e-11,0.322374891147477,0.833,0.628,9.132030744
51544e-07,"1","Cflar"
"Man1a21",5.56134096613102e-11,0.267995844956365,0.746,0.537,9.3775331
3709012e-07,"1","Man1a2"
"Acp21",5.9317568017291e-11,0.270846261294159,0.683,0.488,1.0002128319
0756e-06,"1","Acp2"
"Tmem173",6.32609050956422e-11,0.267974677274954,0.689,0.49,1.06670538
172272e-06,"1","Tmem173"
"Camk2n1",1.19104569710263e-10,0.271307283044241,0.516,0.342,2.0083412
5445445e-06,"1","Camk2n1"
"Il1b",1.22720595324999e-10,0.503340094942486,0.718,0.533,2.0693146783
7013e-06,"1","Il1b"
"Col27a11",1.27453800488936e-10,0.303464370442389,0.513,0.329,2.149125
98384444e-06,"1","Col27a1"
"Tagap",1.82918394410452e-10,0.296648337162135,0.68,0.474,3.0843699665
4905e-06,"1","Tagap"
"Tspan33",1.87422551394937e-10,0.310850709232512,0.424,0.264,3.1603190
6162143e-06,"1","Tspan33"
"Timp2",2.94622033759013e-10,0.263493791001014,0.957,0.795,4.967916733
24447e-06,"1","Timp2"
"Pik3r1",3.08178000958734e-10,0.294862555907278,0.746,0.547,5.19649745
216617e-06,"1","Pik3r1"
"Tacc1",4.87260960465907e-10,0.283629928301903,0.821,0.63,8.2161943153
7613e-06,"1","Tacc1"
"Rasgrp31",5.87821738330425e-10,0.273927945461887,0.651,0.467,9.911850
15172763e-06,"1","Rasgrp3"
"Map3k81",6.1758766434094e-10,0.308110607080156,0.663,0.482,1.04137631
961169e-05,"1","Map3k8"
"Cd371",8.03215458111655e-10,0.258880556538436,0.888,0.746,1.354381905
46787e-05,"1","Cd37"
"Ccl3",9.31405749598106e-10,0.332522412181323,0.945,0.761,1.5705363749
7233e-05,"1","Ccl3"
"Rpl36al",1.02010863202599e-09,0.250775330318884,0.712,0.552,1.7201071
7532222e-05,"1","Rpl36al"
"Gpr341",1.21731036758356e-09,0.25009244536862,0.974,0.787,2.052628741
81941e-05,"1","Gpr34"
"Mafb1",1.56432888215679e-09,0.371592143535969,0.934,0.766,2.637771361
09278e-05,"1","Mafb"
"Pak2",1.60405621331816e-09,0.252591239685818,0.787,0.645,2.7047595868
9708e-05,"1","Pak2"
"Rnase4",1.78950770130526e-09,0.257674894395825,0.916,0.735,3.01746788
594093e-05,"1","Rnase4"
"Maff",1.8141030816006e-09,0.280888016970325,0.68,0.469,3.058940616194
93e-05,"1","Maff"
"Uqcrh",2.67994126714609e-09,0.264904489105783,0.824,0.697,4.518916964
66174e-05,"1","Uqcrh"
"Olfml31",2.77892606051514e-09,0.284727267216512,0.988,0.776,4.6858251
2324063e-05,"1","Olfml3"

"Lhfpl2",2.8605063529799e-09,0.260477672480556,0.539,0.375,4.82338581239471e-05,"1","Lhfpl2"
"AU020206",4.25300463727828e-09,0.295653603396368,0.553,0.392,7.17141641937864e-05,"1","AU020206"
"Rpl31",4.36995644343177e-09,0.267673888803642,0.79,0.665,7.36862055491465e-05,"1","Rpl31"
"Camk1d",5.05005523986973e-09,0.264147802394148,0.643,0.474,8.51540314546835e-05,"1","Camk1d"
"Gramd1a",6.07526773033371e-09,0.252335363901011,0.591,0.436,0.000102441164468887,"1","Gramd1a"
"Sall31",9.32374624587669e-09,0.300523810492482,0.476,0.318,0.000157217009197973,"1","Sall3"
"Nav21",9.87826346678912e-09,0.294562705992486,0.389,0.238,0.000166567278576998,"1","Nav2"
"Tmem881",1.53683867665453e-08,0.286204842296396,0.573,0.404,0.000259141737657487,"1","Tmem88"
"Mthfsl",1.54841260294894e-08,0.3259102851047,0.441,0.299,0.00026109333310925,"1","Mthfsl"
"Phf141",1.73830236462851e-08,0.269462976036844,0.62,0.456,0.000293112544723659,"1","Phf14"
"Pcf111",2.29374605709025e-08,0.258795161347797,0.703,0.54,0.000386771460146558,"1","Pcf11"
"AC160336.1",2.50951225140527e-08,0.495179807473108,0.501,0.364,0.000423153955831956,"1","AC160336.1"
"Swap70",2.58758427809924e-08,0.281285449504982,0.559,0.404,0.000436318460973094,"1","Swap70"
"Cdk121",3.35675018675581e-08,0.298744526667407,0.573,0.424,0.000566015216490765,"1","Cdk12"
"Gm20186",4.17604917511538e-08,0.390286943052126,0.3,0.173,0.000704165411907955,"1","Gm20186"
"Mycbp2",1.45889160369119e-07,0.252638422643706,0.718,0.573,0.00245998302214409,"1","Mycbp2"
"Chka1",4.65845068631687e-07,0.396056559823282,0.461,0.323,0.00785507954726751,"1","Chka"
"Sec24a",1.60227608597406e-05,0.280510732785851,0.366,0.268,0.270175793616946,"1","Sec24a"
"Adamts1",7.16627956624943e-05,0.291887258909815,0.256,0.167,1,"1","Adamts1"
"Phlda1",0.00283934671968014,0.332134476556823,0.441,0.364,1,"1","Phlda1"
"Rcan1",2.39376797928054e-82,2.09482689417885,0.972,0.469,4.03637156666284e-78,"2","Rcan1"
"Nr4a3",1.10186314003393e-70,1.49717046135594,0.762,0.225,1.8579616267252e-66,"2","Nr4a3"
"Egr2",3.5705158460793e-69,1.48149640207329,0.953,0.497,6.02060381965891e-65,"2","Egr2"
"Egr31",2.71338966684212e-59,1.77621712756011,0.808,0.339,4.57531765622919e-55,"2","Egr3"
"Tagap1",1.13857363725586e-51,1.1906118740961,0.893,0.463,1.91986286714083e-47,"2","Tagap"

"Tnf1",3.31460974860731e-51,1.42352452951594,0.977,0.675,5.58909495810164e-47,"2","Tnf"
"Edn1",1.22983473178583e-50,0.96850358752705,0.369,0.057,2.07374732473727e-46,"2","Edn1"
"Ier31",8.34598704416096e-48,1.32386059478909,0.991,0.783,1.40730033538642e-43,"2","Ier3"
"Ccl41",5.1416777879501e-47,1.1768305584011,1,0.768,8.66989708604146e-43,"2","Ccl4"
"Ccl2",5.11526281282283e-45,1.13634549340791,0.958,0.584,8.62535615498185e-41,"2","Ccl2"
"Ccl31",3.14109683806856e-43,1.1371374938615,0.995,0.769,5.2965174883512e-39,"2","Ccl3"
"Ccrl21",2.41596181913929e-42,1.42662660583123,0.953,0.647,4.07379481943267e-38,"2","Ccrl2"
"Nfkbid",6.45515741807627e-41,0.883830963651898,0.986,0.667,1.08846864383602e-36,"2","Nfkbid"
"Prdm1",1.46045299012641e-39,1.0058076843955,0.57,0.194,2.46261583195115e-35,"2","Prdm1"
"Gadd45b1",4.34342833482195e-36,1.02585960753903,0.977,0.641,7.32388885817678e-32,"2","Gadd45b"
"Gm26522",2.24972611815972e-35,1.19929667219587,0.584,0.22,3.79348818044091e-31,"2","Gm26522"
"Plek",8.85598991522294e-35,0.808743746759048,1,0.822,1.49329701950489e-30,"2","Plek"
"Pik3r11",1.9805235302114e-34,1.00370869886555,0.883,0.546,3.33955877664246e-30,"2","Pik3r1"
"Il1a2",7.07898996275343e-34,0.928785804869805,0.995,0.733,1.19365928751948e-29,"2","Il1a"
"Cd831",1.15877887453819e-33,0.794975269876804,1,0.818,1.9539329382463e-29,"2","Cd83"
"Rasgef1b",1.23927020331756e-33,0.873677036537357,0.757,0.382,2.08965741683408e-29,"2","Rasgef1b"
"Nr4a1",7.80680867715435e-32,0.82987419574068,0.93,0.556,1.31638407914177e-27,"2","Nr4a1"
"Sparc2",5.4576441101713e-30,0.681714948456699,1,0.822,9.20267949857084e-26,"2","Sparc"
"Ppp1r15a2",3.41357625619798e-28,0.876244774947375,0.977,0.727,5.75597228320103e-24,"2","Ppp1r15a"
"Bcl2a1b1",2.3654761121207e-26,0.737768941118544,0.995,0.847,3.98866582025792e-22,"2","Bcl2a1b"
"Dennd4a2",3.76712443179334e-25,0.784130778537449,0.986,0.718,6.35212521688992e-21,"2","Dennd4a"
"Ccl12",5.85159700832713e-25,0.761109827145677,0.925,0.664,9.86696287544121e-21,"2","Ccl12"
"Bcl2a1a1",1.06721942146633e-24,0.792216092893639,0.701,0.358,1.79954538847653e-20,"2","Bcl2a1a"
"Maff1",1.28747212028081e-24,0.799934424259611,0.808,0.47,2.17093548921749e-20,"2","Maff"
"Cst31",1.88132511586075e-24,0.546579790305518,1,0.992,3.1722904103644e-20,"2","Cst3"

"Cd86",4.98651838232288e-24,0.65665881781345,0.953,0.686,8.40826729627
283e-20,"2","Cd86"
"Gpr841",3.09979470717388e-22,0.672856323936003,0.991,0.704,5.22687383
523659e-18,"2","Gpr84"
"Marcks2",3.21620743726782e-22,0.485478434807096,0.995,0.913,5.4231689
80721e-18,"2","Marcks"
"Tgif12",7.50429766903291e-22,0.588317606105265,0.958,0.686,1.26537467
295233e-17,"2","Tgif1"
"Tanc22",1.31668371673153e-21,0.551792484031981,0.916,0.58,2.220192083
1527e-17,"2","Tanc2"
"Epb41l22",3.92499409599934e-21,0.577908596796042,0.977,0.754,6.618325
04467408e-17,"2","Epb41l2"
"Abl2",5.64858225088841e-21,0.589496800273631,0.589,0.298,9.5246393914
4804e-17,"2","Abl2"
"Smad71",6.80927654201324e-21,0.667426682534991,0.776,0.482,1.14818021
051427e-16,"2","Smad7"
"Picalm",3.78684490733683e-20,0.609495579644853,0.963,0.787,6.38537788
275136e-16,"2","Picalm"
"Kctd122",4.23793640598024e-20,0.575238567450807,0.981,0.824,7.1460083
6776388e-16,"2","Kctd12"
"Srgn",1.06033037574152e-19,0.535434058340626,0.991,0.838,1.7879290795
7535e-15,"2","Srgn"
"Tnfaip21",1.98376949219142e-19,1.00450381275103,0.65,0.368,3.34503211
773316e-15,"2","Tnfaip2"
"Nfkbia1",2.60494884533827e-19,0.580268990423835,0.995,0.841,4.3924647
4300939e-15,"2","Nfkbia"
"P2ry122",2.80731431432257e-19,0.578811037098226,0.981,0.785,4.7336933
9681071e-15,"2","P2ry12"
"Coq10b",4.69308875745201e-19,0.747341922544625,0.822,0.549,7.91348626
281559e-15,"2","Coq10b"
"Mylip2",4.4033074912875e-18,0.63811635446245,0.864,0.62,7.42485709180
898e-14,"2","Mylip"
"Arl5c1",5.36764347838736e-18,0.733467248027598,0.818,0.521,9.05092043
325677e-14,"2","Arl5c"
"Tsc22d22",5.64276571306224e-18,0.648205164161907,0.86,0.587,9.5148315
4536554e-14,"2","Tsc22d2"
"Frmd4a2",6.52729200726463e-18,0.480246879858747,0.874,0.581,1.1006319
7826496e-13,"2","Frmd4a"
"Tgfbr12",6.55151123327368e-18,0.564397526937975,0.972,0.724,1.1047158
2415461e-13,"2","Tgfbr1"
"Cyfip11",7.10803945252664e-18,0.442391150675575,0.986,0.862,1.1985576
1248504e-13,"2","Cyfip1"
"Dst2",1.94167429415972e-17,0.462758944021115,0.855,0.562,3.2740511948
1212e-13,"2","Dst"
"Ncf11",4.44612386989626e-17,0.565342432782082,0.967,0.772,7.497054069
41908e-13,"2","Ncf1"
"Plk31",5.47072938032617e-17,0.530569104540946,0.832,0.552,9.224743881
10599e-13,"2","Plk3"
"Spry1",8.01125280832676e-17,0.439202621550667,0.29,0.096,1.3508574485
4006e-12,"2","Spry1"

"Pdgfb2",1.10792799949297e-16,0.69658547746166,0.776,0.509,1.868188192
74504e-12,"2","Pdgfb"
"Ahcyl2",1.28045141867481e-16,0.601969881391772,0.411,0.188,2.15909718
216946e-12,"2","Ahcyl2"
"Cd180",1.58128709431987e-16,0.720700129425814,0.864,0.634,2.666366298
44216e-12,"2","Cd180"
"Tnfaip31",2.27760534181547e-16,0.631878461799908,0.953,0.687,3.840498
12736925e-12,"2","Tnfaip3"
"Mgat4a1",2.30687182345502e-16,0.50160943209909,0.785,0.503,3.88984726
870985e-12,"2","Mgat4a"
"Tra2a1",3.72693916856844e-16,0.48191386016749,0.85,0.575,6.2843648260
401e-12,"2","Tra2a"
"Trim8",4.85385049079492e-16,0.429337986480147,0.804,0.53,8.1845626975
784e-12,"2","Trim8"
"Tal1",1.21391316380199e-15,0.443958175340042,0.421,0.196,2.0469003768
0292e-11,"2","Tal1"
"Tmem1731",1.43384119835827e-15,0.446784825222308,0.771,0.495,2.417743
02867171e-11,"2","Tmem173"
"Arhgap52",1.70564348863826e-15,0.390174018128066,0.967,0.667,2.876056
05054183e-11,"2","Arhgap5"
"Nav32",2.71800450377522e-15,0.470481416295929,0.855,0.532,4.583099194
26578e-11,"2","Nav3"
"Mob3c2",3.61426047370723e-15,0.494720417518903,0.715,0.446,6.09436601
076514e-11,"2","Mob3c"
"Csf1r2",8.45627954901223e-15,0.349259845490296,1,0.939,1.425897857554
44e-10,"2","Csf1r"
"Sall12",1.34445121784866e-14,0.401594784510112,0.734,0.456,2.26701364
35364e-10,"2","Sall1"
"Golm12",1.65053960972464e-14,0.395712611019405,0.902,0.645,2.78313988
991769e-10,"2","Golm1"
"Il1b1",1.72708091897555e-14,0.838831974463022,0.776,0.54,2.9122038455
7658e-10,"2","Il1b"
"Gm31410",1.84125595573797e-14,0.324007448948954,0.252,0.086,3.1047257
9256537e-10,"2","Gm31410"
"Cx3cr12",2.07052855498706e-14,0.448464513751349,0.991,0.841,3.4913252
4941919e-10,"2","Cx3cr1"
"Ly861",3.61572881964784e-14,0.407213084150627,1,0.95,6.09684193569019
e-10,"2","Ly86"
"Nfkbiz2",3.7070685309405e-14,0.480480693093122,0.991,0.759,6.25085895
687187e-10,"2","Nfkbiz"
"Runx1",4.15942128692863e-14,0.472713791178922,0.911,0.658,7.013616174
01905e-10,"2","Runx1"
"Csf1",5.83815875748488e-14,0.744409990298099,0.407,0.191,9.8443032968
71e-10,"2","Csf1"
"Qk2",6.27928521682748e-14,0.458600634924334,0.991,0.821,1.05881307326
145e-09,"2","Qk"
"Srgap22",7.3305135022661e-14,0.420182701886323,0.921,0.654,1.23607118
675211e-09,"2","Srgap2"
"Clic4",9.24598804447193e-14,0.615558331453588,0.72,0.455,1.5590585040
5886e-09,"2","Clic4"

"Ubl3",1.06328631232851e-13,0.463209065160414,0.888,0.672,1.7929133798
4834e-09,"2","Ubl3"
"Bcl2a1d1",1.21730812345962e-13,0.468076017892148,0.626,0.371,2.052624
95777762e-09,"2","Bcl2a1d"
"Git2",1.24115187919644e-13,0.667265549499152,0.752,0.546,2.0928302987
0103e-09,"2","Git2"
"Capn31",1.28765336859859e-13,0.380927961501007,0.584,0.328,2.17124111
013095e-09,"2","Capn3"
"Chd7",1.38413880491527e-13,0.517499521749716,0.808,0.583,2.3339348528
4813e-09,"2","Chd7"
"Rel2",1.42023404496196e-13,0.437666103327072,0.939,0.69,2.39479864661
485e-09,"2","Rel"
"Ctss1",2.09567765376829e-13,0.304872289142406,1,0.981,3.5337316597840
9e-09,"2","Ctss"
"Mef2a2",2.35864548943743e-13,0.445072574568261,0.958,0.753,3.97714802
42894e-09,"2","Mef2a"
"Plxdc22",2.63653656339144e-13,0.395897920121988,0.967,0.753,4.4457279
5319064e-09,"2","Plxdc2"
"Zfp362",2.67765126159752e-13,0.447978878204315,1,0.826,4.515055557305
74e-09,"2","Zfp36"
"Itgam2",2.8764407166301e-13,0.378492807534872,0.986,0.785,4.850254336
38167e-09,"2","Itgam"
"Ecscr2",4.84444830789571e-13,0.394510883908543,0.836,0.581,8.16870873
677375e-09,"2","Ecscr"
"Mthfsl1",6.6864258340772e-13,0.451238907004681,0.533,0.299,1.12746512
41421e-08,"2","Mthfsl"
"Rhoh1",6.83401113420007e-13,0.434014307207937,0.748,0.505,1.152350957
44882e-08,"2","Rhoh"
"Rrbp12",8.33026550323568e-13,0.441033006946958,0.977,0.841,1.40464936
91556e-08,"2","Rrbp1"
"Pde3b2",8.56854331010071e-13,0.401710005186858,0.883,0.643,1.44482777
294918e-08,"2","Pde3b"
"Wipf1",8.66816448326946e-13,0.510487943422532,0.664,0.436,1.461625895
1689e-08,"2","Wipf1"
"Bach1",9.60535604103526e-13,0.489436452699818,0.785,0.555,1.619655135
63937e-08,"2","Bach1"
"Cd341",1.10598188523291e-12,0.357902914692866,0.827,0.549,1.864906654
87973e-08,"2","Cd34"
"Tmcc32",1.15436279237979e-12,0.509586746882867,0.827,0.591,1.94648654
05108e-08,"2","Tmcc3"
"Sall32",1.59643217875275e-12,0.373569671371383,0.561,0.319,2.69190393
981288e-08,"2","Sall3"
"Plekho21",2.52460896453937e-12,0.36930345419196,0.879,0.61,4.25699563
600628e-08,"2","Plekho2"
"Selplg2",2.69552889227787e-12,0.387349218471247,1,0.838,4.54520081815
895e-08,"2","Selplg"
"Rasgrp32",3.35010014020449e-12,0.339754496717585,0.72,0.473,5.6489388
5641282e-08,"2","Rasgrp3"
"Macf12",3.55793855245335e-12,0.433919876502653,0.907,0.703,5.99939598
714684e-08,"2","Macf1"

"Ccr51",3.65586573509795e-12,0.37222271631085,0.874,0.594,6.1645208025
2216e-08,"2","Ccr5"
"Itgb52",5.13276921991655e-12,0.363805347781871,1,0.853,8.654875458623
29e-08,"2","Itgb5"
"Zfp36l11",5.73223870598354e-12,0.479273575396157,0.921,0.704,9.665700
90602945e-08,"2","Zfp36l1"
"Pou2f21",6.87213174997764e-12,0.366984609283842,0.967,0.746,1.1587788
5568123e-07,"2","Pou2f2"
"Dock42",9.30257025682843e-12,0.322356506219891,0.808,0.549,1.56859939
670641e-07,"2","Dock4"
"Rab20",1.17962950249335e-11,0.472162440413005,0.724,0.491,1.989091267
10428e-07,"2","Rab20"
"Plcl2",1.21053469905173e-11,0.288453248100634,0.551,0.316,2.041203609
54102e-07,"2","Plcl2"
"Slc35b21",1.47946649637573e-11,0.403399830981586,0.776,0.542,2.494676
40618876e-07,"2","Slc35b2"
"Irf81",1.67618295868437e-11,0.542449863592598,0.958,0.819,2.826379704
93359e-07,"2","Irf8"
"Socs32",1.87619901489589e-11,0.416016513259218,0.981,0.749,3.16364677
891745e-07,"2","Socs3"
"Skil2",1.95856111906308e-11,0.409595320887825,0.981,0.777,3.302525758
96416e-07,"2","Skil"
"Dock101",1.99341041327242e-11,0.395875378225101,0.869,0.611,3.3612886
3885995e-07,"2","Dock10"
"Tmem1192",2.13477811424741e-11,0.349383413792687,0.949,0.69,3.5996628
5624398e-07,"2","Tmem119"
"Mtdh2",2.20678224542065e-11,0.380199623911951,0.93,0.808,3.7210762222
283e-07,"2","Mtdh"
"Lhfpl21",2.28524854918323e-11,0.385434940571473,0.621,0.378,3.8533861
0363275e-07,"2","Lhfpl2"
"Pag12",3.70578516916411e-11,0.3278904574037,0.734,0.49,6.248694952244
52e-07,"2","Pag1"
"Lag32",5.48062472702055e-11,0.303735330167509,0.902,0.681,9.241429414
70206e-07,"2","Lag3"
"Gramd1a1",6.15727945211984e-11,0.401085993960274,0.696,0.435,1.038240
46121645e-06,"2","Gramd1a"
"Lair12",7.00319046847256e-11,0.328899705192179,0.977,0.795,1.18087797
679384e-06,"2","Lair1"
"Zfhx32",7.50071592615705e-11,0.382766275260479,0.939,0.693,1.26477071
94686e-06,"2","Zfhx3"
"Herc21",8.16875520013087e-11,0.314640864138052,0.738,0.52,1.377415501
84607e-06,"2","Herc2"
"Numb1",8.63354764432537e-11,0.339555746472663,0.57,0.357,1.4557888037
8614e-06,"2","Numb"
"Smim3",1.09173442386427e-10,0.423567443736078,0.589,0.376,1.840882585
51993e-06,"2","Smim3"
"Slc29a31",1.13129237491638e-10,0.337111483563732,0.846,0.624,1.907585
202584e-06,"2","Slc29a3"
"Tob22",1.15857380782235e-10,0.429770919826216,0.776,0.537,1.953587154
75005e-06,"2","Tob2"

"Inpp5d1",1.16811828194809e-10,0.339614062419838,0.879,0.649,1.9696810 4702087e-06,"2","Inpp5d"
"Bmp2k2",1.21579179162054e-10,0.314123218586563,0.911,0.68,2.050068119 03055e-06,"2","Bmp2k"
"Mertk1",1.24585314943387e-10,0.372246160305323,0.827,0.573,2.10075758 057539e-06,"2","Mertk"
"Fam102b2",1.3206335367591e-10,0.387075895386547,0.752,0.516,2.2268522 6968319e-06,"2","Fam102b"
"Wasf21",1.36852878793869e-10,0.343173015160385,0.883,0.707,2.30761324 222221e-06,"2","Wasf2"
"Entpd11",1.39431665755622e-10,0.352290394742786,0.752,0.547,2.3510967 4797129e-06,"2","Entpd1"
"Btg11",1.52231323388527e-10,0.355608663771085,0.972,0.827,2.566924574 97735e-06,"2","Btg1"
"Slc38a1",1.60492071177719e-10,0.471386804969695,0.65,0.435,2.70621730 41987e-06,"2","Slc38a1"
"Nrip11",1.76584587289946e-10,0.35081579494034,0.832,0.572,2.977569310 88308e-06,"2","Nrip1"
"Hivep3",1.90723405640988e-10,0.288371392635331,0.654,0.422,3.21597806 591834e-06,"2","Hivep3"
"Adgrg12",2.13929384139474e-10,0.316118546584397,0.785,0.539,3.6072772 7535981e-06,"2","Adgrg1"
"Cd331",2.50111827184435e-10,0.424212036877019,0.846,0.645,4.217385629 98394e-06,"2","Cd33"
"Wnk12",2.73123093589024e-10,0.351295465901567,0.986,0.785,4.605401604 09813e-06,"2","Wnk1"
"Icam12",2.77833182279914e-10,0.34964958126604,0.958,0.717,4.684823119 6039e-06,"2","Icam1"
"Ldhb1",3.25913157275682e-10,0.326824746388469,0.967,0.792,5.495547657 98254e-06,"2","Ldhb"
"Col27a12",3.56204422211187e-10,0.261360174275942,0.575,0.336,6.006318 96732503e-06,"2","Col27a1"
"Ubash3b2",3.8200436089649e-10,0.32123757805094,0.808,0.566,6.44135753 343661e-06,"2","Ubash3b"
"Fchsd2",4.02859181284269e-10,0.38147554160151,0.65,0.404,6.7930115148 1534e-06,"2","Fchsd2"
"Cflar1",4.83545480737697e-10,0.462064231296047,0.864,0.64,8.153543896 19905e-06,"2","Cflar"
"Aif1",5.10637578233316e-10,0.255188971929131,0.958,0.832,8.6103708441 7018e-06,"2","Aif1"
"Sec61g",5.29349637434950e-10,0.31116633680243,0.785,0.584,8.925893586 42812e-06,"2","Sec61g"
"Siglech2",6.16103781308443e-10,0.312171562696808,0.949,0.675,1.038874 1960423e-05,"2","Siglech"
"Rpl18a1",8.75355623969908e-10,0.27863504917371,0.995,0.947,1.47602465 313806e-05,"2","Rpl18a"
"Arhgap31",1.0981198231425e-09,0.408552079363793,0.481,0.302,1.8516496 4578288e-05,"2","Arhgap31"
"Msn",1.17310780320267e-09,0.263005737484651,0.972,0.832,1.97809437776 034e-05,"2","Msn"

"Hif1a",1.23040570826202e-09,0.32133521512575,0.682,0.476,2.0747101052
7141e-05,"2","Hif1a"
"Ski2",1.65318974606625e-09,0.321053483184903,0.836,0.623,2.7876085498
1691e-05,"2","Ski"
"Rnf21",1.72686215597724e-09,0.292319895022515,0.589,0.374,2.911834967
40883e-05,"2","Rnf2"
"Rpl211",1.9131994896855e-09,0.315300841121565,0.981,0.941,3.226036979
50769e-05,"2","Rpl21"
"Itpkb",2.07408012063867e-09,0.45296846778087,0.575,0.374,3.4973138994
2093e-05,"2","Itpkb"
"Rapgef51",2.11771827777676e-09,0.291255216031186,0.589,0.377,3.570896
55998718e-05,"2","Rapgef5"
"Egr12",2.25256935180626e-09,0.327319420567483,0.995,0.771,3.798282441
01572e-05,"2","Egr1"
"Dapp1",2.28126134261657e-09,0.459682984927557,0.556,0.382,3.846662875
92007e-05,"2","Dapp1"
"Map4k4",2.68550606163038e-09,0.286546317612218,0.804,0.571,4.52830032
112115e-05,"2","Map4k4"
"Nlrp32",4.01973539735704e-09,0.351848121568194,0.883,0.672,6.77807782
702344e-05,"2","Nlrp3"
"Orai2",4.10686254937635e-09,0.294410978996365,0.453,0.262,6.924991630
7584e-05,"2","Orai2"
"Fam217b",4.1138651226205e-09,0.34808937539504,0.439,0.25,6.9367993697
6269e-05,"2","Fam217b"
"Map3k82",4.9304962855108e-09,0.28303480578138,0.71,0.49,8.31380283662
831e-05,"2","Map3k8"
"Med21",5.2386851420868e-09,0.926783534719525,0.425,0.261,8.8334708865
8676e-05,"2","Med21"
"Cd811",5.35388445596191e-09,0.286583268808354,1,0.876,9.0277199696429
7e-05,"2","Cd81"
"Casp41",5.93573631408418e-09,0.334237028994199,0.79,0.568,0.000100088
385728087,"2","Casp4"
"Samsn1",6.02390650323100e-09,0.394511645199006,0.79,0.588,0.000101575
111457481,"2","Samsn1"
"Ldlrad4",6.33531043342176e-09,0.251148712210273,0.467,0.275,0.0001068
26004528358,"2","Ldlrad4"
"Zbtb1",8.81309273448662e-09,0.421364471727651,0.486,0.313,0.000148606
369688913,"2","Zbtb1"
"Helz",9.12630713408482e-09,0.31744241925318,0.467,0.285,0.00015388779
0894938,"2","Helz"
"Spata13",9.25503554794955e-09,0.303649793912213,0.495,0.307,0.0001560
58409409525,"2","Spata13"
"Rps121",1.24132473230811e-08,0.307991375597656,0.995,0.944,0.00020931
2176361794,"2","Rps12"
"Itgav",1.24136359301936e-08,0.292334452337432,0.621,0.412,0.000209318
729054925,"2","Itgav"
"Rplp11",1.29016741173261e-08,0.324553466827606,1,0.952,0.000217548028
966353,"2","Rplp1"
"Gcnt2",1.30613145642743e-08,0.290282444158122,0.421,0.24,0.0002202398
86182794,"2","Gcnt2"

"Spag91",1.3487433779686e-08,0.287277772757636,0.86,0.645,0.0002274251
08393065,"2","Spag9"
"M6pr",1.3776715056074e-08,0.287968461369868,0.813,0.619,0.00023230296
927552,"2","M6pr"
"Dip2b1",1.38347335359647e-08,0.252690501991952,0.612,0.436,0.00023328
1276883438,"2","Dip2b"
"Gpr155",1.44027012708258e-08,0.266277393590461,0.481,0.287,0.00024285
8348828665,"2","Gpr155"
"Cadm1",1.62286943532375e-08,0.300703158230258,0.72,0.511,0.0002736482
44184291,"2","Cadm1"
"Tifa",1.63656617894977e-08,0.357447188916097,0.687,0.501,0.0002759577
8909451,"2","Tifa"
"Rtn4rl11",1.6995441080978e-08,0.252317399618856,0.584,0.371,0.0002865
77127507451,"2","Rtn4rl1"
"Ell2",1.72523846830444e-08,0.358847450386133,0.551,0.362,0.0002909097
10525495,"2","Ell2"
"Rps201",2.06850579402437e-08,0.277344116245793,0.977,0.919,0.00034879
1446988389,"2","Rps20"
"Rps291",2.38177176926252e-08,0.320474873762949,0.986,0.948,0.00040161
4355733046,"2","Rps29"
"Nfkbie",2.79619951160096e-08,0.326804489439176,0.5,0.323,0.0004714951
61646155,"2","Nfkbie"
"Mrpl521",2.89669800917619e-08,0.287704863155701,0.678,0.485,0.0004884
41218307289,"2","Mrpl52"
"Marcksl12",3.03866207565584e-08,0.312891845066632,0.93,0.712,0.000512
379199197088,"2","Marcksl1"
"Serinc31",3.10488564096998e-08,0.254688440777618,0.991,0.926,0.000523
545816780359,"2","Serinc3"
"Ltc4s",3.38996247389111e-08,0.364197622104281,0.836,0.663,0.000571615
472347519,"2","Ltc4s"
"Rpl37a1",3.4133742871239e-08,0.348837670726185,0.972,0.892,0.00057556
3172294831,"2","Rpl37a"
"Abca92",3.42353639596148e-08,0.299983274694913,0.626,0.42,0.000577276
707087024,"2","Abca9"
"Tnfsf9",3.56687667060442e-08,0.535202261048674,0.262,0.122,0.00060144
6744197317,"2","Tnfsf9"
"Nsa2",3.74504395459137e-08,0.328730250670136,0.841,0.697,0.0006314893
11623197,"2","Nsa2"
"Tulp4",3.87919899048821e-08,0.356037810827022,0.57,0.398,0.0006541105
33776121,"2","Tulp4"
"Rps181",4.77768413050762e-08,0.255458835526627,0.995,0.911,0.00080561
3098086195,"2","Rps18"
"Rps211",5.17273450468181e-08,0.350457495353555,0.963,0.885,0.00087222
6492179447,"2","Rps21"
"Dock8",5.2200841774659e-08,0.272539031930246,0.678,0.476,0.0008802105
940043,"2","Dock8"
"Lpar6",5.64675936623365e-08,0.284389429789605,0.467,0.29,0.0009521565
64334317,"2","Lpar6"
"Nuak2",5.90426717993529e-08,0.299753979676237,0.561,0.378,0.000995577
531880689,"2","Nuak2"

"Ctsc",6.23390517342812e-08,0.281502560210044,0.986,0.877,0.0010511610
9034345,"2","Ctsc"
"Malat11",6.71045167491439e-08,0.287363933850521,0.995,0.807,0.0011315
1636142406,"2","Malat1"
"Arf2",7.0466181311442e-08,0.265324321089006,0.542,0.357,0.00118820074
927354,"2","Arf2"
"Serpine22",7.17342389409434e-08,0.252636262919495,0.958,0.717,0.00120
958273702219,"2","Serpine2"
"Zfp6221",7.3369272826687e-08,0.347630404976326,0.748,0.546,0.00123715
26784036,"2","Zfp622"
"Rpl27a1",7.35524126950876e-08,0.261176715288221,0.991,0.954,0.0012402
4078286457,"2","Rpl27a"
"Nfe2l2",8.20447933937884e-08,0.333842362799659,0.907,0.704,0.00138343
930620606,"2","Nfe2l2"
"Tmem135",8.73235335271091e-08,0.264497966594904,0.533,0.348,0.0014724
4942233411,"2","Tmem135"
"Rps15a1",9.30447394723063e-08,0.311819989441271,0.986,0.918,0.0015689
2039698203,"2","Rps15a"
"Rpl35a1",9.81418814765948e-08,0.314415615926599,0.995,0.915,0.0016548
6840545834,"2","Rpl35a"
"Prex1",1.0019867257207e-07,0.332490295220654,0.682,0.504,0.0016895500
1691025,"2","Prex1"
"Rpl221",1.02147915780995e-07,0.276014008568754,0.944,0.83,0.001722418
15589915,"2","Rpl22"
"Cxcl10",1.04066910231978e-07,0.774590585665368,0.519,0.346,0.00175477
624033161,"2","Cxcl10"
"Rps151",1.04504229995326e-07,0.292445020582027,0.949,0.847,0.00176215
032618119,"2","Rps15"
"Rpl36a1",1.06891864117529e-07,0.33636814622086,0.874,0.75,0.001802410
61274978,"2","Rpl36a"
"Dclre1c",1.13442407647006e-07,0.253880751718959,0.411,0.248,0.0019128
6587774382,"2","Dclre1c"
"Rcsd11",1.22362829293298e-07,0.40488239839557,0.621,0.448,0.002063282
02754358,"2","Rcsd1"
"Smap2",1.26546858855539e-07,0.252913713224594,0.907,0.781,0.002133833
13402209,"2","Smap2"
"Zfp292",1.28689953009026e-07,0.273272781912142,0.579,0.389,0.00216996
99876382,"2","Zfp292"
"Slc2a51",1.31018678059349e-07,0.273980017114212,0.495,0.315,0.0022092
3694943674,"2","Slc2a5"
"Stag1",1.34049510277136e-07,0.279660699917636,0.542,0.368,0.002260342
84229306,"2","Stag1"
"Fau1",1.35121848298578e-07,0.331347328600025,1,0.966,0.00227842460601
063,"2","Fau"
"Slfn2",1.37841092178437e-07,0.4169698348016,0.841,0.675,0.00232427649
631281,"2","Slfn2"
"Rpl371",1.42993973599277e-07,0.317747961123345,0.977,0.876,0.00241116
4382831,"2","Rpl37"
"C5ar2",1.69270865550551e-07,0.345358755111268,0.407,0.248,0.002854245
3349134,"2","C5ar2"

"Ptgs2",1.76668392111007e-07,0.706290314989333,0.271,0.141,0.0029789824277758,"2","Ptgs2"
"Ophn11",1.76769837004731e-07,0.261186076845533,0.706,0.5,0.00298069299157377,"2","Ophn1"
"Rilpl2",1.86373290168694e-07,0.250045186068659,0.696,0.471,0.00314262641882452,"2","Rilpl2"
"Cited2",1.92038590515876e-07,0.264159467775127,0.818,0.613,0.00323815471327869,"2","Cited2"
"Mapk6",1.94341328472243e-07,0.300184989246217,0.537,0.363,0.00327698348069895,"2","Mapk6"
"Sema4d",1.96858355170929e-07,0.299928435938087,0.706,0.521,0.0033194255848922,"2","Sema4d"
"Adap2os1",1.99965064472848e-07,0.254073206237327,0.495,0.315,0.00337181091714116,"2","Adap2os"
"Phf142",2.18192391679426e-07,0.272067002114176,0.65,0.465,0.00367916010849848,"2","Phf14"
"Rpl391",2.2209096800948e-07,0.283069765563257,0.967,0.907,0.00374489790257585,"2","Rpl39"
"Maml31",2.47196399887046e-07,0.261382459767405,0.453,0.279,0.00416822569489537,"2","Maml3"
"Glrx",2.71065128083006e-07,0.349441702926799,0.565,0.382,0.00457070018973565,"2","Glrx"
"Xist1",2.80987078659787e-07,0.657595780734762,0.435,0.276,0.00473800412036134,"2","Xist"
"Tjp1",2.81682718661371e-07,0.26716203425907,0.425,0.26,0.00474973400206804,"2","Tjp1"
"Slc1a3",2.86544937070107e-07,0.306208139534109,0.28,0.145,0.00483172072887614,"2","Slc1a3"
"Cep170",3.15633053941817e-07,0.292377419702509,0.523,0.362,0.00532220455556691,"2","Cep170"
"Rplp21",3.52059986740243e-07,0.296853780943319,0.972,0.857,0.00593643549641397,"2","Rplp2"
"Gripap11",3.84112178027259e-07,0.253283724532121,0.519,0.339,0.00647689954589564,"2","Gripap1"
"Rpl261",3.88148357287635e-07,0.263939270101347,1,0.929,0.0065449576005841,"2","Rpl26"
"Elmo11",4.00849314089896e-07,0.256162762084319,0.79,0.601,0.00675912113418383,"2","Elmo1"
"Gch1",4.04266426529065e-07,0.285837592393995,0.486,0.32,0.0068167404841331,"2","Gch1"
"Rps251",4.28210331516755e-07,0.28037628845535,0.949,0.853,0.00722048261003553,"2","Rps25"
"Zeb21",4.53894821164783e-07,0.301409447738147,0.953,0.774,0.00765357447448058,"2","Zeb2"
"Ppp6r1",4.72566214412438e-07,0.261525091457281,0.528,0.352,0.00796841150742253,"2","Ppp6r1"
"Zcchc61",5.30426470820361e-07,0.325065914346895,0.846,0.645,0.00894405115097293,"2","Zcchc6"
"B4galt11",5.32800969036773e-07,0.261587143097178,0.551,0.36,0.00898408993989806,"2","B4galt1"

"Mef2c1",5.89175717856189e-07,0.291250836036805,0.944,0.801,0.00993468095449107,"2","Mef2c"
"Rps261",7.15924764441954e-07,0.306135503760863,0.925,0.853,0.0120719233780202,"2","Rps26"
"N4bp1",8.64488974227591e-07,0.347082737809671,0.636,0.486,0.0145770130834256,"2","N4bp1"
"Lacc1",9.70035237611316e-07,0.257072138191617,0.757,0.572,0.016356734176602,"2","Lacc1"
"Fscn12",1.09200531893549e-06,0.271113111750137,0.678,0.497,0.0184133936878902,"2","Fscn1"
"Tlr13",1.14339510526346e-06,0.333912766223987,0.439,0.294,0.0192799282649524,"2","Tlr13"
"Nfat51",1.14933973102303e-06,0.28422163396651,0.617,0.452,0.0193801665445103,"2","Nfat5"
"Bod1l",1.17800294117172e-06,0.295917962587271,0.65,0.48,0.0198634855940376,"2","Bod1l"
"Rps281",1.22507020635281e-06,0.313880184482376,0.897,0.797,0.0206571338195212,"2","Rps28"
"Fyb",1.34953907506423e-06,0.261108183020408,0.986,0.833,0.022755927883733,"2","Fyb"
"Csrnp12",1.47210205676076e-06,0.26401638829124,0.902,0.662,0.0248225848810999,"2","Csrnp1"
"Arsb1",1.50506899399743e-06,0.288652537113816,0.743,0.538,0.0253784733767846,"2","Arsb"
"Rpl361",1.79739402298143e-06,0.253962991073094,0.939,0.821,0.0303076580155129,"2","Rpl36"
"Cd841",1.86453525147339e-06,0.313281701363912,0.818,0.646,0.0314397934103444,"2","Cd84"
"Rps271",2.98894891733894e-06,0.277732123377995,0.967,0.864,0.0503996566441691,"2","Rps27"
"Sdc41",3.08060266715407e-06,0.386343835655953,0.57,0.389,0.0519451221735519,"2","Sdc4"
"Rps231",3.27017745356898e-06,0.253310892853641,0.977,0.928,0.0551417322220801,"2","Rps23"
"Rgs1",3.33728109949805e-06,0.394745609996837,0.486,0.328,0.0562732338997361,"2","Rgs1"
"Trim35",5.05090230801108e-06,1.0128128585368,0.393,0.271,0.0851683147176829,"2","Trim35"
"Rpl301",5.23141138980564e-06,0.258872260888102,0.977,0.914,0.0882120588549027,"2","Rpl30"
"Sgk11",5.51084115019973e-06,0.299228950542026,0.706,0.541,0.0929238034746679,"2","Sgk1"
"Erbin",5.66263576224213e-06,0.261641810148956,0.664,0.498,0.0954833642229268,"2","Erbin"
"Cpeb4",5.99912298518492e-06,0.271643000027022,0.5,0.356,0.101157211776188,"2","Cpeb4"
"Olfml32",6.07930840188084e-06,0.269083588209373,0.972,0.795,0.102509298272515,"2","Olfml3"
"Tnfaip81",6.50887307354396e-06,0.295164192995983,0.855,0.686,0.109752617766098,"2","Tnfaip8"

"Kdm2b",7.34408655290637e-06,0.257098137077096,0.551,0.409,0.123835987455107,"2","Kdm2b"
"Pde4b",7.69517498917038e-06,0.291688105108401,0.505,0.354,0.129756040667391,"2","Pde4b"
"Osm",9.73157317725158e-06,0.365765451044117,0.579,0.439,0.164093786914816,"2","Osm"
"Dhx401",1.00057064205279e-05,0.279929493020051,0.467,0.308,0.168716221662942,"2","Dhx40"
"Rpl341",1.20469333131113e-05,0.275233421984126,0.949,0.897,0.203135389525682,"2","Rpl34"
"Rpl411",1.23211310012914e-05,0.261671480596259,0.972,0.892,0.207758910943776,"2","Rpl41"
"Rpl351",1.27752275768475e-05,0.250523785212554,0.864,0.763,0.215415887400803,"2","Rpl35"
"Gm37233",2.01434031831308e-05,0.293193335084282,0.257,0.147,0.339658064473952,"2","Gm37233"
"Gxylt11",2.4381570371498e-05,0.262445017942921,0.453,0.314,0.4111220396042,"2","Gxylt1"
"Slc16a61",3.00320947332446e-05,0.251226713644267,0.715,0.56,0.50640118139197,"2","Slc16a6"
"AC160336.11",5.22402490213797e-05,0.68657813452774,0.505,0.375,0.880875078998505,"2","AC160336.1"
"Dot1l",5.83445972201809e-05,0.296645367363292,0.509,0.382,0.98380659832669,"2","Dot1l"
"Sox4",6.34167877888181e-05,0.296582158652933,0.416,0.296,1,"2","Sox4"
"5430427O19Rik",6.68924164223868e-05,0.29878795444127,0.36,0.247,1,"2","5430427O19Rik"
"Tle4",6.80027055349101e-05,0.253047319077048,0.453,0.325,1,"2","Tle4"
"Adamts11",0.000104992894576463,0.53810046059439,0.276,0.171,1,"2","Adamts1"
"Gm201861",0.000120847829766415,0.264013296151498,0.294,0.184,1,"2","Gm20186"
"Cd14",0.00068626948783679,0.262628775563182,0.953,0.826,1,"2","Cd14"
"Dusp21",0.000850890323755134,0.268018191008768,0.71,0.556,1,"2","Dusp2"
"Ccl9",0.0018220832306228,0.333508840710001,0.589,0.465,1,"2","Ccl9"
"Kcnq1ot11",0.00182873489336591,0.272835515642925,0.509,0.402,1,"2","Kcnq1ot1"
"Nupr1",0.00460393192933647,0.45128291245188,0.36,0.282,1,"2","Nupr1"
"Tmsb4x",3.37176670894173e-28,0.759698447395651,1,0.997,5.68547302461754e-24,"3","Tmsb4x"
"Rps2",8.63079024127632e-28,0.854742121309068,0.938,0.992,1.45532385048401e-23,"3","Rps2"
"Rplp0",2.14453008301171e-27,0.806574983135683,0.956,0.988,3.61610662597434e-23,"3","Rplp0"
"Rpl19",9.64734136822443e-21,0.660080941964772,0.963,0.985,1.62673470151e-16,"3","Rpl19"
"Gnas",1.33914702301554e-20,0.71449174778209,0.938,0.973,2.2580697102088e-16,"3","Gnas"
"Fth1",2.34167912039126e-20,0.787983468767909,0.981,0.995,3.9485393328

0374e-16,"3","Fth1"
"Gapdh",8.15814659173486e-20,1.54351927882762,0.9,0.961,1.37562667829833e-15,"3","Gapdh"
"Rpl3",8.00408242818659e-19,0.640756677479436,0.919,0.978,1.34964837904082e-14,"3","Rpl3"
"Rps4x",3.42846858236075e-18,0.692741010833655,0.938,0.988,5.7810837235767e-14,"3","Rps4x"
"Eef1a11",2.23425983477499e-17,0.559307471250523,0.956,0.996,3.76740893339759e-13,"3","Eef1a1"
"Rpl7",1.45709360922893e-16,0.715789748538089,0.938,0.975,2.45695124388182e-12,"3","Rpl7"
"Rps3a1",2.53289251126194e-16,0.639288929246444,0.956,0.986,4.27096335248989e-12,"3","Rps3a1"
"Rack1",1.18369852058031e-15,0.60290744306786,0.931,0.982,1.99595244540252e-11,"3","Rack1"
"Rpl13",1.27018847727889e-15,0.61842300518419,0.944,0.991,2.14179181038767e-11,"3","Rpl13"
"Rps3",1.27200135348293e-15,0.625664892609881,0.887,0.983,2.14484868224292e-11,"3","Rps3"
"Ppia",7.03383333173887e-15,0.627310703483144,0.906,0.974,1.18604497639781e-10,"3","Ppia"
"Rpl5",3.72473889249233e-14,0.690203161656479,0.875,0.97,6.28065472052057e-10,"3","Rpl5"
"Rpl61",2.34144070417362e-13,0.57893495497613,0.906,0.975,3.94813731537755e-09,"3","Rpl6"
"Rpsa",5.42169221477667e-13,0.594134363405979,0.925,0.992,9.14205741255642e-09,"3","Rpsa"
"Cfl1",7.00075059341713e-13,0.595983328419457,0.919,0.985,1.180466565062e-08,"3","Cfl1"
"Gm26917",1.66266497224872e-12,0.610385972158337,0.025,0.296,2.80358567620579e-08,"3","Gm26917"
"Rpl15",2.96138720677547e-12,0.544419750526381,0.906,0.97,4.99349110806479e-08,"3","Rpl15"
"Rpl14",3.77920242755755e-12,0.604948811524254,0.875,0.953,6.37249113334755e-08,"3","Rpl14"
"Rpl10a",3.95209329031081e-12,0.661411278810764,0.856,0.959,6.66401970612209e-08,"3","Rpl10a"
"Pkm",4.54512006314614e-12,1.48162005982841,0.781,0.861,7.66398145047703e-08,"3","Pkm"
"Pfn1",6.09910405703688e-12,0.64953606214361,0.869,0.954,1.02843092609756e-07,"3","Pfn1"
"Rpl8",9.64852766005715e-12,0.475525452412803,0.912,0.982,1.62693473403884e-07,"3","Rpl8"
"C1qb",5.67480363199055e-11,0.529557524347341,0.925,0.951,9.56885388426247e-07,"3","C1qb"
"Rpl7a",6.75060651896716e-11,0.555349868914726,0.869,0.957,1.13828727122824e-06,"3","Rpl7a"
"Itm2b",1.53157232445803e-10,0.557628542122894,0.925,0.996,2.58253725350113e-06,"3","Itm2b"
"Sh3bgrl3",3.4515634045449e-10,0.769886659820417,0.819,0.926,5.8200262

1274361e-06,"3","Sh3bgrl3"
"Rpl9",6.51709100926034e-10,0.499073690389996,0.894,0.971,1.0989118859
8148e-05,"3","Rpl9"
"Akr1a1",7.8732791241681e-10,0.730776387398887,0.781,0.869,1.327592325
91722e-05,"3","Akr1a1"
"Rbm3",2.8450791448543e-09,0.624671079714368,0.825,0.933,4.79737245405
332e-05,"3","Rbm3"
"Ldhb2",6.78027353624642e-09,0.763869423557972,0.762,0.816,0.000114328
972368187,"3","Ldhb"
"Capzb",1.01843349063408e-08,0.667680402566658,0.769,0.895,0.000171728
255190718,"3","Capzb"
"Arpc1b",1.05622925043107e-08,0.461823907295281,0.9,0.982,0.0001781013
76207687,"3","Arpc1b"
"Cdc42",2.365451443889e-08,0.477063968071065,0.875,0.966,0.00039886242
2468564,"3","Cdc42"
"Aldoa",4.21982948718024e-08,1.04455174423925,0.725,0.838,0.0007115476
48128332,"3","Aldoa"
"Slc25a3",9.7865671310152e-08,0.6374921708901,0.794,0.922,0.0016502109
4963178,"3","Slc25a3"
"Ctsd",1.28169804484358e-07,0.696612371255593,0.944,0.975,0.0021611992
4321525,"3","Ctsd"
"Naca",1.75850017550663e-07,0.508560350207807,0.8,0.931,0.002965182995
93929,"3","Naca"
"Atp5f1",2.23775820642437e-07,0.754040060590938,0.719,0.827,0.00377330
788767278,"3","Atp5f1"
"Rps5",2.23863181763571e-07,0.474641841106584,0.869,0.976,0.0037747809
7089734,"3","Rps5"
"Btf3",2.42768363059416e-07,0.580740694100008,0.744,0.914,0.0040935601
3790787,"3","Btf3"
"Rgs101",3.36157149097456e-07,0.503604418676692,0.831,0.922,0.00566828
184808131,"3","Rgs10"
"Tubb2a1",4.94620165852201e-07,0.295002220290479,0.262,0.589,0.0083402
8523659982,"3","Tubb2a"
"Cd812",6.1661001363031e-07,0.535773918114309,0.894,0.89,0.01039727804
98343,"3","Cd81"
"Oaz1",6.56205946457802e-07,0.42609776742132,0.881,0.976,0.01106494466
91715,"3","Oaz1"
"Ckb",6.67604251340275e-07,0.607297057397136,0.8,0.914,0.0112571428860
997,"3","Ckb"
"Tpt1",1.2849716487138e-06,0.37144540951517,0.963,0.993,0.021667191940
6121,"3","Tpt1"
"Ybx1",1.88829133887385e-06,0.808329789593626,0.781,0.909,0.0318403685
560908,"3","Ybx1"
"Lsm12",2.28918574969001e-06,0.657227170811952,0.181,0.444,0.038600250
1112729,"3","Lsm12"
"Rps6",2.68374827259413e-06,0.493840212209696,0.85,0.977,0.04525336337
24821,"3","Rps6"
"Bsg",3.25141595199378e-06,1.06031451425678,0.719,0.87,0.0548253757825
192,"3","Bsg"
"Pgam1",4.24487457003608e-06,1.42801217824454,0.625,0.692,0.0715770749

999484,"3","Pgam1"
"Maf1",5.62969900988252e-06,0.34084155568493,0.162,0.398,0.09492798470
46391,"3","Maf1"
"Npc2",5.72300702987847e-06,0.349898623305725,0.856,0.972,0.0965013445
378108,"3","Npc2"
"Rpl36al1",7.47043135331747e-06,0.256446868138378,0.294,0.608,0.125966
413479639,"3","Rpl36al"
"Gpi1",7.73140993233403e-06,0.894767359074974,0.688,0.798,0.1303670342
79016,"3","Gpi1"
"C1qc",8.29711806305741e-06,0.391550217330609,0.925,0.95,0.13990600477
9274,"3","C1qc"
"Basp1",8.63847909825866e-06,0.544333414752427,0.875,0.89,0.1456620345
54837,"3","Basp1"
"Slc2a1",8.70892663811843e-06,0.296450461470119,0.106,0.303,0.14684992
0971953,"3","Slc2a1"
"Capza2",9.53590855376106e-06,0.414857751202288,0.781,0.947,0.16079449
0033519,"3","Capza2"
"Gabarap",1.23169205790181e-05,0.411219983169569,0.775,0.942,0.2076879
14803403,"3","Gabarap"
"Vps35",1.67552046672243e-05,0.305038645903546,0.269,0.57,0.2825262610
98737,"3","Vps35"
"Tpi1",2.09855695079534e-05,1.59794676103045,0.556,0.601,0.35385867304
3111,"3","Tpi1"
"Ctsz",2.34794588011722e-05,0.416592031613686,0.863,0.985,0.3959106343
05365,"3","Ctsz"
"Eef1b2",2.97064352365601e-05,0.434362656752212,0.806,0.936,0.50090991
0958877,"3","Eef1b2"
"Tmem189",3.19889712297172e-05,0.3491430694588,0.162,0.395,0.539398032
875492,"3","Tmem189"
"Timm17a",3.22057050162536e-05,0.271784103795602,0.119,0.309,0.5430525
97984068,"3","Timm17a"
"Clta",3.45718696500291e-05,0.393857266583959,0.856,0.97,0.58295086603
879,"3","Clta"
"Eif3f",3.81657877887749e-05,0.538308745631462,0.706,0.887,0.643551513
694322,"3","Eif3f"
"Vdac2",4.59708449386278e-05,0.524948074066465,0.731,0.894,0.775160387
355142,"3","Vdac2"
"Mettl23",5.78944487876336e-05,0.297302849123202,0.169,0.385,0.9762161
95457078,"3","Mettl23"
"Atp5c1",6.29777670769816e-05,0.729526341508703,0.644,0.791,1,"3","Atp
5c1"
"Qars",6.72274829243868e-05,0.279856320842219,0.175,0.397,1,"3","Qars"
"Ost4",7.14332441942067e-05,0.325347283256305,0.212,0.461,1,"3","Ost4"
"Mpc1",7.9425461068274e-05,0.812317130536666,0.65,0.801,1,"3","Mpc1"
"Rex1bd",7.98454842952814e-05,0.259760145803496,0.131,0.323,1,"3","Rex
1bd"
"Ywhag",8.71074785159279e-05,0.255814546567509,0.181,0.4,1,"3","Ywhag"
"Card19",8.76028233302186e-05,0.250920311569639,0.206,0.447,1,"3","Car
d19"
"Ilk",9.20707137369327e-05,0.312509750847541,0.244,0.521,1,"3","Ilk"

"C1qa",0.000100211252197729,0.383478069750621,0.938,0.949,1,"3","C1qa"
"Ndufa6",0.000110102131061754,0.25470202922437,0.275,0.554,1,"3","Nduf
a6"
"Ftl1",0.000115984949864645,0.390717810747398,0.981,0.999,1,"3","Ftl1"
"Rpl121",0.000118075564136289,0.498457773507781,0.838,0.948,1,"3","Rpl
12"
"Supt4a",0.000119880938002239,0.332567160275841,0.219,0.467,1,"3","Sup
t4a"
"Eno1",0.000122170943350126,1.16410030325,0.6,0.685,1,"3","Eno1"
"Impdh2",0.000126843384431686,0.251559762429819,0.225,0.468,1,"3","Imp
dh2"
"Emc7",0.000127808403685465,0.306553440783104,0.288,0.595,1,"3","Emc7"
"Ldha",0.00013008159044962,1.3450318053563,0.656,0.819,1,"3","Ldha"
"Aif11",0.000178857872348995,0.564339901529343,0.713,0.859,1,"3","Aif1
"
"Rpl101",0.000179914271955863,0.391332521507564,0.887,0.972,1,"3","Rpl
10"
"Slc16a3",0.000207854095105215,0.269547123587573,0.212,0.431,1,"3","Sl
c16a3"
"Park7",0.000208666853360909,0.722192842218342,0.575,0.698,1,"3","Park
7"
"Rpl212",0.000239533457210008,0.547189083046848,0.881,0.951,1,"3","Rpl
21"
"Pgk1",0.000254098724834588,1.35786708765928,0.556,0.584,1,"3","Pgk1"
"Stx12",0.000254813275789392,0.293047050947919,0.15,0.337,1,"3","Stx12
"
"Rpl4",0.000279180512531789,0.443632984671647,0.762,0.943,1,"3","Rpl4"
"Rps91",0.000285850894546242,0.402711090133323,0.9,0.98,1,"3","Rps9"
"Selenof",0.00030502728525016,0.545483262445702,0.7,0.873,1,"3","Selen
of"
"Cotl1",0.000328798307522115,0.566994136877628,0.825,0.951,1,"3","Cotl
1"
"Emg1",0.000352868614548256,0.257012810526925,0.231,0.467,1,"3","Emg1"
"Slc25a17",0.000368507814911577,0.330159124115401,0.106,0.261,1,"3","S
lc25a17"
"Arpc2",0.0004148215699316,0.443879065271842,0.863,0.97,1,"3","Arpc2"
"St6galnac4",0.000416946318229469,0.305477401344776,0.231,0.476,1,"3",
"St6galnac4"
"1810037I17Rik",0.000458445419151448,0.27892847253535,0.212,0.427,1,"3
","1810037I17Rik"
"Dcaf12",0.000520150646619597,0.318418252231603,0.125,0.283,1,"3","Dca
f12"
"Arhgdia",0.000604144877600805,0.510740511435479,0.713,0.897,1,"3","Ar
hgdia"
"Rps71",0.000632179976611998,0.426475075178337,0.863,0.952,1,"3","Rps7
"
"Eef21",0.000673512208754432,0.347077017855727,0.831,0.967,1,"3","Eef2
"
"Tomm7",0.000701486982025984,0.252705252015678,0.244,0.487,1,"3","Tomm
7"

"Mxi1",0.000715832584697852,0.335760747873682,0.188,0.386,1,"3","Mxi1"
"Hadhb",0.000749444551229558,0.26264837143774,0.219,0.431,1,"3","Hadhb
"
"Pgp",0.000782232138328857,0.283760344109251,0.175,0.362,1,"3","Pgp"
"Eif3h",0.000864681401149211,0.572240257301611,0.662,0.808,1,"3","Eif3
h"
"Prelid1",0.000874747977900869,0.866144630520071,0.537,0.609,1,"3","Pr
elid1"
"Ndufs7",0.000895854283618779,0.396292059872368,0.269,0.531,1,"3","Ndu
fs7"
"Polr2c",0.000913314164229857,0.376426727593988,0.169,0.352,1,"3","Pol
r2c"
"Abhd17c",0.000998484659506644,0.293490871303533,0.112,0.256,1,"3","Ab
hd17c"
"Sf3a3",0.00103703517671984,0.260363905678292,0.125,0.275,1,"3","Sf3a3
"
"Nkap",0.00108008867869753,0.250946828867688,0.119,0.26,1,"3","Nkap"
"Rps6ka4",0.00111045748623278,0.274185680541085,0.188,0.375,1,"3","Rps
6ka4"
"Smox",0.0011484137430482,0.337949847813633,0.25,0.492,1,"3","Smox"
"Il11ra1",0.00118965395068124,0.283818585013778,0.175,0.356,1,"3","Il1
1ra1"
"Dazap1",0.00122657082646195,0.287350927553779,0.212,0.419,1,"3","Daza
p1"
"Ube2r2",0.00136012843803269,0.257574919988146,0.231,0.445,1,"3","Ube2
r2"
"Xpnpep1",0.00152958385858685,0.410586387140885,0.125,0.271,1,"3","Xpn
pep1"
"Got1",0.00158649092109467,0.356955849377439,0.144,0.297,1,"3","Got1"
"Psmd2",0.00170280224415479,0.311470029728718,0.256,0.477,1,"3","Psmd2
"
"Ube2e1",0.00176894780313159,0.380518878639448,0.25,0.474,1,"3","Ube2e
1"
"Gps1",0.00182600007959429,0.255636774205663,0.212,0.413,1,"3","Gps1"
"Dtnbp1",0.00189109261876592,0.297580240379913,0.25,0.472,1,"3","Dtnbp
1"
"Imp4",0.00193106016099453,0.282466009364553,0.169,0.336,1,"3","Imp4"
"Ebp",0.0019989261257818,0.324312684158442,0.269,0.506,1,"3","Ebp"
"Atp6v1g1",0.00205506301248256,0.603990367803659,0.681,0.854,1,"3","At
p6v1g1"
"Myl6",0.00209583527658009,0.939005577645763,0.662,0.874,1,"3","Myl6"
"Rab11a",0.00209612444352726,0.356632718661275,0.338,0.6,1,"3","Rab11a
"
"Mkrn1",0.00209642515187937,0.751336932651879,0.225,0.436,1,"3","Mkrn1
"
"Gatad1",0.00210372610250703,0.254719998993932,0.138,0.283,1,"3","Gata
d1"
"Jam2",0.00232595376411677,0.255313877549912,0.119,0.251,1,"3","Jam2"
"Fez2",0.00234415682524805,0.28622534372587,0.225,0.43,1,"3","Fez2"
"Trem2",0.00234577233082398,0.371648186024093,0.875,0.941,1,"3","Trem2

"
"Sarnp",0.00234980619534786,0.263011437409934,0.219,0.413,1,"3","Sarnp
"
"Uba2",0.00247976545854372,0.343103523542294,0.256,0.489,1,"3","Uba2"
"Rpl111",0.00248919753790222,0.456539785552984,0.806,0.964,1,"3","Rpl1
1"
"Gipc1",0.00250525477814256,0.30795135367148,0.225,0.424,1,"3","Gipc1"
"Snrpf",0.00258318891847816,0.254874199311335,0.194,0.374,1,"3","Snrpf
"
"Syf2",0.0026289225448869,0.296883422204314,0.281,0.53,1,"3","Syf2"
"Lamp1",0.00265189265701971,0.358182670744672,0.825,0.971,1,"3","Lamp1
"
"Uqcr10",0.00270319221028778,0.319201886669393,0.238,0.452,1,"3","Uqcr
10"
"Rhoa",0.00272558007444715,0.45498621258038,0.781,0.95,1,"3","Rhoa"
"Camk2n11",0.00280981244616648,0.281201341076327,0.206,0.39,1,"3","Cam
k2n1"
"Bcap31",0.00283276747550636,0.260482458597473,0.381,0.685,1,"3","Bcap
31"
"Cst7",0.00293301986953085,0.907247600440413,0.288,0.23,1,"3","Cst7"
"Ncoa4",0.00297291972283215,0.294086453011682,0.156,0.308,1,"3","Ncoa4
"
"Smim14",0.00301971050881798,0.34089257272378,0.262,0.487,1,"3","Smim1
4"
"Spg21",0.00313632277174803,0.323020264483918,0.256,0.479,1,"3","Spg21
"
"Vta1",0.00314651613699591,0.278481815390648,0.169,0.332,1,"3","Vta1"
"Commd6",0.00330068968587801,0.349875542297621,0.131,0.265,1,"3","Comm
d6"
"Slc25a11",0.00336655593842764,0.258414741859063,0.331,0.614,1,"3","Sl
c25a11"
"Tspan4",0.00341467905664879,0.411117221911646,0.256,0.483,1,"3","Tspa
n4"
"Txnl1",0.00343177899977761,0.329918210841647,0.331,0.603,1,"3","Txnl1
"
"Ube2e3",0.00346360461446658,0.255113609200503,0.262,0.485,1,"3","Ube2
e3"
"Elob",0.00351222928428778,0.3801650451103,0.331,0.602,1,"3","Elob"
"Sdhc",0.0037056937338014,0.298806091573578,0.2,0.371,1,"3","Sdhc"
"Gpx4",0.00377772483607521,0.65356953981121,0.662,0.836,1,"3","Gpx4"
"Ppp3r1",0.00380658656940278,0.259412227263171,0.138,0.279,1,"3","Ppp3
r1"
"Bad",0.00396815343487914,0.264142475898729,0.188,0.358,1,"3","Bad"
"Pomp",0.0040520845012732,0.514999869042136,0.644,0.827,1,"3","Pomp"
"Mfsd10",0.00409545995396384,0.319514620589606,0.181,0.345,1,"3","Mfsd
10"
"Atp6v0d1",0.00411513910441405,0.279764833329186,0.312,0.565,1,"3","At
p6v0d1"
"Ifi35",0.00423401677452365,0.281778897241098,0.175,0.331,1,"3","Ifi35
"

"Aamp",0.00430926425540914,0.290008020729112,0.344,0.624,1,"3","Aamp"
"Emc2",0.00431993240395402,0.268175052118218,0.219,0.416,1,"3","Emc2"
"Cops8",0.00439044844192568,0.358009639306214,0.125,0.255,1,"3","Cops8
"
"Bag1",0.00462554716290312,0.734716535469641,0.569,0.702,1,"3","Bag1"
"Arpc5l",0.00466553179289392,0.434744316270709,0.269,0.501,1,"3","Arpc
5l"
"Lgals3",0.00473393372459398,1.37655395133588,0.312,0.238,1,"3","Lgals
3"
"Map1lc3a",0.00476081702589028,0.353222006556899,0.275,0.506,1,"3","Ma
p1lc3a"
"Papss1",0.00490743921225488,0.257709184974759,0.269,0.485,1,"3","Paps
s1"
"Rpl271",0.00493545360965896,0.300754106486121,0.4,0.707,1,"3","Rpl27"
"Etfa",0.00507543807272235,0.338025083754983,0.175,0.336,1,"3","Etfa"
"Hexa",0.00512807094371858,0.452120060757904,0.738,0.923,1,"3","Hexa"
"Cuta",0.00515094710438368,0.277819411368812,0.269,0.489,1,"3","Cuta"
"Mrpl32",0.00527008941994778,0.283228590010215,0.138,0.274,1,"3","Mrpl
32"
"Psma4",0.00540110977595829,0.272297584804416,0.344,0.6,1,"3","Psma4"
"Eif3g",0.00561114867842785,0.389615405829866,0.238,0.428,1,"3","Eif3g
"
"Commd9",0.00588702850418463,0.328183648561176,0.181,0.343,1,"3","Comm
d9"
"Cpsf3",0.0059902231390233,0.373285197475589,0.162,0.312,1,"3","Cpsf3"
"Ssna1",0.00604507748960363,0.317088439723404,0.144,0.279,1,"3","Ssna1
"
"Cfdp1",0.00604855107706263,0.317991364743179,0.206,0.376,1,"3","Cfdp1
"
"Tspan3",0.00609107482952016,0.259108755360704,0.238,0.44,1,"3","Tspan
3"
"Prmt1",0.00640234766743713,0.340349376651845,0.262,0.475,1,"3","Prmt1
"
"Trappc6b",0.00646670189628842,0.290848673461051,0.244,0.434,1,"3","Tr
appc6b"
"Eif5a",0.00650393906228269,0.650587314813234,0.706,0.888,1,"3","Eif5a
"
"Nap1l1",0.00656004702790347,0.260120551879349,0.275,0.488,1,"3","Nap1
l1"
"Ppp1r14b",0.00658218513677428,0.783628722257045,0.575,0.728,1,"3","Pp
p1r14b"
"Lsm6",0.00663263916110869,0.301661130242346,0.194,0.362,1,"3","Lsm6"
"Thoc7",0.00667390951983607,0.259230941282169,0.194,0.357,1,"3","Thoc7
"
"Rpl27a2",0.00672763077205682,0.491305985761849,0.881,0.966,1,"3","Rpl
27a"
"Krtcap2",0.00675922411892707,0.359767938155625,0.238,0.428,1,"3","Krt
cap2"
"9030624J02Rik",0.0068375127547093,0.304778948722894,0.188,0.346,1,"3"
,"9030624J02Rik"

"Eif3b",0.0068749822280281,0.323961934141421,0.225,0.413,1,"3","Eif3b"
"Abracl",0.0070053861110636,0.281304098827921,0.331,0.577,1,"3","Abrac
l"
"Gabarapl2",0.00724371015803614,0.36623797264419,0.35,0.613,1,"3","Gab
arapl2"
"Arl6ip5",0.00727770991218988,0.259334468914089,0.344,0.603,1,"3","Arl
6ip5"
"Cd63",0.00734917527172908,0.554519410207372,0.75,0.899,1,"3","Cd63"
"2310011J03Rik",0.00737812305966508,0.328607530621003,0.156,0.299,1,"3
","2310011J03Rik"
"Lrpap1",0.00737992488750966,0.294877247708483,0.331,0.587,1,"3","Lrpa
p1"
"Cuedc2",0.00743085233652866,0.298635077011162,0.306,0.551,1,"3","Cued
c2"
"Hspa4",0.0078097495492414,0.266709689554192,0.375,0.647,1,"3","Hspa4"
"Psmb7",0.00792678893520008,0.310011981805546,0.281,0.497,1,"3","Psmb7
"
"Dctn2",0.00795479981547866,0.292910191209133,0.344,0.62,1,"3","Dctn2"
"Cox4i1",0.00807171238543782,0.464389963491932,0.719,0.917,1,"3","Cox4
i1"
"Clptm1l",0.00816345237504925,0.393724998225295,0.3,0.546,1,"3","Clptm
1l"
"Zcrb1",0.00826742898805223,0.281189525204903,0.269,0.468,1,"3","Zcrb1
"
"1110004F10Rik",0.00861551900012203,0.420158610811312,0.262,0.469,1,"3
","1110004F10Rik"
"Akr1b3",0.00865450781495445,0.372462864287857,0.325,0.553,1,"3","Akr1
b3"
"Fcrls",0.00881325931793974,0.549652473464445,0.781,0.896,1,"3","Fcrls
"
"Cox7a2l",0.00883004003708319,0.501045320706049,0.656,0.861,1,"3","Cox
7a2l"
"Trappc2l",0.00887258067902806,0.252006863831918,0.206,0.376,1,"3","Tr
appc2l"
"Rps81",0.00919574015636966,0.355397703264637,0.9,0.977,1,"3","Rps8"
"Anapc2",0.00926234844761108,0.268753038264321,0.131,0.251,1,"3","Anap
c2"
"Rpl362",0.009304704195589,0.321646024482248,0.6,0.857,1,"3","Rpl36"
"Arhgdib",0.00935576223615316,0.452686041276021,0.7,0.884,1,"3","Arhgd
ib"
"Rogdi",0.00941982215534282,0.449103844053172,0.231,0.421,1,"3","Rogdi
"
"Xlr",0.00946079769462435,0.347072484578997,0.225,0.406,1,"3","Xlr"
"Cd38",2.32756353725412e-231,1.77126417250513,0.759,0.019,3.9247376365
1789e-227,"4","Cd38"
"Bmp2",1.73264901812027e-229,2.92982924339247,0.677,0.01,2.92159277435
44e-225,"4","Bmp2"
"Cp",4.38875684407228e-216,2.20177342573187,0.722,0.02,7.4003217904746
8e-212,"4","Cp"
"Ms4a7",1.39255762636649e-215,2.77830160703549,0.962,0.065,2.348130669

57918e-211,"4","Ms4a7"
"Mrc1",1.12424140819354e-212,3.30777371502049,0.949,0.066,1.8956958624
9595e-208,"4","Mrc1"
"Serpinb8",4.66471742135221e-197,1.49850879015473,0.658,0.018,7.865646
5158841e-193,"4","Serpinb8"
"Pf4",3.86946527839643e-177,3.15746926850581,0.899,0.076,6.52469235243
206e-173,"4","Pf4"
"Ifi203",9.0184643531902e-174,1.92779176660217,0.741,0.041,1.520693459
23493e-169,"4","Ifi203"
"Clec4n",4.75543968981023e-165,2.30597974554569,0.899,0.086,8.01862240
495801e-161,"4","Clec4n"
"Cd163",1.41480061748754e-162,1.66811816465106,0.595,0.02,2.3856368012
0749e-158,"4","Cd163"
"F13a1",4.65037474582776e-155,2.48354318181875,0.753,0.057,7.841461896
41477e-151,"4","F13a1"
"Aoah",1.19279253636907e-154,1.16001014522222,0.703,0.044,2.0112867748
2553e-150,"4","Aoah"
"Lyve1",3.61978400768153e-153,2.5201334156914,0.589,0.024,6.1036797937
526e-149,"4","Lyve1"
"Ccr1",1.60873450541076e-148,2.67346359403476,0.962,0.147,2.7126481230
2363e-144,"4","Ccr1"
"Ednrb",2.02191609604663e-147,1.25236939809237,0.538,0.018,3.409354921
15382e-143,"4","Ednrb"
"Cd93",3.76695126565566e-147,1.03293592759983,0.5,0.013,6.351833224148
58e-143,"4","Cd93"
"Adam19",8.40795844468033e-139,0.674565279206652,0.418,0.006,1.4177499
52942e-134,"4","Adam19"
"C2",2.36376356408636e-138,1.02303952718241,0.557,0.025,3.985778121762
42e-134,"4","C2"
"Dab2",1.04913155446814e-137,2.80160873634128,0.937,0.15,1.76904562714
419e-133,"4","Dab2"
"Itsn1",6.3138494263843e-132,0.756052915934252,0.532,0.024,1.064641290
27692e-127,"4","Itsn1"
"Gm4951",8.89904595413579e-131,0.74864143230613,0.437,0.011,1.50055712
878638e-126,"4","Gm4951"
"Cd28",9.14405699278103e-127,0.887665410540476,0.418,0.009,1.541870890
12274e-122,"4","Cd28"
"Ifi207",2.83057614965833e-121,2.28332888346385,0.956,0.194,4.77291750
355388e-117,"4","Ifi207"
"Iigp1",8.54322530959345e-118,0.801337054178518,0.418,0.012,1.44055865
170365e-113,"4","Iigp1"
"Mndal",1.54903478108652e-117,1.26752151257415,0.703,0.069,2.611982447
86809e-113,"4","Mndal"
"Pltp",3.61495738314933e-111,0.968612293264343,0.557,0.04,6.0955411394
664e-107,"4","Pltp"
"Eps8",1.5769226657258e-109,1.20495540806548,0.69,0.078,2.659006998946
84e-105,"4","Eps8"
"Pla2g7",3.93311078815485e-109,1.5553571896922,0.854,0.129,6.632011410
98672e-105,"4","Pla2g7"
"Cfp",5.74846202340551e-109,1.3488115128126,0.665,0.069,9.693056663866

37e-105,"4","Cfp"
"Iqgap2",2.12126243711359e-105,0.659562660386181,0.475,0.027,3.5768727
2146094e-101,"4","Iqgap2"
"Ccl24",9.72375590482054e-105,2.09018517232677,0.525,0.04,1.6396197206
7084e-100,"4","Ccl24"
"Tspan8",1.11784922733552e-104,0.589489374529106,0.386,0.013,1.8849173
6713315e-100,"4","Tspan8"
"Adam33",1.97904373966598e-104,0.664316547709695,0.367,0.011,3.3370635
5382478e-100,"4","Adam33"
"Cybb",1.22858160770708e-98,1.67361428192511,0.968,0.2,2.0716343069156
8e-94,"4","Cybb"
"Gpx3",8.16721266737439e-98,1.13867284347476,0.684,0.088,1.37715539997
267e-93,"4","Gpx3"
"Gas7",4.70971106445899e-95,1.18038725932952,0.715,0.107,7.94151479689
075e-91,"4","Gas7"
"Fcna",2.49505538120432e-94,1.02266875878416,0.449,0.029,4.20716238378
672e-90,"4","Fcna"
"Ecm1",5.0020914119087e-94,0.707398636184863,0.5,0.039,8.4345265387604
5e-90,"4","Ecm1"
"Msr1",1.06548010469546e-93,0.959247696222434,0.627,0.071,1.7966125525
3748e-89,"4","Msr1"
"Cxcl2",7.28725935322498e-93,3.36652731766054,0.861,0.201,1.2287776721
408e-88,"4","Cxcl2"
"Rnasel",8.21237719589734e-92,1.23127625097618,0.709,0.108,1.384771042
77221e-87,"4","Rnasel"
"Ifi211",9.63659251434651e-92,1.00044665399467,0.576,0.058,1.624922229
76911e-87,"4","Ifi211"
"Colec12",1.12230070828246e-90,0.806111543151693,0.43,0.027,1.89242345
430589e-86,"4","Colec12"
"St8sia4",2.98305045996005e-90,1.19482621295906,0.601,0.07,5.030019685
58464e-86,"4","St8sia4"
"Ms4a14",3.35068369082364e-90,0.425942492299178,0.291,0.006,5.64992283
946682e-86,"4","Ms4a14"
"Mdfic",9.14790768556969e-87,0.598496286398835,0.481,0.04,1.5425201939
4076e-82,"4","Mdfic"
"Ccl7",1.5222504549251e-86,3.77976265959142,0.816,0.197,2.566818717094
71e-82,"4","Ccl7"
"Oasl2",1.32784122154776e-85,1.07854149029725,0.595,0.073,2.2390058677
7383e-81,"4","Oasl2"
"Wwp1",3.60482413731496e-85,1.08218544781611,0.791,0.157,6.07845446034
049e-81,"4","Wwp1"
"Ms4a6c",4.43952964792919e-85,1.77086994734882,0.975,0.318,7.485934892
3382e-81,"4","Ms4a6c"
"Ifi213",7.2073255429051e-82,0.613613904204232,0.361,0.019,1.215299233
04466e-77,"4","Ifi213"
"Sash1",2.64066569854833e-81,1.08448275019552,0.766,0.152,4.4526905008
9219e-77,"4","Sash1"
"Oasl1",5.88792496248465e-81,0.924648535626992,0.38,0.023,9.9282190717
4161e-77,"4","Oasl1"
"Rsad2",2.16816399276094e-80,1.05393008147748,0.418,0.031,3.6559581245

935e-76,"4","Rsad2"
"Ms4a6b",2.251371608536e-80,1.70621063748841,0.943,0.305,3.79626280631341e-76,"4","Ms4a6b"
"Folr2",5.6422608847983e-79,1.41615000485263,0.532,0.063,9.51398030394689e-75,"4","Folr2"
"Ddx60",4.08357245608533e-77,0.850780681127906,0.506,0.057,6.88571987545109e-73,"4","Ddx60"
"Clec4a1",1.47340422522448e-75,0.845904439150249,0.652,0.096,2.48445420457351e-71,"4","Clec4a1"
"Gm6377",2.2191781082903e-75,1.31934736888455,0.709,0.133,3.74197812619911e-71,"4","Gm6377"
"Cyr61",6.88550712672414e-74,0.767072869662519,0.278,0.01,1.16103421170822e-69,"4","Cyr61"
"Tfec",6.93316861136492e-73,0.445539561643566,0.373,0.027,1.16907089124835e-68,"4","Tfec"
"Bank1",2.71780305234621e-72,0.757513525150764,0.557,0.076,4.58275950686618e-68,"4","Bank1"
"Dse",6.76779293195048e-72,0.804124085389237,0.633,0.103,1.14118524418549e-67,"4","Dse"
"Ifi47",1.3374027816234e-71,0.487526598016681,0.424,0.038,2.25512857037338e-67,"4","Ifi47"
"Tgfbi",8.11119843272339e-71,1.2693021553029,0.867,0.229,1.36771027972582e-66,"4","Tgfbi"
"Mtss1",2.23926848613914e-70,1.17320392524672,0.778,0.178,3.77585452132782e-66,"4","Mtss1"
"Igfbp4",7.2419656010201e-69,1.79598226899854,0.842,0.248,1.22114023964401e-64,"4","Igfbp4"
"Tmem2",1.10991975305967e-67,0.496535023092528,0.354,0.026,1.87154668760922e-63,"4","Tmem2"
"Gbp7",3.96396993201883e-67,1.27312401391715,0.797,0.211,6.68404609937016e-63,"4","Gbp7"
"Bst2",4.20604143038984e-67,1.73178084852482,0.981,0.568,7.09222705992335e-63,"4","Bst2"
"Oas2",7.4003663780238e-67,0.397590280917789,0.285,0.014,1.24784977866237e-62,"4","Oas2"
"Stab1",4.71187772189856e-66,1.78468342112134,0.949,0.442,7.94516821466536e-62,"4","Stab1"
"Rnf150",6.75020537225431e-66,0.743905222521685,0.551,0.081,1.13821962986952e-61,"4","Rnf150"
"Egfl7",2.01793089341025e-64,0.433301964341939,0.272,0.013,3.40263507246836e-60,"4","Egfl7"
"Ifit3",5.31510684745379e-63,1.06534611780171,0.481,0.064,8.96233316617659e-59,"4","Ifit3"
"Slfn5",6.04423410149545e-63,1.04633719900886,0.658,0.128,1.01917875419416e-58,"4","Slfn5"
"Kitl",9.32248210649402e-62,0.380833908997883,0.316,0.023,1.57195693279702e-57,"4","Kitl"
"Tmem8",2.39976206009109e-61,0.387921518687859,0.285,0.017,4.0464787857256e-57,"4","Tmem8"
"Serpinb6a",8.0551561507402e-61,0.904162190855076,0.671,0.138,1.358260

43013781e-56,"4","Serpinb6a"
"Gpr65",1.81723573170733e-60,1.14625321315651,0.772,0.208,3.0642228908
049e-56,"4","Gpr65"
"Cbr2",2.81809089665347e-60,0.647470763492224,0.304,0.021,4.7518648699
3708e-56,"4","Cbr2"
"Ifi27l2a",8.78066833166203e-60,1.48221858156852,0.854,0.264,1.4805962
9408485e-55,"4","Ifi27l2a"
"Myo5a",4.73481782184398e-59,1.08877756555898,0.747,0.203,7.9838498111
9332e-55,"4","Myo5a"
"Aph1c",1.81002653261433e-58,0.491519175904615,0.475,0.066,3.052066739
29429e-54,"4","Aph1c"
"Irf7",2.39998587130986e-58,0.852409545204226,0.722,0.166,4.0468561762
0268e-54,"4","Irf7"
"Tbc1d4",2.91766737110622e-58,0.754124310215181,0.475,0.067,4.91977072
11593e-54,"4","Tbc1d4"
"Rab7b",3.13093454205215e-58,0.975645808631665,0.696,0.155,5.279381824
80834e-54,"4","Rab7b"
"Cd200r1",9.97318707031018e-58,0.523603402680393,0.481,0.069,1.6816788
037957e-53,"4","Cd200r1"
"Rtp4",4.97425734251705e-57,1.04074048856625,0.652,0.142,8.38759273095
225e-53,"4","Rtp4"
"Psd3",6.81760071957218e-57,0.490140255149483,0.31,0.025,1.14958383333
426e-52,"4","Psd3"
"Ifi206",8.36860099748092e-57,0.476445889540113,0.291,0.021,1.41111350
019523e-52,"4","Ifi206"
"Clec10a",8.4322214720343e-56,0.355015539964831,0.253,0.014,1.42184118
461442e-51,"4","Clec10a"
"Iqgap1",1.11343708947077e-55,0.543711785995609,0.81,0.194,1.877477620
26561e-51,"4","Iqgap1"
"Ifi204",1.49755243065985e-55,1.34211787404603,0.867,0.312,2.525172908
57864e-51,"4","Ifi204"
"Ctsc1",1.88432910200962e-55,1.43258356489423,0.994,0.88,3.17735573180
863e-51,"4","Ctsc"
"Ifitm3",7.46677899406294e-55,1.01330361364718,0.854,0.254,1.259048273
97889e-50,"4","Ifitm3"
"Tiparp",7.84485222407995e-54,1.39424020522569,0.848,0.315,1.322798982
02436e-49,"4","Tiparp"
"Reps2",2.83963285239547e-53,0.348377103024638,0.272,0.019,4.788188915
70925e-49,"4","Reps2"
"Tmem106a",3.55053592224665e-53,0.716189721675064,0.677,0.161,5.986913
6720923e-49,"4","Tmem106a"
"Raph1",5.4649029693503e-53,0.693021812177612,0.494,0.082,9.2149193869
1848e-49,"4","Raph1"
"Stard8",2.42501291950733e-52,0.8708783357058,0.589,0.128,4.0890567848
7327e-48,"4","Stard8"
"Ehd4",4.58736122695995e-52,1.38793971786415,0.962,0.677,7.73520850089
986e-48,"4","Ehd4"
"Clec4a2",7.93940105396347e-52,0.999532027322301,0.835,0.29,1.33874180
571932e-47,"4","Clec4a2"
"Rad51b",1.76849502974997e-51,0.36995161935552,0.31,0.028,2.9820363191

644e-47,"4","Rad51b"
"Il18bp",8.09587029183847e-51,0.339816255443624,0.278,0.022,1.36512564 86098e-46,"4","Il18bp"
"Hip1",1.94087653118275e-50,0.617649863610907,0.443,0.067,3.2727060068 8036e-46,"4","Hip1"
"Apoe",2.49882259650027e-50,1.05560140658468,0.994,0.99,4.213514662218 75e-46,"4","Apoe"
"Lzts2",5.98790117028198e-49,0.529854306480931,0.316,0.033,1.009679895 33295e-44,"4","Lzts2"
"Nfxl1",1.1035873685556e-48,0.73309341631232,0.614,0.145,1.86086902085 845e-44,"4","Nfxl1"
"Fcgrt",3.56623886262347e-48,1.5651825429944,0.943,0.594,6.01339197015 57e-44,"4","Fcgrt"
"Maf",6.23732185528814e-48,1.56783768677904,0.93,0.543,1.0517372112386 9e-43,"4","Maf"
"Mpp1",1.18765485547369e-47,0.921326937895977,0.804,0.264,2.0026236172 9973e-43,"4","Mpp1"
"Afap1l1",4.09214300594613e-47,0.37308977105884,0.354,0.044,6.90017153 662637e-43,"4","Afap1l1"
"Ets2",5.55764236638314e-47,1.0230210392303,0.854,0.335,9.371296558195 25e-43,"4","Ets2"
"Ehd1",5.87517217170821e-47,1.38079881911753,0.949,0.519,9.90671531593 439e-43,"4","Ehd1"
"Samd9l",1.01679166528915e-46,0.480074152071077,0.38,0.053,1.714514106 01056e-42,"4","Samd9l"
"Gimap6",2.2420349325375e-46,0.873041034070401,0.481,0.093,3.780519303 24473e-42,"4","Gimap6"
"Ifitm2",2.67486968757504e-46,0.314839954743054,0.722,0.184,4.51036526 718904e-42,"4","Ifitm2"
"Rilpl21",3.20777560138124e-46,1.34861736768696,0.924,0.458,5.40895121 904905e-42,"4","Rilpl2"
"Lrp6",5.19901362713406e-46,0.762472152860455,0.715,0.212,8.7665767780 7346e-42,"4","Lrp6"
"Hgsnat",2.41994113284569e-45,1.5340399110682,0.715,0.233,4.0805047382 0441e-41,"4","Hgsnat"
"Ifit2",2.43506516812405e-45,0.981675337950717,0.354,0.048,4.106006886 49078e-41,"4","Ifit2"
"Ccl21",2.68876271119073e-45,2.2144817335562,0.949,0.597,4.53379168360 98e-41,"4","Ccl2"
"Cass4",7.96556806867061e-45,0.508064294396087,0.43,0.072,1.3431540877 3924e-40,"4","Cass4"
"Myof",8.48491576697328e-45,0.296653744608975,0.259,0.022,1.4307264966 2703e-40,"4","Myof"
"Nr4a2",9.25078141796464e-45,0.96668447002377,0.468,0.089,1.5598667626 972e-40,"4","Nr4a2"
"Snx2",1.25779309193532e-44,1.3324040802826,0.937,0.563,2.120890711621 34e-40,"4","Snx2"
"Hbegf",2.58853088902413e-43,0.741159051618925,0.354,0.051,4.364780785 07249e-39,"4","Hbegf"
"Lgals1",4.3814041840223e-43,0.543451056492251,0.633,0.155,7.387923735

0984e-39,"4","Lgals1"
"Klf61",8.6236611695928e-43,1.37688935429723,1,0.754,1.45412174641674e-38,"4","Klf6"
"Nrap",1.42774845177065e-42,0.336036959212774,0.253,0.023,2.40746943937568e-38,"4","Nrap"
"Fcgr2b",1.96787631202762e-42,1.53028204533693,0.943,0.664,3.31823303734098e-38,"4","Fcgr2b"
"Rcbtb2",3.66775761680398e-42,1.03141422934561,0.937,0.555,6.18457289345487e-38,"4","Rcbtb2"
"Fam43a",6.8918902991713e-42,0.486153420843543,0.253,0.024,1.16211054224627e-37,"4","Fam43a"
"Hacd4",8.44069075403003e-42,0.894147467120743,0.778,0.295,1.42326927494454e-37,"4","Hacd4"
"Rgl1",1.88485839332058e-41,0.911676098907934,0.684,0.205,3.17824822281716e-37,"4","Rgl1"
"Cpq",2.10183183287005e-41,0.564695226658324,0.513,0.11,3.54410883658548e-37,"4","Cpq"
"Gab3",3.3248483241322e-41,0.330243077675761,0.297,0.035,5.60635924415171e-37,"4","Gab3"
"Smagp",3.44140192818493e-41,0.77462304876314,0.778,0.266,5.80289193130543e-37,"4","Smagp"
"Tubb6",9.76749751263561e-41,0.83795715585559,0.69,0.214,1.64699543058062e-36,"4","Tubb6"
"Isg15",1.18296378289856e-40,1.36246211952675,0.595,0.157,1.99471353072356e-36,"4","Isg15"
"Cd861",2.83850017724865e-40,1.0845437275866,0.981,0.692,4.78627899887668e-36,"4","Cd86"
"H2-
K1",4.41544272950522e-40,0.95828940212178,1,0.904,7.44531953049169e-36,"4","H2-K1"
"Pim1",4.83030896592853e-40,1.26501027756244,0.968,0.592,8.14486697834868e-36,"4","Pim1"
"Stap1",2.21138925124621e-39,0.557993850333981,0.424,0.076,3.72884455545136e-35,"4","Stap1"
"Alox5",5.20553194618002e-39,0.417939006760599,0.392,0.069,8.77756796764875e-35,"4","Alox5"
"Oas1a",7.33285100223427e-39,0.458484554837389,0.437,0.085,1.23646533599674e-34,"4","Oas1a"
"P2rx7",8.47783822405674e-39,0.969565629421659,0.728,0.266,1.42953308134045e-34,"4","P2rx7"
"Tmem176b",1.14421042952882e-38,0.914152442072004,0.994,0.852,1.9293676262715e-34,"4","Tmem176b"
"Paox",1.51191631794136e-38,0.579172816452866,0.633,0.184,2.54939329531272e-34,"4","Paox"
"Baiap2",4.86569260446214e-38,0.926554341689705,0.601,0.17,8.20453086964406e-34,"4","Baiap2"
"Fosb1",5.15212849801066e-38,1.17524215973944,0.937,0.535,8.68751907334557e-34,"4","Fosb"
"Sp100",5.98445869199938e-38,0.663790835282722,0.665,0.205,1.00909942464494e-33,"4","Sp100"

"Ptger4",8.15164060193741e-38,0.830277437264786,0.475,0.104,1.37452963829869e-33,"4","Ptger4"
"Trpv4",8.21742106694542e-38,0.291468574988033,0.272,0.032,1.38562154030834e-33,"4","Trpv4"
"Arhgap15",3.52785146933094e-37,0.495645275949139,0.532,0.128,5.94866314758582e-33,"4","Arhgap15"
"Hcar2",8.31987265338529e-37,0.921830189414757,0.544,0.14,1.40289692681383e-32,"4","Hcar2"
"Dclre1c1",1.44468079827285e-36,0.807029296354234,0.696,0.227,2.43602076204767e-32,"4","Dclre1c"
"Gas6",1.53114187890644e-36,1.3110447629663,0.905,0.582,2.58181143621203e-32,"4","Gas6"
"Vcam1",2.85536056528416e-36,0.999160423171492,0.348,0.061,4.81470898518216e-32,"4","Vcam1"
"Blvrb",9.18807707022134e-36,0.821707404738004,0.905,0.499,1.54929355558072e-31,"4","Blvrb"
"Plk2",2.01413372019324e-35,0.955524840924945,0.411,0.086,3.39623227898985e-31,"4","Plk2"
"Lpar61",2.05294881641704e-35,0.930210725207771,0.715,0.273,3.46168229424241e-31,"4","Lpar6"
"Blvra",2.15328429727621e-35,0.911964704757886,0.728,0.28,3.63086798206715e-31,"4","Blvra"
"Trps1",3.82850424984619e-35,0.401806359250675,0.551,0.138,6.45562386609064e-31,"4","Trps1"
"Ifi209",6.52705007321042e-35,0.442530230403279,0.411,0.083,1.10059118334474e-30,"4","Ifi209"
"Nrp1",1.30124551753352e-34,1.12714751682414,0.886,0.472,2.19416019166503e-30,"4","Nrp1"
"Cyp27a1",1.45851670341007e-34,0.544099759313219,0.595,0.171,2.45935086529007e-30,"4","Cyp27a1"
"Lifr",1.67345621542089e-34,0.718477997373757,0.608,0.182,2.8217818704427e-30,"4","Lifr"
"Igf1",1.88653344514314e-34,0.471345386730251,0.696,0.217,3.18107269520037e-30,"4","Igf1"
"Metrnl",1.91422964811901e-34,0.626391564635374,0.582,0.163,3.22777403265827e-30,"4","Metrnl"
"Slc9a9",2.18957603040838e-34,0.705780647901849,0.753,0.283,3.69206310247462e-30,"4","Slc9a9"
"Clec12a",2.50449846784776e-34,0.493880452001167,0.348,0.06,4.2230853164849e-30,"4","Clec12a"
"Zbp1",3.33800616771994e-34,0.577343214021606,0.462,0.105,5.62854600000937e-30,"4","Zbp1"
"Eps15",3.41561380390839e-34,0.615844205257656,0.747,0.281,5.75940799615032e-30,"4","Eps15"
"Ifit1",6.43392302173145e-34,0.513501405625123,0.253,0.032,1.08488809992436e-29,"4","Ifit1"
"Pxdc1",6.6413974117886e-34,0.788185055792222,0.652,0.219,1.11987243157579e-29,"4","Pxdc1"
"Clcn5",7.99124065871582e-34,0.446310712563794,0.399,0.083,1.34748299987266e-29,"4","Clcn5"

"Adgre1",9.91687908288732e-34,0.802583134687199,0.981,0.726,1.67218415095646e-29,"4","Adgre1"
"C4b",1.90825674678566e-33,0.540429233949753,0.316,0.053,3.21770252642998e-29,"4","C4b"
"Nfil3",2.16259689367236e-33,0.676505413165786,0.513,0.131,3.64657088211033e-29,"4","Nfil3"
"Arhgef3",2.21729466381601e-33,0.406210176220522,0.367,0.07,3.73880226212656e-29,"4","Arhgef3"
"Selenop",1.05652785261741e-32,0.94556562960447,0.994,0.926,1.78151726508348e-28,"4","Selenop"
"Sbf2",1.5308798973331e-32,0.695828229860641,0.715,0.275,2.58136968288307e-28,"4","Sbf2"
"Rcan11",2.86805687068537e-32,1.25869477065399,0.905,0.492,4.83611749534966e-28,"4","Rcan1"
"Fosl2",4.60487652269849e-32,0.808577898414594,0.747,0.29,7.76474279257419e-28,"4","Fosl2"
"Prkacb",7.63228310947307e-32,0.559514953862056,0.601,0.189,1.28695557791935e-27,"4","Prkacb"
"Ralgds",1.66567045437695e-31,0.371764666687206,0.38,0.078,2.80865352017042e-27,"4","Ralgds"
"Rasgef1b1",2.50350362154705e-31,0.977215963572301,0.829,0.388,4.22140780665263e-27,"4","Rasgef1b"
"Stk17b",2.50485517731063e-31,0.580310289445768,0.778,0.329,4.22368679998119e-27,"4","Stk17b"
"Ap2a2",3.89326054024628e-31,0.549872656011644,0.658,0.222,6.56481592296328e-27,"4","Ap2a2"
"Ap1b1",6.87547606127497e-31,0.753696262911358,0.696,0.277,1.15934277345219e-26,"4","Ap1b1"
"Lilrb4a",6.95151572990389e-31,0.517688826989154,0.595,0.179,1.17216458237639e-26,"4","Lilrb4a"
"Rnf213",7.21531722671269e-31,0.658587491629367,0.582,0.189,1.21664679076829e-26,"4","Rnf213"
"Usp18",1.14363012719058e-30,0.443929003363883,0.323,0.06,1.92838912046876e-26,"4","Usp18"
"Twf1",2.39077069843189e-30,0.884241766133371,0.741,0.318,4.03131755169586e-26,"4","Twf1"
"Rbms1",2.49271158062311e-30,0.661016141468765,0.854,0.371,4.20321026724668e-26,"4","Rbms1"
"Mapre2",1.19954344688186e-29,0.745018029869192,0.753,0.327,2.0226701601322e-25,"4","Mapre2"
"Arhgap18",2.13224993233836e-29,0.516482195014164,0.544,0.165,3.59539983590894e-25,"4","Arhgap18"
"Snx6",2.78448679044974e-29,0.933946744903236,0.892,0.555,4.69520162605635e-25,"4","Snx6"
"Atp2b1",2.98740991758192e-29,0.895318597955332,0.962,0.703,5.03737060302664e-25,"4","Atp2b1"
"Sesn1",4.0308335322598e-29,0.417949505898714,0.437,0.108,6.79679150209647e-25,"4","Sesn1"
"Sdc42",5.28787888953047e-29,0.716157421530643,0.861,0.368,8.91642138352627e-25,"4","Sdc4"

"Zfp704",9.5894297218917e-29,0.409270432564341,0.468,0.127,1.616969639
70538e-24,"4","Zfp704"
"Stom",1.52057623336723e-28,0.369590827624458,0.316,0.059,2.5639956447
0383e-24,"4","Stom"
"Rapgef2",2.26532110772799e-28,0.617880827404071,0.5,0.151,3.819784451
85094e-24,"4","Rapgef2"
"Laptm4a",3.68945107075272e-28,0.678637808513832,0.987,0.781,6.2211523
9550324e-24,"4","Laptm4a"
"Ncoa7",4.03991474862134e-28,0.456081540031016,0.367,0.082,6.812104249
1253e-24,"4","Ncoa7"
"Tcf4",4.23958924917629e-28,0.742615269164833,0.943,0.552,7.1487953919
6106e-24,"4","Tcf4"
"Arhgap19",4.43463986229374e-28,0.420464167522932,0.386,0.09,7.4776897
3579971e-24,"4","Arhgap19"
"Gm269171",4.66275834993914e-28,0.552309766796081,0.646,0.239,7.862343
12966738e-24,"4","Gm26917"
"Cd164",6.004661331472e-28,0.72545557614066,0.804,0.393,1.012505993712
81e-23,"4","Cd164"
"Jdp2",1.13136307568588e-27,0.69955723351489,0.494,0.144,1.90770441822
153e-23,"4","Jdp2"
"Ube2l6",1.14097401016698e-27,0.255278625273847,0.424,0.103,1.92391037
594356e-23,"4","Ube2l6"
"Sulf2",2.15182136734279e-27,0.421077113302279,0.399,0.099,3.628401189
61341e-23,"4","Sulf2"
"Lgals3bp",3.14217066177331e-27,0.750193712962612,0.911,0.528,5.298328
16988215e-23,"4","Lgals3bp"
"Lilra5",3.20319937189046e-27,0.402458879478513,0.418,0.104,5.40123478
088169e-23,"4","Lilra5"
"Galc",4.65118929033294e-27,0.353530309701112,0.386,0.093,7.8428353813
594e-23,"4","Galc"
"Xaf1",5.55013976090342e-27,0.489675696268554,0.481,0.136,9.3586456648
3534e-23,"4","Xaf1"
"Ifih1",5.88732326186597e-27,0.661354441906985,0.57,0.189,9.9272044841
5839e-23,"4","Ifih1"
"Clec2d",7.04229874093636e-27,0.850537921256869,0.456,0.132,1.18747241
369669e-22,"4","Clec2d"
"Rasal2",1.94543407779073e-26,0.292292573777338,0.316,0.064,3.28039094
197073e-22,"4","Rasal2"
"Ms4a6d",3.11280292656549e-26,0.786633044250154,0.899,0.554,5.24880829
477473e-22,"4","Ms4a6d"
"Pid1",5.84831614865434e-26,0.537383889280109,0.69,0.266,9.86143068986
094e-22,"4","Pid1"
"Epsti1",6.00886970742064e-26,0.510618042551235,0.791,0.337,1.01321561
006527e-21,"4","Epsti1"
"Rap2b",9.07150769623779e-26,0.716096994433245,0.589,0.225,1.529637627
73962e-21,"4","Rap2b"
"Ccnd1",1.47396143522074e-25,0.633310786500401,0.722,0.311,2.485393772
06921e-21,"4","Ccnd1"
"Milr1",1.4833369519572e-25,0.551650448224161,0.766,0.344,2.5012027683
9023e-21,"4","Milr1"

"Xylt2",2.26349602937796e-25,0.30229119969452,0.367,0.087,3.8167070047
3711e-21,"4","Xylt2"
"Nampt",2.47538147804082e-25,0.496249358059312,0.601,0.21,4.1739882482
7242e-21,"4","Nampt"
"Ninj1",2.88410151507899e-25,0.676522438128491,0.924,0.619,4.863171974
72619e-21,"4","Ninj1"
"Pml",3.30809397899103e-25,0.426948469006257,0.335,0.075,5.57810806737
468e-21,"4","Pml"
"Gpr183",4.35738209987527e-25,1.01825055269901,0.823,0.414,7.347417696
80969e-21,"4","Gpr183"
"Snx8",5.16335143914412e-25,0.405670809243705,0.538,0.176,8.7064431966
8481e-21,"4","Snx8"
"Slc31a1",5.99696659062863e-25,0.522674651033455,0.741,0.314,1.0112085
065118e-20,"4","Slc31a1"
"Filip1l",6.0407626610146e-25,0.878361661884255,0.677,0.312,1.01859339
990028e-20,"4","Filip1l"
"Clic41",9.02372462897177e-25,0.872397588872009,0.823,0.454,1.52158044
693722e-20,"4","Clic4"
"Frmd4b1",1.01398407136709e-24,0.717954469455087,0.892,0.489,1.7097799
4113919e-20,"4","Frmd4b"
"Phf11d",1.14274755177192e-24,0.597869737791998,0.481,0.152,1.92690092
179782e-20,"4","Phf11d"
"Parp14",1.18167190449425e-24,0.911398238100528,0.684,0.303,1.99253516
53582e-20,"4","Parp14"
"Rbpj",1.58030240527138e-24,0.706303174472996,0.835,0.476,2.6647059157
686e-20,"4","Rbpj"
"Isy1",2.81745838811063e-24,0.644749300283132,0.487,0.158,4.7507983340
3215e-20,"4","Isy1"
"Idh2",3.14644169974718e-24,0.689367604269218,0.797,0.42,5.30552999411
37e-20,"4","Idh2"
"Hspa4l",6.85138745211062e-24,0.414473129276558,0.354,0.089,1.15528095
217489e-19,"4","Hspa4l"
"Clec4a3",7.04259010993731e-24,0.29343983363238,0.646,0.241,1.18752154
433763e-19,"4","Clec4a3"
"Gbp3",7.93382558406332e-24,0.264764081625707,0.424,0.116,1.3378016699
8476e-19,"4","Gbp3"
"Plekhg5",1.14161775675437e-23,0.351044557861888,0.31,0.069,1.92499586
143922e-19,"4","Plekhg5"
"Cd36",1.16294112848859e-23,0.307569803813863,0.31,0.067,1.96095133085
745e-19,"4","Cd36"
"H2-
D1",1.62624636747168e-23,0.607470181288285,0.987,0.944,2.7421766248307
5e-19,"4","H2-D1"
"Dusp11",1.85252609053257e-23,0.883639869569095,0.975,0.781,3.12372949
385602e-19,"4","Dusp1"
"Anxa5",1.90805635282606e-23,0.438730480166588,0.861,0.409,3.217364622
1353e-19,"4","Anxa5"
"Stat1",2.83454068377806e-23,0.503696948736756,0.639,0.246,4.779602500
98656e-19,"4","Stat1"
"Tmem176a",7.54931641793571e-23,0.729247627664178,0.937,0.777,1.272965

73439232e-18,"4","Tmem176a"
"Stard4",7.57767330990339e-23,0.29193766922495,0.285,0.06,1.2777472735
1591e-18,"4","Stard4"
"Slc38a6",1.09845541233924e-22,0.294651951611095,0.399,0.112,1.8522155
1628643e-18,"4","Slc38a6"
"P2rx4",1.37662853024591e-22,0.603621466642257,0.886,0.511,2.321271027
70066e-18,"4","P2rx4"
"Herc6",1.4478854534358e-22,0.447974394898182,0.411,0.121,2.4414244515
8345e-18,"4","Herc6"
"Prune2",2.42475342444595e-22,0.407199780645459,0.563,0.205,4.08861922
430075e-18,"4","Prune2"
"Atp6v1a",2.596916746117e-22,0.617250396108338,0.772,0.392,4.378921017
30249e-18,"4","Atp6v1a"
"Sh3bgrl",2.74881589445528e-22,0.3785258739039,0.595,0.214,4.635053361
23049e-18,"4","Sh3bgrl"
"B3galnt1",3.119370573976e-22,0.352754412890923,0.456,0.144,5.25988266
183833e-18,"4","B3galnt1"
"Hdac9",4.1889732683409e-22,0.395689219039095,0.373,0.1,7.063446725076
42e-18,"4","Hdac9"
"Trim30a",4.94180463642305e-22,0.71744731454594,0.785,0.406,8.33287097
793654e-18,"4","Trim30a"
"Ddx58",4.94431862136301e-22,0.400584849450746,0.38,0.104,8.3371100593
4232e-18,"4","Ddx58"
"Abca1",5.21672251482021e-22,0.627008979153016,0.842,0.454,8.796437504
48984e-18,"4","Abca1"
"Wasl",5.36764511270756e-22,0.585835690733897,0.532,0.203,9.0509231890
4749e-18,"4","Wasl"
"Dmac1",7.28175286198228e-22,0.262858272501826,0.5,0.163,1.22784916758
745e-17,"4","Dmac1"
"Parp12",8.55118429818516e-22,0.42317248701667,0.481,0.165,1.441900696
35998e-17,"4","Parp12"
"Nfe2l21",1.12507821587368e-21,0.733545235798509,0.968,0.705,1.8971068
8760619e-17,"4","Nfe2l2"
"Engase",1.41363304409159e-21,0.268076070673777,0.418,0.121,2.38366803
894724e-17,"4","Engase"
"Nr4a31",1.5406931617666e-21,0.880063994202188,0.595,0.257,2.597916809
37084e-17,"4","Nr4a3"
"Ly6e",1.56945208299037e-21,0.701064514098448,0.994,0.893,2.6464101023
3837e-17,"4","Ly6e"
"Dennd1b",1.61348912663775e-21,0.525625757996418,0.487,0.172,2.7206653
6533657e-17,"4","Dennd1b"
"Sh3bp5",2.44221998398409e-21,0.429381968787245,0.525,0.187,4.11807133
699398e-17,"4","Sh3bp5"
"Pde7a",2.70052568981852e-21,0.27949205362699,0.291,0.067,4.5536264181
7199e-17,"4","Pde7a"
"2610507B11Rik",3.41710079681829e-21,0.410066636815645,0.671,0.281,5.7
6191536359501e-17,"4","2610507B11Rik"
"Sult1a1",3.49227476271694e-21,0.411594198088496,0.462,0.154,5.8886737
0489331e-17,"4","Sult1a1"
"Cltc",4.64694465496435e-21,0.716592988630398,0.968,0.769,7.8356780772

0088e-17,"4","Cltc"
"Lacc11",5.64150922949654e-21,0.766900299181322,0.899,0.565,9.51271286
277707e-17,"4","Lacc1"
"Irgm1",7.20993309136897e-21,0.348910436478835,0.418,0.127,1.215738917
86664e-16,"4","Irgm1"
"Il10rb",8.19404279386129e-21,0.622749684767401,0.924,0.59,1.381679495
90089e-16,"4","Il10rb"
"Fgl2",1.21949415423556e-20,0.529985994500802,0.513,0.185,2.0563110428
7199e-16,"4","Fgl2"
"Ahnak",1.27483252184093e-20,0.360331665114153,0.316,0.076,2.149622598
32817e-16,"4","Ahnak"
"Serp1",1.6080449544792e-20,0.64472948984293,0.943,0.702,2.71148540224
283e-16,"4","Serp1"
"Fnip1",2.63901914977462e-20,0.702630726430476,0.797,0.424,4.449914090
34996e-16,"4","Fnip1"
"Rnf141",2.7157949618022e-20,0.289400715464747,0.342,0.092,4.579373464
59087e-16,"4","Rnf141"
"Nisch",3.66241958375771e-20,0.565277185358636,0.816,0.439,6.175571902
13225e-16,"4","Nisch"
"Crem",4.19045110992889e-20,0.424709444864194,0.519,0.195,7.0659386615
6209e-16,"4","Crem"
"Hsd17b12",4.72047220047751e-20,0.525599583959367,0.835,0.466,7.959660
22444518e-16,"4","Hsd17b12"
"Apoc1",4.76808856461114e-20,0.664605473627787,0.437,0.143,8.039950937
6473e-16,"4","Apoc1"
"Aldh2",6.11810300266652e-20,0.405387321515731,0.861,0.488,1.031634528
30963e-15,"4","Aldh2"
"Fam46a1",6.56883133120246e-20,0.799603671107117,0.772,0.384,1.1076363
3906736e-15,"4","Fam46a"
"H2-
T23",6.70531314608403e-20,0.583888959103599,0.873,0.531,1.130649902692
69e-15,"4","H2-T23"
"Slfn21",7.20054135414328e-20,0.715587750556889,0.943,0.671,1.21415528
313564e-15,"4","Slfn2"
"Ctsb",8.12125703195736e-20,0.45229463898202,0.994,0.973,1.36940636072
865e-15,"4","Ctsb"
"Phf11b",1.09835615036735e-19,0.461468457237333,0.532,0.204,1.85204814
074943e-15,"4","Phf11b"
"Ier32",1.16336762916265e-19,0.856484742771542,0.987,0.79,1.9616704962
9406e-15,"4","Ier3"
"Cysltr1",1.2826198788116e-19,0.418993271062018,0.703,0.318,2.16275363
965212e-15,"4","Cysltr1"
"Hlx",1.29459382893765e-19,0.359117310801721,0.411,0.13,2.182944114354
67e-15,"4","Hlx"
"Agtrap",1.69530862672866e-19,0.268560701404138,0.361,0.103,2.85862940
638987e-15,"4","Agtrap"
"Eea1",1.71301615520353e-19,0.454713288695655,0.671,0.297,2.8884878409
0419e-15,"4","Eea1"
"Fam234a",1.87052277851081e-19,0.283767295621155,0.475,0.161,3.1540755
0912492e-15,"4","Fam234a"

"Egr21",2.46352726563663e-19,0.92810392680624,0.854,0.521,4.15399967531648e-15,"4","Egr2"
"Zfp36l12",2.68768712740343e-19,0.889016585666516,0.949,0.708,4.53197803422767e-15,"4","Zfp36l1"
"Klf41",4.72448328314596e-19,0.838088993555957,0.728,0.383,7.96642371204072e-15,"4","Klf4"
"Ocrl",5.08534263874317e-19,0.329935837653794,0.291,0.074,8.57490475744873e-15,"4","Ocrl"
"Axl",7.00421530141492e-19,0.552306752300105,0.747,0.379,1.18105078412458e-14,"4","Axl"
"Prdm11",8.45191750726183e-19,0.488024898852685,0.538,0.209,1.42516233007449e-14,"4","Prdm1"
"Gm265321",1.25566453345196e-18,0.806394574399546,0.747,0.406,2.11730153630669e-14,"4","Gm26532"
"Fads1",1.2640576298698e-18,0.291784668996238,0.405,0.13,2.13145397548646e-14,"4","Fads1"
"Fam213b",1.45102323092087e-18,0.272209449496554,0.506,0.185,2.44671537197878e-14,"4","Fam213b"
"Phactr2",2.37991361417869e-18,0.282169724683059,0.373,0.114,4.01301033622811e-14,"4","Phactr2"
"Fh1",2.89228855391561e-18,0.404598084124667,0.5,0.197,4.87697695961251e-14,"4","Fh1"
"C3ar11",2.91693532960977e-18,0.589456390232826,0.981,0.716,4.918536352788e-14,"4","C3ar1"
"Scpep1",2.91873207556557e-18,0.421163621892933,0.741,0.358,4.92156602581867e-14,"4","Scpep1"
"Cd141",4.40637373565757e-18,0.78875388285121,0.981,0.828,7.43002739306579e-14,"4","Cd14"
"Adrb21",5.8559061969476e-18,0.732589987886806,0.728,0.396,9.87422902929304e-14,"4","Adrb2"
"Zdhhc14",6.65727983022927e-18,0.346079453477569,0.405,0.135,1.12255052497326e-13,"4","Zdhhc14"
"Trf",7.41414748756318e-18,0.691916276217709,0.987,0.91,1.2501735493529e-13,"4","Trf"
"Dusp22",7.70037559437322e-18,0.260003200900787,0.487,0.176,1.29843733272321e-13,"4","Dusp22"
"Ccl121",9.22689944601233e-18,1.24221430803957,0.905,0.674,1.5558397845866e-13,"4","Ccl12"
"Rgs11",1.03136041842138e-17,1.08428195709357,0.646,0.318,1.73907993754213e-13,"4","Rgs1"
"Gbp2",1.32617438445801e-17,0.279993522280074,0.316,0.088,2.23619524707309e-13,"4","Gbp2"
"Lmna1",1.37881375044971e-17,0.541969787464671,0.57,0.233,2.32495574600831e-13,"4","Lmna"
"Spred1",1.50969325468348e-17,0.4377539426863,0.658,0.304,2.54564476604728e-13,"4","Spred1"
"Dbi",1.654153676025e-17,0.435068381441551,0.797,0.441,2.78923392851335e-13,"4","Dbi"
"Cd47",1.65990344747761e-17,0.46162677164586,0.899,0.566,2.79892919313675e-13,"4","Cd47"

"Dnajc3",1.70119603608641e-17,0.552413615907448,0.848,0.546,2.86855675604891e-13,"4","Dnajc3"
"Kansl1l1",1.8014780910702e-17,0.617281313935601,0.741,0.397,3.03765235716258e-13,"4","Kansl1l"
"Trib11",2.11468202486482e-17,0.792317705166425,0.899,0.582,3.56577683032706e-13,"4","Trib1"
"Osbpl9",2.11507084166552e-17,0.54944914836664,0.734,0.408,3.5664324532164e-13,"4","Osbpl9"
"Plau1",2.36604012048152e-17,0.503451549116117,0.487,0.187,3.98961685115594e-13,"4","Plau"
"Hs2st1",2.41929683918064e-17,0.351944590439347,0.475,0.177,4.07941833022639e-13,"4","Hs2st1"
"App",2.45713694223735e-17,0.536473142416905,0.797,0.48,4.14322431200062e-13,"4","App"
"Adam9",2.55547380518164e-17,0.419371062576152,0.563,0.242,4.30903993029729e-13,"4","Adam9"
"Il1b2",3.01959737505792e-17,0.907741211346927,0.886,0.538,5.09164509382267e-13,"4","Il1b"
"Creb51",3.43221862128938e-17,0.422812247772698,0.525,0.212,5.78740703921816e-13,"4","Creb5"
"Apaf1",3.68257617737382e-17,0.319760999664721,0.392,0.13,6.20955995028773e-13,"4","Apaf1"
"Lrrc25",3.7566605564888e-17,0.517956338974658,0.88,0.565,6.33448103035141e-13,"4","Lrrc25"
"Tln2",4.27804789801365e-17,0.489863426176319,0.646,0.306,7.21364436563061e-13,"4","Tln2"
"Gnpda1",4.98547965240554e-17,0.394030847735114,0.601,0.271,8.40651578988622e-13,"4","Gnpda1"
"Sptbn1",5.04091523580038e-17,0.311264863262086,0.418,0.145,8.49999127060661e-13,"4","Sptbn1"
"Rnf130",5.5220583169034e-17,0.532730876749338,0.962,0.785,9.31129473396252e-13,"4","Rnf130"
"Fos1",6.51744030431244e-17,0.735271404322329,0.987,0.843,1.09897078411316e-12,"4","Fos"
"Nrros",6.90507821753906e-17,0.49848369801266,0.842,0.486,1.16433428904144e-12,"4","Nrros"
"Lilr4b",9.59992558133743e-17,0.258908386201353,0.373,0.12,1.61873945152512e-12,"4","Lilr4b"
"Rbfa",1.13890051339753e-16,0.396971555769718,0.665,0.33,1.92041404569091e-12,"4","Rbfa"
"Wtip",1.17692253743628e-16,0.330652846595673,0.544,0.223,1.98452678262505e-12,"4","Wtip"
"Phf20",1.17845453051973e-16,0.313695496842244,0.614,0.258,1.98711002936237e-12,"4","Phf20"
"H2-T22",1.21506793042897e-16,0.33493430517754,0.563,0.238,2.04884754428932e-12,"4","H2-T22"
"Idh1",1.31727731831157e-16,0.272259540410053,0.487,0.189,2.22119301413696e-12,"4","Idh1"
"H2-

M3",1.46215424121331e-16,0.36077835630634,0.608,0.273,2.46548448153389e-12,"4","H2-M3"
"Gatm",1.57866548335376e-16,0.507028600442435,0.949,0.696,2.66194573803111e-12,"4","Gatm"
"Man1a",1.73273670982042e-16,0.646643058993485,0.785,0.454,2.92174064009919e-12,"4","Man1a"
"C5ar1",2.12321482773017e-16,0.679546086433326,0.949,0.702,3.58016484251861e-12,"4","C5ar1"
"Etv1",2.18074356415331e-16,0.286187283597714,0.354,0.114,3.67716979787532e-12,"4","Etv1"
"Rgs21",3.56517559189827e-16,0.735705588658597,0.785,0.507,6.01159908305887e-12,"4","Rgs2"
"Fnbp1",3.82905036123328e-16,0.512693497119187,0.766,0.421,6.45654471911155e-12,"4","Fnbp1"
"Psmb9",4.88891981894603e-16,0.324643182456447,0.709,0.35,8.2436965987068e-12,"4","Psmb9"
"Gpr160",4.98902824027492e-16,0.293903873266506,0.38,0.127,8.41249941875156e-12,"4","Gpr160"
"Cited21",4.99060063424551e-16,1.46319548319683,0.88,0.614,8.41515078946478e-12,"4","Cited2"
"Tmem37",5.11046620379361e-16,0.413904119440047,0.873,0.532,8.61726811283678e-12,"4","Tmem37"
"Sp140",5.49929192981045e-16,0.336826954189844,0.544,0.226,9.27290605204639e-12,"4","Sp140"
"Eif2ak2",5.73878590970725e-16,0.439198501003069,0.62,0.285,9.67674080094837e-12,"4","Eif2ak2"
"Asah1",6.36383443171321e-16,0.426452301956157,0.987,0.841,1.07306976187548e-11,"4","Asah1"
"Fgd4",6.43646920329522e-16,0.311810590271286,0.506,0.204,1.08531743705964e-11,"4","Fgd4"
"Klhl18",6.89556338758375e-16,0.315781152979153,0.443,0.168,1.16272989841437e-11,"4","Klhl18"
"Clta1",9.4644805007933e-16,0.42216781784051,0.994,0.957,1.59590070204377e-11,"4","Clta"
"Dera",9.67058184569503e-16,0.262670591462676,0.481,0.189,1.6306535108211e-11,"4","Dera"
"Man2a1",1.07162687231336e-15,0.256243283141905,0.373,0.127,1.80697723209479e-11,"4","Man2a1"
"Hmgcl",1.40790100834736e-15,0.305934405312876,0.703,0.337,2.37400268027531e-11,"4","Hmgcl"
"Myo1c",1.43801055391621e-15,0.513971961916906,0.589,0.269,2.42477339601351e-11,"4","Myo1c"
"Slfn8",1.67222734050045e-15,0.399127147727396,0.519,0.215,2.81970974155187e-11,"4","Slfn8"
"Ing2",1.67540390153401e-15,0.406234735317451,0.563,0.24,2.82506605876665e-11,"4","Ing2"
"Mfsd1",1.67863976260987e-15,0.458135241071183,0.722,0.388,2.83052236771276e-11,"4","Mfsd1"
"Egr32",2.6515836129154e-15,0.482309809849934,0.715,0.363,4.47110028809794e-11,"4","Egr3"

"Fam174a",2.69140423469625e-15,0.269124500491076,0.766,0.406,4.53824582054482e-11,"4","Fam174a"
"Tm2d2",5.36922439818619e-15,0.445001925392211,0.829,0.508,9.05358618022155e-11,"4","Tm2d2"
"Mctp1",5.73802543876949e-15,0.301523172897112,0.31,0.099,9.67545849485311e-11,"4","Mctp1"
"Gdap10",6.34080596321461e-15,0.255108436693119,0.259,0.072,1.06918670151725e-10,"4","Gdap10"
"Nfkbid1",6.69170459383801e-15,0.662227455157636,0.975,0.679,1.12835522861297e-10,"4","Nfkbid"
"Idi1",6.98511176409092e-15,0.37737549303164,0.329,0.107,1.17782954566101e-10,"4","Idi1"
"Crtc3",7.07586312981783e-15,0.340633389225444,0.43,0.163,1.19313204094988e-10,"4","Crtc3"
"Plin2",7.96507307859242e-15,0.606889954112165,0.791,0.45,1.34307062251225e-10,"4","Plin2"
"Ptgs21",8.04995932484781e-15,0.644543085751061,0.373,0.135,1.35738414135584e-10,"4","Ptgs2"
"Lbr",8.78854355350386e-15,0.252402341213936,0.38,0.136,1.48192421399182e-10,"4","Lbr"
"Rab11fip5",8.83717919731512e-15,0.352266780930693,0.595,0.273,1.49012515625128e-10,"4","Rab11fip5"
"Dennd1a",1.08250958500844e-14,0.265020994498531,0.348,0.113,1.82532766224124e-10,"4","Dennd1a"
"Ctnnd1",1.1980310729873e-14,0.295763075924164,0.43,0.17,2.02011999527119e-10,"4","Ctnnd1"
"Slc43a2",1.57829705465046e-14,0.367201833351961,0.639,0.33,2.66132449355161e-10,"4","Slc43a2"
"Mfsd11",1.6449437209568e-14,0.345300201861657,0.576,0.266,2.77370410227735e-10,"4","Mfsd11"
"Sdcbp",1.70836783980099e-14,0.457218063308528,0.981,0.862,2.88064985147243e-10,"4","Sdcbp"
"Tnfrsf11a",1.90948284700825e-14,0.438809917490441,0.646,0.316,3.21976997662531e-10,"4","Tnfrsf11a"
"Trafd1",2.32430242228088e-14,0.509614921788951,0.766,0.451,3.91923874445002e-10,"4","Trafd1"
"Hfe",2.42910057382743e-14,0.426601410965579,0.778,0.45,4.09594938758781e-10,"4","Hfe"
"Crlf2",2.43400585123615e-14,0.398572988561125,0.797,0.444,4.10422066635439e-10,"4","Crlf2"
"B4galt6",2.4992716985344e-14,0.344621961515464,0.443,0.171,4.2142719380687e-10,"4","B4galt6"
"Klf21",2.61632255373878e-14,0.94151363821516,0.867,0.646,4.41164309011434e-10,"4","Klf2"
"Dhx58",3.19600725327496e-14,0.266943909036807,0.335,0.116,5.38910743047223e-10,"4","Dhx58"
"Zcchc11",3.3932123719306e-14,0.37116929842023,0.57,0.27,5.72163470154938e-10,"4","Zcchc11"
"Ggta1",3.61032086048507e-14,0.278524965924237,0.481,0.201,6.08772303494992e-10,"4","Ggta1"

"Itgav1",3.64194511258173e-14,0.419570302450875,0.734,0.409,6.14104784
883532e-10,"4","Itgav"
"Ldlr",4.21833198372705e-14,0.387587460814519,0.361,0.13,7.11295139096
055e-10,"4","Ldlr"
"Zfp703",4.91079398512953e-14,0.343628205500225,0.551,0.25,8.280580817
72541e-10,"4","Zfp703"
"H2-
Q6",4.9905351058148e-14,0.515632057899643,0.437,0.182,8.41504029542492
e-10,"4","H2-Q6"
"Zfp281",5.67131178441833e-14,0.250743090658051,0.354,0.124,9.56296593
088619e-10,"4","Zfp281"
"Apobec1",5.82529135088479e-14,0.431404957744989,0.525,0.243,9.8226062
7586192e-10,"4","Apobec1"
"Tpr",5.84288513664552e-14,0.473595133337362,0.918,0.695,9.85227291741
168e-10,"4","Tpr"
"Runx11",6.05454564908176e-14,0.57827121270624,0.924,0.665,1.020917487
34817e-09,"4","Runx1"
"Acat1",6.2608681157068e-14,0.293721190901864,0.646,0.315,1.0557075816
7048e-09,"4","Acat1"
"Nr4a11",6.94924950053418e-14,0.639702040278063,0.892,0.572,1.17178245
078007e-09,"4","Nr4a1"
"Igtp",7.02514746004549e-14,0.319990109299063,0.418,0.163,1.1845803647
1287e-09,"4","Igtp"
"Mrpl3",7.30802572278643e-14,0.264709627658397,0.481,0.207,1.232279297
37625e-09,"4","Mrpl3"
"Dusp61",7.83842320659358e-14,0.510621441958163,0.677,0.358,1.32171492
109581e-09,"4","Dusp6"
"Specc1l",8.27778478315856e-14,0.259377854518589,0.43,0.168,1.39580007
01362e-09,"4","Specc1l"
"Lamtor3",8.74325435224874e-14,0.303254167476834,0.759,0.412,1.4742875
4887618e-09,"4","Lamtor3"
"Synj1",8.99538709648239e-14,0.395923604916893,0.646,0.338,1.516802172
20886e-09,"4","Synj1"
"Gadd45g1",9.53054145198084e-14,0.467937026313142,0.418,0.168,1.607039
89963301e-09,"4","Gadd45g"
"Ap2s1",1.01947282395653e-13,0.386335798154067,0.766,0.455,1.719035075
75549e-09,"4","Ap2s1"
"Fcgr1",1.12460049334981e-13,0.43911606864508,0.975,0.811,1.8963013518
8646e-09,"4","Fcgr1"
"Sap30",1.17704534535009e-13,0.318278845917932,0.506,0.22,1.9847338613
2932e-09,"4","Sap30"
"Nudt16",1.26424286852486e-13,0.267324677891913,0.335,0.118,2.13176632
490661e-09,"4","Nudt16"
"Slc40a1",1.64747152790462e-13,0.271005538326287,0.582,0.277,2.7779664
9035277e-09,"4","Slc40a1"
"Msmo1",2.33850228814577e-13,0.507090564082037,0.468,0.208,3.943182558
2714e-09,"4","Msmo1"
"Uvrag",2.42936214100741e-13,0.43483535985145,0.671,0.356,4.0963904421
6670e-09,"4","Uvrag"
"Rnf145",2.47469959275366e-13,0.303545113858283,0.449,0.185,4.17283845

330122e-09,"4","Rnf145"
"Fbxo3",2.49831497620405e-13,0.275999875884415,0.424,0.168,4.212658712
87527e-09,"4","Fbxo3"
"Cdc42se2",2.5219695915979e-13,0.261263278951012,0.652,0.315,4.2525451
2535238e-09,"4","Cdc42se2"
"Stat2",2.62955262487436e-13,0.266146939533071,0.329,0.116,4.433951636
06314e-09,"4","Stat2"
"Tbxas1",2.76890368631418e-13,0.408037182641585,0.924,0.659,4.66892539
586297e-09,"4","Tbxas1"
"Snap23",2.9518740403721e-13,0.322015386539598,0.703,0.384,4.977450006
87543e-09,"4","Snap23"
"Dcxr",3.80948022471183e-13,0.255170688621147,0.608,0.297,6.4235455549
0908e-09,"4","Dcxr"
"Vwa5a",3.96077822273784e-13,0.301550489805564,0.589,0.279,6.678664239
18055e-09,"4","Vwa5a"
"Il1rn",4.00095986996951e-13,0.527834601175676,0.31,0.107,6.7464185327
4260e-09,"4","Il1rn"
"Tspan31",4.80641183744483e-13,0.278966831861824,0.722,0.395,8.1045716
4029948e-09,"4","Tspan3"
"Csf2rb",5.72898331495875e-13,0.511144033299548,0.766,0.43,9.660211665
68344e-09,"4","Csf2rb"
"Fcho2",6.79637701763041e-13,0.442421902334002,0.785,0.49,1.1460050927
1284e-08,"4","Fcho2"
"Cmklr1",7.60748557243399e-13,0.274770369495687,0.627,0.299,1.28277421
722382e-08,"4","Cmklr1"
"Rapgef6",9.37554566797931e-13,0.39725056758356,0.722,0.393,1.58090451
053467e-08,"4","Rapgef6"
"Ammecr1l",9.45170087340953e-13,0.253800799703938,0.361,0.134,1.593745
80127432e-08,"4","Ammecr1l"
"Agpat3",1.0131795116681e-12,0.369860679122562,0.741,0.43,1.7084232925
7475e-08,"4","Agpat3"
"Arpc3",1.01563480022729e-12,0.366371126082608,0.987,0.903,1.712563400
14325e-08,"4","Arpc3"
"AW112010",1.15495277691863e-12,0.323260133925967,0.323,0.116,1.947481
3724402e-08,"4","AW112010"
"Tmem2511",1.1602963252267e-12,0.263975064127173,0.658,0.325,1.9564916
6359725e-08,"4","Tmem251"
"H2-
Q7",1.17433270103134e-12,0.357990704623994,0.696,0.384,1.9801598004790
5e-08,"4","H2-Q7"
"Wdfy3",1.23518075002271e-12,0.357018145604473,0.595,0.298,2.082761780
6883e-08,"4","Wdfy3"
"Dnajc13",1.23529580901245e-12,0.328015576990907,0.62,0.313,2.08295579
315679e-08,"4","Dnajc13"
"Snx9",1.3608346078336e-12,0.317038406926863,0.538,0.262,2.29463931572
902e-08,"4","Snx9"
"Fndc3a",1.57607185576678e-12,0.272046374525809,0.601,0.288,2.65757236
319394e-08,"4","Fndc3a"
"Grn",1.57836781887355e-12,0.377553872534461,0.994,0.937,2.66144381618
459e-08,"4","Grn"

"Nmd3",1.77168636972062e-12,0.30272238342012,0.481,0.217,2.98741755662291e-08,"4","Nmd3"
"Ptafr",2.53375327706906e-12,0.477562662432568,0.734,0.431,4.27241477579386e-08,"4","Ptafr"
"Tmbim1",2.68393060904192e-12,0.279509742271937,0.525,0.246,4.52564379296649e-08,"4","Tmbim1"
"Tet2",2.83580494656409e-12,0.496322973954593,0.576,0.298,4.78173430089637e-08,"4","Tet2"
"Lipa",2.96510869984166e-12,0.315873146363681,0.804,0.466,4.999766289673e-08,"4","Lipa"
"Rabgef11",3.06609524354688e-12,0.363275944622768,0.722,0.391,5.17004979966875e-08,"4","Rabgef1"
"Sp110",3.22340797723903e-12,0.274906187482213,0.595,0.296,5.43531053122046e-08,"4","Sp110"
"Spry11",3.69366517257562e-12,0.449394959426403,0.291,0.102,6.228258213997e-08,"4","Spry1"
"Srgn1",3.74937805951635e-12,0.399729058169699,0.962,0.845,6.32220128395648e-08,"4","Srgn"
"Mapkapk2",5.20367529805103e-12,0.568190312095088,0.918,0.709,8.77443728757365e-08,"4","Mapkapk2"
"Txnip",5.88849335076233e-12,0.748138384505145,0.62,0.365,9.92917748805544e-08,"4","Txnip"
"Wwp2",6.08294382389773e-12,0.301252253222579,0.456,0.205,1.02570598758564e-07,"4","Wwp2"
"Snx3",6.35327227795317e-12,0.448167766916067,0.918,0.747,1.07128877150846e-07,"4","Snx3"
"Irf2bp21",7.05110864076392e-12,0.415671469564241,0.924,0.625,1.18895793900561e-07,"4","Irf2bp2"
"Ankrd12",7.51207732137882e-12,0.253701337487856,0.614,0.298,1.2666864779309e-07,"4","Ankrd12"
"Osm1",1.00517481569211e-11,0.619800451138445,0.709,0.431,1.69492577422004e-07,"4","Osm"
"Aff1",1.44048858997252e-11,0.293156787073725,0.424,0.18,2.42895186041166e-07,"4","Aff1"
"Max",1.73494808944813e-11,0.298407663651946,0.557,0.271,2.92546946842744e-07,"4","Max"
"Itpkb1",2.05728017077123e-11,0.377138987664564,0.671,0.371,3.46898582395445e-07,"4","Itpkb"
"Ppp1r9a1",3.3213463100308e-11,0.275913368208901,0.646,0.347,5.60045414797393e-07,"4","Ppp1r9a"
"Selenok",3.47416932986178e-11,0.334845204888734,0.956,0.797,5.85814432401294e-07,"4","Selenok"
"Tet3",3.75364699286613e-11,0.359169793766939,0.684,0.364,6.32939955937087e-07,"4","Tet3"
"Plek1",4.73366090520339e-11,0.47965037358245,0.981,0.83,7.98189901835396e-07,"4","Plek"
"Atxn7",4.84069759964071e-11,0.303730641069815,0.449,0.208,8.16238429251416e-07,"4","Atxn7"
"Znfx1",4.89529523710674e-11,0.376564705382802,0.462,0.219,8.25444682880938e-07,"4","Znfx1"

"Dmxl1",5.09966979297371e-11,0.254591638123655,0.506,0.238,8.59906320491227e-07,"4","Dmxl1"
"Papd4",5.1617018344967e-11,0.286151168381079,0.582,0.296,8.70366163332833e-07,"4","Papd4"
"Arid5b",5.95586437427304e-11,0.380922309756599,0.557,0.3,1.00427785078992e-06,"4","Arid5b"
"Zfp263",7.10034494596226e-11,0.253003249460964,0.411,0.18,1.19726016478816e-06,"4","Zfp263"
"Hspa8",7.33409959821163e-11,0.330291862664759,0.994,0.975,1.23667587425045e-06,"4","Hspa8"
"Lima1",7.83468905169637e-11,0.341989980272988,0.437,0.203,1.32108526789704e-06,"4","Lima1"
"Cebpb",9.76536066203511e-11,0.392821611425078,0.975,0.86,1.64663511483236e-06,"4","Cebpb"
"Atp6v1h",1.00742348749731e-10,0.299746102534845,0.538,0.267,1.69871748461797e-06,"4","Atp6v1h"
"Neu11",1.00963850786487e-10,0.262762759273431,0.785,0.466,1.70245245196174e-06,"4","Neu1"
"Cxcr4",1.07658244443848e-10,0.264808390511162,0.272,0.095,1.81533331781217e-06,"4","Cxcr4"
"Tcp11l2",1.30866267094403e-10,0.283928178414743,0.361,0.156,2.20666699574582e-06,"4","Tcp11l2"
"Lyz2",1.36317406178441e-10,0.864264890473333,0.57,0.365,2.29858410298087e-06,"4","Lyz2"
"Peak1",1.38077840722723e-10,0.318873730808544,0.348,0.143,2.32826855026656e-06,"4","Peak1"
"Exoc3",1.39043476118517e-10,0.259898114193002,0.595,0.318,2.34455109431043e-06,"4","Exoc3"
"Tpp1",1.42101328848771e-10,0.350217286528176,0.905,0.641,2.39611260704798e-06,"4","Tpp1"
"Rpn1",1.64453507381371e-10,0.336696827333867,0.671,0.396,2.77301504146468e-06,"4","Rpn1"
"Ralbp1",1.94271022707319e-10,0.300347175263058,0.703,0.395,3.27579798489081e-06,"4","Ralbp1"
"Rasa1",2.0100863184283e-10,0.300555883452519,0.57,0.298,3.3894075501338e-06,"4","Rasa1"
"Neat11",2.2642041955124e-10,0.491602776172303,0.962,0.747,3.81790111447301e-06,"4","Neat1"
"Pvr",2.51974506949115e-10,0.250402529192283,0.424,0.188,4.24879413617598e-06,"4","Pvr"
"Sptlc2",2.59341339915792e-10,0.324150094322451,0.753,0.446,4.37301367366009e-06,"4","Sptlc2"
"Csrnp13",2.70402946082715e-10,0.458411736471794,0.93,0.667,4.55953447684674e-06,"4","Csrnp1"
"Sft2d2",2.98014559086487e-10,0.2578674213918,0.544,0.276,5.02512149531635e-06,"4","Sft2d2"
"Galnt1",3.34372229905256e-10,0.30842046920492,0.753,0.439,5.63818454066243e-06,"4","Galnt1"
"Hexa1",3.97895873388266e-10,0.327138757093921,0.987,0.9,6.70932021707294e-06,"4","Hexa"

"Lman2",4.07941785402001e-10,0.304609485833881,0.772,0.5,6.87871438544854e-06,"4","Lman2"
"Tlr7",4.56701546838062e-10,0.31273415015428,0.791,0.505,7.7009014827834e-06,"4","Tlr7"
"Slc25a28",4.59518373068294e-10,0.366027104067751,0.411,0.187,7.74839880667757e-06,"4","Slc25a28"
"Dync1h1",4.95422807775656e-10,0.304031830900579,0.709,0.394,8.35381938471311e-06,"4","Dync1h1"
"Cxcl16",5.03922530628631e-10,0.520875134570447,0.753,0.529,8.49714171145998e-06,"4","Cxcl16"
"Lamp11",6.26065204669327e-10,0.324444112331333,0.994,0.956,1.05567114811342e-05,"4","Lamp1"
"Akap9",6.66149347100195e-10,0.301544693900123,0.671,0.372,1.12326102908035e-05,"4","Akap9"
"Hccs",7.0367202246274e-10,0.307116921408333,0.456,0.221,1.18653176427667e-05,"4","Hccs"
"Ap3d1",7.37195647022222e-10,0.266540607940839,0.551,0.278,1.24305930000887e-05,"4","Ap3d1"
"Snx5",8.14147513669298e-10,0.329589487799449,0.981,0.801,1.37281553754917e-05,"4","Snx5"
"Ebi3",8.78427040296576e-10,0.262593157043388,0.563,0.299,1.48120367534809e-05,"4","Ebi3"
"Gna13",8.88828800617633e-10,0.40380503603212,0.797,0.552,1.49874312360145e-05,"4","Gna13"
"Marcksl13",9.22972306638469e-10,0.404865764776849,0.943,0.717,1.55631590345379e-05,"4","Marcksl1"
"Il4ra1",9.57294943496624e-10,0.386218315110382,0.816,0.514,1.61419073372401e-05,"4","Il4ra"
"Use1",1.08083268603064e-09,0.26027417929795,0.791,0.538,1.82250007518487e-05,"4","Use1"
"Mgat11",1.34151333609832e-09,0.284276852609511,0.696,0.392,2.262059787329e-05,"4","Mgat1"
"Tm6sf1",1.43907890472384e-09,0.508940721677616,0.848,0.632,2.42657484914534e-05,"4","Tm6sf1"
"Pim3",1.54319596768324e-09,0.355777706131651,0.538,0.281,2.60213704070748e-05,"4","Pim3"
"Hmgcs1",1.60115110486067e-09,0.25455418912064,0.462,0.221,2.69986099301606e-05,"4","Hmgcs1"
"Atxn7l3b",1.76896681930083e-09,0.268041441907529,0.69,0.397,2.98283185070506e-05,"4","Atxn7l3b"
"Slc6a6",1.96707803911482e-09,0.323231871336765,0.892,0.653,3.31688698955542e-05,"4","Slc6a6"
"Cbl",2.1320980080253e-09,0.365048839400694,0.753,0.483,3.59514366113226e-05,"4","Cbl"
"Picalm1",2.29752944012659e-09,0.465086215546077,0.981,0.791,3.87409414194146e-05,"4","Picalm"
"Npl",2.44857365514511e-09,0.444886620070049,0.614,0.404,4.12878489730568e-05,"4","Npl"
"Dot1l1",3.05828112730539e-09,0.358719027313635,0.658,0.372,5.15687363686235e-05,"4","Dot1l"

"P2ry61",3.64008805568717e-09,0.307385706923746,0.892,0.625,6.13791647949971e-05,"4","P2ry6"
"Tagap2",5.72387468402411e-09,0.336007301643844,0.753,0.49,9.65159749220146e-05,"4","Tagap"
"Birc3",5.97844874957646e-09,0.352988746255715,0.759,0.507,0.000100808602815358,"4","Birc3"
"Zfand51",6.72258195823688e-09,0.430581649810787,0.968,0.758,0.00011335617697979,"4","Zfand5"
"Insig1",9.08677620542989e-09,0.374967877684194,0.449,0.236,0.000153221220375959,"4","Insig1"
"Tor3a",1.0333206912776e-08,0.290987720311579,0.551,0.304,0.00017423853496323,"4","Tor3a"
"Nr3c1",1.37379619443229e-08,0.306190607560161,0.81,0.521,0.000231649514305172,"4","Nr3c1"
"Srxn11",1.65283795053956e-08,0.377726336369198,0.563,0.316,0.000278701535219981,"4","Srxn1"
"Pdia6",1.68178377922089e-08,0.386963287210391,0.918,0.696,0.000283582380852226,"4","Pdia6"
"Gnaq1",1.91193310002807e-08,0.317903335565521,0.684,0.412,0.000322390159326732,"4","Gnaq"
"Wdfy2",1.9367676017295e-08,0.312025920306935,0.43,0.217,0.000326577753003629,"4","Wdfy2"
"H2-
Q41",2.23271787847566e-08,0.398619454120461,0.608,0.348,0.000376480888668565,"4","H2-Q4"
"Zfand6",2.28616602741555e-08,0.273053349603857,0.715,0.451,0.00038549331554281,"4","Zfand6"
"Sav1",2.40090716288059e-08,0.283055927923763,0.443,0.232,0.000404840965804925,"4","Sav1"
"5430427O19Rik1",3.24747835062522e-08,0.2526114485381,0.462,0.241,0.000547589799482425,"4","5430427O19Rik"
"Phlda11",3.48271922887431e-08,0.409142790305276,0.608,0.357,0.000587256116372786,"4","Phlda1"
"Ccl32",3.503480072952e-08,0.41202331355922,0.981,0.778,0.000590756809901167,"4","Ccl3"
"Pdia3",3.81707030824202e-08,0.355850052986987,0.968,0.842,0.000643634395375769,"4","Pdia3"
"Rab201",5.0875561189825e-08,0.33136467076913,0.759,0.496,0.000857863712782829,"4","Rab20"
"5031425E22Rik",6.56594983033862e-08,0.25207998274557,0.424,0.215,0.0011071504603917,"4","5031425E22Rik"
"Dhrs3",8.6529249912873e-08,0.283868452071321,0.797,0.535,0.00145905621203086,"4","Dhrs3"
"Bbx",9.20565594702891e-08,0.253184840472234,0.456,0.249,0.00155225770578801,"4","Bbx"
"Fcgr3",1.76469950864336e-07,0.302052405267527,0.981,0.923,0.00297563631147444,"4","Fcgr3"
"Jund1",1.95850090131452e-07,0.346381682407603,0.994,0.894,0.00330242421979654,"4","Jund"
"Ankrd111",1.99871509735058e-07,0.349685265544412,0.93,0.693,0.0033702

3339715254,"4","Ankrd11"
"Aftph",2.1835143206253e-07,0.265048997341957,0.614,0.372,0.0036818418
4743838,"4","Aftph"
"Ubl31",2.2340415500958e-07,0.346231810780412,0.873,0.68,0.00376704086
177154,"4","Ubl3"
"Cd74",2.62391267459101e-07,1.71774994709047,0.665,0.455,0.00442444155
189537,"4","Cd74"
"Klf71",2.67300381193834e-07,0.362623048616847,0.696,0.428,0.004507219
02769044,"4","Klf7"
"Fcer1g",5.02448702855891e-07,0.268033657255077,0.994,0.964,0.00847229
002755603,"4","Fcer1g"
"Odc1",8.15540146535645e-07,0.691819855718729,0.418,0.249,0.0137516379
50884,"4","Odc1"
"Ifrd11",9.73385759078523e-07,0.325116405874649,0.956,0.747,0.01641323
06695821,"4","Ifrd1"
"Fli11",1.16451283152896e-06,0.261991020835297,0.734,0.49,0.0196360153
652413,"4","Fli1"
"Zeb22",1.2618092243098e-06,0.37571198319845,0.956,0.779,0.02127662714
03118,"4","Zeb2"
"Kctd123",1.55457762097085e-06,0.331166224560732,0.987,0.829,0.0262132
878448104,"4","Kctd12"
"Mt11",1.69983107525595e-06,0.384143324996663,0.81,0.581,0.02866255159
09659,"4","Mt1"
"Pmp22",1.98269470592951e-06,0.315353687838312,0.772,0.545,0.033432198
1313834,"4","Pmp22"
"Ccnt11",2.47334136727785e-06,0.294413181928255,0.69,0.447,0.041705482
1350391,"4","Ccnt1"
"Litaf",2.63026497090145e-06,0.275356788547662,0.918,0.723,0.044351527
9393402,"4","Litaf"
"Zfp516",3.04542618775712e-06,0.30916906872432,0.329,0.172,0.051351976
3779606,"4","Zfp516"
"Nfkbil1",3.19564275415769e-06,0.28277120102994,0.399,0.216,0.05388492
8120607,"4","Nfkbil1"
"Nfat52",3.40696851226216e-06,0.301043238468698,0.684,0.451,0.05744830
30537646,"4","Nfat5"
"Zcchc62",3.44193856720808e-06,0.302957518217615,0.873,0.649,0.0580379
681202627,"4","Zcchc6"
"Hspa1a1",4.23495474815454e-06,0.793057324978691,0.519,0.335,0.0714098
069633819,"4","Hspa1a"
"Akr1a11",4.56871646630947e-06,0.265042114896621,0.949,0.853,0.0770376
970549103,"4","Akr1a1"
"Zfp363",4.66631863306757e-06,0.3345531865475,0.994,0.832,0.0786834647
907854,"4","Zfp36"
"Ltc4s1",5.11609132822554e-06,0.322486914745268,0.886,0.664,0.08626753
19765391,"4","Ltc4s"
"Casp42",5.71536888530636e-06,0.280477278352214,0.816,0.573,0.09637255
01440358,"4","Casp4"
"Hsp90b1",5.92070978489557e-06,0.321977390267491,0.956,0.853,0.0998350
083929091,"4","Hsp90b1"
"Zufsp",7.19991550194435e-06,0.303723261565235,0.424,0.246,0.121404975

193786,"4","Zufsp"
"Slc3a2",9.33156859619232e-06,0.25803237953871,0.943,0.786,0.157348909668995,"4","Slc3a2"
"Cd842",1.17033169186784e-05,0.350462101236055,0.861,0.648,0.197341329882755,"4","Cd84"
"Top11",1.85402643769209e-05,0.2646618418638,0.867,0.674,0.31262593792364,"4","Top1"
"Mroh1",4.30461316880326e-05,0.263688101326652,0.335,0.186,0.725843872523605,"4","Mroh1"
"Ripk11",6.28809584800907e-05,0.252511292441643,0.57,0.369,1,"4","Ripk1"
"Trim81",7.95432436695099e-05,0.277354804218302,0.791,0.54,1,"4","Trim8"
"Ubn11",8.20993276991011e-05,0.257767139024313,0.816,0.585,1,"4","Ubn1"
"Nfkbiz3",0.000204310788358331,0.264417024651334,0.981,0.768,1,"4","Nfkbiz"
"Jun1",0.000218672814030239,0.313069869474072,0.994,0.82,1,"4","Jun"
"Id21",0.000415742500455221,0.337956574269814,0.677,0.519,1,"4","Id2"
"Cxcl101",0.000480175444928207,0.382907749414779,0.525,0.351,1,"4","Cxcl10"
"Rel3",0.000509906542312065,0.276083352047047,0.924,0.699,1,"4","Rel"
"BC0055371",0.00149667614910715,0.266015339761682,0.772,0.611,1,"4","BC005537"
"Ccrl22",0.00506343957473742,0.341841894943069,0.772,0.674,1,"4","Ccrl2"
"Luc7l21",0.00906574886328039,0.263150115643148,0.911,0.715,1,"4","Luc7l2"
"Zfp6222",0.00928502420211357,0.257686990947314,0.684,0.558,1,"4","Zfp622"
"Top2a",6.12110048440855e-214,2.64170335895549,0.701,0.018,1.03213996368097e-209,"5","Top2a"
"Ccna2",3.19605034351526e-208,1.78711841422634,0.619,0.009,5.38918008923544e-204,"5","Ccna2"
"Pclaf",1.46588884359813e-201,2.35255871283608,0.707,0.023,2.47178176807517e-197,"5","Pclaf"
"Pbk",9.51348326113822e-199,1.94065765167814,0.585,0.008,1.60416354749313e-194,"5","Pbk"
"Ube2c",1.2083099975193e-182,2.70848344581882,0.667,0.024,2.03745231781704e-178,"5","Ube2c"
"Nusap1",3.63443680493923e-182,1.6927653497739,0.51,0.004,6.12838734048853e-178,"5","Nusap1"
"Birc5",4.32138690942269e-178,2.47947787986873,0.667,0.026,7.28672260666854e-174,"5","Birc5"
"Ccnb1",1.03507152815722e-174,1.76121391475344,0.531,0.008,1.74533761077871e-170,"5","Ccnb1"
"Cdca3",8.85475580214171e-172,1.81788634446095,0.578,0.015,1.49308892335714e-167,"5","Cdca3"
"Cdca8",1.90632272846742e-168,1.80944677712407,0.599,0.019,3.21444138474176e-164,"5","Cdca8"

"Rrm2",4.06391123782822e-167,1.57991110061304,0.49,0.006,6.85256712922
594e-163,"5","Rrm2"
"Aurkb",4.64172854763948e-163,1.60813483241503,0.578,0.019,7.826882677
02969e-159,"5","Aurkb"
"Prc1",1.82470518141313e-162,1.54696585281705,0.51,0.009,3.07681787689
883e-158,"5","Prc1"
"Tk1",3.03153428061016e-161,1.69744638875553,0.605,0.023,5.11177310396
485e-157,"5","Tk1"
"Cenpe",3.56100171493404e-155,1.72112395198914,0.544,0.016,6.004561091
72178e-151,"5","Cenpe"
"Ndc80",8.06596644208417e-154,1.20531486276979,0.476,0.008,1.360083261
46423e-149,"5","Ndc80"
"Hist1h1b",1.13836165572296e-152,1.77941589922692,0.429,0.003,1.919505
42388005e-148,"5","Hist1h1b"
"Ncapg",9.91284000840711e-152,0.907078301470867,0.422,0.003,1.67150308
221761e-147,"5","Ncapg"
"Melk",4.67835219228656e-144,0.966197942644122,0.415,0.004,7.888637466
63359e-140,"5","Melk"
"Neil3",2.69454331749543e-143,0.745881090227904,0.388,0.002,4.54353894
19608e-139,"5","Neil3"
"Tpx2",2.57651856672016e-141,1.42499228732013,0.497,0.015,4.3445256072
0353e-137,"5","Tpx2"
"Kif11",3.95840334347435e-139,1.34943268583868,0.476,0.013,6.674659717
76645e-135,"5","Kif11"
"Plk1",1.44026491765479e-138,0.965248822882853,0.401,0.004,2.428574704
14951e-134,"5","Plk1"
"Shcbp1",6.41981643477685e-133,0.986950031537927,0.415,0.007,1.0825094
4723207e-128,"5","Shcbp1"
"Spc24",3.56861172296665e-131,1.37179328180888,0.49,0.018,6.0173930872
6636e-127,"5","Spc24"
"Mki67",4.07150956830901e-129,2.47350580176935,0.612,0.043,6.865379434
08265e-125,"5","Mki67"
"Pimreg",2.57632413427945e-127,0.956456079903781,0.367,0.003,4.3441977
5522201e-123,"5","Pimreg"
"Racgap1",2.41004319905434e-125,1.43173218839804,0.531,0.027,4.0638148
4224543e-121,"5","Racgap1"
"Bub1",2.69065418253911e-124,0.770929270485079,0.388,0.006,4.536981082
59745e-120,"5","Bub1"
"Kif23",5.44720075967299e-124,1.1969754721048,0.476,0.019,9.1850699209
606e-120,"5","Kif23"
"Ccnf",2.84793153845135e-123,0.953674253761132,0.367,0.005,4.802182160
13666e-119,"5","Ccnf"
"Esco2",4.43193360369009e-122,0.839738801398266,0.32,0.001,7.473126442
54223e-118,"5","Esco2"
"Ccnb2",6.22889234952661e-121,1.70249681201133,0.517,0.026,1.050315827
97718e-116,"5","Ccnb2"
"Cdk1",6.62341650563327e-120,2.45536356860287,0.762,0.099,1.1168404911
7988e-115,"5","Cdk1"
"Knl1",8.70871685301404e-120,1.08124385499362,0.442,0.015,1.4684638357
5523e-115,"5","Knl1"

"Nuf2",1.37229788633014e-119,0.918628043439726,0.395,0.009,2.31396869592989e-115,"5","Nuf2"
"Fbxo5",1.93509631977167e-117,0.755798173275677,0.354,0.005,3.262959414399e-113,"5","Fbxo5"
"Dlgap5",2.90278779460072e-117,0.77096788031197,0.374,0.007,4.89468077925573e-113,"5","Dlgap5"
"Cks1b",7.83618702026272e-115,1.81252878638445,0.701,0.078,1.3213378553567e-110,"5","Cks1b"
"Kif22",3.5061136540502e-114,0.72534658097689,0.367,0.007,5.91200884345946e-110,"5","Kif22"
"Kif4",4.19787975174977e-114,0.782068514085037,0.32,0.002,7.07846483740045e-110,"5","Kif4"
"Ska1",4.6856694701827e-114,0.540648734594245,0.299,0.001,7.90097586062206e-110,"5","Ska1"
"Mis18bp1",1.0676201084335e-112,0.924783441674135,0.408,0.013,1.80022102684057e-108,"5","Mis18bp1"
"Hmmr",1.39021730280534e-112,0.969748753139939,0.34,0.005,2.34418441599036e-108,"5","Hmmr"
"Kif20a",1.66353036413251e-112,0.806268609947261,0.34,0.005,2.80504490000024e-108,"5","Kif20a"
"Sgo2a",2.91553324504661e-112,0.763470534146191,0.361,0.007,4.91617215779759e-108,"5","Sgo2a"
"Cdc20",1.98420588839841e-111,1.73959084855141,0.442,0.019,3.3457679690174e-107,"5","Cdc20"
"Stmn1",2.28746459275021e-111,3.26619188418487,0.966,0.258,3.85712279629541e-107,"5","Stmn1"
"Cdca2",7.14694232674013e-111,0.729053844118461,0.347,0.006,1.20511741513492e-106,"5","Cdca2"
"Asf1b",4.06286145372805e-110,1.18224021786853,0.51,0.031,6.85079698327623e-106,"5","Asf1b"
"Cep55",6.5152829620137e-110,0.692891934714313,0.34,0.005,1.09860701305475e-105,"5","Cep55"
"Aurka",5.99206494844729e-106,1.18737582212929,0.401,0.015,1.01038199160718e-101,"5","Aurka"
"Anln",3.40413483478418e-105,0.852654154339338,0.361,0.009,5.74005215841308e-101,"5","Anln"
"Kifc1",6.71943166113415e-105,0.61240576300777,0.327,0.005,1.13303056670044e-100,"5","Kifc1"
"Hist1h2ap",1.07758677524051e-103,1.08011858966556,0.327,0.006,1.81702682041054e-99,"5","Hist1h2ap"
"Smc2",2.89574334565844e-103,1.95004349878728,0.694,0.094,4.88280242944926e-99,"5","Smc2"
"Cenpm",6.04611797166076e-99,0.829246547927283,0.367,0.012,1.01949641238144e-94,"5","Cenpm"
"Kif20b",3.27789343823116e-98,0.953445299105752,0.395,0.017,5.52718391554538e-94,"5","Kif20b"
"Cit",1.28408647068506e-97,0.517291195711217,0.313,0.006,2.16522660686915e-93,"5","Cit"
"Knstrn",4.04644560779366e-96,1.07199917926036,0.415,0.021,6.82311658386167e-92,"5","Knstrn"

```
"Aspm",2.03343701830026e-95,0.669542320249851,0.272,0.002,3.4287815002
579e-91,"5","Aspm"
"Rad51",9.89474327155885e-95,0.581694708187385,0.34,0.01,1.66845161045
025e-90,"5","Rad51"
"Ect2",2.59778022186442e-91,0.73887903736452,0.306,0.007,4.38037701010
778e-87,"5","Ect2"
"Cdkn3",4.99834260770755e-91,0.958076323279821,0.34,0.012,8.4282053051
1646e-87,"5","Cdkn3"
"Mad2l1",5.84229692015289e-91,0.682068157023408,0.388,0.019,9.85128106
67618e-87,"5","Mad2l1"
"Tacc3",3.5923900792426e-90,1.24951479755037,0.551,0.055,6.05748815161
887e-86,"5","Tacc3"
"Ckap2",2.81768491379904e-89,0.671660945212783,0.34,0.012,4.7511803016
4793e-85,"5","Ckap2"
"Cenpf",2.88679080828874e-89,1.60116123305408,0.456,0.034,4.8677066609
3648e-85,"5","Cenpf"
"Bub1b",1.95618378992439e-86,0.754622850077075,0.367,0.017,3.298517106
5705e-82,"5","Bub1b"
"Rad51ap1",1.96484042842295e-86,0.789933092189799,0.361,0.016,3.313113
93040679e-82,"5","Rad51ap1"
"Cenpi",2.78701728546715e-86,0.505965083305417,0.272,0.005,4.699468546
75471e-82,"5","Cenpi"
"Spc25",8.44992932668204e-86,0.915143988452074,0.422,0.027,1.424827083
06512e-81,"5","Spc25"
"Ube2t",1.90165385344879e-84,0.687441492813219,0.327,0.012,3.206568727
68535e-80,"5","Ube2t"
"Ncapd2",2.41229708310873e-84,0.632536736393108,0.361,0.017,4.06761534
153794e-80,"5","Ncapd2"
"Smc4",6.33165678970587e-83,1.96429145539861,0.701,0.129,1.06764396788
02e-78,"5","Smc4"
"Uhrf1",7.41119877969198e-81,0.723759990050299,0.381,0.023,1.249676338
23166e-76,"5","Uhrf1"
"Diaph3",5.93342065316362e-80,0.686613684488378,0.327,0.014,1.00049339
053645e-75,"5","Diaph3"
"Spag5",9.81014130655506e-80,0.47254929710893,0.265,0.006,1.6541860271
1131e-75,"5","Spag5"
"Kif15",1.00062543236293e-78,0.78604587983113,0.347,0.018,1.6872546040
5038e-74,"5","Kif15"
"H2afx",1.04192530278348e-78,2.28547551330531,0.741,0.165,1.7568944455
5351e-74,"5","H2afx"
"Cdca5",1.5886989715473e-78,0.591462774321254,0.252,0.005,2.6788642058
2306e-74,"5","Cdca5"
"Tyms",5.11484519079613e-78,1.49702836044807,0.531,0.065,8.62465196072
044e-74,"5","Tyms"
"Cdkn2c",2.26238176211052e-75,1.55736201490123,0.565,0.079,3.814828127
27075e-71,"5","Cdkn2c"
"Rrm1",7.35528891274687e-74,1.23509567359514,0.592,0.091,1.24024881646
738e-69,"5","Rrm1"
"Hmgb2",2.20779996585582e-71,2.53263963791838,0.966,0.526,3.7227923024
2608e-67,"5","Hmgb2"
```

"Cenpa",3.64732172409329e-71,1.70112462184576,0.558,0.083,6.15011389116611e-67,"5","Cenpa"
"Cenpn",2.32958932483577e-70,0.434783678843535,0.272,0.01,3.92815351953808e-66,"5","Cenpn"
"Tuba1b",5.66001537444242e-70,2.20754014220915,0.993,0.834,9.54391792438481e-66,"5","Tuba1b"
"Incenp",2.34429551332475e-69,1.40318900135574,0.592,0.1,3.95295109456819e-65,"5","Incenp"
"Gmnn",3.30760343654065e-68,1.0358488421641,0.551,0.078,5.57728091469484e-64,"5","Gmnn"
"Fignl1",3.63731130279324e-68,0.625358689370319,0.313,0.017,6.13323431876997e-64,"5","Fignl1"
"Cdc45",1.01059933913455e-67,0.368460542103544,0.272,0.011,1.70407260564867e-63,"5","Cdc45"
"Mcm7",9.68067982199795e-67,1.16071770898991,0.544,0.081,1.6323562315853e-62,"5","Mcm7"
"H2afz",2.53629252752027e-66,2.45572618164224,0.993,0.87,4.27669645990467e-62,"5","H2afz"
"Mcm5",3.36754149435741e-65,0.955246862505754,0.49,0.063,5.67834846778547e-61,"5","Mcm5"
"Tubb5",2.65192967098747e-62,2.03287516612177,0.98,0.832,4.47168381121907e-58,"5","Tubb5"
"Pkmyt1",2.45139149451124e-61,0.435789854192066,0.286,0.016,4.13353633804486e-57,"5","Pkmyt1"
"Dut",3.13294054756525e-61,1.13639318055795,0.483,0.067,5.28276435130453e-57,"5","Dut"
"Tcf19",2.70981734559631e-58,0.547454023821544,0.306,0.022,4.5692940081445e-54,"5","Tcf19"
"Slc43a3",4.34174462813591e-57,0.540746813800213,0.354,0.033,7.32104979196277e-53,"5","Slc43a3"
"Rfc4",8.89077078455029e-56,0.76700345470797,0.422,0.053,1.49916176969087e-51,"5","Rfc4"
"Chaf1b",1.45652431226425e-55,0.727274523817501,0.299,0.023,2.45599129533998e-51,"5","Chaf1b"
"Dnajc9",3.05087821474376e-55,0.913481068124164,0.531,0.092,5.14439084570093e-51,"5","Dnajc9"
"Prim1",4.06379581611349e-55,0.736496524328765,0.381,0.042,6.85237250513058e-51,"5","Prim1"
"Atad2",2.38289111685655e-54,1.06840944765324,0.558,0.106,4.01803100124351e-50,"5","Atad2"
"Mcm6",8.21797645263671e-54,1.33359083984564,0.544,0.104,1.3857151894436e-49,"5","Mcm6"
"Ran",8.39167308877285e-54,1.84426628248699,0.952,0.704,1.41500391622888e-49,"5","Ran"
"Fen1",1.80434390081007e-53,1.10757193724605,0.558,0.113,3.04248468554594e-49,"5","Fen1"
"Gins2",3.74937369474758e-53,0.877196049388521,0.469,0.072,6.32219392408337e-49,"5","Gins2"
"Plk4",5.7910682011932e-53,0.645866458275149,0.374,0.042,9.76489920085198e-49,"5","Plk4"

"Tipin",1.60118195613944e-51,1.05610746384936,0.592,0.133,2.6999130144
4233e-47,"5","Tipin"
"Clspn",2.44863639433665e-51,0.733837432272516,0.395,0.052,4.128890688
13046e-47,"5","Clspn"
"Lmnb1",1.35584349390578e-50,1.37002659401311,0.687,0.201,2.2862232994
2392e-46,"5","Lmnb1"
"Kpna2",1.47614830102386e-49,1.34634397326057,0.524,0.104,2.4890812651
8643e-45,"5","Kpna2"
"Ptma",2.60722486154944e-49,1.16510660971919,1,0.96,4.39630256154466e-
45,"5","Ptma"
"Mcm2",2.76805172785743e-49,0.912222677608428,0.415,0.059,4.6674888235
1319e-45,"5","Mcm2"
"Hmgn2",4.1242994730792e-49,1.90140908559328,0.85,0.417,6.954393771506
14e-45,"5","Hmgn2"
"Kif2c",1.83408261774383e-47,0.581662332598319,0.347,0.041,3.092630110
03964e-43,"5","Kif2c"
"Dhfr",2.50953426708806e-47,0.640927572040793,0.313,0.032,4.2315766811
6388e-43,"5","Dhfr"
"Hmgb3",2.8245654919906e-47,0.499774035863519,0.259,0.02,4.76278233259
455e-43,"5","Hmgb3"
"Tagln2",1.3319064328589e-46,1.62233276184142,0.871,0.445,2.2458606270
8668e-42,"5","Tagln2"
"Anp32b",3.42663745771386e-46,1.66361026638385,0.912,0.577,5.777996081
19711e-42,"5","Anp32b"
"Lig1",1.18473316118904e-45,1.09406849353354,0.456,0.084,1.99769705639
696e-41,"5","Lig1"
"Cenpl",1.40271710099252e-45,0.46086203545716,0.265,0.023,2.3652615756
9359e-41,"5","Cenpl"
"Ncaph",3.28174176918103e-45,0.60514752874246,0.333,0.039,5.5336729711
9305e-41,"5","Ncaph"
"Ndc1",5.83860161595201e-45,0.378113153194356,0.293,0.028,9.8450500448
1828e-41,"5","Ndc1"
"Pola1",7.02464564681006e-45,0.556145788940312,0.293,0.03,1.1844957489
6511e-40,"5","Pola1"
"Tmpo",2.3350826066723e-44,1.44681098308787,0.707,0.27,3.9374162913708
3e-40,"5","Tmpo"
"Ube2s",1.74973784248997e-43,1.68064484293595,0.898,0.62,2.95040795000
659e-39,"5","Ube2s"
"Poc1a",2.81824620568058e-42,0.421292182676962,0.252,0.022,4.752126752
01859e-38,"5","Poc1a"
"Hmgb1",1.07078873839154e-41,1.34971671433939,0.959,0.814,1.8055639706
7581e-37,"5","Hmgb1"
"Dbf4",1.47066682252184e-41,0.861713268824725,0.442,0.086,2.4798383961
3633e-37,"5","Dbf4"
"Rfc5",2.82163346778519e-40,0.833586260330566,0.422,0.077,4.7578383533
7939e-36,"5","Rfc5"
"Pmf1",4.71842789851277e-40,0.828566484675954,0.49,0.107,7.95621312247
223e-36,"5","Pmf1"
"Haus5",1.58275432209636e-38,0.379960658313988,0.259,0.027,2.668840337
91888e-34,"5","Haus5"

"Ppia1",1.67223127642242e-38,0.960810587706286,0.986,0.967,2.819716378
30348e-34,"5","Ppia"
"Tubb4b",6.49136471429318e-38,1.93316266421344,0.796,0.408,1.094573918
12412e-33,"5","Tubb4b"
"Rbm31",1.158182663366e-36,1.06879690515437,0.973,0.92,1.9529276069677
5e-32,"5","Rbm3"
"Alyref",1.17128519957724e-36,1.35208255481283,0.81,0.388,1.9750211035
2714e-32,"5","Alyref"
"Ranbp1",4.73880196202135e-36,1.48092976725913,0.85,0.532,7.9905678683
6041e-32,"5","Ranbp1"
"Ncapg2",1.23778562433229e-35,0.415275105584472,0.333,0.051,2.08715411
974911e-31,"5","Ncapg2"
"Hmgn1",1.90992371672318e-35,1.50429622450514,0.891,0.625,3.2205133711
3862e-31,"5","Hmgn1"
"E2f1",2.08740777897682e-35,0.612493868219624,0.401,0.079,3.5197869969
1071e-31,"5","E2f1"
"Hells",2.66113450599385e-35,0.467780213563927,0.272,0.034,4.487205004
00682e-31,"5","Hells"
"Ybx11",4.84120160002391e-35,1.16694449540939,0.952,0.893,8.1632341379
6032e-31,"5","Ybx1"
"Tfdp1",5.52434789370676e-35,0.937082841628015,0.51,0.138,9.3151554183
6833e-31,"5","Tfdp1"
"Cenpq",6.97088800610164e-35,0.579396392659118,0.32,0.049,1.1754311355
8886e-30,"5","Cenpq"
"Sae1",3.79332173707287e-33,1.13387602488876,0.599,0.198,6.39629911305
227e-29,"5","Sae1"
"Lsm2",3.23828630616356e-32,0.941348113991528,0.599,0.202,5.4603983694
5299e-28,"5","Lsm2"
"Cbx5",4.78554376957694e-32,0.689496210114602,0.483,0.126,8.0693839042
6064e-28,"5","Cbx5"
"Hirip3",4.78883262904586e-32,0.709552201736855,0.415,0.093,8.07492957
909713e-28,"5","Hirip3"
"Ezh2",4.82435564953738e-32,0.909420360002736,0.476,0.131,8.1348284962
4994e-28,"5","Ezh2"
"Csrp1",2.39989253147411e-31,0.579906978601989,0.415,0.092,4.046698786
57164e-27,"5","Csrp1"
"Hist1h1e",2.72996572375084e-31,0.839049729331031,0.299,0.05,4.6032682
0338866e-27,"5","Hist1h1e"
"Atad5",5.04321131171517e-31,0.408440689176633,0.272,0.04,8.5038629138
1413e-27,"5","Atad5"
"Hat1",5.36630321122193e-31,1.00493930229284,0.633,0.246,9.04866047476
243e-27,"5","Hat1"
"Rangap1",3.10665693572168e-30,0.857245553202871,0.537,0.175,5.2384449
250139e-26,"5","Rangap1"
"Topbp1",3.76349975290098e-30,0.51211181357858,0.422,0.099,6.346013283
34163e-26,"5","Topbp1"
"Eif5a1",4.04657912119826e-30,0.976118967772607,0.952,0.866,6.82334171
41645e-26,"5","Eif5a"
"Mcm3",4.10057885122635e-30,1.26135325799447,0.639,0.262,6.91439605893
787e-26,"5","Mcm3"

"Iqgap3",4.27584617184693e-30,0.346133663594449,0.252,0.034,7.20993181
49683e-26,"5","Iqgap3"
"Dtymk",7.12850060075364e-30,0.77721614391737,0.503,0.147,1.2020077712
9908e-25,"5","Dtymk"
"Ckap2l",1.10633091954465e-29,1.07428629429201,0.517,0.163,1.865495196
53619e-25,"5","Ckap2l"
"H2afv",1.92195016466322e-29,1.41120179029052,0.653,0.304,3.2407923676
5513e-25,"5","H2afv"
"Orc6",2.55800474326793e-29,0.560777440336832,0.408,0.096,4.3133075980
9839e-25,"5","Orc6"
"Dek",5.93973254747933e-29,1.30454056098084,0.823,0.575,1.001557702155
96e-24,"5","Dek"
"Nup43",1.2562612705238e-28,0.485779280352294,0.293,0.051,2.1183077543
5722e-24,"5","Nup43"
"Pa2g4",1.89078642906086e-28,1.03556724582082,0.741,0.365,3.1882440766
8242e-24,"5","Pa2g4"
"Nt5dc2",5.74169265261481e-28,0.941217347960555,0.503,0.164,9.68164215
08391e-24,"5","Nt5dc2"
"Haus4",6.29426509919947e-28,0.733854935875873,0.401,0.096,1.061338981
02702e-23,"5","Haus4"
"Ddx39",7.75960377838146e-28,1.04964483688483,0.626,0.255,1.3084243891
1068e-23,"5","Ddx39"
"Dck",8.99622767598863e-28,0.638235865226892,0.381,0.089,1.51694391072
52e-23,"5","Dck"
"Nudc",1.11915703956464e-27,1.18628944315424,0.782,0.509,1.88712260011
389e-23,"5","Nudc"
"Cdkn2d",2.28945742999334e-27,1.11157297431518,0.531,0.187,3.860483118
45478e-23,"5","Cdkn2d"
"Snrpb",8.26110994781468e-27,0.963175138118135,0.871,0.76,1.3929883594
0051e-22,"5","Snrpb"
"Gapdh1",1.61552618424821e-26,0.730129118382552,1,0.952,2.724100251879
33e-22,"5","Gapdh"
"Haus1",2.5993864673741e-26,0.592172210121736,0.32,0.066,4.38308546128
62e-22,"5","Haus1"
"Ccdc34",3.53751001333711e-26,0.820771110347242,0.456,0.136,5.96494938
448903e-22,"5","Ccdc34"
"Mcm4",8.06690451045925e-26,0.594332322116904,0.354,0.082,1.3602414385
5364e-21,"5","Mcm4"
"Psip1",9.327196281312e-26,0.73591693068206,0.395,0.103,1.572751836954
83e-21,"5","Psip1"
"Cdk4",1.67784614264138e-25,1.15730340181376,0.762,0.432,2.82918416572
19e-21,"5","Cdk4"
"Prdx4",1.68530578956744e-24,1.0196254876834,0.707,0.385,2.84176262236
861e-20,"5","Prdx4"
"Mis18a",2.58944856206299e-24,0.511892100325815,0.34,0.08,4.3663281653
5062e-20,"5","Mis18a"
"Selenoh",2.89636245534198e-24,1.08681971531768,0.626,0.292,4.88384637
219764e-20,"5","Selenoh"
"Exosc8",4.33731504794467e-24,0.80289703539732,0.619,0.259,7.313580633
8443e-20,"5","Exosc8"

"Syce2",4.36232351999652e-24,0.506526470806082,0.299,0.063,7.355749919
41813e-20,"5","Syce2"
"Hnrnpf",4.7675702441022e-24,0.827442267043066,0.939,0.909,8.039076945
60512e-20,"5","Hnrnpf"
"Sumo2",1.38765111692231e-23,1.01084016617604,0.878,0.773,2.3398573133
5441e-19,"5","Sumo2"
"Bub3",2.24093837387241e-23,1.19622235752446,0.741,0.457,3.77867028602
366e-19,"5","Bub3"
"Cfl11",3.43702806455804e-23,0.58001147197127,0.993,0.979,5.7955167224
5776e-19,"5","Cfl1"
"Rbl1",4.98035422437594e-23,0.467309808496738,0.327,0.077,8.3978732931
4271e-19,"5","Rbl1"
"Nup37",5.21939400338891e-23,0.425096313889579,0.252,0.045,8.800942168
51437e-19,"5","Nup37"
"Jpt2",7.6596787236579e-23,0.583516127373839,0.361,0.093,1.29157502638
32e-18,"5","Jpt2"
"Rnaseh2b",7.9063156857577e-23,0.92856514076752,0.442,0.14,1.333162950
93246e-18,"5","Rnaseh2b"
"Tubg1",1.5584001353705e-22,0.553199989097917,0.395,0.11,2.62777430826
173e-18,"5","Tubg1"
"Atp5b",2.79590396515388e-22,0.827497853805836,0.925,0.852,4.714453266
04247e-18,"5","Atp5b"
"Nde1",3.21419901404839e-22,0.876371269303126,0.51,0.184,5.41978237748
84e-18,"5","Nde1"
"Pold1",3.26228509685782e-22,0.451525436382935,0.252,0.048,5.500865130
32165e-18,"5","Pold1"
"Ppil1",8.02903640110912e-22,0.467505061573053,0.327,0.077,1.353856117
95502e-17,"5","Ppil1"
"Hjurp",9.80565800812223e-22,0.574828574707858,0.381,0.111,1.653430053
32957e-17,"5","Hjurp"
"Nucks1",1.12091517112217e-21,1.20449405149964,0.728,0.436,1.890087161
54621e-17,"5","Nucks1"
"Clic1",2.11211345519234e-21,0.617228348900601,0.966,0.939,3.561445708
14533e-17,"5","Clic1"
"Phgdh",3.48047352825614e-21,1.117038747907,0.66,0.36,5.86877446334551
e-17,"5","Phgdh"
"Hpf1",3.55867967521808e-21,1.02498454682035,0.66,0.346,6.000645668352
73e-17,"5","Hpf1"
"Rpa2",4.17447516363108e-21,0.595998681457778,0.327,0.084,7.0390000209
1472e-17,"5","Rpa2"
"Usp1",5.76921266211833e-21,0.590717449709505,0.463,0.161,9.7280463908
6394e-17,"5","Usp1"
"Lbr1",6.5893456613295e-21,0.559455124319138,0.422,0.134,1.11109546541
338e-16,"5","Lbr"
"Nup85",1.89008863400417e-20,0.408566971621513,0.306,0.074,3.187067454
65783e-16,"5","Nup85"
"H3f3a",2.01783352878589e-20,0.64172406141539,1,0.964,3.40247089623877
e-16,"5","H3f3a"
"Ckap5",2.02058614260093e-20,0.875309406548576,0.517,0.21,3.4071123536
537e-16,"5","Ckap5"

"Pfn11",2.10256012103678e-20,0.660770490677435,0.959,0.945,3.54533687609223e-16,"5","Pfn1"
"Dnmt1",2.4826196789101e-20,0.615004692824877,0.422,0.14,4.18619330257821e-16,"5","Dnmt1"
"Atp5f11",2.71130785200154e-20,0.848881740401516,0.918,0.809,4.571807300045e-16,"5","Atp5f1"
"Eif1ad",4.75936322902037e-20,0.857825726716524,0.51,0.198,8.02523827677415e-16,"5","Eif1ad"
"Rbbp7",5.42641543147694e-20,0.845807275767305,0.687,0.385,9.15002170055641e-16,"5","Rbbp7"
"Oaz11",7.14233580008328e-20,0.597107897038372,0.98,0.967,1.20434066261004e-15,"5","Oaz1"
"Rpsa1",8.41622135466881e-20,0.572975635040027,1,0.985,1.41914324482425e-15,"5","Rpsa"
"Anapc5",9.30029391572645e-20,0.926194890836647,0.728,0.46,1.56821556006979e-15,"5","Anapc5"
"Ubb",9.64085507303358e-20,0.593207416328005,0.993,0.977,1.62564098241492e-15,"5","Ubb"
"Rps22",9.64435342364293e-20,0.566891195551918,1,0.987,1.62623087429467e-15,"5","Rps2"
"Nmral1",1.06930909696042e-19,0.644443949166307,0.374,0.11,1.80306899929466e-15,"5","Nmral1"
"Nsd2",1.38181478431439e-19,0.640717670003502,0.49,0.193,2.33001608931092e-15,"5","Nsd2"
"Hspa14",1.38795017628706e-19,0.935543432273617,0.646,0.314,2.34036158725524e-15,"5","Hspa14"
"Tuba1c1",1.90994496978558e-19,1.18261114007121,0.782,0.568,3.22054920805244e-15,"5","Tuba1c"
"Anp32e",2.5850349411977e-19,0.912704176226398,0.667,0.382,4.35888591784756e-15,"5","Anp32e"
"Mrpl18",2.8938988180209e-19,0.929049428849356,0.653,0.337,4.87969218694685e-15,"5","Mrpl18"
"Rbbp4",3.90087177912913e-19,1.09169996057279,0.789,0.532,6.57764999396753e-15,"5","Rbbp4"
"Pcna",7.66858935158404e-19,1.29341167737378,0.619,0.371,1.2930775364641e-14,"5","Pcna"
"Slc25a10",7.98219674107279e-19,0.551368182177832,0.347,0.102,1.34595801447969e-14,"5","Slc25a10"
"Hnrnpa3",9.40575476560519e-19,0.851602225203011,0.864,0.774,1.58599836857635e-14,"5","Hnrnpa3"
"Nedd1",1.64395998281701e-18,0.351679374196833,0.306,0.078,2.77204532302604e-14,"5","Nedd1"
"G2e3",1.72737508158764e-18,0.643225941981738,0.313,0.088,2.91269986257308e-14,"5","G2e3"
"Alad",2.21524714498053e-18,0.37084571713593,0.272,0.064,3.73534973586616e-14,"5","Alad"
"Adk",3.38176885640718e-18,0.384133737458808,0.333,0.093,5.70233864567378e-14,"5","Adk"
"Nrm",4.66910353153492e-18,0.71064058548416,0.585,0.278,7.87304237487419e-14,"5","Nrm"

"Arhgap11a",5.75851580834718e-18,0.52897824013346,0.347,0.106,9.71000935603502e-14,"5","Arhgap11a"
"Srsf7",7.32220669518507e-18,0.846178290741442,0.762,0.549,1.23467049294211e-13,"5","Srsf7"
"Cmc2",8.72206181466547e-18,0.474378291640671,0.279,0.073,1.47071406318889e-13,"5","Cmc2"
"Mrfap1",1.10135483847832e-17,0.825991782809902,0.932,0.8,1.85710452864214e-13,"5","Mrfap1"
"Jpt1",1.11633625109049e-17,1.03131472381959,0.823,0.718,1.88236618658879e-13,"5","Jpt1"
"Raly",1.52706707386634e-17,0.785906849469022,0.83,0.611,2.57494049995342e-13,"5","Raly"
"Timm50",2.48933320178088e-17,0.694456385794828,0.544,0.249,4.19751364484291e-13,"5","Timm50"
"Gatm1",3.56050892584291e-17,0.897115282890899,0.85,0.706,6.00373015075631e-13,"5","Gatm"
"Nsmce1",4.32850900854519e-17,0.875898426863853,0.544,0.254,7.2987318902089e-13,"5","Nsmce1"
"Reep4",5.6362180590502e-17,0.571114813059657,0.388,0.136,9.50379089117045e-13,"5","Reep4"
"Ywhaq",1.10113603881445e-16,0.908014251348017,0.782,0.599,1.85673558864892e-12,"5","Ywhaq"
"Nup205",1.37891255183016e-16,0.356008272352279,0.279,0.073,2.32512234489601e-12,"5","Nup205"
"Pgp1",1.47391091334682e-16,0.812827132422652,0.619,0.323,2.48530858208541e-12,"5","Pgp"
"Cenpc1",1.60339794784734e-16,0.260614285521734,0.279,0.073,2.70364961966018e-12,"5","Cenpc1"
"Mrpl51",1.84825733420599e-16,0.845395759081721,0.51,0.228,3.11653151693814e-12,"5","Mrpl51"
"Xpo1",2.24923911672913e-16,0.462818384656568,0.476,0.193,3.79266699862866e-12,"5","Xpo1"
"Med30",2.4785274352659e-16,0.835571896088026,0.558,0.262,4.17929296134536e-12,"5","Med30"
"Set",2.6974807884216e-16,0.802160355638155,0.85,0.713,4.54849210543651e-12,"5","Set"
"Banf1",3.12114998673293e-16,0.76304925669511,0.565,0.263,5.26288310762907e-12,"5","Banf1"
"Hprt",3.57344147604794e-16,0.87338548435604,0.81,0.632,6.02553701691204e-12,"5","Hprt"
"Rcc1",3.98030202334459e-16,0.397159602395994,0.279,0.075,6.71158527176365e-12,"5","Rcc1"
"Rpa1",1.00161438210284e-15,0.441096405560362,0.367,0.129,1.6889221711018e-11,"5","Rpa1"
"Paics",1.03916651756774e-15,0.719371074855405,0.558,0.284,1.75224258192272e-11,"5","Paics"
"Eif4a11",1.03991484662814e-15,0.530020667936687,0.946,0.937,1.75350441438437e-11,"5","Eif4a1"
"Txnl11",1.26914889104574e-15,0.753226554143549,0.782,0.563,2.14003886008133e-11,"5","Txnl1"

"Erh",1.37643151188445e-15,0.62383409836898,0.49,0.211,2.3209388153395
7e-11,"5","Erh"
"Rnf26",1.49312363220131e-15,0.457090269472953,0.313,0.096,2.517705068
61785e-11,"5","Rnf26"
"Nxt1",1.55502255369265e-15,0.388912370909051,0.361,0.12,2.62207903003
654e-11,"5","Nxt1"
"Ppih",1.59859217545448e-15,0.436714285293506,0.279,0.078,2.6955461262
5135e-11,"5","Ppih"
"Fkbp3",2.35002501695082e-15,0.776045044758199,0.565,0.291,3.962612183
58247e-11,"5","Fkbp3"
"Nans",2.36888628825178e-15,0.614346609231217,0.531,0.241,3.9944160592
5015e-11,"5","Nans"
"Ppp1cc",2.60557715451644e-15,0.751072785945431,0.769,0.591,4.39352419
794562e-11,"5","Ppp1cc"
"Ssrp1",2.84153985537031e-15,0.794089981398819,0.626,0.382,4.791404504
12541e-11,"5","Ssrp1"
"Nelfe",4.63828133619732e-15,0.73934825214027,0.497,0.23,7.82106998909
592e-11,"5","Nelfe"
"Psmb8",5.44113296542447e-15,0.731502026704422,0.891,0.76,9.1748384062
9874e-11,"5","Psmb8"
"Alg8",6.03426849582681e-15,0.356206959563307,0.313,0.097,1.0174983537
6632e-10,"5","Alg8"
"Rfc2",6.49435722957746e-15,0.800063040768772,0.605,0.338,1.0950785160
5135e-10,"5","Rfc2"
"Fh11",8.32664336016506e-15,0.568942337659533,0.476,0.201,1.4040386033
9103e-10,"5","Fh1"
"Npl1",8.47833478600925e-15,0.888570255208586,0.667,0.401,1.4296168116
1688e-10,"5","Npl"
"Stub1",1.0443103463254e-14,0.706954036528373,0.707,0.439,1.7609161059
7389e-10,"5","Stub1"
"Ppp1ca",1.22192815843669e-14,0.642221680480323,0.884,0.796,2.06041526
075594e-10,"5","Ppp1ca"
"Gas2l3",1.28627837776286e-14,0.352638088695065,0.252,0.069,2.16892260
058373e-10,"5","Gas2l3"
"Nudt5",1.29343408886057e-14,0.62362261833583,0.429,0.177,2.1809885606
3669e-10,"5","Nudt5"
"Tex30",1.34109636390254e-14,0.444217858243353,0.354,0.123,2.261356688
81246e-10,"5","Tex30"
"Fam96a",1.84295910359498e-14,0.749485558179461,0.714,0.483,3.10759764
048186e-10,"5","Fam96a"
"Idh3a",1.96953153720878e-14,0.621691549674119,0.449,0.191,3.321024078
04144e-10,"5","Idh3a"
"Lmf2",2.56325530029244e-14,0.458675096490477,0.333,0.114,4.3221610873
5312e-10,"5","Lmf2"
"Ncaph2",2.7806838603695e-14,0.67765545422076,0.497,0.237,4.6887891253
5505e-10,"5","Ncaph2"
"Arl6ip1",3.6189877912183e-14,1.08714667386037,0.857,0.806,6.102337213
55229e-10,"5","Arl6ip1"
"Snrnp40",3.72127393193023e-14,0.595062697123409,0.537,0.251,6.2748121
0402076e-10,"5","Snrnp40"

"Cks21",4.75625314571189e-14,0.882549365158409,0.565,0.307,8.01999405429938e-10,"5","Cks2"
"Cotl11",4.92597672740423e-14,0.521558936649813,0.959,0.939,8.30618195774901e-10,"5","Cotl1"
"Impdh21",5.1022745209675e-14,0.757176754593932,0.687,0.426,8.60345529725539e-10,"5","Impdh2"
"Mrpl12",6.37302714286609e-14,0.683675722272819,0.605,0.335,1.07461983683008e-09,"5","Mrpl12"
"Rad21",8.08277238175246e-14,0.72492613965045,0.565,0.314,1.3629170790111e-09,"5","Rad21"
"Myef2",8.42532892831633e-14,0.397346183305896,0.32,0.107,1.4206789638927e-09,"5","Myef2"
"Hdgf",9.85406024643378e-14,0.808896624230578,0.66,0.44,1.66159163875366e-09,"5","Hdgf"
"Rfwd3",1.14725069390735e-13,0.265715161535393,0.286,0.087,1.93449412006657e-09,"5","Rfwd3"
"Serbp1",1.19981299890269e-13,0.721386129446826,0.898,0.8,2.02312467874972e-09,"5","Serbp1"
"Vdac3",1.51881053102729e-13,0.878325758669618,0.762,0.556,2.56101831741822e-09,"5","Vdac3"
"Acadl",2.09605384651438e-13,0.682827007248135,0.605,0.338,3.53436599599255e-09,"5","Acadl"
"Dna2",2.44674975213302e-13,0.253402975284734,0.354,0.122,4.1257094320467e-09,"5","Dna2"
"Rnaseh2a",2.82919319365352e-13,0.433452472248935,0.333,0.117,4.77058556313856e-09,"5","Rnaseh2a"
"Bag11",3.21407322629409e-13,0.715088229392809,0.823,0.679,5.41957027417709e-09,"5","Bag1"
"Smc1a",4.02706649572045e-13,0.749046229595362,0.633,0.417,6.79043952508383e-09,"5","Smc1a"
"A430005L14Rik",4.04459559066336e-13,0.366677068774337,0.415,0.166,6.81999708497656e-09,"5","A430005L14Rik"
"Mrpl28",4.15325244831749e-13,0.760292742763743,0.531,0.276,7.00321427835296e-09,"5","Mrpl28"
"Snrnp25",4.17214040621328e-13,0.369160971058171,0.272,0.086,7.03506315295684e-09,"5","Snrnp25"
"Cct7",6.72621124286269e-13,0.688748312436046,0.714,0.519,1.13417373977151e-08,"5","Cct7"
"Calm2",6.98074344308102e-13,0.653176503422542,0.932,0.875,1.17709295937232e-08,"5","Calm2"
"Zwint",7.01445069592439e-13,0.587728815291953,0.578,0.309,1.18277667634677e-08,"5","Zwint"
"Thoc71",8.99649275097132e-13,0.786671083200744,0.585,0.323,1.51698860766878e-08,"5","Thoc7"
"Metrn",9.26933817554787e-13,0.267516841627288,0.293,0.095,1.56299580316088e-08,"5","Metrn"
"Cyc1",9.35849702205088e-13,0.755359663176867,0.694,0.48,1.57802976785822e-08,"5","Cyc1"
"Zcwpw1",1.07898621814616e-12,0.42244751723025,0.293,0.1,1.81938656103805e-08,"5","Zcwpw1"

"Cd72",1.13800993775773e-12,0.773729751263451,0.612,0.345,1.9189123570
4708e-08,"5","Cd72"
"Prelid11",1.18536150475725e-12,0.792103768327101,0.776,0.588,1.998756
56932167e-08,"5","Prelid1"
"Eif1ax",1.29788633217455e-12,0.630009793351578,0.558,0.279,2.18849593
331273e-08,"5","Eif1ax"
"Ybx3",1.40127636640763e-12,0.687011321996622,0.476,0.219,2.3628322090
3655e-08,"5","Ybx3"
"Rplp01",1.50510922812767e-12,0.412353908209927,0.993,0.984,2.53791518
046887e-08,"5","Rplp0"
"Tspo",1.92705982545856e-12,0.703165143225712,0.85,0.715,3.24940827768
822e-08,"5","Tspo"
"Cops5",2.25337121275846e-12,0.656015991269433,0.626,0.356,3.799634538
95332e-08,"5","Cops5"
"Smc3",2.44057631444449e-12,0.634679891482947,0.619,0.387,4.1152997814
1631e-08,"5","Smc3"
"Saal1",2.67746234855819e-12,0.282283760303757,0.259,0.08,4.5147370121
3882e-08,"5","Saal1"
"Dynll2",2.82446139743448e-12,0.699250497286124,0.619,0.372,4.76260680
835402e-08,"5","Dynll2"
"Hnrnpa1",3.16531671843197e-12,0.656550058935488,0.816,0.746,5.3373570
5061998e-08,"5","Hnrnpa1"
"Nes",3.26510549471195e-12,0.743124625184586,0.578,0.328,5.50562088518
329e-08,"5","Nes"
"Siva1",3.44235999638076e-12,0.487471459717996,0.354,0.14,5.8045074258
9723e-08,"5","Siva1"
"Ywhae",3.6150345753257e-12,0.596996326410268,0.884,0.848,6.0956713009
1419e-08,"5","Ywhae"
"Shmt2",3.61563145637708e-12,0.303650533659864,0.293,0.1,6.09667776174
303e-08,"5","Shmt2"
"Elof1",4.31023004093964e-12,0.575142645386583,0.537,0.292,7.267909895
03242e-08,"5","Elof1"
"Ssna11",4.41116999109113e-12,0.701002021437865,0.49,0.248,7.438114838
97786e-08,"5","Ssna1"
"Tceal9",4.74163400499655e-12,0.615511872930098,0.565,0.293,7.99534325
922518e-08,"5","Tceal9"
"Actr3",5.17668985351003e-12,0.545731604965911,0.939,0.886,8.728934430
98861e-08,"5","Actr3"
"Rfc3",6.02893850091606e-12,0.413157905316016,0.286,0.1,1.016599610024
47e-07,"5","Rfc3"
"Eri1",7.02460486423443e-12,0.49919993281771,0.442,0.201,1.18448887220
721e-07,"5","Eri1"
"Tmem14c",7.81600694551794e-12,0.711785255005869,0.823,0.703,1.3179350
9115323e-07,"5","Tmem14c"
"Coq7",8.0729607719316e-12,0.34879124764066,0.395,0.166,1.361262645363
11e-07,"5","Coq7"
"Gins4",8.38594645614297e-12,0.36843643446266,0.388,0.164,1.4140382914
3483e-07,"5","Gins4"
"Psmb5",8.59075713746882e-12,0.658203647063403,0.782,0.606,1.448573468
51999e-07,"5","Psmb5"

"Psma1",9.14920663341078e-12,0.735277988711523,0.796,0.652,1.542739222
52573e-07,"5","Psma1"
"Hint1",1.01799190220351e-11,0.760698219712631,0.816,0.728,1.716537945
49557e-07,"5","Hint1"
"Bex3",1.07533556221504e-11,0.600596711132739,0.306,0.114,1.8132308250
07e-07,"5","Bex3"
"Cbx3",1.11479772513439e-11,0.973705803677579,0.653,0.433,1.8797719241
2161e-07,"5","Cbx3"
"Psmc4",1.52607348879454e-11,0.70047977900342,0.66,0.443,2.57326511680
535e-07,"5","Psmc4"
"Slc25a5",1.5512032404128e-11,0.563935531995182,0.905,0.897,2.61563890
398407e-07,"5","Slc25a5"
"Cct8",1.60110983537977e-11,0.744466243029043,0.803,0.615,2.6997914044
1737e-07,"5","Cct8"
"Rdm1",1.64121409171544e-11,0.440004942084457,0.279,0.099,2.7674152014
5058e-07,"5","Rdm1"
"Cdt1",1.64460619162969e-11,0.363798183114123,0.272,0.096,2.7731349603
2598e-07,"5","Cdt1"
"Tpm4",1.92477701977183e-11,0.618698353952802,0.735,0.568,3.2455590107
3925e-07,"5","Tpm4"
"Rpl42",2.57944497851333e-11,0.490402925082715,0.946,0.926,4.349460122
76918e-07,"5","Rpl4"
"Srsf9",2.63264425058876e-11,0.713016327781988,0.844,0.745,4.439164735
34277e-07,"5","Srsf9"
"Cxcl102",2.80978051985563e-11,1.38795310355068,0.571,0.348,4.73785191
258057e-07,"5","Cxcl10"
"Pold3",2.81719276359446e-11,0.423301656832721,0.367,0.148,4.750350437
97297e-07,"5","Pold3"
"Srsf3",2.97826841597386e-11,0.595135417895635,0.844,0.806,5.021956203
01513e-07,"5","Srsf3"
"Psat1",3.07177305583943e-11,0.414516810066285,0.415,0.184,5.179623726
75646e-07,"5","Psat1"
"Ift27",3.1215455262698e-11,0.371168776389769,0.286,0.102,5.2635500663
9613e-07,"5","Ift27"
"Fundc2",3.16161602755854e-11,0.434459385727441,0.558,0.297,5.33111694
566921e-07,"5","Fundc2"
"Tmsb4x1",3.68140932839264e-11,0.457281537726375,1,0.997,6.20759240953
568e-07,"5","Tmsb4x"
"Akr1b31",4.14207389257711e-11,0.630625902218465,0.714,0.519,6.9843649
9766351e-07,"5","Akr1b3"
"Miip",4.47288265243853e-11,0.420562662735916,0.354,0.15,7.54217472854
186e-07,"5","Miip"
"Nxt2",4.98083679304097e-11,0.59272027919416,0.429,0.201,8.39868700042
569e-07,"5","Nxt2"
"Hsp90aa11",5.40017328004123e-11,0.532268001769546,0.85,0.824,9.105772
18480552e-07,"5","Hsp90aa1"
"Psmd14",6.80342639134723e-11,0.602393658849365,0.612,0.371,1.14719375
810897e-06,"5","Psmd14"
"Nsmce4a",7.32512659833712e-11,0.802062984492252,0.551,0.333,1.2351628
4701161e-06,"5","Nsmce4a"

"H2afy",7.88178995178131e-11,0.634166266973329,0.823,0.752,1.32902742166936e-06,"5","H2afy"
"Fkbp4",8.99057785401119e-11,0.5503305360404,0.605,0.362,1.51599123774337e-06,"5","Fkbp4"
"Cct3",9.0383242779221e-11,0.627539824832031,0.728,0.529,1.52404223974322e-06,"5","Cct3"
"Nap1l11",9.44142687250051e-11,0.904509307124045,0.646,0.455,1.59201339924104e-06,"5","Nap1l1"
"Calm3",1.0021260426697e-10,0.647511087254085,0.687,0.519,1.68978493314965e-06,"5","Calm3"
"Hnrnpd",1.04886926102348e-10,0.686798430218106,0.762,0.639,1.76860334793779e-06,"5","Hnrnpd"
"Arpc1b1",1.06035242419967e-10,0.383136185877353,0.966,0.976,1.78796625768548e-06,"5","Arpc1b"
"Pgk11",1.07363352678e-10,0.426941562989845,0.782,0.564,1.81036085285644e-06,"5","Pgk1"
"Dkc1",1.20487996289968e-10,0.381721579246816,0.347,0.142,2.03166859344144e-06,"5","Dkc1"
"Lamtor1",1.23748718634196e-10,0.75378184105556,0.844,0.708,2.08665089360981e-06,"5","Lamtor1"
"Psma6",1.46431467303452e-10,0.557870795419869,0.673,0.469,2.46912740167081e-06,"5","Psma6"
"Capza21",1.63072708461651e-10,0.454946447907553,0.946,0.932,2.74973201008036e-06,"5","Capza2"
"Rhoa1",1.74602806942756e-10,0.450819065351644,0.952,0.934,2.94415253066876e-06,"5","Rhoa"
"Pgam11",1.88861882941341e-10,0.400076435296656,0.83,0.674,3.18458907015688e-06,"5","Pgam1"
"Nudcd2",1.97530283780519e-10,0.308792879258162,0.415,0.191,3.33075564510711e-06,"5","Nudcd2"
"Hacd2",1.99281749556407e-10,0.55057662217617,0.524,0.291,3.36028886102014e-06,"5","Hacd2"
"Nop58",2.13507879456928e-10,0.685284692126831,0.558,0.324,3.60016986340271e-06,"5","Nop58"
"Bckdk",2.33940516450423e-10,0.586290415341031,0.456,0.233,3.94470498838703e-06,"5","Bckdk"
"Uchl5",2.36021851665256e-10,0.539388860197296,0.456,0.23,3.97980046277954e-06,"5","Uchl5"
"Lsm4",2.3886834351028e-10,0.736517295987774,0.633,0.398,4.02779800827034e-06,"5","Lsm4"
"Vars",2.63753868999224e-10,0.56257224971125,0.537,0.3,4.44741773906492e-06,"5","Vars"
"Fzr1",2.70414317127541e-10,0.591675037287518,0.435,0.222,4.55972621540459e-06,"5","Fzr1"
"Ilf2",2.78368723385269e-10,0.457459410549878,0.442,0.217,4.6938534137224e-06,"5","Ilf2"
"U2af1",3.21525397580288e-10,0.597419911585082,0.639,0.435,5.42156125399882e-06,"5","U2af1"
"Prmt11",3.44340837134829e-10,0.635064640493456,0.639,0.441,5.80627519576749e-06,"5","Prmt1"

"Rpp30",3.61814781989898e-10,0.32403724705695,0.313,0.125,6.1009208539
1366e-06,"5","Rpp30"
"Maz",3.6417279232407e-10,0.579298390816337,0.578,0.345,6.140681624168
47e-06,"5","Maz"
"Snx21",3.84836763364776e-10,0.781954345034521,0.762,0.581,6.489117503
85685e-06,"5","Snx2"
"Rps61",4.70900846685467e-10,0.357025930090622,0.986,0.964,7.940330076
81034e-06,"5","Rps6"
"Stip1",6.58287196999562e-10,0.401988432393388,0.503,0.255,1.110003871
58066e-05,"5","Stip1"
"Arhgdib1",6.7793654635516e-10,0.535802421146262,0.918,0.864,1.1431366
0446407e-05,"5","Arhgdib"
"Aes",7.02626938348858e-10,0.75849703799859,0.762,0.627,1.184769543443
84e-05,"5","Aes"
"Park71",8.58732601433384e-10,0.697910710413965,0.782,0.679,1.44799491
253697e-05,"5","Park7"
"Tbc1d31",8.93752774735083e-10,0.276505411518165,0.299,0.118,1.5070459
287583e-05,"5","Tbc1d31"
"Pycard",8.95993742186207e-10,0.675454017751302,0.85,0.744,1.510824648
07438e-05,"5","Pycard"
"Psmd21",9.67884418136683e-10,0.571106064197411,0.639,0.443,1.63204670
586207e-05,"5","Psmd2"
"Erp29",9.74452916429864e-10,0.393373037802919,0.898,0.922,1.643122507
68404e-05,"5","Erp29"
"Nasp1",9.88121849876587e-10,0.707617449747523,0.571,0.39,1.6661710632
619e-05,"5","Nasp"
"Strap",1.00872870479151e-09,0.594490576061054,0.673,0.454,1.700918342
01945e-05,"5","Strap"
"Naa50",1.01681092668419e-09,0.597926009928315,0.619,0.407,1.714546584
57488e-05,"5","Naa50"
"Psmd13",1.04898703273929e-09,0.663879270061853,0.646,0.446,1.76880193
4605e-05,"5","Psmd13"
"Prpf31",1.16124120722095e-09,0.514347444227593,0.361,0.165,1.95808492
361597e-05,"5","Prpf31"
"Rac2",1.31402832931241e-09,0.638332194644823,0.857,0.754,2.2157145688
8658e-05,"5","Rac2"
"Gps11",1.38881556426522e-09,0.561952802881547,0.612,0.378,2.341820804
46402e-05,"5","Gps1"
"Mrpl40",1.69553840989192e-09,0.494836226766737,0.395,0.189,2.85901686
675975e-05,"5","Mrpl40"
"Apex1",1.87118945216477e-09,0.546634834827989,0.469,0.253,3.155199654
24023e-05,"5","Apex1"
"Hnrnpk",1.91008853327697e-09,0.404057815401919,0.939,0.899,3.22079128
481162e-05,"5","Hnrnpk"
"Ppp1r14b1",2.18681000635575e-09,0.665133500136808,0.796,0.708,3.68739
903271707e-05,"5","Ppp1r14b"
"Tomm40",2.29714741272977e-09,0.632082722114554,0.592,0.377,3.87344996
734494e-05,"5","Tomm40"
"Cenpb",2.4473651933801e-09,0.762317547497482,0.633,0.416,4.1267471890
7752e-05,"5","Cenpb"

"Fam111a",2.52846692566857e-09,0.520145396020818,0.544,0.335,4.2635009
3006233e-05,"5","Fam111a"
"Hdac2",2.92057118704563e-09,0.518352969033044,0.429,0.215,4.924667135
59634e-05,"5","Hdac2"
"Sap301",2.94323888090546e-09,0.601222228845384,0.442,0.227,4.96288940
098279e-05,"5","Sap30"
"Nono",2.95401652418549e-09,0.574739618308452,0.694,0.492,4.9810626630
8157e-05,"5","Nono"
"Asf1a",3.26234450268605e-09,0.346250806578817,0.333,0.145,5.500965300
42921e-05,"5","Asf1a"
"Acp1",3.48287394512257e-09,0.591829187111389,0.544,0.324,5.8728220462
6567e-05,"5","Acp1"
"Snrpd1",3.55288646802097e-09,0.592675877122915,0.503,0.317,5.99087716
237695e-05,"5","Snrpd1"
"Cdk2ap1",3.55662825991041e-09,0.726987200665709,0.633,0.444,5.9971865
7186093e-05,"5","Cdk2ap1"
"Ddost",3.69773324381983e-09,0.565927464869939,0.741,0.613,6.235117795
72899e-05,"5","Ddost"
"Lrrc59",4.24163297243203e-09,0.573968826678878,0.592,0.388,7.15224151
811488e-05,"5","Lrrc59"
"Tmx1",4.34948697750708e-09,0.517487543278319,0.551,0.321,7.3341049414
7244e-05,"5","Tmx1"
"Cdpf1",4.73256998839908e-09,0.272023445874365,0.299,0.119,7.980059514
43853e-05,"5","Cdpf1"
"Dcps",4.98080445438159e-09,0.428179832472467,0.395,0.188,8.3986324709
7823e-05,"5","Dcps"
"Arpc21",5.15738338948771e-09,0.353550497691316,0.946,0.962,8.69637987
135419e-05,"5","Arpc2"
"Nubp1",5.27777670352267e-09,0.444936245905901,0.524,0.292,8.899387077
47992e-05,"5","Nubp1"
"Ppa1",5.63303468229164e-09,0.420369041949851,0.374,0.172,9.4984230812
8016e-05,"5","Ppa1"
"Psmg2",5.79633460501789e-09,0.416927498984719,0.299,0.124,9.773779410
98117e-05,"5","Psmg2"
"Snrpa1",5.96402906380191e-09,0.578487848238379,0.483,0.258,0.00010056
5458073828,"5","Snrpa1"
"Pkig",6.26466067187382e-09,0.72870271525388,0.782,0.635,0.00010563470
8249136,"5","Pkig"
"Apip",6.35777507957128e-09,0.400429187072697,0.367,0.172,0.0001072048
03391731,"5","Apip"
"Sept11",6.88950845286656e-09,0.253978103854597,0.279,0.11,0.000116170
891532236,"5","Sept11"
"Elavl1",7.88079466486913e-09,0.519475151430063,0.694,0.519,0.00013288
5959639023,"5","Elavl1"
"Ywhah",7.90941263641039e-09,0.480385236605481,0.864,0.817,0.000133368
515875152,"5","Ywhah"
"Arhgdia1",8.22926200666482e-09,0.424018417406637,0.891,0.88,0.0001387
61815956382,"5","Arhgdia"
"Cryl1",9.10198343019162e-09,0.518635576451952,0.605,0.387,0.000153477
644599891,"5","Cryl1"

"Gnb2",9.46046369629995e-09,0.449245860161438,0.932,0.906,0.0001595223
3884701,"5","Gnb2"
"Psma5",9.89327129646252e-09,0.543915355778734,0.728,0.555,0.000166820
340600951,"5","Psma5"
"Nudt21",9.92985452111505e-09,0.501673202491021,0.503,0.304,0.00016743
7206935042,"5","Nudt21"
"Bcas2",1.04658430013893e-08,0.628299068746207,0.639,0.425,0.000176475
044689427,"5","Bcas2"
"Akr1a12",1.11446486731336e-08,0.492440356951635,0.898,0.858,0.0001879
21065926378,"5","Akr1a1"
"Emc8",1.18538287586597e-08,0.397280001385705,0.49,0.267,0.00019987926
0528519,"5","Emc8"
"Ssb",1.27342548568258e-08,0.583261751994411,0.748,0.633,0.00021472500
5395797,"5","Ssb"
"Ints11",1.28625050615501e-08,0.401878623259131,0.388,0.188,0.00021688
7560347857,"5","Ints11"
"Hnrnpab1",1.30946815123638e-08,0.711310088356282,0.755,0.629,0.000220
802519661478,"5","Hnrnpab"
"Ncl",1.48091889582727e-08,0.480603602891696,0.85,0.813,0.000249712544
214394,"5","Ncl"
"Ptgr1",1.49803960125602e-08,0.318128436215139,0.32,0.138,0.0002525994
3756379,"5","Ptgr1"
"Pin1",1.54176416892578e-08,0.521146986243322,0.524,0.307,0.0002599722
74164264,"5","Pin1"
"Psmb91",1.60883964508873e-08,0.515564191626303,0.578,0.363,0.00027128
2540954862,"5","Psmb9"
"Dxo",1.69716241040861e-08,0.271253755326982,0.313,0.132,0.00028617552
56431,"5","Dxo"
"Erg28",1.79882460671194e-08,0.408028291171143,0.422,0.22,0.0003033178
05183768,"5","Erg28"
"Ddx39b",1.81460534114209e-08,0.54613874675979,0.762,0.673,0.000305978
75262338,"5","Ddx39b"
"Ube2m",1.83076936510355e-08,0.558148601695502,0.701,0.537,0.000308704
330343761,"5","Ube2m"
"Psmc2",1.85792684742444e-08,0.524479158349604,0.66,0.521,0.0003132836
2501271,"5","Psmc2"
"Sf3a31",2.13296018127962e-08,0.289803253751244,0.456,0.246,0.00035965
9745767369,"5","Sf3a3"
"Npm1",2.2466983779971e-08,0.49264478654766,0.884,0.889,0.000378838280
497871,"5","Npm1"
"Eif2s1",2.24674293088417e-08,0.591628544916695,0.612,0.441,0.00037884
5793005689,"5","Eif2s1"
"Mrpl13",2.34460371837019e-08,0.417039997220276,0.463,0.251,0.00039534
7078991581,"5","Mrpl13"
"Rpl33",2.352599557752e-08,0.355746401633406,0.98,0.972,0.000396695337
428143,"5","Rpl3"
"Coro1a",2.58861674508671e-08,0.324872958638941,0.932,0.933,0.00043649
2555556522,"5","Coro1a"
"Golt1b",2.60215436785418e-08,0.544999357537866,0.361,0.179,0.00043877
5269507573,"5","Golt1b"

"Brip1os",2.76132454838576e-08,0.306992652783944,0.327,0.148,0.0004656 14545348807,"5","Brip1os"
"Commd3",2.83226559778808e-08,0.517095114851195,0.701,0.474,0.00047757 6625099026,"5","Commd3"
"Hsp90ab1",2.84011832004187e-08,0.400342883820788,0.952,0.963,0.000478 90075112546,"5","Hsp90ab1"
"1110008F13Rik",2.91830019853385e-08,0.581165636282401,0.796,0.629,0.0 00492083779476777,"5","1110008F13Rik"
"Eef1e1",3.24156986861303e-08,0.332844233468781,0.388,0.187,0.00054659 3511245529,"5","Eef1e1"
"Slc29a1",3.44370245457071e-08,0.567809597678908,0.605,0.449,0.0005806 77107889713,"5","Slc29a1"
"Mthfd2",3.62768564198944e-08,0.330055600529732,0.259,0.103,0.00061170 035295226,"5","Mthfd2"
"Ckb1",4.03086739272657e-08,0.476268709811158,0.925,0.903,0.0006796848 59761554,"5","Ckb"
"Actb",4.47837170488355e-08,0.261751526140139,1,0.998,0.00075514303687 7464,"5","Actb"
"Supt16",4.75249610305674e-08,0.528193149155385,0.544,0.35,0.000801365 892897428,"5","Supt16"
"Capzb1",4.93233468728959e-08,0.367041988806965,0.912,0.882,0.00083169 027497077,"5","Capzb"
"Pcmt1",5.33064553954855e-08,0.523743068612281,0.626,0.434,0.000898853 450878677,"5","Pcmt1"
"Ahsa1",5.33357126854047e-08,0.500089457240895,0.551,0.354,0.000899346 787301294,"5","Ahsa1"
"Slbp1",5.4660268059796e-08,0.736052928517534,0.612,0.437,0.0009216814 4002428,"5","Slbp"
"Cep57",5.50549689227205e-08,0.287027570699793,0.32,0.141,0.0009283368 85974913,"5","Cep57"
"Faf1",5.57341633791993e-08,0.378022745540448,0.381,0.187,0.0009397894 62900058,"5","Faf1"
"Psme3",5.87477407835213e-08,0.355454582701261,0.347,0.164,0.000990604 405091736,"5","Psme3"
"Mdh2",5.87959840313382e-08,0.533015705474565,0.735,0.584,0.0009914178 82736425,"5","Mdh2"
"Hras",5.91387344570672e-08,0.559705328546931,0.49,0.295,0.00099719734 0415067,"5","Hras"
"Hmbs",6.48123385541862e-08,0.339187252554,0.367,0.18,0.00109286565270 069,"5","Hmbs"
"Orc2",6.52046409875772e-08,0.366868553547496,0.286,0.125,0.0010994806 5633253,"5","Orc2"
"Ndufv2",6.92782752654013e-08,0.455567995699856,0.653,0.442,0.00116817 02775252,"5","Ndufv2"
"Apobec3",7.21665302781997e-08,0.524999178994647,0.714,0.573,0.0012168 72033551,"5","Apobec3"
"Snrpa",8.1515054727484e-08,0.395360759298714,0.51,0.293,0.00137450685 281484,"5","Snrpa"
"Ddx1",8.46285071637878e-08,0.315261343754126,0.429,0.233,0.0014270058 8779579,"5","Ddx1"

"Cacybp",8.59649172530518e-08,0.592407936998169,0.571,0.373,0.00144954043472096,"5","Cacybp"
"Sptssa",8.66752735142262e-08,0.535491364167912,0.707,0.593,0.00146151846199688,"5","Sptssa"
"Tsn",8.68635316483307e-08,0.482424203659997,0.66,0.509,0.00146469287065415,"5","Tsn"
"G3bp11",8.82171795697359e-08,0.466872698193,0.748,0.626,0.00148751808190489,"5","G3bp1"
"Chchd3",9.13546375422115e-08,0.420536653854628,0.456,0.247,0.00154042189823677,"5","Chchd3"
"Mtch2",9.73605113501731e-08,0.448636478291232,0.578,0.375,0.00164169294238662,"5","Mtch2"
"Tmed2",9.75566585690901e-08,0.50935710604651,0.816,0.731,0.001645000376792,"5","Tmed2"
"Sumo3",9.84069606093001e-08,0.580425314435243,0.558,0.356,0.00165933816979402,"5","Sumo3"
"Sephs1",1.0166245052526e-07,0.255104700441837,0.259,0.105,0.00171423224075693,"5","Sephs1"
"Mrps26",1.04530734111596e-07,0.379330234171688,0.469,0.267,0.00176259723858973,"5","Mrps26"
"H1f0",1.05392051911878e-07,0.634022853265048,0.381,0.202,0.00177712077933809,"5","H1f0"
"Cox5a",1.07084020709402e-07,0.524140906826011,0.741,0.618,0.00180565075720194,"5","Cox5a"
"Nabp2",1.07804030870884e-07,0.468271976642392,0.408,0.213,0.00181779156854484,"5","Nabp2"
"Cuedc21",1.09514006021333e-07,0.474961503799561,0.68,0.517,0.00184662516953171,"5","Cuedc2"
"Vegfb",1.28471859849683e-07,0.540727137169643,0.517,0.317,0.00216629250078536,"5","Vegfb"
"Cmpk1",1.32347685612822e-07,0.605226756292477,0.748,0.589,0.0022316466748034,"5","Cmpk1"
"Tcp1",1.34140194538759e-07,0.507181239247229,0.673,0.557,0.00226187196031256,"5","Tcp1"
"Psmd8",1.41229926058164e-07,0.596665599748653,0.789,0.676,0.00238141901319277,"5","Psmd8"
"Pgls",1.49793431723833e-07,0.460738475160713,0.81,0.671,0.00252581684572728,"5","Pgls"
"Nop16",1.52277969811226e-07,0.32309644642405,0.306,0.14,0.0025677111269569,"5","Nop16"
"Hdac3",1.57982768486116e-07,0.316409308168083,0.395,0.201,0.00266390544221289,"5","Hdac3"
"Dpy30",1.62842392954934e-07,0.449398398279188,0.395,0.213,0.00274584843000609,"5","Dpy30"
"Rtraf",1.93314814499761e-07,0.616299309129659,0.796,0.675,0.00325967440209497,"5","Rtraf"
"Ap1s1",2.02350078433705e-07,0.452968607951558,0.483,0.277,0.00341202702254913,"5","Ap1s1"
"Mtx2",2.16599890819915e-07,0.37146088947787,0.469,0.258,0.0036523073590054,"5","Mtx2"

"Ptges3",2.18769880825537e-07,0.536686310558913,0.721,0.577,0.00368889773048021,"5","Ptges3"
"Naa20",2.20042780979992e-07,0.480801249988781,0.537,0.336,0.00371036137288462,"5","Naa20"
"Rwdd1",2.2179122233873e-07,0.516436234199795,0.537,0.334,0.00373984359107567,"5","Rwdd1"
"Mrpl15",2.29035353328938e-07,0.317893885002255,0.469,0.258,0.00386199412783255,"5","Mrpl15"
"Actl6a",2.33277051302805e-07,0.468251375852375,0.429,0.24,0.00393351763906789,"5","Actl6a"
"Srsf21",2.38383761020438e-07,0.372281665551424,0.844,0.774,0.00401962697832663,"5","Srsf2"
"Mrps17",2.45925353217606e-07,0.421260549658038,0.51,0.298,0.00414679330595528,"5","Mrps17"
"Gemin7",2.49526021603708e-07,0.507481331652795,0.361,0.186,0.00420750777628173,"5","Gemin7"
"Tusc3",2.54158747511218e-07,0.367941683411838,0.381,0.201,0.00428562480053415,"5","Tusc3"
"Lpl",2.65481010427957e-07,0.413036600589415,0.633,0.437,0.00447654079783621,"5","Lpl"
"Cars2",2.7086284852722e-07,0.351347645543068,0.299,0.139,0.00456728935186598,"5","Cars2"
"Ap2s11",2.8389352878779e-07,0.557449233854277,0.653,0.467,0.00478701268241971,"5","Ap2s1"
"Sdf2l1",3.08733058818569e-07,0.42498415794116,0.701,0.528,0.00520585683779871,"5","Sdf2l1"
"Ak6",3.16540048427353e-07,0.384919023343821,0.306,0.142,0.00533749829658203,"5","Ak6"
"Ctcf",3.30320544067575e-07,0.676229408029682,0.592,0.414,0.00556986501406745,"5","Ctcf"
"Atp5a1",3.31904451793636e-07,0.572733376103773,0.837,0.806,0.0055965728661443,"5","Atp5a1"
"Me2",3.38022456995189e-07,0.287884994266873,0.313,0.146,0.00569973466985288,"5","Me2"
"Timm22",3.61261063438098e-07,0.34594521073984,0.333,0.165,0.00609158405169321,"5","Timm22"
"Ftsj3",3.66436695687124e-07,0.378161916901214,0.374,0.194,0.00617885556267629,"5","Ftsj3"
"Haus8",3.77185939175024e-07,0.252904845543148,0.313,0.147,0.00636010930636926,"5","Haus8"
"Mpp6",3.78712395219031e-07,0.315310261122745,0.503,0.296,0.0063858484081833,"5","Mpp6"
"Rnf7",3.84287220399537e-07,0.489541816935312,0.769,0.643,0.00647985111037699,"5","Rnf7"
"Psmc5",3.84291043179423e-07,0.474863679135238,0.639,0.462,0.00647991557009143,"5","Psmc5"
"Psmb4",3.9025532039195e-07,0.53483989512979,0.755,0.646,0.00658048521244907,"5","Psmb4"
"Ube2i",4.03710320418768e-07,0.492627920669225,0.789,0.689,0.00680736342290126,"5","Ube2i"

"Aimp2",4.08782914825624e-07,0.286001863507367,0.259,0.112,0.006892897
50978967,"5","Aimp2"
"Mad2l2",4.170757138387e-07,0.260852352538014,0.272,0.121,0.0070327306
8674816,"5","Mad2l2"
"Dtnbp11",4.26995229452554e-07,0.601879067465799,0.605,0.44,0.00719999
355902897,"5","Dtnbp1"
"Dhrs4",4.36825863487136e-07,0.404192838946669,0.333,0.165,0.007365757
71012009,"5","Dhrs4"
"Polr2m",4.57527067737355e-07,0.413313205367139,0.469,0.271,0.00771482
141618729,"5","Polr2m"
"Srm",4.58700780368725e-07,0.460276555382654,0.442,0.258,0.00773461255
857745,"5","Srm"
"Selenof1",4.75822288493714e-07,0.380094948373321,0.844,0.859,0.008023
315428581,"5","Selenof"
"Agpat5",4.87619250053549e-07,0.296196705728417,0.415,0.223,0.00822223
579440294,"5","Agpat5"
"Sgf29",4.95355107746403e-07,0.391830253343297,0.306,0.148,0.008352677
82681985,"5","Sgf29"
"Gart",5.19470959105422e-07,0.277743000017651,0.395,0.208,0.0087593193
1243562,"5","Gart"
"Tlnrd1",5.41727852102059e-07,0.298664551618145,0.367,0.185,0.00913461
504214491,"5","Tlnrd1"
"Ppp1r7",5.44710290060309e-07,0.462332440862938,0.429,0.247,0.00918490
491099693,"5","Ppp1r7"
"Ndufab1",5.54344858860552e-07,0.590963135202838,0.605,0.4,0.009347363
01010662,"5","Ndufab1"
"Ldha1",5.62680477035434e-07,0.509669226709031,0.83,0.803,0.0094879182
0377149,"5","Ldha"
"Hnrnpr",5.81889823757221e-07,0.474640694236148,0.578,0.377,0.00981182
620819425,"5","Hnrnpr"
"Xpnpep11",5.848693021044e-07,0.359082547668676,0.435,0.244,0.00986206
617208439,"5","Xpnpep1"
"Drg1",6.09120542906498e-07,0.517500507455213,0.422,0.243,0.0102709905
944894,"5","Drg1"
"Emp3",6.15220840082313e-07,0.455769641857223,0.714,0.564,0.0103738538
05468,"5","Emp3"
"Acat11",6.34966056553115e-07,0.460596516264576,0.497,0.329,0.01070679
76455986,"5","Acat1"
"Mdh1",6.48318604610683e-07,0.545205887104504,0.728,0.585,0.0109319483
109453,"5","Mdh1"
"Arl6ip4",6.73665962901852e-07,0.496315175322079,0.483,0.281,0.0113593
55466451,"5","Arl6ip4"
"Eif3i",7.13721246118984e-07,0.563634851992439,0.776,0.648,0.012034767
6520583,"5","Eif3i"
"Smc6",7.19358900520974e-07,0.528544705777992,0.537,0.378,0.0121298297
805847,"5","Smc6"
"Rnf126",7.2150380024531e-07,0.389010738527643,0.422,0.231,0.012165997
0797364,"5","Rnf126"
"Rcc2",7.60323448131366e-07,0.348088029168446,0.476,0.272,0.0128205739
823911,"5","Rcc2"

"Stard7",8.0268965495491e-07,0.474372603415668,0.279,0.131,0.013534952
9618497,"5","Stard7"
"Fam162a",8.41838296273621e-07,0.519366033384611,0.374,0.196,0.0141950
773517658,"5","Fam162a"
"Atp5g3",8.42520485678404e-07,0.516435962167989,0.639,0.459,0.01420658
04295092,"5","Atp5g3"
"Snx31",8.4659696277986e-07,0.549425424879116,0.81,0.758,0.01427531798
6394,"5","Snx3"
"Rack11",8.80797265506239e-07,0.329123444639195,0.986,0.977,0.01485200
34909662,"5","Rack1"
"Odf2",8.81830094431106e-07,0.390354340495309,0.32,0.159,0.01486941905
22973,"5","Odf2"
"Pds5b",8.88008170285082e-07,0.275431044007361,0.313,0.155,0.014973593
767347,"5","Pds5b"
"Donson",9.06050283201838e-07,0.301723436904118,0.293,0.141,0.01527781
98753494,"5","Donson"
"Ddx19a",9.33174145798223e-07,0.302083647350481,0.333,0.166,0.01573518
24464496,"5","Ddx19a"
"Vdac21",1.05158729754787e-06,0.55661709345268,0.871,0.881,0.017731865
0112521,"5","Vdac2"
"Psmb6",1.05862659331106e-06,0.555159958573089,0.769,0.64,0.0178505616
16411,"5","Psmb6"
"Vps29",1.08089461243536e-06,0.665837256588767,0.694,0.506,0.018226044
954885,"5","Vps29"
"Ebna1bp2",1.08545316360889e-06,0.500396690815546,0.456,0.27,0.0183029
112447731,"5","Ebna1bp2"
"Uchl3",1.1032664845075e-06,0.32957502502052,0.422,0.224,0.01860327946
17654,"5","Uchl3"
"Pnp",1.14216530687151e-06,0.445810509420214,0.864,0.815,0.01925919140
44674,"5","Pnp"
"Ppib",1.15754313870778e-06,0.395011884536769,0.878,0.872,0.0195184924
048906,"5","Ppib"
"Cdkn2aipnl",1.16730328892758e-06,0.258679785176932,0.265,0.121,0.0196
830680578968,"5","Cdkn2aipnl"
"Mtch1",1.18280674750476e-06,0.707454419188832,0.762,0.628,0.019944487
3764252,"5","Mtch1"
"Cbfb",1.26063083386026e-06,0.482990590672132,0.49,0.306,0.02125675712
05517,"5","Cbfb"
"Tial1",1.32983698954277e-06,0.385860452885162,0.571,0.377,0.022423711
3176702,"5","Tial1"
"Arpc4",1.34379195846287e-06,0.435127686082814,0.864,0.839,0.022659020
0036009,"5","Arpc4"
"Sh3bgrl1",1.4291094124416e-06,0.434165041029088,0.422,0.231,0.0240976
429125903,"5","Sh3bgrl"
"Prelid3b",1.47317108590212e-06,0.527993050324947,0.483,0.322,0.024840
6108504816,"5","Prelid3b"
"Cdc123",1.5896636616201e-06,0.485690101588834,0.435,0.258,0.026804908
6622381,"5","Cdc123"
"Tsfm",1.61783341766904e-06,0.312140258717512,0.306,0.15,0.02727990708
87354,"5","Tsfm"

"Ndufb11",1.63577283503554e-06,0.685426601342318,0.673,0.523,0.0275824015443693,"5","Ndufb11"
"Sf3b6",1.64000260592031e-06,0.454415106420934,0.653,0.479,0.0276537239410283,"5","Sf3b6"
"Arl2",1.65663376797335e-06,0.289378696017703,0.299,0.143,0.0279341585955666,"5","Arl2"
"Eef1g",1.77446728629349e-06,0.480754825148964,0.789,0.799,0.0299210673814809,"5","Eef1g"
"Rpl7l1",1.79494888463086e-06,0.41378609407547,0.571,0.386,0.0302664280926456,"5","Rpl7l1"
"Rap1a",1.83066132066598e-06,0.419645483040063,0.864,0.81,0.0308686111890698,"5","Rap1a"
"Galk1",1.874510030587e-06,0.26225843767349,0.401,0.22,0.0316079881357581,"5","Galk1"
"Clta2",1.93339152189955e-06,0.346987304706569,0.98,0.958,0.0326008478422703,"5","Clta"
"Cpd",2.08420838648655e-06,0.477055269409539,0.578,0.433,0.0351439218129362,"5","Cpd"
"Tmem109",2.16434074221078e-06,0.676137106114445,0.578,0.424,0.0364951135951582,"5","Tmem109"
"Bcl7c",2.16843452652389e-06,0.465083044150455,0.442,0.26,0.0365641429862458,"5","Bcl7c"
"Nsmce2",2.17245537024203e-06,0.261835367693392,0.408,0.233,0.0366319424530211,"5","Nsmce2"
"Vbp1",2.27965171724109e-06,0.479101804826582,0.497,0.318,0.0384394872561192,"5","Vbp1"
"Timm8a1",2.37154019955957e-06,0.452804748927295,0.286,0.144,0.0399889108449735,"5","Timm8a1"
"Pdcl3",2.52433031107012e-06,0.467433622794463,0.619,0.417,0.0425652577052644,"5","Pdcl3"
"Pde6d",2.71682330789682e-06,0.3495465036869,0.32,0.165,0.0458110746177561,"5","Pde6d"
"Nop56",2.7255850507072e-06,0.49873188828329,0.558,0.374,0.0459588151250248,"5","Nop56"
"Ttc13",2.76427427008524e-06,0.305253833881697,0.313,0.158,0.0466111927421774,"5","Ttc13"
"Glod4",2.78009566116911e-06,0.382618161929225,0.49,0.29,0.0468779730386335,"5","Glod4"
"Prps2",2.78900536427854e-06,0.39990832771657,0.388,0.222,0.0470282084524647,"5","Prps2"
"Eif6",2.893157089774e-06,0.43764480608092,0.653,0.521,0.0487844148477692,"5","Eif6"
"Osgep",2.90392670942622e-06,0.628546201842289,0.592,0.44,0.048966012174345,"5","Osgep"
"Mrps7",2.97061015494945e-06,0.404367714930662,0.456,0.273,0.0500904284327576,"5","Mrps7"
"Sord",3.1021769063713e-06,0.463077544170133,0.388,0.219,0.0523089069952329,"5","Sord"
"Psmd7",3.15423563983947e-06,0.372953800399274,0.605,0.44,0.0531867213589731,"5","Psmd7"

"Srsf4",3.27245047278533e-06,0.348133637734368,0.354,0.191,0.055180059
8721062,"5","Srsf4"
"Sumo1",3.33280668514018e-06,0.493031369630256,0.714,0.575,0.056197786
3248337,"5","Sumo1"
"Smchd1",3.37296190464004e-06,0.543465207509888,0.599,0.477,0.05687488
36360404,"5","Smchd1"
"Tmed3",3.67768213601879e-06,0.450565183451726,0.714,0.574,0.062013076
1775489,"5","Tmed3"
"Kmt5a",3.71960672374581e-06,0.372727657208998,0.449,0.264,0.062720008
5758018,"5","Kmt5a"
"Cct5",3.74912062689119e-06,0.570027187087321,0.755,0.638,0.0632176720
106393,"5","Cct5"
"Lgals11",3.81748584890313e-06,0.534094094813269,0.34,0.183,0.06437044
63842046,"5","Lgals1"
"Suds3",3.95184030016151e-06,0.488379478062367,0.476,0.291,0.066635931
1413233,"5","Suds3"
"Ptpa",3.96995999575201e-06,0.57228970762145,0.565,0.41,0.066941465448
3704,"5","Ptpa"
"Mrpl42",3.98216212399314e-06,0.445889434410352,0.463,0.288,0.06714721
77347723,"5","Mrpl42"
"Kdelr2",4.13979976131166e-06,0.387638138887792,0.592,0.409,0.06980530
35752372,"5","Kdelr2"
"Cetn3",4.19969563397859e-06,0.428044109325597,0.619,0.456,0.070815267
7801469,"5","Cetn3"
"Gtf3a",4.20738462013428e-06,0.428785052613181,0.463,0.288,0.070944919
4647041,"5","Gtf3a"
"Atic",4.48359573407303e-06,0.379732947330368,0.333,0.18,0.07560239126
79394,"5","Atic"
"Ddah2",4.63960112217284e-06,0.508588104117103,0.429,0.257,0.078232954
1220784,"5","Ddah2"
"Ino80e",4.68931881290073e-06,0.309638181293763,0.442,0.258,0.07907129
38231321,"5","Ino80e"
"Eif3l",4.69893949346226e-06,0.397620155559177,0.619,0.461,0.079233517
7387606,"5","Eif3l"
"Tmem11",4.9080975328043e-06,0.303206881629808,0.388,0.216,0.082760340
5981461,"5","Tmem11"
"Blvra1",5.33338016045218e-06,0.3804377825508,0.483,0.303,0.0899314562
655446,"5","Blvra"
"Stk16",5.3381133392374e-06,0.339515270198339,0.327,0.172,0.0900112671
26221,"5","Stk16"
"Atp5j",5.51760127179296e-06,0.469332827139865,0.735,0.629,0.093037792
6449728,"5","Atp5j"
"Pih1d1",5.54761924291898e-06,0.478346247032116,0.449,0.269,0.09354395
56740998,"5","Pih1d1"
"Stx121",5.69062336388004e-06,0.274413844927361,0.503,0.305,0.09595529
11617453,"5","Stx12"
"Commd1",5.6957594692498e-06,0.353884420210481,0.531,0.347,0.096041896
17049,"5","Commd1"
"Cltb",5.80166512066204e-06,0.335357143955846,0.476,0.296,0.0978276772
646033,"5","Cltb"

"Cnbp",5.8225107812289e-06,0.462699948284093,0.782,0.755,0.0981791767930818,"5","Cnbp"
"Mbd3",6.0034926337743e-06,0.455556772873925,0.646,0.513,0.101230892790702,"5","Mbd3"
"Psma3",6.06487047528281e-06,0.428817134819383,0.803,0.688,0.102265845954219,"5","Psma3"
"Smarcb1",6.14855416982748e-06,0.354443623255254,0.449,0.266,0.103676920411631,"5","Smarcb1"
"Psmb2",6.22022388443568e-06,0.565306619353096,0.701,0.631,0.104885415139354,"5","Psmb2"
"Babam1",6.22039485161071e-06,0.487869848702931,0.388,0.23,0.10488829798786,"5","Babam1"
"Fcrls1",6.2225972607335e-06,0.295303493976792,0.905,0.885,0.104925435010488,"5","Fcrls"
"Eif4a3",6.43143249152546e-06,0.655683315504364,0.599,0.452,0.108446814672102,"5","Eif4a3"
"Psmd3",6.68656220476133e-06,0.286093656473261,0.585,0.399,0.112748811896686,"5","Psmd3"
"Ppm1g",6.7172983965222e-06,0.361834812507125,0.551,0.387,0.113267085562157,"5","Ppm1g"
"Ndufa10",6.73892374469813e-06,0.533554113067124,0.585,0.404,0.1136317321831,"5","Ndufa10"
"Nhp2",6.76016891703688e-06,0.404055020708053,0.497,0.323,0.113989968279076,"5","Nhp2"
"Dctpp1",6.83549413714838e-06,0.374052803189225,0.401,0.234,0.115260102140596,"5","Dctpp1"
"Vdac1",6.92528456421814e-06,0.503404614572436,0.687,0.544,0.116774148321846,"5","Vdac1"
"Fmr1",6.93772762443118e-06,0.287183587271925,0.415,0.233,0.116983963203159,"5","Fmr1"
"Gipc11",7.06717604079109e-06,0.427395330039149,0.571,0.393,0.119166722399819,"5","Gipc1"
"Rheb",7.09056298587396e-06,0.41706623687968,0.769,0.723,0.119561073067807,"5","Rheb"
"Etfb",7.1403969011467e-06,0.508860186406313,0.558,0.405,0.120401372547136,"5","Etfb"
"Cenpx",7.14472620852009e-06,0.620117125842644,0.578,0.392,0.120474373328066,"5","Cenpx"
"Commd10",7.20643926331447e-06,0.28380376822585,0.34,0.183,0.121514978858009,"5","Commd10"
"Hsp90b11",7.35023558014447e-06,0.305175822243902,0.816,0.865,0.123939672352396,"5","Hsp90b1"
"Cct4",7.40557319610472e-06,0.404286053312726,0.741,0.614,0.124872775232718,"5","Cct4"
"Tpi11",7.4565920179202e-06,0.460974418696949,0.728,0.586,0.12573305460617,"5","Tpi1"
"Psmb1",7.53761684336444e-06,0.424296779239999,0.83,0.716,0.127099295212811,"5","Psmb1"
"Eef1d",7.86328925833924e-06,0.431918790882932,0.844,0.751,0.132590783474116,"5","Eef1d"

"H3f3b1",8.03818587380134e-06,0.383902826078659,0.993,0.975,0.135539890204038,"5","H3f3b"
"Idh21",8.27795613990941e-06,0.545543824953533,0.605,0.439,0.139582896431152,"5","Idh2"
"C1d",8.7806934395869e-06,0.450806633558647,0.544,0.345,0.148060052778314,"5","C1d"
"Ctbp1",8.83522041328535e-06,0.527062905028954,0.639,0.488,0.148979486608817,"5","Ctbp1"
"Sf3b4",9.24295469416094e-06,0.455999808210486,0.429,0.267,0.155854702052942,"5","Sf3b4"
"Rpl81",9.44887838905259e-06,0.283886779805981,0.986,0.975,0.159326987396205,"5","Rpl8"
"Usp39",1.01949581168743e-05,0.271349720693542,0.293,0.148,0.171907383766734,"5","Usp39"
"Sdhb",1.03457824571256e-05,0.475425479622021,0.673,0.539,0.174450583792052,"5","Sdhb"
"Usp14",1.07017240363302e-05,0.379777563923536,0.497,0.339,0.1804524707006,"5","Usp14"
"Txn1",1.07410231294923e-05,0.568131479011317,0.619,0.461,0.1811151320095,"5","Txn1"
"Tada1",1.10776719281237e-05,0.304589242553843,0.279,0.142,0.186791704052023,"5","Tada1"
"Nme1",1.12825083511675e-05,0.60917952691616,0.612,0.474,0.190245655817386,"5","Nme1"
"Pdzd11",1.17198544707633e-05,0.458903740532959,0.374,0.206,0.197620186086011,"5","Pdzd11"
"Ssr2",1.18966080753334e-05,0.417498254212578,0.68,0.559,0.200600605366271,"5","Ssr2"
"Ccnd11",1.2483102201464e-05,0.452373316655344,0.51,0.332,0.210490069321087,"5","Ccnd1"
"Nsun2",1.26068656835404e-05,0.385594002809892,0.483,0.3,0.212576969155858,"5","Nsun2"
"Gpaa1",1.26146521532224e-05,0.316343285152495,0.531,0.357,0.212708264607635,"5","Gpaa1"
"Cops3",1.26313656985602e-05,0.263307090721597,0.408,0.244,0.212990088409122,"5","Cops3"
"Ech1",1.26392458648293e-05,0.499798403542124,0.667,0.495,0.213122963772752,"5","Ech1"
"Cops6",1.28747314037981e-05,0.50097440640236,0.578,0.415,0.217093720930844,"5","Cops6"
"Tyw1",1.31629360800643e-05,0.490682648046646,0.313,0.167,0.221953428182045,"5","Tyw1"
"Adrm1",1.3247130315562e-05,0.434613158029458,0.605,0.41,0.223373111381007,"5","Adrm1"
"Rab24",1.33397426085185e-05,0.441378687279975,0.605,0.436,0.224934739864839,"5","Rab24"
"Snf8",1.34896265287362e-05,0.466861936796984,0.551,0.382,0.227462082527549,"5","Snf8"
"Exosc9",1.36359644257541e-05,0.300487010946425,0.313,0.164,0.229929632147065,"5","Exosc9"

"Lmna2",1.3868716485644e-05,0.311369088262968,0.408,0.249,0.233854297380928,"5","Lmna"
"Ranbp3",1.39874266398868e-05,0.33423848416518,0.327,0.175,0.235855988001771,"5","Ranbp3"
"Triap1",1.40612966656879e-05,0.50093555117123,0.34,0.184,0.237101584376829,"5","Triap1"
"Hccs1",1.44933386820822e-05,0.357994826449408,0.401,0.227,0.24438667685727,"5","Hccs"
"Slc25a4",1.46720639778099e-05,0.487271795628623,0.769,0.67,0.247400342793831,"5","Slc25a4"
"Hspa81",1.4883225877108e-05,0.326340649136459,0.98,0.976,0.250960954739795,"5","Hspa8"
"Tbcb",1.5283808827336e-05,0.481925932705942,0.626,0.455,0.25771558444654,"5","Tbcb"
"Serpinb6a1",1.56190862374824e-05,0.482607150832312,0.313,0.172,0.263369032136428,"5","Serpinb6a"
"Espl1",1.60478674669411e-05,0.288071711403871,0.279,0.14,0.270599141227561,"5","Espl1"
"Ube2d2a",1.61058056435303e-05,0.470089005808962,0.762,0.681,0.271576094761207,"5","Ube2d2a"
"Acbd6",1.66272114076237e-05,0.399141753312409,0.531,0.334,0.28036803875535,"5","Acbd6"
"Ube2e31",1.71204536466003e-05,0.413457070995463,0.599,0.454,0.288685089388974,"5","Ube2e3"
"C1qbp",1.74878555393803e-05,0.44290193779138,0.585,0.434,0.29488022010503,"5","C1qbp"
"Prpf19",1.81379185970934e-05,0.329925190562873,0.524,0.344,0.305841583384189,"5","Prpf19"
"Rad23a",1.82374214559132e-05,0.465342594030674,0.51,0.36,0.307519400589608,"5","Rad23a"
"Dazap11",1.83747327783957e-05,0.439668081873933,0.558,0.388,0.309834744109308,"5","Dazap1"
"Hars",1.85402532673823e-05,0.299602380629203,0.367,0.204,0.3126257505946,"5","Hars"
"Eif4e",1.86945034236831e-05,0.511154231491685,0.585,0.431,0.315226716730145,"5","Eif4e"
"Brix1",1.88550360757826e-05,0.398967760577675,0.313,0.173,0.317933618309847,"5","Brix1"
"Stoml2",1.8878806985088e-05,0.483021230201134,0.469,0.292,0.318334443382553,"5","Stoml2"
"Mettl9",1.90550501015351e-05,0.351297703657236,0.497,0.338,0.321306254812086,"5","Mettl9"
"Trim28",1.94804571726245e-05,0.363757297242726,0.524,0.385,0.328479468844794,"5","Trim28"
"Snu13",1.97105707334018e-05,0.535545101095916,0.66,0.538,0.332359643706622,"5","Snu13"
"Naxe",1.97294045374915e-05,0.574940231360735,0.524,0.355,0.332677219311182,"5","Naxe"
"Naca1",2.00400361967574e-05,0.321202936106566,0.939,0.918,0.337915090349724,"5","Naca"

"Mff",2.01000880454881e-05,0.522930207983209,0.558,0.393,0.33892768462
3021,"5","Mff"
"Hist1h1c",2.03327719873373e-05,0.452806877984345,0.306,0.172,0.342851
201250481,"5","Hist1h1c"
"Imp41",2.05535892753838e-05,0.310718792374175,0.483,0.308,0.346574622
361521,"5","Imp4"
"Isoc1",2.07754974543534e-05,0.342093011743036,0.306,0.16,0.3503164380
75307,"5","Isoc1"
"Eny2",2.0829426032852e-05,0.502376341082124,0.422,0.258,0.35122578176
595,"5","Eny2"
"Phf5a",2.19677503594473e-05,0.44212769715288,0.476,0.328,0.3704202065
61,"5","Phf5a"
"2310011J03Rik1",2.19769131865264e-05,0.47549100148116,0.422,0.276,0.3
70574710151208,"5","2310011J03Rik"
"Lage3",2.24581690492613e-05,0.273731098888333,0.388,0.219,0.378689646
508644,"5","Lage3"
"Ddb1",2.27082208818489e-05,0.395959734542837,0.531,0.333,0.3829060205
09735,"5","Ddb1"
"Atp5d",2.33752914246701e-05,0.447237815685469,0.823,0.756,0.394154164
002787,"5","Atp5d"
"Phf11b1",2.48756991855428e-05,0.624022797910757,0.374,0.219,0.4194540
39666622,"5","Phf11b"
"Rexo2",2.49657033835028e-05,0.332741410753231,0.551,0.379,0.420971690
452625,"5","Rexo2"
"Psma2",2.50572374543256e-05,0.425041522011458,0.728,0.644,0.422515137
954838,"5","Psma2"
"Glrx3",2.67684637183312e-05,0.395932643218901,0.646,0.49,0.4513698352
18502,"5","Glrx3"
"Rer1",2.68149746844859e-05,0.491402293604491,0.68,0.606,0.45215410312
9802,"5","Rer1"
"Trabd",2.81084828730083e-05,0.298576326843297,0.422,0.257,0.473965238
204665,"5","Trabd"
"Gtf2a2",2.93712376969138e-05,0.489123531601345,0.531,0.379,0.49525781
004536,"5","Gtf2a2"
"Ilf3",2.96726152290779e-05,0.295567335313402,0.408,0.245,0.5003396379
92712,"5","Ilf3"
"Hmgxb4",2.96934828867144e-05,0.2519987566143,0.259,0.129,0.5006915084
35778,"5","Hmgxb4"
"Gltp",3.11284862941326e-05,0.469457210838805,0.748,0.673,0.5248885358
91664,"5","Gltp"
"Pebp1",3.13171945400399e-05,0.408307047848841,0.782,0.684,0.528070534
334153,"5","Pebp1"
"Yif1b",3.15141278529042e-05,0.457710826847581,0.605,0.44,0.5313912238
55671,"5","Yif1b"
"Slc25a39",3.15238665092064e-05,0.44064665916458,0.531,0.375,0.5315554
37078238,"5","Slc25a39"
"Mrpl58",3.29413556341689e-05,0.276041589153278,0.388,0.227,0.55545713
8703356,"5","Mrpl58"
"Qdpr",3.30398763558229e-05,0.383794065279599,0.463,0.286,0.5571183951
11885,"5","Qdpr"

"Fam3c",3.4858246841335e-05,0.299500452617412,0.429,0.26,0.58777975823
8591,"5","Fam3c"
"Nubp2",3.55778327911894e-05,0.439999232183242,0.374,0.227,0.599913416
525035,"5","Nubp2"
"Bccip",3.56849975718013e-05,0.312558101351008,0.401,0.243,0.601720429
055714,"5","Bccip"
"Nfyb",3.64524493730245e-05,0.480283650050477,0.279,0.15,0.61466120132
794,"5","Nfyb"
"Tcerg1",3.79222070939179e-05,0.265838989920385,0.401,0.25,0.639444256
017643,"5","Tcerg1"
"Slc25a31",3.83471587348846e-05,0.306510895978004,0.905,0.911,0.646609
790587625,"5","Slc25a3"
"Larp7",3.86886489666592e-05,0.261014782299294,0.381,0.22,0.6523679988
75808,"5","Larp7"
"Vps351",3.89246773698068e-05,0.387940512850737,0.653,0.535,0.65634790
9809682,"5","Vps35"
"Mzt1",3.91476156182488e-05,0.390665833724866,0.279,0.155,0.6601070945
54911,"5","Mzt1"
"Nudt19",3.94266538824525e-05,0.34733110301021,0.272,0.144,0.664812237
765913,"5","Nudt19"
"Zcchc17",3.95610013724646e-05,0.32597144105904,0.374,0.219,0.66707760
5142497,"5","Zcchc17"
"Fopnl",4.15794870677855e-05,0.271129579276976,0.265,0.141,0.701113310
936999,"5","Fopnl"
"Sept7",4.28134253992181e-05,0.404738276980077,0.701,0.645,0.721919979
081615,"5","Sept7"
"Bud23",4.30488798372766e-05,0.433794048653143,0.347,0.205,0.725890211
816158,"5","Bud23"
"Ndufa8",4.33420060194632e-05,0.449583927470913,0.646,0.483,0.73083290
5500188,"5","Ndufa8"
"Mrpl4",4.40198247463062e-05,0.312664079062797,0.456,0.3,0.74226228487
2215,"5","Mrpl4"
"Pole4",4.42602703173532e-05,0.457713320443097,0.476,0.309,0.746316678
09121,"5","Pole4"
"Trim27",4.47622912396416e-05,0.36231819835449,0.531,0.365,0.754781754
882836,"5","Trim27"
"Hspa41",4.48942684338935e-05,0.443472344877304,0.714,0.616,0.75700715
4332313,"5","Hspa4"
"Mettl16",4.52876229322002e-05,0.299476722649566,0.252,0.128,0.7636398
9788276,"5","Mettl16"
"Lsm1",4.59705947632886e-05,0.291691388090886,0.327,0.182,0.7751561688
98572,"5","Lsm1"
"Necap2",4.61821482467525e-05,0.353805914548828,0.755,0.639,0.77872338
373674,"5","Necap2"
"Cnih1",4.6955461537229e-05,0.463407911795193,0.442,0.291,0.7917629924
40755,"5","Cnih1"
"Gusb",4.70956290666531e-05,0.30222627266982,0.776,0.806,0.79412649732
1904,"5","Gusb"
"Rnpep",4.73092643964494e-05,0.463118399293157,0.619,0.466,0.797728816
25293,"5","Rnpep"

"E2f4",4.98030982899032e-05,0.252694788916328,0.395,0.239,0.839779843364348,"5","E2f4"
"Oat",5.08498092998077e-05,0.420742740808746,0.544,0.378,0.857429484413357,"5","Oat"
"Pigt",5.39208702377699e-05,0.277802846608856,0.503,0.34,0.909213713949276,"5","Pigt"
"Nip7",5.48130876070911e-05,0.252479834303759,0.32,0.18,0.92425828323077,"5","Nip7"
"Pbdc1",5.88770923460818e-05,0.364748314576991,0.395,0.237,0.992785531139631,"5","Pbdc1"
"Atp5o.1",5.94840275940966e-05,0.525988296597531,0.626,0.502,1,"5","Atp5o.1"
"Psma41",6.06861065025998e-05,0.424000092215534,0.673,0.57,1,"5","Psma4"
"Cst71",6.08876217803854e-05,0.330275996221636,0.374,0.223,1,"5","Cst7"
"Glipr1",6.2369819694767e-05,0.37843319325139,0.517,0.364,1,"5","Glipr1"
"Vrk1",6.62790919477294e-05,0.362819548409152,0.517,0.356,1,"5","Vrk1"
"Psmb71",6.95583103903238e-05,0.346656311720418,0.612,0.467,1,"5","Psmb7"
"Carnmt1",7.18552073680698e-05,0.309961263824317,0.265,0.14,1,"5","Carnmt1"
"Gpx1",7.81146522069338e-05,0.311896684462817,0.925,0.909,1,"5","Gpx1"
"Tubb61",7.82262514757209e-05,0.315088199550566,0.401,0.241,1,"5","Tubb6"
"Coa3",7.82399621804461e-05,0.46391546023733,0.517,0.349,1,"5","Coa3"
"Sec13",7.84253789646104e-05,0.284237502135329,0.524,0.352,1,"5","Sec13"
"Myg1",8.32159989003251e-05,0.312688044895972,0.327,0.191,1,"5","Myg1"
"Psmd6",8.60075269570874e-05,0.368591175469077,0.558,0.418,1,"5","Psmd6"
"Rad50",8.67445526144954e-05,0.353849472576451,0.415,0.258,1,"5","Rad50"
"Brcc3",8.72549150232217e-05,0.256114898972853,0.252,0.131,1,"5","Brcc3"
"Eef1b21",8.79014496398151e-05,0.342420682645602,0.946,0.923,1,"5","Eef1b2"
"Hspd1",9.21951668684232e-05,0.418547978944617,0.619,0.495,1,"5","Hspd1"
"Psmc6",9.41061759802557e-05,0.360878527274012,0.626,0.523,1,"5","Psmc6"
"Rab5c",9.54159717687166e-05,0.441701568189686,0.769,0.719,1,"5","Rab5c"
"Ctps2",9.89592149195727e-05,0.314638343282952,0.395,0.233,1,"5","Ctps2"
"Sdhd",0.000106240141289739,0.379305529068217,0.585,0.45,1,"5","Sdhd"
"Ndufs3",0.000106312290696828,0.625408944920044,0.612,0.47,1,"5","Ndufs3"
"Psmc3",0.000112797237422999,0.467014348277485,0.667,0.569,1,"5","Psmc

3"
"Stt3b",0.000115820309226187,0.28913458992938,0.469,0.308,1,"5","Stt3b
"
"Bin1",0.000116352316363229,0.275893490427518,0.925,0.879,1,"5","Bin1"
"Mrpl20",0.000117715410823477,0.465862624451901,0.497,0.354,1,"5","Mrp
l20"
"Ndufs8",0.000121458344086156,0.415484363431474,0.565,0.438,1,"5","Ndu
fs8"
"Stim1",0.000124697033324614,0.252235853525278,0.354,0.206,1,"5","Stim
1"
"Psma7",0.000125408415618529,0.376192001246436,0.755,0.657,1,"5","Psma
7"
"Ubald2",0.000129014081350261,0.488409755947745,0.544,0.439,1,"5","Uba
ld2"
"Ola1",0.000130709022377895,0.314881491434052,0.537,0.397,1,"5","Ola1"
"Ctsc2",0.000131878036730433,0.252937962828458,0.857,0.892,1,"5","Ctsc
"
"Ndufb5",0.000132186446577058,0.317386314413814,0.701,0.521,1,"5","Ndu
fb5"
"2610507B11Rik1",0.000136443204090513,0.343560119610683,0.449,0.302,1,
"5","2610507B11Rik"
"Tubb2a2",0.000139743223973602,0.49220676343767,0.68,0.55,1,"5","Tubb2
a"
"Sf3b2",0.000140257762060438,0.414672006057218,0.714,0.661,1,"5","Sf3b
2"
"Rdx",0.000144523614584097,0.319656833321845,0.633,0.53,1,"5","Rdx"
"Gars",0.000149209890611931,0.269999883503341,0.463,0.299,1,"5","Gars"
"Uba21",0.000150657917227879,0.461855375095937,0.571,0.46,1,"5","Uba2"
"Phb",0.000159895071342588,0.255158612306657,0.408,0.253,1,"5","Phb"
"Rbm17",0.000161047012592632,0.447572020609527,0.463,0.315,1,"5","Rbm1
7"
"Anapc11",0.000167437205050783,0.358301996886212,0.497,0.343,1,"5","An
apc11"
"Naaa",0.000169436817853183,0.406973897492335,0.571,0.429,1,"5","Naaa"
"Taf12",0.000172454794782938,0.278762442795769,0.34,0.205,1,"5","Taf12
"
"Gtf2f1",0.000176225593789187,0.275066608183763,0.361,0.219,1,"5","Gtf
2f1"
"Hdgfl2",0.000176745747127144,0.28227707505175,0.32,0.19,1,"5","Hdgfl2
"
"Cfdp11",0.000181354519660062,0.408303576296238,0.51,0.349,1,"5","Cfdp
1"
"Sms",0.000187227007096869,0.364486939358069,0.354,0.213,1,"5","Sms"
"Btf31",0.000187836777378934,0.29835237908406,0.884,0.901,1,"5","Btf3"
"Glrx5",0.000189331275179583,0.358665384564105,0.497,0.354,1,"5","Glrx
5"
"Grpel1",0.000192570634033115,0.306437728975909,0.476,0.328,1,"5","Grp
el1"
"BC035044",0.000193102210329561,0.378955093634135,0.435,0.287,1,"5","B
C035044"

"Kpnb1",0.00019315592161556,0.427548007199055,0.605,0.469,1,"5","Kpnb1
"
"Smn1",0.000193418223674683,0.321306903555575,0.313,0.184,1,"5","Smn1"
"Exosc7",0.000195166096001542,0.256512493865613,0.327,0.188,1,"5","Exo
sc7"
"Ap2m1",0.000205785534556644,0.409522299891557,0.707,0.614,1,"5","Ap2m
1"
"Fam192a",0.000206259733833069,0.286320988484791,0.388,0.236,1,"5","Fa
m192a"
"Rnf187",0.000207065201052395,0.33703030450985,0.633,0.522,1,"5","Rnf1
87"
"Lsm8",0.000211513142638447,0.283332633008298,0.361,0.224,1,"5","Lsm8"
"Hsd17b4",0.000215540611365167,0.345788015843495,0.66,0.529,1,"5","Hsd
17b4"
"Pfdn6",0.000219924823421934,0.351873324230674,0.374,0.239,1,"5","Pfdn
6"
"Kif2a",0.000223390973898351,0.372018597049264,0.347,0.213,1,"5","Kif2
a"
"Rps51",0.000225811480612076,0.286351043687142,0.986,0.965,1,"5","Rps5
"
"Ssr1",0.000235695156154281,0.396429669099744,0.639,0.53,1,"5","Ssr1"
"Atp5c11",0.000244210072115781,0.343698991659533,0.796,0.777,1,"5","At
p5c1"
"Dnajc2",0.000244304365250756,0.383404022438005,0.49,0.337,1,"5","Dnaj
c2"
"Polr2e",0.000250741005589811,0.308717702398497,0.626,0.464,1,"5","Pol
r2e"
"Phf10",0.000257461916638497,0.319209238722587,0.388,0.239,1,"5","Phf1
0"
"Tcf3",0.000258812458233474,0.354043752385355,0.429,0.277,1,"5","Tcf3"
"Srrt",0.000269358405698973,0.35294968151211,0.408,0.274,1,"5","Srrt"
"Thoc3",0.000270530929225575,0.299532746452598,0.279,0.162,1,"5","Thoc
3"
"Zbtb8os",0.000273404026771001,0.307527878893469,0.313,0.183,1,"5","Zb
tb8os"
"Lsm3",0.000277806571601867,0.33220158484655,0.327,0.195,1,"5","Lsm3"
"Ak2",0.000300327209312855,0.394038062808479,0.565,0.417,1,"5","Ak2"
"Wbp11",0.00031184686736263,0.347912372236087,0.483,0.339,1,"5","Wbp11
"
"Rars",0.000312281278080442,0.402158385090295,0.442,0.295,1,"5","Rars"
"Polr1d",0.000348069820335802,0.354677864432281,0.776,0.68,1,"5","Polr
1d"
"Atp6v1g11",0.000353348728244932,0.36272389074965,0.857,0.838,1,"5","A
tp6v1g1"
"Utp3",0.000357273232519207,0.363935832928063,0.51,0.356,1,"5","Utp3"
"Swi5",0.000359404993220976,0.345517334588719,0.626,0.509,1,"5","Swi5"
"Aurkaip1",0.000381221739329296,0.571116520465502,0.531,0.407,1,"5","A
urkaip1"
"Igfbp41",0.00038317977939017,0.565155509214367,0.429,0.287,1,"5","Igf
bp4"

"Btbd1",0.00039628918991176,0.250650854597382,0.456,0.298,1,"5","Btbd1
"
"Fkbp8",0.000403337622605831,0.384151376455345,0.728,0.62,1,"5","Fkbp8
"
"Ccdc124",0.000411021719479699,0.319489083583834,0.395,0.254,1,"5","Cc
dc124"
"Scoc",0.000428042327227016,0.358951088508687,0.796,0.683,1,"5","Scoc"
"Ndufb10",0.000436489227074684,0.507402734829373,0.626,0.483,1,"5","Nd
ufb10"
"Exosc5",0.000440653987674645,0.446125897209274,0.422,0.289,1,"5","Exo
sc5"
"Txn2",0.00044143370252399,0.415608053314998,0.551,0.397,1,"5","Txn2"
"Tecr",0.000452682046912652,0.400218272734958,0.68,0.6,1,"5","Tecr"
"Tmem165",0.000455850430279114,0.256815665303805,0.503,0.36,1,"5","Tme
m165"
"Pak1ip1",0.000462805082172549,0.36077866731539,0.476,0.336,1,"5","Pak
1ip1"
"Srpk1",0.000463070580661311,0.334359848483117,0.408,0.27,1,"5","Srpk1
"
"Etfa1",0.00046540233920498,0.348062576444009,0.456,0.311,1,"5","Etfa"
"Tkt",0.000468525224515407,0.286191359453166,0.782,0.756,1,"5","Tkt"
"Mrpl17",0.000488448037024393,0.432874357948644,0.51,0.362,1,"5","Mrpl
17"
"Mrpl2",0.000490354581597024,0.412545081619595,0.361,0.237,1,"5","Mrpl
2"
"Aco2",0.000502091149397321,0.362882240259016,0.531,0.412,1,"5","Aco2"
"Rsl1d1",0.000504671377228131,0.404334672624785,0.565,0.466,1,"5","Rsl
1d1"
"Nsmce3",0.000504783776665851,0.268010080236188,0.354,0.221,1,"5","Nsm
ce3"
"Lsm61",0.000506603177528907,0.386938357534899,0.483,0.336,1,"5","Lsm6
"
"Rnaseh2c",0.000512601712242057,0.445247327735185,0.435,0.308,1,"5","R
naseh2c"
"Med28",0.000535469128883319,0.368247999098123,0.517,0.381,1,"5","Med2
8"
"Ipo5",0.000543723740781645,0.288758632198282,0.449,0.306,1,"5","Ipo5"
"Mydgf",0.000547149081723307,0.32468033596641,0.612,0.479,1,"5","Mydgf
"
"Commd91",0.000561554081111899,0.3803892994714,0.449,0.319,1,"5","Comm
d9"
"Thoc6",0.000591795338601661,0.400752630260566,0.32,0.195,1,"5","Thoc6
"
"Samm50",0.000600445283265551,0.381371824590485,0.565,0.416,1,"5","Sam
m50"
"Trir",0.000602413791632094,0.469651802973871,0.755,0.682,1,"5","Trir"
"Mid1ip1",0.000607286182259473,0.302485629887846,0.558,0.407,1,"5","Mi
d1ip1"
"Tmem70",0.00061149643226441,0.390919913989888,0.293,0.174,1,"5","Tmem
70"

"Sec11c",0.000616575710672982,0.364166262049021,0.701,0.643,1,"5","Sec
11c"
"Asna1",0.000621406547809974,0.349022770990436,0.367,0.237,1,"5","Asna
1"
"Rrp1",0.000639591954336662,0.388283305423181,0.626,0.53,1,"5","Rrp1"
"Pdap1",0.000652537515922029,0.424422265485189,0.537,0.389,1,"5","Pdap
1"
"Ccdc115",0.000664289987595829,0.404723709706724,0.449,0.312,1,"5","Cc
dc115"
"Slc35a4",0.000705736419130738,0.2900310481684,0.32,0.204,1,"5","Slc35
a4"
"Ndufa9",0.00071002392856653,0.406530803512649,0.544,0.408,1,"5","Nduf
a9"
"Hspa9",0.00072011238828027,0.371680017831622,0.565,0.448,1,"5","Hspa
9"
"Srp9",0.000729986249092438,0.416576153943623,0.68,0.581,1,"5","Srp9"
"2610001J05Rik",0.000742342969233314,0.364649200531489,0.456,0.32,1,"5
","2610001J05Rik"
"Polr2c1",0.000752420185818042,0.367014415319935,0.463,0.325,1,"5","Po
lr2c"
"Slamf9",0.000781464846660109,0.508063018673112,0.585,0.52,1,"5","Slam
f9"
"Pmvk",0.000785381417482605,0.384837414556877,0.306,0.188,1,"5","Pmvk"
"Nifk",0.000787070173076722,0.283264509828392,0.272,0.162,1,"5","Nifk"
"Tiprl",0.00079649742794694,0.367257594594297,0.456,0.305,1,"5","Tiprl
"
"Ywhab",0.000826821307977707,0.382937112342407,0.735,0.705,1,"5","Ywha
b"
"Hnrnpa01",0.000857538021694465,0.254112121099923,0.85,0.854,1,"5","Hn
rnpa0"
"Lamtor31",0.000870742253183048,0.350607554670673,0.551,0.432,1,"5","L
amtor3"
"Dbi1",0.00087952453069022,0.414661483169884,0.599,0.461,1,"5","Dbi"
"Bax",0.000882814448832793,0.362552404672914,0.673,0.561,1,"5","Bax"
"Eif4h",0.000887334466896848,0.370579800480461,0.782,0.687,1,"5","Eif4
h"
"Mpp11",0.000927703200492742,0.350129852375949,0.442,0.299,1,"5","Mpp1
"
"Suz12",0.00095122623248537,0.320483056358289,0.429,0.316,1,"5","Suz12
"
"Ppp4c",0.0009787880355324,0.35417020322823,0.612,0.492,1,"5","Ppp4c"
"Snrpc",0.000990418637742344,0.260192193210642,0.503,0.355,1,"5","Snrp
c"
"Pop5",0.00100847417273135,0.289149777293878,0.361,0.244,1,"5","Pop5"
"Pilra",0.00102401301201718,0.347549663599599,0.408,0.282,1,"5","Pilra
"
"Eif3g1",0.00104739096063502,0.299959410636531,0.531,0.402,1,"5","Eif3
g"
"Pdia31",0.00105664086541643,0.264242376486258,0.857,0.852,1,"5","Pdia
3"

"Msl3",0.00106337832335877,0.335562128918568,0.333,0.215,1,"5","Msl3"
"Gdi2",0.00106645817306551,0.302606263394368,0.884,0.892,1,"5","Gdi2"
"Psmc1",0.00107177247179423,0.359710667253594,0.442,0.308,1,"5","Psmc1
"
"Tmco1",0.001071863320518,0.315683588064432,0.68,0.601,1,"5","Tmco1"
"Rnps1",0.00109571593901932,0.294718004604228,0.374,0.253,1,"5","Rnps1
"
"Srprb",0.00109595756116828,0.252564008730708,0.333,0.206,1,"5","Srprb
"
"Sod2",0.00110698866342915,0.321167024940124,0.612,0.497,1,"5","Sod2"
"Pdia4",0.00111277618527773,0.367465865533157,0.517,0.375,1,"5","Pdia4
"
"Chpt1",0.00112120742935449,0.267678604286192,0.327,0.205,1,"5","Chpt1
"
"Milr11",0.00117212639141167,0.291648429337061,0.517,0.368,1,"5","Milr
1"
"Arpc5l1",0.00121135688634429,0.292911106833396,0.592,0.472,1,"5","Arp
c5l"
"Tuba1a",0.00122104800012403,0.286088894957743,0.673,0.54,1,"5","Tuba1
a"
"Ndufs2",0.00130984850390719,0.494222450458971,0.633,0.567,1,"5","Nduf
s2"
"Magoh",0.00136208262746975,0.297411330058422,0.422,0.286,1,"5","Magoh
"
"Trappc1",0.00140026817732411,0.452043889785308,0.463,0.335,1,"5","Tra
ppc1"
"Htatsf1",0.00145900010199021,0.265559934813832,0.279,0.169,1,"5","Hta
tsf1"
"Ruvbl1",0.0015117609260107,0.29416584633612,0.34,0.219,1,"5","Ruvbl1"
"Idh3b",0.00152577026248795,0.361257157408591,0.483,0.362,1,"5","Idh3b
"
"1110004F10Rik1",0.00158484706435775,0.430359034789684,0.565,0.442,1,"
5","1110004F10Rik"
"Cxcl161",0.00159756202311111,0.409278412539482,0.626,0.541,1,"5","Cxc
l16"
"Rps27l",0.00159759851188493,0.419631431936722,0.585,0.48,1,"5","Rps27
l"
"Polr1c",0.00164826142846875,0.341527447980902,0.265,0.163,1,"5","Polr
1c"
"Eif3d",0.00171088920382094,0.277153269764345,0.558,0.42,1,"5","Eif3d"
"Ube2n",0.00185496865022008,0.400723623000932,0.68,0.591,1,"5","Ube2n"
"Ruvbl2",0.00189771498222174,0.333984677521859,0.272,0.168,1,"5","Ruvb
l2"
"Ngdn",0.00194865583923318,0.327223570962098,0.374,0.253,1,"5","Ngdn"
"Rbmxl1",0.00196380211417071,0.270880154977748,0.531,0.402,1,"5","Rbmx
l1"
"Srsf1",0.00198810974565193,0.341399167533838,0.415,0.3,1,"5","Srsf1"
"Phb2",0.00201591528826811,0.427782029847393,0.68,0.613,1,"5","Phb2"
"Psmd11",0.00202422235086783,0.301033501296207,0.639,0.528,1,"5","Psmd
11"

"Srp19",0.00204789751194978,0.369715114518421,0.449,0.311,1,"5","Srp19
"
"Pnn",0.00205619247927922,0.453000914919693,0.544,0.421,1,"5","Pnn"
"Setd3",0.00205755537516949,0.35342630557872,0.639,0.53,1,"5","Setd3"
"Polr2g",0.0021199519843134,0.434320490696266,0.503,0.379,1,"5","Polr2
g"
"Gnb1",0.0021470402869456,0.288195131470247,0.782,0.752,1,"5","Gnb1"
"Mrpl9",0.00221105719285758,0.281816633726691,0.306,0.194,1,"5","Mrpl9
"
"Rpn2",0.00227735012688164,0.284035055744937,0.741,0.738,1,"5","Rpn2"
"Rab7",0.00228168492161612,0.296878899915955,0.769,0.737,1,"5","Rab7"
"Blmh",0.00232498584579849,0.261050143203847,0.442,0.308,1,"5","Blmh"
"Atxn10",0.00234967728450531,0.294126792107349,0.626,0.513,1,"5","Atxn
10"
"Utp11",0.00238786821367582,0.339509719493839,0.449,0.335,1,"5","Utp11
"
"Vps72",0.00243072180367581,0.420922994707323,0.333,0.218,1,"5","Vps72
"
"Tmem219",0.00244790065925335,0.271506940744083,0.599,0.476,1,"5","Tme
m219"
"Map2k2",0.00262382644742704,0.382357697371596,0.721,0.646,1,"5","Map2
k2"
"Fam32a",0.00263199215322759,0.323330707374736,0.476,0.34,1,"5","Fam32
a"
"Wtip1",0.00287866691168461,0.320824315934617,0.354,0.241,1,"5","Wtip"
"Pex7",0.0029236865895027,0.318497820224009,0.388,0.257,1,"5","Pex7"
"Mrps15",0.00297303911278628,0.422805698904293,0.565,0.437,1,"5","Mrps
15"
"Ciao1",0.00297586794703884,0.269733750268281,0.293,0.185,1,"5","Ciao1
"
"Uqcrfs1",0.00311796738662054,0.282232285243739,0.633,0.556,1,"5","Uqc
rfs1"
"Myl12a",0.00312938775243568,0.282273513426098,0.83,0.79,1,"5","Myl12a
"
"Dnaja2",0.00316951655491284,0.362245004460806,0.646,0.556,1,"5","Dnaj
a2"
"Timm17a1",0.00333140588803926,0.256790474704142,0.415,0.282,1,"5","Ti
mm17a"
"Tor1a",0.00333944951079677,0.274921002279386,0.469,0.365,1,"5","Tor1a
"
"Mpdu1",0.00336182581381579,0.288770761610882,0.483,0.372,1,"5","Mpdu1
"
"Aldh9a1",0.00350784987829625,0.296544321446202,0.347,0.233,1,"5","Ald
h9a1"
"Fkbp2",0.00368022562840141,0.306942086706298,0.537,0.399,1,"5","Fkbp2
"
"Eif3m",0.00376269698894649,0.565535076005542,0.707,0.617,1,"5","Eif3m
"
"Coq2",0.00380750053481557,0.256838726117685,0.333,0.219,1,"5","Coq2"
"Rhoc",0.00382300983857206,0.325502441930713,0.558,0.437,1,"5","Rhoc"

"Rtcb",0.00403922972700393,0.305960318653593,0.701,0.621,1,"5","Rtcb"
"Cdc37",0.0040993218432038,0.515484813719957,0.707,0.621,1,"5","Cdc37"
"Uqcrc2",0.00412071834593987,0.330460808372373,0.612,0.517,1,"5","Uqcr
c2"
"Eif3h1",0.00420862306025505,0.293213497389595,0.816,0.794,1,"5","Eif3
h"
"Ndufb8",0.00437142191032943,0.340809890812308,0.612,0.507,1,"5","Nduf
b8"
"Akt1",0.00438345739363244,0.340845697049573,0.694,0.624,1,"5","Akt1"
"Hmox2",0.00442079573308311,0.316608501157267,0.66,0.567,1,"5","Hmox2"
"Ccl22",0.0046299045196287,0.493754748573973,0.673,0.623,1,"5","Ccl2"
"Anapc15",0.00463999198968631,0.301734074684202,0.279,0.184,1,"5","Ana
pc15"
"Nagpa",0.00467853576018163,0.281452601781279,0.503,0.369,1,"5","Nagpa
"
"Tomm22",0.0047223141721013,0.418716835625718,0.653,0.556,1,"5","Tomm
22"
"Drap1",0.00480380935469365,0.314440338097058,0.653,0.559,1,"5","Drap1
"
"Syncrip",0.00505510481511136,0.336662812498382,0.626,0.544,1,"5","Syn
crip"
"Ssr3",0.0052327376948648,0.357764859660849,0.551,0.433,1,"5","Ssr3"
"Atg3",0.00538702289060006,0.303391074336363,0.544,0.411,1,"5","Atg3"
"Mrps34",0.00541513572645702,0.330881567665487,0.361,0.255,1,"5","Mrps
34"
"Eif3b1",0.00553765955036731,0.271691876296172,0.497,0.388,1,"5","Eif3
b"
"Tmem183a",0.00579306550921915,0.325636004274565,0.306,0.211,1,"5","Tm
em183a"
"Mrpl57",0.00589066071849284,0.327096040614477,0.354,0.244,1,"5","Mrpl
57"
"Vapb",0.00614951517655541,0.313377916423105,0.49,0.364,1,"5","Vapb"
"Ube2k",0.00618230116165452,0.320935301068916,0.585,0.481,1,"5","Ube2k
"
"BC004004",0.00626069668284147,0.342793990473169,0.442,0.328,1,"5","BC
004004"
"Sf3b5",0.00627221330572436,0.390978842430454,0.49,0.385,1,"5","Sf3b5"
"Slc35b1",0.00638759365955787,0.262924875140974,0.401,0.287,1,"5","Slc
35b1"
"Mapre1",0.00642110904418247,0.27492301502069,0.816,0.787,1,"5","Mapre
1"
"Ube2l3",0.00644526083673152,0.399894899638702,0.707,0.618,1,"5","Ube2
l3"
"Ccz1",0.00647936245194803,0.29105766811649,0.422,0.303,1,"5","Ccz1"
"Sdha",0.00695906985976453,0.361771354058663,0.592,0.483,1,"5","Sdha"
"Vta11",0.00696876774211513,0.289279499124908,0.415,0.31,1,"5","Vta1"
"Taf15",0.00705234449877597,0.433961070827411,0.51,0.378,1,"5","Taf15"
"Ccdc88a",0.00713715385270375,0.259361077319103,0.469,0.355,1,"5","Ccd
c88a"
"Rinl",0.00715334075338471,0.359766339437374,0.34,0.234,1,"5","Rinl"

"Tap1",0.00718628454007399,0.405382045283099,0.463,0.371,1,"5","Tap1"
"Kcmf1",0.00745843929623641,0.263383597617791,0.605,0.471,1,"5","Kcmf1
"
"Mfsd111",0.00753175309495132,0.30302396256676,0.401,0.283,1,"5","Mfsd
11"
"Phf11d1",0.00754550230885346,0.540191805131625,0.259,0.173,1,"5","Phf
11d"
"Sarnp1",0.00756877121316374,0.433257430696703,0.497,0.387,1,"5","Sarn
p"
"Cyb5r3",0.00762741858088481,0.379285321788481,0.435,0.328,1,"5","Cyb5
r3"
"Gde1",0.00763667380696935,0.263268473989271,0.639,0.53,1,"5","Gde1"
"Clec5a",0.0076375836763921,0.479690065121045,0.469,0.361,1,"5","Clec5
a"
"Kpna3",0.00772108520275859,0.318247873347164,0.449,0.339,1,"5","Kpna3
"
"Acsl5",0.00778284320914214,0.305283652577908,0.476,0.369,1,"5","Acsl5
"
"Gyg",0.00779120468383377,0.280286467544781,0.313,0.212,1,"5","Gyg"
"Smarcd2",0.00795219815411389,0.269051015925255,0.361,0.247,1,"5","Sma
rcd2"
"Prkar1a",0.00795323756971498,0.298661811572013,0.667,0.644,1,"5","Prk
ar1a"
"Ube2a",0.00799029017571216,0.292341899069827,0.551,0.407,1,"5","Ube2a
"
"Vapa",0.00809561522825348,0.262372968386986,0.789,0.777,1,"5","Vapa"
"Brk1",0.00824229402953378,0.295542797200997,0.844,0.827,1,"5","Brk1"
"Cd48",0.00852756039351574,0.282144193951502,0.667,0.599,1,"5","Cd48"
"Sri",0.0087227547990397,0.259919506607298,0.599,0.503,1,"5","Sri"
"Commd4",0.00880172799093966,0.35379844524614,0.449,0.359,1,"5","Commd
4"
"Denr",0.00893142493918922,0.36661236943928,0.558,0.433,1,"5","Denr"
"Stk25",0.00898245861889754,0.264208615350787,0.313,0.208,1,"5","Stk25
"
"Arf5",0.00922629802336627,0.250578017887109,0.769,0.759,1,"5","Arf5"
"Derl2",0.00955973772660512,0.265528122540248,0.503,0.398,1,"5","Derl2
"
"Glmp",0.00956723915162704,0.27679993636359,0.714,0.642,1,"5","Glmp"
"Pts",0.00960095836621872,0.356827149489617,0.469,0.345,1,"5","Pts"
"Pycr2",0.0098937307580037,0.254622423704745,0.388,0.285,1,"5","Pycr2"
"Sdhc1",0.00997154558825872,0.383080403820635,0.442,0.349,1,"5","Sdhc"
"Plac8",0,4.35831376940728,0.92,0.012,0,"6","Plac8"
"Napsa",0,3.24470745877453,0.949,0.01,0,"6","Napsa"
"Ifitm6",9.63982997404178e-268,2.70072312383414,0.804,0.013,1.62546813
022293e-263,"6","Ifitm6"
"S100a6",9.44115097066059e-266,3.26743190444711,0.79,0.012,1.591966876
67279e-261,"6","S100a6"
"Plbd1",2.7061079047521e-264,2.22565547705092,0.819,0.016,4.5630391489
9299e-260,"6","Plbd1"
"Sirpb1c",1.28039195854572e-253,1.8288845738064,0.71,0.006,2.158996920

49979e-249,"6","Sirpb1c"
"Adgre5",2.75658588258003e-252,2.70817489219258,0.913,0.035,4.64815511
520645e-248,"6","Adgre5"
"S100a4",2.94469436111857e-250,2.77959686103861,0.754,0.012,4.96534363
171814e-246,"6","S100a4"
"Gda",1.03829712384268e-248,2.07269055908664,0.667,0.003,1.75077661022
353e-244,"6","Gda"
"Ltb4r1",1.59313526551045e-246,2.0233763554727,0.703,0.008,2.686344684
70372e-242,"6","Ltb4r1"
"Cytip",1.70805140037052e-236,2.92163365367226,0.942,0.049,2.880116271
30477e-232,"6","Cytip"
"Hp",2.00592331123828e-221,2.41704120615121,0.594,0.003,3.382387887409
99e-217,"6","Hp"
"Adgre4",3.64824444233475e-220,1.79307037719608,0.638,0.008,6.15166977
866486e-216,"6","Adgre4"
"Klra2",1.36383318002048e-215,1.7091391488539,0.623,0.007,2.2996955081
5054e-211,"6","Klra2"
"Itgb7",1.36390294682267e-212,1.56120781379868,0.58,0.003,2.2998131489
3238e-208,"6","Itgb7"
"Gpr141",1.06750286522535e-211,1.68092678302037,0.659,0.012,1.80002333
134298e-207,"6","Gpr141"
"Tmem51",1.31274250894571e-210,1.5489123203757,0.659,0.012,2.213546418
58425e-206,"6","Tmem51"
"Emb",4.26668705755311e-207,2.1552579323613,0.797,0.034,7.194487716446
06e-203,"6","Emb"
"Itgal",1.57995815701387e-205,2.53787803548593,0.79,0.035,2.6641254443
5678e-201,"6","Itgal"
"Trem3",1.82400952128844e-203,1.18226080559176,0.551,0.003,3.075644854
79657e-199,"6","Trem3"
"Ace",3.95871729007409e-203,1.96314825495676,0.587,0.006,6.67518909452
292e-199,"6","Ace"
"Clec4e",7.56967649071894e-203,2.2005834325177,0.659,0.014,1.276398849
86503e-198,"6","Clec4e"
"Mcemp1",5.76920763484408e-201,1.82445043961138,0.674,0.017,9.72803791
387409e-197,"6","Mcemp1"
"Pglyrp1",6.44480974151525e-198,1.80884487788855,0.58,0.007,1.08672381
86143e-193,"6","Pglyrp1"
"Itga4",4.75471405862446e-195,2.7268587048427,0.87,0.058,8.01739884565
257e-191,"6","Itga4"
"Emilin2",1.08952095207169e-188,2.07266287790593,0.754,0.036,1.8371502
2938329e-184,"6","Emilin2"
"Spn",3.68971786289399e-187,1.51319952103865,0.551,0.007,6.22160226041
184e-183,"6","Spn"
"Ahnak1",1.4138788695594e-181,2.42688111911116,0.775,0.042,2.384082549
85107e-177,"6","Ahnak"
"S100a11",1.25162573269639e-177,2.28085675354972,0.703,0.031,2.1104913
1047265e-173,"6","S100a11"
"Ear2",2.01598813477411e-176,1.82662899056538,0.522,0.006,3.3993591928
561e-172,"6","Ear2"
"Ccr2",5.41592270325072e-176,2.16689215591509,0.558,0.01,9.13232886222

137e-172,"6","Ccr2"
"Gm21188",2.01729650731144e-175,1.44313574707399,0.594,0.015,3.4015653
7062855e-171,"6","Gm21188"
"Gpr132",3.25420091341143e-174,1.50444349325435,0.732,0.037,5.48723358
019435e-170,"6","Gpr132"
"Treml4",3.40367145228269e-170,1.66403115161664,0.587,0.016,5.73927080
283908e-166,"6","Treml4"
"Anxa1",4.70262145539782e-166,1.69404221215783,0.522,0.009,7.929560298
09181e-162,"6","Anxa1"
"F10",1.10017682701771e-165,1.23094046394047,0.464,0.003,1.85511816571
726e-161,"6","F10"
"Nxpe4",3.17337248970594e-157,0.861160580042921,0.442,0.003,5.35094069
214216e-153,"6","Nxpe4"
"Trem1",1.0444896310495e-153,1.5090710824057,0.536,0.015,1.76121841587
566e-149,"6","Trem1"
"Sirpb1b",7.81769191657024e-152,0.923349478540541,0.435,0.004,1.318219
21097207e-147,"6","Sirpb1b"
"Stap11",3.46847880926735e-151,1.94640689104361,0.754,0.054,5.84854896
81866e-147,"6","Stap1"
"Krt80",3.38693886481025e-149,1.03941988707216,0.457,0.007,5.711056313
84305e-145,"6","Krt80"
"Mgst1",5.63756909404481e-146,1.83517478923004,0.623,0.031,9.506069006
37836e-142,"6","Mgst1"
"Ccdc88c",2.65363826864942e-143,0.978713701392639,0.413,0.004,4.474564
84859665e-139,"6","Ccdc88c"
"Cxcr41",4.38684355886734e-143,1.77723361965709,0.746,0.059,7.39709560
896211e-139,"6","Cxcr4"
"Agpat4",1.88336073835679e-138,1.5772806338291,0.674,0.047,3.175722877
01722e-134,"6","Agpat4"
"Iqgap11",2.39741037702316e-136,2.8825297407532,1,0.186,4.042513377736
45e-132,"6","Iqgap1"
"Rasgrp2",1.00429738443244e-134,1.33631677780596,0.536,0.022,1.6934462
4962998e-130,"6","Rasgrp2"
"Atp8b4",6.53724107700915e-134,0.817564583888752,0.384,0.003,1.1023095
9040528e-129,"6","Atp8b4"
"Tppp3",3.68222069148295e-130,1.27627345275317,0.457,0.013,6.208960529
97856e-126,"6","Tppp3"
"Fgr",5.86926219692858e-130,1.73724577837308,0.783,0.083,9.89674991646
097e-126,"6","Fgr"
"Ifitm21",1.2825564823845e-125,3.01288239968217,0.949,0.173,2.16264674
059674e-121,"6","Ifitm2"
"Dock5",1.30548898990511e-124,0.66485748643932,0.333,0.001,2.201315534
77799e-120,"6","Dock5"
"Cd44",1.7226713965234e-124,2.64300393235098,0.949,0.18,2.904768508817
76e-120,"6","Cd44"
"Pilrb2",4.70460503023817e-124,1.02870255388657,0.471,0.017,7.93290500
19876e-120,"6","Pilrb2"
"Ly6i",7.63615343154269e-123,1.22382459701737,0.355,0.003,1.2876081916
2673e-118,"6","Ly6i"
"Ldlrad3",5.57173242158115e-120,0.806657840557471,0.377,0.006,9.395055

20927013e-116,"6","Ldlrad3"
"Heg1",1.31627255884827e-119,0.746056011006197,0.341,0.003,2.219498788
72995e-115,"6","Heg1"
"Nfe2",1.9308128477778e-117,0.67681076398679,0.355,0.005,3.25573662392
292e-113,"6","Nfe2"
"Rab11fip1",2.49117993070719e-115,0.850053886899047,0.442,0.016,4.2006
2759915847e-111,"6","Rab11fip1"
"Gm36161",2.71407774025305e-114,0.734462912639861,0.37,0.007,4.5764778
8561469e-110,"6","Gm36161"
"Clec4a11",5.00275719108782e-114,1.80246876171262,0.775,0.093,8.435649
17561228e-110,"6","Clec4a1"
"Tgm2",3.27190917505854e-112,1.64237320860685,0.572,0.042,5.5170932509
8372e-108,"6","Tgm2"
"Clec12a1",1.66867260809553e-109,1.20525652537038,0.587,0.044,2.813715
75177069e-105,"6","Clec12a"
"Nedd9",2.67637645291998e-109,1.26594738248112,0.63,0.058,4.5129059749
1367e-105,"6","Nedd9"
"4833407H14Rik",4.43547413537014e-108,0.562170056362613,0.304,0.002,7.
47909648706112e-104,"6","4833407H14Rik"
"S1pr5",1.15948716407596e-107,0.671817103030592,0.297,0.002,1.95512725
606488e-103,"6","S1pr5"
"Gm5150",1.22826053309939e-107,0.686767888991763,0.29,0.001,2.07109291
09122e-103,"6","Gm5150"
"Ly6c2",1.72227618566407e-107,1.40719102844108,0.29,0.001,2.9041021042
6676e-103,"6","Ly6c2"
"Smpdl3b",1.11880288382253e-106,1.31499764550026,0.543,0.039,1.8865254
2270156e-102,"6","Smpdl3b"
"Smpdl3a",1.58191799678537e-106,2.62216166278879,0.877,0.177,2.6674301
2617949e-102,"6","Smpdl3a"
"Lgals31",1.25575484619203e-105,2.18874714653422,0.978,0.186,2.1174538
2164899e-101,"6","Lgals3"
"Hopx",1.57566249929347e-104,0.971298481395968,0.399,0.014,2.656882106
30865e-100,"6","Hopx"
"Rara",5.14823984145227e-104,0.961116389094063,0.435,0.02,8.6809620206
5683e-100,"6","Rara"
"Runx2",6.42044510448942e-104,0.698882278141364,0.319,0.005,1.08261545
351901e-99,"6","Runx2"
"G0s2",7.57589933437099e-103,1.53774561772913,0.333,0.006,1.2774481457
6164e-98,"6","G0s2"
"Cd300e",4.76418807824224e-102,0.952002214833957,0.268,0.001,8.0333739
3753207e-98,"6","Cd300e"
"Alcam",4.32827347539212e-101,0.876891009662564,0.37,0.011,7.298334734
20619e-97,"6","Alcam"
"Runx3",7.65571551835947e-101,1.04625690816761,0.406,0.016,1.290906750
70577e-96,"6","Runx3"
"Cybb1",8.27992874619417e-101,2.62052968789428,0.942,0.211,1.396161585
18326e-96,"6","Cybb"
"Pfkp",1.30941874258498e-99,1.04923030142338,0.493,0.032,2.20794188374
68e-95,"6","Pfkp"
"Msrb1",5.70820755397708e-99,2.79959870991508,0.957,0.263,9.6251795775

1616e-95,"6","Msrb1"
"Grk3",8.44561842736376e-99,1.6887485952683,0.71,0.096,1.4241001792220
8e-94,"6","Grk3"
"Thbs1",1.12343649548201e-98,3.42399394790801,0.609,0.065,1.8943386186
8177e-94,"6","Thbs1"
"Fpr2",7.4239575171461e-98,0.743369536675212,0.297,0.004,1.25182771654
118e-93,"6","Fpr2"
"Cyfip2",1.03875011412296e-97,0.97652053866952,0.384,0.014,1.751540442
43413e-93,"6","Cyfip2"
"Crip1",2.87573767669178e-97,2.94477565778679,0.775,0.129,4.8490688704
3768e-93,"6","Crip1"
"Ifitm31",3.56063204528895e-96,3.23868599676906,0.957,0.253,6.00393775
476623e-92,"6","Ifitm3"
"Dgkg",6.6155842120521e-96,0.522042066305862,0.268,0.002,1.11551980983
623e-91,"6","Dgkg"
"Cyp4f18",6.33271759851399e-93,1.87960140829859,0.819,0.151,1.06782284
146143e-88,"6","Cyp4f18"
"Gsr",1.86406916847204e-90,2.44306034302472,0.877,0.21,3.1431934318775
5e-86,"6","Gsr"
"Bhlhe40",4.08084321075901e-90,1.287959220234,0.406,0.021,6.8811178219
8184e-86,"6","Bhlhe40"
"Fxyd5",1.4434329632172e-88,2.63753610889337,0.986,0.357,2.43391666257
684e-84,"6","Fxyd5"
"Fam49a",3.54035642989402e-88,1.42777588406735,0.674,0.096,5.969749012
0873e-84,"6","Fam49a"
"Vav3",2.45492677880441e-87,0.908147643555365,0.471,0.035,4.1394975344
1999e-83,"6","Vav3"
"Thbd",1.28666087482296e-86,0.719021262851501,0.304,0.008,2.1695675671
2647e-82,"6","Thbd"
"Metrnl1",3.5575576196793e-85,1.91292926393538,0.775,0.152,5.998753658
30323e-81,"6","Metrnl"
"Samhd1",4.28182512921367e-85,2.83950140324197,0.964,0.377,7.220013532
8801e-81,"6","Samhd1"
"Anxa2",6.80435277577503e-85,2.21341439602536,0.899,0.23,1.14734996505
119e-80,"6","Anxa2"
"Cd300ld",6.42621603260087e-83,1.1602895053714,0.471,0.039,1.083588547
41716e-78,"6","Cd300ld"
"Bst1",9.81398722946946e-82,0.741736706483862,0.304,0.01,1.65483452663
314e-77,"6","Bst1"
"Cd361",9.97474208072703e-81,1.66788760625632,0.514,0.053,1.6819410096
5219e-76,"6","Cd36"
"Trps11",2.38216087953781e-79,1.5351432757544,0.71,0.13,4.016799675076
65e-75,"6","Trps1"
"Pilrb1",3.32494983123493e-79,0.535803075565294,0.275,0.007,5.60653040
542833e-75,"6","Pilrb1"
"Capn2",6.49601615637077e-79,0.772830672963269,0.471,0.042,1.095358244
28724e-74,"6","Capn2"
"B4galt5",2.39196871571083e-78,1.11103015672535,0.601,0.083,4.03333764
84316e-74,"6","B4galt5"
"Unc119",2.8695932677676e-78,1.20126782846857,0.638,0.092,4.8387081681

0973e-74,"6","Unc119"
"Pira2",2.94341927752232e-78,0.593877373278705,0.297,0.01,4.9631935857
5814e-74,"6","Pira2"
"Myo1g",7.96986253323071e-78,1.95883917587922,0.826,0.211,1.3438782203
5336e-73,"6","Myo1g"
"Flna",1.27127774052085e-77,2.07321371281141,0.891,0.281,2.14362852606
625e-73,"6","Flna"
"C3",2.69599627124621e-76,1.07236748945169,0.486,0.049,4.5459889125753
6e-72,"6","C3"
"Clec4a31",3.48949776366626e-76,1.96712509032703,0.848,0.229,5.8839911
2909404e-72,"6","Clec4a3"
"Stk17b1",5.88125480942173e-75,2.2096372128794,0.92,0.323,9.9169718596
4692e-71,"6","Stk17b"
"Hip11",1.66890838357449e-74,1.18669836467809,0.536,0.064,2.8141133163
833e-70,"6","Hip1"
"Vim",2.02152765580147e-73,2.78969350227277,0.877,0.256,3.408699933212
44e-69,"6","Vim"
"Sik1",6.76685353439617e-71,0.966045535374002,0.471,0.05,1.14102684296
988e-66,"6","Sik1"
"Cyp4f16",2.62582325454049e-70,0.740934165691306,0.37,0.026,4.42766317
180618e-66,"6","Cyp4f16"
"S100a10",9.04117696506619e-70,1.41342361597994,0.471,0.052,1.52452325
984946e-65,"6","S100a10"
"Diaph1",7.93653913312232e-69,1.60503485039722,0.812,0.23,1.3382592286
2709e-64,"6","Diaph1"
"Grk5",1.16983377921438e-68,0.585655163566305,0.268,0.01,1.97257371851
129e-64,"6","Grk5"
"Ms4a4c",1.55403872922044e-67,1.68147692533395,0.486,0.061,2.620420105
21151e-63,"6","Ms4a4c"
"Nr4a21",3.5860534443848e-67,1.59886689338693,0.572,0.085,6.0468033179
2165e-63,"6","Nr4a2"
"Glipr2",3.68525238397413e-67,0.620308923307494,0.268,0.01,6.214072569
85718e-63,"6","Glipr2"
"Pla2g71",3.97495228117e-66,1.60367313670198,0.725,0.148,6.70256453650
886e-62,"6","Pla2g7"
"Plp2",4.99798835635688e-66,0.589380103860918,0.312,0.017,8.4276079664
8898e-62,"6","Plp2"
"Eno3",1.46203581034995e-65,1.68892682237768,0.522,0.076,2.46528478341
209e-61,"6","Eno3"
"Zfyve9",2.67861026894674e-65,1.06007241707988,0.507,0.069,4.516672635
498e-61,"6","Zfyve9"
"Lsp1",7.92349463149773e-65,2.28901936193086,0.978,0.675,1.33605966476
315e-60,"6","Lsp1"
"Skint3",2.04964043251142e-63,0.538172593289533,0.254,0.01,3.456103697
30075e-59,"6","Skint3"
"Xdh",3.24835344025124e-63,0.739430107806073,0.362,0.03,5.477373570951
64e-59,"6","Xdh"
"Zbp11",4.09125582308293e-63,1.25753179574119,0.594,0.099,6.8986755688
8243e-59,"6","Zbp1"
"Traf1",8.49356760096246e-63,0.930771720510483,0.355,0.028,1.432185368

87429e-58,"6","Traf1"
"Rnase6",1.87077170087642e-62,1.43454135611484,0.717,0.171,3.154495242
01783e-58,"6","Rnase6"
"Ccnd3",3.40860778733872e-61,1.58989909334253,0.833,0.265,5.7475944510
1055e-57,"6","Ccnd3"
"Adssl1",5.76828556197174e-61,1.29904943134018,0.71,0.162,9.7264831145
9674e-57,"6","Adssl1"
"Cd300a",4.51330670553262e-60,1.8108040858358,0.935,0.447,7.6103377668
691e-56,"6","Cd300a"
"Cdk2ap2",8.87642384741384e-60,1.99398625124974,0.899,0.381,1.49674258
915092e-55,"6","Cdk2ap2"
"Nfil31",9.00060479502866e-60,1.48167144388827,0.623,0.127,1.517681980
53773e-55,"6","Nfil3"
"Rasgrp4",1.0811593124369e-59,0.590238605348023,0.283,0.016,1.82305083
26311e-55,"6","Rasgrp4"
"Coro1a1",1.33931260715792e-59,1.54562134606634,1,0.928,2.258348918189
69e-55,"6","Coro1a"
"Hes1",2.2539231250904e-59,0.975347299726879,0.333,0.026,3.80056517352
744e-55,"6","Hes1"
"Plec",4.13329140748504e-59,1.09190613175326,0.457,0.059,6.96955597130
128e-55,"6","Plec"
"Sept9",5.65890500866102e-59,1.21919555596081,0.688,0.16,9.54204562560
421e-55,"6","Sept9"
"Trim25",1.36215411110692e-57,1.39098118148463,0.688,0.174,2.296864262
1485e-53,"6","Trim25"
"Arrdc3",1.02928244410469e-56,0.799044559323491,0.391,0.042,1.73557605
724933e-52,"6","Arrdc3"
"Cdc42ep2",1.13361837295803e-56,0.849560435770378,0.428,0.053,1.911507
30048183e-52,"6","Cdc42ep2"
"Ezr",2.12440349791121e-56,1.33508934631409,0.623,0.134,3.582169178177
88e-52,"6","Ezr"
"Mcub",3.47270408367299e-56,0.902292990878726,0.471,0.067,5.8556736258
894e-52,"6","Mcub"
"Slfn1",3.47693398260662e-56,0.967223264135792,0.268,0.016,5.862806081
47127e-52,"6","Slfn1"
"Srgn2",2.32644480712949e-55,1.65377358713417,1,0.844,3.92285123378174
e-51,"6","Srgn"
"Sh3bgrl2",5.19159123469848e-55,1.15292505756043,0.746,0.207,8.7540611
3994858e-51,"6","Sh3bgrl"
"Arhgef10l",1.0648329747974e-54,1.03595972995576,0.486,0.076,1.7955213
6210338e-50,"6","Arhgef10l"
"Cnn2",1.82286837060779e-54,1.25568005597255,0.746,0.214,3.07372064651
885e-50,"6","Cnn2"
"H3f3a1",5.17671241762827e-54,1.48473624055833,0.993,0.965,8.728972478
60479e-50,"6","H3f3a"
"Dusp5",6.52605937830689e-54,1.40031559135139,0.717,0.207,1.1004241323
7011e-49,"6","Dusp5"
"Cd300lb",2.30014138282019e-53,0.628967626746837,0.275,0.018,3.8784983
997114e-49,"6","Cd300lb"
"Ccnd2",7.13823373274236e-53,1.29782140064007,0.348,0.035,1.2036489720

1502e-48,"6","Ccnd2"
"H2-
D11",3.48685374229031e-52,1.47990462011126,1,0.943,5.87953278024991e-4
8,"6","H2-D1"
"4930523C07Rik",6.30925890359612e-52,0.898047196460643,0.333,0.032,1.0
6386723632438e-47,"6","4930523C07Rik"
"Cebpb1",9.69053439308931e-52,1.9149657256636,0.978,0.862,1.6340179093
6272e-47,"6","Cebpb"
"6430548M08Rik",2.60274053761388e-51,0.790968945236083,0.464,0.072,4.3
8874109452452e-47,"6","6430548M08Rik"
"Calm1",3.16349848709377e-51,1.37691020645029,1,0.911,5.33429114893751
e-47,"6","Calm1"
"Fn1",5.68872287470317e-51,2.02241865269527,0.428,0.06,9.5923245113244
8e-47,"6","Fn1"
"Cd471",8.34994218329277e-51,1.54871608543017,0.957,0.565,1.4079672509
4683e-46,"6","Cd47"
"Ms4a6c1",2.09208526405059e-50,1.62988469452425,0.877,0.333,3.52767417
224211e-46,"6","Ms4a6c"
"Clec4d",3.4581131791377e-50,0.874014615805295,0.312,0.029,5.831070442
662e-46,"6","Clec4d"
"Alox5ap",6.21234288836651e-50,1.73673557729084,0.964,0.704,1.04752525
783636e-45,"6","Alox5ap"
"Cd244",1.70572109695181e-49,0.785052355511027,0.428,0.062,2.876186913
68015e-45,"6","Cd244"
"Plaur",6.02439502115821e-49,1.66852412341268,0.913,0.468,1.0158334884
677e-44,"6","Plaur"
"AB124611",6.5918848020137e-48,1.2057933062034,0.717,0.218,1.111523615
31555e-43,"6","AB124611"
"St3gal4",4.53771628530953e-47,0.896975174961659,0.514,0.102,7.6514972
0028893e-43,"6","St3gal4"
"Ptpn22",4.73963333372131e-47,0.470175249986662,0.254,0.018,7.99196972
732087e-43,"6","Ptpn22"
"Lilr4b1",7.71481302437873e-46,1.12160255480175,0.543,0.11,1.300871772
17074e-41,"6","Lilr4b"
"Irf71",2.39031820014494e-45,1.11601019732699,0.681,0.176,4.0305545490
844e-41,"6","Irf7"
"Arhgap151",6.21771744315178e-45,0.929988836940825,0.572,0.13,1.048431
51526425e-40,"6","Arhgap15"
"Rbms11",1.56988990615655e-44,1.19881909502668,0.848,0.377,2.647148359
76117e-40,"6","Rbms1"
"Tnfrsf1b",2.02905592746083e-44,1.36947725278698,0.804,0.343,3.4213941
0488445e-40,"6","Tnfrsf1b"
"Pstpip1",2.20489423874702e-44,0.76639088141648,0.428,0.07,3.717892665
37522e-40,"6","Pstpip1"
"Pirb",3.83789998486928e-44,1.15720138825028,0.688,0.215,6.47146695448
659e-40,"6","Pirb"
"Fosl21",7.56382444333145e-44,1.43668053823373,0.768,0.293,1.275412077
63455e-39,"6","Fosl2"
"Gpd2",7.64727916411365e-44,0.53458821208199,0.362,0.048,1.28948421265
284e-39,"6","Gpd2"

"Mdm1",1.03427191353443e-43,0.85435350981542,0.384,0.057,1.74398930060175e-39,"6","Mdm1"
"Prdx6",1.5895719569946e-43,1.17763131235889,0.804,0.315,2.6803362338843e-39,"6","Prdx6"
"Ceacam1",6.04510468004029e-43,1.29317324156753,0.558,0.141,1.01932555114839e-38,"6","Ceacam1"
"Gpr35",1.32945751529417e-42,1.13733815789775,0.623,0.175,2.24173126228902e-38,"6","Gpr35"
"Mndal1",2.39112210807019e-42,0.846771094514519,0.493,0.093,4.03191009862796e-38,"6","Mndal"
"Dgat2",2.54734797141387e-42,0.511882592040474,0.268,0.025,4.29533814939808e-38,"6","Dgat2"
"Gsn",4.34883253694003e-42,0.95815879214128,0.601,0.15,7.33300142378828e-38,"6","Gsn"
"Pqlc3",4.46418600326673e-42,0.368365650083257,0.29,0.029,7.52751043870837e-38,"6","Pqlc3"
"Cd80",4.70373184916394e-42,0.783175440166162,0.435,0.078,7.93143264406023e-38,"6","Cd80"
"Pim11",1.03854247286867e-41,1.37449815579475,0.971,0.596,1.75119031775116e-37,"6","Pim1"
"Sorl1",1.13832562772188e-41,1.18803048505884,0.543,0.132,1.91944467346463e-37,"6","Sorl1"
"Taldo1",1.24574587923841e-41,1.20452984900068,0.993,0.779,2.10057670157181e-37,"6","Taldo1"
"Msn1",2.49469094965086e-41,1.0939222588261,0.986,0.837,4.20654787930128e-37,"6","Msn"
"Sema4a",2.50300789992715e-41,0.725171712117405,0.42,0.074,4.22057192085717e-37,"6","Sema4a"
"Gfpt1",4.41598353896072e-41,1.36890076982588,0.645,0.205,7.44623144339556e-37,"6","Gfpt1"
"Btg12",4.50421363102449e-41,1.35847722646982,1,0.832,7.59500502463349e-37,"6","Btg1"
"Nadk",1.11961675068231e-40,1.34271393825519,0.819,0.378,1.8878977650005e-36,"6","Nadk"
"Ppp2r5a",4.16657762798342e-40,1.25944632007369,0.775,0.341,7.02568319630564e-36,"6","Ppp2r5a"
"Ptprc",1.16410842548259e-39,1.37220772661013,0.971,0.765,1.96291962704874e-35,"6","Ptprc"
"Stk24",1.41150169003884e-39,1.2628550550665,0.761,0.31,2.38007414974348e-35,"6","Stk24"
"Cd300lf",1.59743717550976e-39,0.90951707405672,0.399,0.067,2.69359856534455e-35,"6","Cd300lf"
"Lrrfip1",3.0851620775173e-39,1.26079409627172,0.906,0.522,5.20220029510967e-35,"6","Lrrfip1"
"Cdc42ep3",2.47060617945313e-38,0.875265782703174,0.399,0.07,4.16593613979387e-34,"6","Cdc42ep3"
"Lrrc8c",2.48861080257702e-38,0.565548596350624,0.319,0.044,4.19629553530538e-34,"6","Lrrc8c"
"Emp31",2.86966293205263e-38,1.43245678212278,0.928,0.548,4.83882563602714e-34,"6","Emp3"

"Tmsb10",3.06083303347687e-38,2.54774740209652,0.761,0.357,5.161176661
04869e-34,"6","Tmsb10"
"Fam129a",3.37249350473495e-38,1.01741899170394,0.616,0.183,5.68669854
768408e-34,"6","Fam129a"
"Gpcpd1",3.87007305515413e-38,1.43734828195629,0.739,0.319,6.525717185
6009e-34,"6","Gpcpd1"
"Anxa51",5.21990560113957e-38,1.07666468312876,0.877,0.413,8.801804824
64154e-34,"6","Anxa5"
"Zyx",5.3044347097381e-38,1.21082572365927,0.848,0.446,8.9443378075603
9e-34,"6","Zyx"
"Svil",5.60511275055719e-38,1.00970277105535,0.536,0.137,9.45134111998
953e-34,"6","Svil"
"Stk38",1.1472536786651e-37,1.14390752920865,0.717,0.289,1.93449915296
509e-33,"6","Stk38"
"Ccl6",1.34407554735234e-37,1.8077526404067,0.855,0.513,2.266380187945
52e-33,"6","Ccl6"
"Stk10",1.7259597921659e-37,1.45643269198193,0.768,0.358,2.91031340155
014e-33,"6","Stk10"
"Fam107b",1.93366295232018e-37,1.02098388664937,0.638,0.198,3.26054247
020228e-33,"6","Fam107b"
"Arpc22",4.08631871129365e-37,0.792243314888797,1,0.957,6.890350610983
34e-33,"6","Arpc2"
"Add3",4.48028816169065e-37,0.975942534068932,0.696,0.241,7.5546618982
4277e-33,"6","Add3"
"Prr13",4.51918705464665e-37,1.30165305479639,0.877,0.505,7.6202532115
4518e-33,"6","Prr13"
"Fem1c",5.75035281527703e-37,0.989237912834758,0.601,0.178,9.696244917
12012e-33,"6","Fem1c"
"Gm2a",8.21329697294e-37,1.18326346188477,0.942,0.707,1.38492613557714
e-32,"6","Gm2a"
"Klf13",9.66398108321629e-37,1.19449390973386,0.891,0.575,1.6295404902
5193e-32,"6","Klf13"
"Utrn",1.15406405073718e-36,0.526368588937794,0.362,0.059,1.9459828023
5303e-32,"6","Utrn"
"Rap1gap2",1.80207480464198e-36,0.741314794356242,0.42,0.083,3.0386585
3558731e-32,"6","Rap1gap2"
"Stx11",2.13708795256874e-36,0.518148122130332,0.261,0.029,3.603557705
62141e-32,"6","Stx11"
"Ncf2",2.54064611793049e-36,1.15451812117806,0.942,0.726,4.28403748405
439e-32,"6","Ncf2"
"Sp1001",2.93957424045982e-36,0.956529640386599,0.652,0.211,4.95671008
426335e-32,"6","Sp100"
"Megf9",3.57346168722508e-36,0.426166538225375,0.268,0.031,6.025571096
99892e-32,"6","Megf9"
"Prdx5",4.03936160795364e-36,1.35151177471791,0.978,0.823,6.8111715433
3144e-32,"6","Prdx5"
"Rras",4.48624850279584e-36,1.12493322980288,0.688,0.256,7.56471222541
435e-32,"6","Rras"
"Myl12b",1.27404799195808e-35,1.09890987130579,0.978,0.73,2.1482997240
3972e-31,"6","Myl12b"

"Mbp",1.29879541710321e-35,0.952293363838727,0.529,0.143,2.19002883231943e-31,"6","Mbp"
"Tgfbi1",1.4510947794108e-35,1.63215618940715,0.688,0.25,2.4468360170425e-31,"6","Tgfbi"
"Cdkn2d1",2.43510153137027e-35,0.762495225520873,0.616,0.182,4.10606820219655e-31,"6","Cdkn2d"
"Slc16a31",3.03027835876753e-35,1.36094770263344,0.819,0.38,5.10965536855382e-31,"6","Slc16a3"
"Themis2",3.0446894741753e-35,1.05513645177366,0.565,0.171,5.1339553913544e-31,"6","Themis2"
"Ncf4",3.25506422542128e-35,1.15738280719452,0.862,0.483,5.48868929690536e-31,"6","Ncf4"
"Cyth1",5.49864627791643e-35,0.813993233366802,0.543,0.145,9.27181735382269e-31,"6","Cyth1"
"Cyba",7.2548654181788e-35,0.819019609882471,1,0.953,1.22331540681331e-30,"6","Cyba"
"Pygl",8.65271994000643e-35,1.03542998376746,0.659,0.22,1.45902163628388e-30,"6","Pygl"
"Actr31",1.30061085254841e-34,1.03353626217475,0.978,0.883,2.19309001956713e-30,"6","Actr3"
"Rbpms",1.72005663351614e-34,1.05052129930999,0.688,0.245,2.90035949543492e-30,"6","Rbpms"
"Pot1b",4.97645807369783e-34,0.606870923992656,0.297,0.043,8.39130360386928e-30,"6","Pot1b"
"Cers6",5.36214823791441e-34,0.710155249294275,0.471,0.115,9.04165435877127e-30,"6","Cers6"
"Gpx11",5.5856113360462e-34,1.00758065288713,0.986,0.904,9.4184578348411e-30,"6","Gpx1"
"Itgb2",7.29141545891716e-34,1.1542210743547,0.942,0.744,1.22947847468261e-29,"6","Itgb2"
"Frat2",7.9725570642654e-34,0.392496608305257,0.261,0.031,1.34433257217643e-29,"6","Frat2"
"Tpd52",1.56038773652714e-33,1.26280094248187,0.935,0.761,2.63112580133206e-29,"6","Tpd52"
"Ikbkb",5.09714989232805e-33,1.10084945686367,0.783,0.387,8.59481414844356e-29,"6","Ikbkb"
"Cblb",1.82470856820359e-32,0.644740422604709,0.341,0.059,3.0768235877049e-28,"6","Cblb"
"Rac21",3.4312703841842e-32,1.11951013327905,0.964,0.746,5.7858081218114e-28,"6","Rac2"
"Pid11",3.9089382721245e-32,1.13110871422676,0.681,0.271,6.59125171445633e-28,"6","Pid1"
"Fam69a",5.77315860040666e-32,0.724317915126274,0.493,0.127,9.73470003200571e-28,"6","Fam69a"
"Nhsl2",7.6615722569063e-32,0.509115070088937,0.297,0.046,1.29189431395954e-27,"6","Nhsl2"
"Fcgr4",1.03925719653286e-31,1.47799599213088,0.667,0.273,1.75239548479371e-27,"6","Fcgr4"
"Cdkn1b",1.98639738386926e-31,1.09854618671267,0.674,0.272,3.34946326868035e-27,"6","Cdkn1b"

"Atp1a1",2.84392075038722e-31,1.21528930947482,0.906,0.645,4.795419169
30293e-27,"6","Atp1a1"
"Actg11",2.36443603144139e-30,0.898389606175004,0.993,0.991,3.98691203
621647e-26,"6","Actg1"
"Xylt1",3.09957983706195e-30,0.755233836457037,0.377,0.08,5.2265115212
5387e-26,"6","Xylt1"
"Rnf149",3.26258815635862e-30,1.12638786234592,0.862,0.561,5.501376149
25191e-26,"6","Rnf149"
"Tkt1",4.16870371346678e-30,1.00008967472182,0.935,0.744,7.02926820164
768e-26,"6","Tkt"
"Arpc1b2",4.5078930728857e-30,0.702557694121417,0.993,0.974,7.60120929
949986e-26,"6","Arpc1b"
"Slk",5.24841089481192e-30,1.01153091545006,0.71,0.31,8.84987045083187
e-26,"6","Slk"
"Abca7",7.48628419112074e-30,0.663112867929776,0.428,0.103,1.262337240
30678e-25,"6","Abca7"
"St3gal1",1.00358422300039e-29,0.45886250545663,0.283,0.044,1.69224371
682325e-25,"6","St3gal1"
"Rasa3",1.30188978299935e-29,0.919103044840093,0.667,0.274,2.195246552
0935e-25,"6","Rasa3"
"Rap1b",2.04492178319287e-29,0.915465908201504,0.993,0.893,3.448147110
81982e-25,"6","Rap1b"
"Tln1",2.5948022617514e-29,1.0999019314352,0.957,0.746,4.3753555737652
e-25,"6","Tln1"
"Wfdc17",3.50031105660103e-29,1.20239050963684,0.384,0.087,5.902224503
64065e-25,"6","Wfdc17"
"Bri3bp",3.62518564760773e-29,0.731562799685247,0.529,0.16,6.112788038
99616e-25,"6","Bri3bp"
"St8sia41",6.2349379867632e-29,0.559727725220328,0.406,0.092,1.0513352
4332801e-24,"6","St8sia4"
"H2-
T231",9.22678790932376e-29,1.07849666970263,0.891,0.534,1.555820977270
17e-24,"6","H2-T23"
"Nod1",1.45607320817724e-28,0.495993184506627,0.326,0.062,2.4552306436
2845e-24,"6","Nod1"
"Prkcd",1.89856979791014e-28,1.09267284493138,0.891,0.692,3.2013683932
3607e-24,"6","Prkcd"
"Fam241a",2.00907978130945e-28,0.548505223702394,0.29,0.049,3.38771032
724399e-24,"6","Fam241a"
"Dusp16",2.40877655678848e-28,1.3906271295103,0.696,0.325,4.0616790300
5673e-24,"6","Dusp16"
"Nr4a12",2.7526511449243e-28,1.10429813164562,0.935,0.572,4.6415203605
7135e-24,"6","Nr4a1"
"Il17ra",3.33951077900709e-28,1.05046038905617,0.739,0.373,5.631083075
56175e-24,"6","Il17ra"
"Degs1",4.75164057827071e-28,0.92242570100328,0.746,0.365,8.0122163430
8008e-24,"6","Degs1"
"Ifi27l2a1",6.86854843405213e-28,1.31499629946726,0.71,0.282,1.1581746
3694987e-23,"6","Ifi27l2a"
"Fgd41",7.21355579295059e-28,0.835451571933056,0.58,0.202,1.2163497778

0733e-23,"6","Fgd4"
"Ttc7",1.21362723663939e-27,0.856968202732326,0.551,0.192,2.0464182464
2134e-23,"6","Ttc7"
"Myh9",1.28988408608616e-27,0.974311128730345,0.92,0.658,2.17500254595
849e-23,"6","Myh9"
"Nab1",2.37694853104094e-27,0.973299075712471,0.703,0.326,4.0080106130
4123e-23,"6","Nab1"
"Capzb2",2.92705057523624e-27,0.775911339972056,0.978,0.877,4.93559267
996334e-23,"6","Capzb"
"Stat11",3.80162295151092e-27,1.34193793860241,0.623,0.252,6.410296620
83772e-23,"6","Stat1"
"Ass1",4.87238517618145e-27,0.485775938428363,0.261,0.04,8.21581588407
715e-23,"6","Ass1"
"Klf42",5.49066283826204e-27,1.09373965040719,0.775,0.383,9.2583556778
7744e-23,"6","Klf4"
"Malt1",6.3465694207628e-27,1.0210262363116,0.486,0.148,1.070158535729
02e-22,"6","Malt1"
"Arpc5",6.53871024121978e-27,0.793705033277991,0.971,0.811,1.102557320
87448e-22,"6","Arpc5"
"Myadm",6.91522903183825e-27,0.826128189090898,0.587,0.208,1.166045919
34857e-22,"6","Myadm"
"Gk",1.09239023551665e-26,0.7664882851761,0.341,0.073,1.84198841512817
e-22,"6","Gk"
"Gngt2",1.55253715272382e-26,1.56228759979339,0.826,0.534,2.6178881469
2291e-22,"6","Gngt2"
"Gm63771",1.83133594395516e-26,0.727355369640735,0.529,0.153,3.0879986
686972e-22,"6","Gm6377"
"Lgals12",2.10903595518637e-26,1.0130707044715,0.529,0.168,3.556256427
63526e-22,"6","Lgals1"
"Dedd2",2.75608788260228e-26,0.625312159790016,0.268,0.046,4.647315387
64397e-22,"6","Dedd2"
"Grk6",3.33989144665274e-26,0.721223990103587,0.522,0.166,5.6317249573
4586e-22,"6","Grk6"
"H2-
K11",4.69725675601124e-26,1.01392274634822,0.986,0.906,7.9205143419861
5e-22,"6","H2-K1"
"Foxn2",4.82301163832938e-26,0.574868678861126,0.449,0.128,8.132562224
55099e-22,"6","Foxn2"
"D16Ertd472e",6.04331069278023e-26,0.512327866913775,0.333,0.072,1.019
0230490166e-21,"6","D16Ertd472e"
"Limd1",6.6673822001988e-26,0.697448232821882,0.442,0.126,1.1242539865
9752e-21,"6","Limd1"
"Ifngr1",1.75665450865597e-25,0.971891190447122,0.964,0.844,2.96207083
24957e-21,"6","Ifngr1"
"Klf3",1.91256320967177e-25,1.03667728485122,0.826,0.501,3.22496408414
853e-21,"6","Klf3"
"Ptpre",2.26068425374402e-25,1.15307551797118,0.848,0.535,3.8119657886
6317e-21,"6","Ptpre"
"Noct",3.44089408248708e-25,0.66247282140475,0.399,0.103,5.80203560188
972e-21,"6","Noct"

"Sem1",3.61330084741211e-25,1.07942845685583,0.884,0.6,6.0927478889063
e-21,"6","Sem1"
"Jarid2",3.6197479934572e-25,0.776466475487867,0.551,0.196,6.103619066
56754e-21,"6","Jarid2"
"Tmem164",5.13457475643876e-25,0.581173811886845,0.442,0.125,8.6579199
5430704e-21,"6","Tmem164"
"Fam26f",6.55132493977734e-25,0.733971473711976,0.319,0.069,1.10468441
134525e-20,"6","Fam26f"
"Snx20",7.23235803363514e-25,0.986968677605858,0.819,0.5,1.21952021163
156e-20,"6","Snx20"
"Atp2b11",1.02228342526045e-24,1.06862543730031,0.92,0.709,1.723774311
67416e-20,"6","Atp2b1"
"Dok2",1.54285019225247e-24,0.704427151652814,0.275,0.051,2.6015539941
7612e-20,"6","Dok2"
"Stom1",1.73101534025494e-24,0.484547937596092,0.304,0.063,2.918838066
73788e-20,"6","Stom"
"Cfp1",1.78368661295919e-24,0.538698064884165,0.399,0.097,3.0076523667
7178e-20,"6","Cfp"
"Gstm1",1.83961802848525e-24,0.409280802491586,0.341,0.076,3.101963919
63183e-20,"6","Gstm1"
"1110008P14Rik",2.13910997411394e-24,0.380813648261076,0.261,0.046,3.6
0696723835092e-20,"6","1110008P14Rik"
"Herc4",2.96588185819594e-24,0.773112988035318,0.536,0.194,5.001069989
28999e-20,"6","Herc4"
"Atp11b",3.07843792618816e-24,0.654007285324789,0.457,0.137,5.19086203
113847e-20,"6","Atp11b"
"Syk",3.12133458138005e-24,0.976905664956532,0.732,0.365,5.26319437112
305e-20,"6","Syk"
"Atp1a3",3.32518030159761e-24,0.493276680842797,0.283,0.055,5.60691902
455389e-20,"6","Atp1a3"
"Shisa5",3.35792816544729e-24,0.76350967609071,0.891,0.638,5.662138472
57722e-20,"6","Shisa5"
"Glud1",3.64028528179437e-24,0.936384957449267,0.826,0.54,6.1382490421
6166e-20,"6","Glud1"
"Fam32a1",4.60639750230396e-24,0.884958756230934,0.688,0.324,7.7673074
6838494e-20,"6","Fam32a"
"Ptpn12",4.61524176015229e-24,0.669909841627173,0.399,0.108,7.78222065
59688e-20,"6","Ptpn12"
"Dna21",6.36894852866054e-24,0.458818031944315,0.428,0.118,1.073932100
90274e-19,"6","Dna2"
"Lst1",7.27899647856961e-24,1.56280811595227,0.797,0.508,1.22738438621
641e-19,"6","Lst1"
"Id3",8.05169429625887e-24,0.685504846361761,0.29,0.057,1.357676692235
17e-19,"6","Id3"
"Ostf1",8.58661304792064e-24,0.798687800734818,0.949,0.823,1.447874692
14038e-19,"6","Ostf1"
"Arhgdib2",1.05027121423949e-23,0.777346408465435,0.971,0.86,1.7709673
2145063e-19,"6","Arhgdib"
"Rap1a1",1.07774008905359e-23,0.869686703149379,0.964,0.802,1.81728533
816216e-19,"6","Rap1a"

"Psmb81",1.46074951653327e-23,0.885268257177842,0.935,0.758,2.46311583
47784e-19,"6","Psmb8"
"Apobec11",1.56429103320868e-23,0.76003172198269,0.587,0.241,2.6377075
4019647e-19,"6","Apobec1"
"Pilra1",1.71569264749795e-23,0.949719404477402,0.609,0.267,2.89300094
221104e-19,"6","Pilra"
"Zc3hav1",1.79623536157163e-23,0.870269826520621,0.717,0.358,3.0288120
6668209e-19,"6","Zc3hav1"
"Arpc31",2.42412684646966e-23,0.618607349428427,0.986,0.904,4.08756268
851714e-19,"6","Arpc3"
"Ifi2111",3.26014482763402e-23,0.64147750112274,0.348,0.083,5.49725620
835648e-19,"6","Ifi211"
"Isg20",3.39434136411843e-23,0.602093921432916,0.268,0.052,5.723538408
17649e-19,"6","Isg20"
"Zfp36l21",4.21905085385917e-23,1.08433721239254,0.891,0.683,7.1141635
4977734e-19,"6","Zfp36l2"
"Gyg1",4.66996397590516e-23,0.626645700439455,0.536,0.195,7.8744932561
7129e-19,"6","Gyg"
"Vcl",6.71927967217271e-23,0.364999429666252,0.268,0.051,1.13300493832
176e-18,"6","Vcl"
"Sh2d1b1",8.52469683113012e-23,0.490930455725933,0.304,0.066,1.4374343
7966516e-18,"6","Sh2d1b1"
"Rgs22",8.73346085108226e-23,0.978529891514883,0.797,0.509,1.472636168
70949e-18,"6","Rgs2"
"Slc25a20",9.23286562031737e-23,0.526479175808819,0.399,0.111,1.556845
80089791e-18,"6","Slc25a20"
"Elmo2",9.48329318812431e-23,0.664614301249816,0.449,0.147,1.599072897
38152e-18,"6","Elmo2"
"Plekhm3",1.12690580595519e-22,0.6287333704107,0.5,0.17,1.900188570001
64e-18,"6","Plekhm3"
"Raf1",1.2869484147595e-22,0.718650006250428,0.638,0.276,2.17005241696
747e-18,"6","Raf1"
"Lnpep",1.31678117619809e-22,0.789513418906551,0.717,0.366,2.220356419
30521e-18,"6","Lnpep"
"Arhgef31",1.65748760875578e-22,0.562774032413391,0.326,0.077,2.794855
605884e-18,"6","Arhgef3"
"Rassf5",2.36648075700201e-22,0.894131714648603,0.703,0.342,3.99035985
245679e-18,"6","Rassf5"
"Adcy7",2.41040325240428e-22,0.869041874247972,0.739,0.394,4.064421964
2041e-18,"6","Adcy7"
"Aprt",2.43406366494431e-22,0.910242396237772,0.833,0.511,4.1043181518
291e-18,"6","Aprt"
"Nin",3.1997918875308e-22,0.790218538849863,0.522,0.197,5.395489080754
43e-18,"6","Nin"
"Plin21",3.33869244596928e-22,0.922192867987998,0.797,0.454,5.62970320
23934e-18,"6","Plin2"
"Cd274",3.34966773285881e-22,0.468590286759781,0.29,0.062,5.6482097311
4652e-18,"6","Cd274"
"Dok3",3.52471488571188e-22,0.813262214861459,0.594,0.248,5.9433742402
8737e-18,"6","Dok3"

"Pdha1",4.77903441710518e-22,0.834655773662543,0.58,0.233,8.0584078341
2275e-18,"6","Pdha1"
"Tpm41",5.16492562190118e-22,0.873615564781635,0.855,0.56,8.7090975836
4977e-18,"6","Tpm4"
"Epsti11",5.20491237615329e-22,0.808197980285809,0.71,0.349,8.77652324
866967e-18,"6","Epsti1"
"Ifi2071",8.23741716144294e-22,0.417634413148066,0.63,0.229,1.38899328
176251e-17,"6","Ifi207"
"Fmnl1",8.6533170697011e-22,0.785677916775756,0.775,0.465,1.4591223242
93e-17,"6","Fmnl1"
"Aldh3b1",1.05295710842282e-21,0.708951267832139,0.522,0.195,1.7754962
7622256e-17,"6","Aldh3b1"
"Pitpna",1.19256862744167e-21,0.863445238577662,0.877,0.709,2.01090921
959214e-17,"6","Pitpna"
"Rhoa2",1.34877949788184e-21,0.621065874013202,0.993,0.931,2.274311989
32835e-17,"6","Rhoa"
"Sgk3",1.42734525172272e-21,0.828076438509448,0.623,0.289,2.4067895634
5484e-17,"6","Sgk3"
"Capg",2.70919382112539e-21,1.1281205961415,0.688,0.343,4.568242621181
63e-17,"6","Capg"
"C130050O18Rik",3.24935809134885e-21,0.480729158398898,0.319,0.077,5.4
7906761363243e-17,"6","C130050O18Rik"
"Cast",3.34853913017351e-21,0.797249304647963,0.558,0.232,5.6463066812
9857e-17,"6","Cast"
"Ptk2b",3.88828016748913e-21,0.851186786417276,0.638,0.319,6.556418018
42016e-17,"6","Ptk2b"
"Ifi2031",5.04042372719568e-21,0.469723777365244,0.333,0.081,8.4991624
8879735e-17,"6","Ifi203"
"Mapkapk3",5.32433125520989e-21,0.592382401543039,0.406,0.123,8.977887
36253492e-17,"6","Mapkapk3"
"Cbfa2t3",7.4546541648751e-21,0.702938501536209,0.493,0.174,1.25700378
528124e-16,"6","Cbfa2t3"
"Eif4a2",7.54264381847883e-21,0.693514455070496,0.826,0.456,1.27184060
06719e-16,"6","Eif4a2"
"Mkl1",1.12100969291723e-20,0.434355220073296,0.326,0.081,1.8902465441
9703e-16,"6","Mkl1"
"Sh2d3c",1.44647439395864e-20,0.466330897997078,0.297,0.069,2.43904512
309306e-16,"6","Sh2d3c"
"Camkk2",1.45157132106702e-20,0.796636549850347,0.616,0.271,2.44763956
158321e-16,"6","Camkk2"
"Spi1",1.50947522855018e-20,0.724930822610604,0.986,0.905,2.5452771303
8131e-16,"6","Spi1"
"Arf51",1.65416950549715e-20,0.674554632811697,0.942,0.745,2.789260620
16929e-16,"6","Arf5"
"Nfam1",2.16291498861374e-20,0.92916529159821,0.761,0.462,3.6471072538
0049e-16,"6","Nfam1"
"Bcl2",2.42275308305317e-20,0.653074124204917,0.341,0.091,4.0852462486
4425e-16,"6","Bcl2"
"Ccdc88a1",2.9011699966063e-20,0.842767903080756,0.674,0.339,4.8919528
4827754e-16,"6","Ccdc88a"

"Lyz21",3.2890429510109e-20,3.09765376790362,0.652,0.361,5.54598422399
458e-16,"6","Lyz2"
"Sept6",3.84405205080941e-20,0.412748811782058,0.312,0.077,6.481840568
07483e-16,"6","Sept6"
"Ptp4a1",5.00501427919753e-20,0.808905298578997,0.768,0.462,8.43945507
758287e-16,"6","Ptp4a1"
"Myl61",5.22468334645802e-20,0.710997101216862,0.949,0.848,8.809861058
79751e-16,"6","Myl6"
"Lcp1",6.08850901596367e-20,0.825070594318259,0.957,0.798,1.0266443902
7179e-15,"6","Lcp1"
"Csgalnact2",6.79026706370783e-20,0.597436361690488,0.333,0.091,1.1449
7483228241e-15,"6","Csgalnact2"
"Prkch",7.73652784874604e-20,0.589534187974644,0.413,0.134,1.304533325
85556e-15,"6","Prkch"
"Dnase1l1",8.8734990846858e-20,0.449467339700104,0.348,0.096,1.4962494
1565972e-15,"6","Dnase1l1"
"Cyb5r4",9.26026354544974e-20,0.56497566159572,0.529,0.21,1.5614656390
3374e-15,"6","Cyb5r4"
"Inpp1",1.05364305676164e-19,0.510156605305399,0.29,0.07,1.77665292231
148e-15,"6","Inpp1"
"Hectd1",1.25521239473713e-19,0.798029400640044,0.812,0.485,2.11653914
000575e-15,"6","Hectd1"
"Apobr",1.4772924105809e-19,0.673518475738904,0.428,0.148,2.4910104627
2151e-15,"6","Apobr"
"Bak1",1.78183184982084e-19,0.681361772423189,0.594,0.258,3.0045248651
679e-15,"6","Bak1"
"Csf2ra",1.84373401848814e-19,0.72606959074769,0.79,0.475,3.1089043019
747e-15,"6","Csf2ra"
"Il1b3",2.02375145593348e-19,1.12230679826131,0.891,0.541,3.4124497049
9503e-15,"6","Il1b"
"Pde4b1",2.4737295437935e-19,0.690790580957583,0.696,0.345,4.171202756
7446e-15,"6","Pde4b"
"Ptprj",2.5190987195045e-19,0.914408190594761,0.739,0.439,4.2477042608
2849e-15,"6","Ptprj"
"Ddhd1",2.73637925746661e-19,0.547960810829721,0.435,0.147,4.614082703
94019e-15,"6","Ddhd1"
"Dgkh",2.77873066506748e-19,0.435116351784797,0.275,0.065,4.6854956474
3678e-15,"6","Dgkh"
"Ms4a6b1",2.83579992976275e-19,0.699192694141507,0.688,0.333,4.7817258
4156594e-15,"6","Ms4a6b"
"Ybx31",3.03096345016478e-19,0.531443699548011,0.536,0.216,5.110810569
66785e-15,"6","Ybx3"
"Kdm7a",3.21440404749497e-19,0.820268494227278,0.703,0.4,5.42012810488
602e-15,"6","Kdm7a"
"Chmp2a",3.39255468775433e-19,0.710693415113203,0.848,0.572,5.72052571
449134e-15,"6","Chmp2a"
"Psma71",3.84445480973655e-19,0.738604610748857,0.87,0.649,6.482519700
17777e-15,"6","Psma7"
"Igsf6",3.99593455417792e-19,0.96016876924234,0.768,0.48,6.73794484525
481e-15,"6","Igsf6"

"Nuak21",4.36472932281752e-19,0.743329376020641,0.725,0.373,7.35980658
41349e-15,"6","Nuak2"
"Klhl2",4.79432623159558e-19,0.544698004381732,0.341,0.096,8.084192891
71647e-15,"6","Klhl2"
"Osgin2",7.32545140772933e-19,0.646747981611432,0.37,0.111,1.235217616
37132e-14,"6","Osgin2"
"D17Wsu92e",7.39379088298334e-19,0.670616237718775,0.558,0.246,1.24674
101868865e-14,"6","D17Wsu92e"
"Ly6e1",7.80950364309626e-19,0.754112846703658,0.971,0.896,1.316838504
29889e-14,"6","Ly6e"
"Rgs14",9.24024147585499e-19,0.439594237181813,0.254,0.055,1.558089517
65867e-14,"6","Rgs14"
"Aldh21",9.39616807578134e-19,0.655212492365646,0.826,0.495,1.58438186
093825e-14,"6","Aldh2"
"Rab8a",1.3921086699813e-18,0.805492451354909,0.681,0.358,2.3473736393
2248e-14,"6","Rab8a"
"Sgms1",1.54576177083727e-18,0.431872147641189,0.406,0.131,2.606463497
98581e-14,"6","Sgms1"
"Mrpl33",1.68812591742798e-18,0.838183642336213,0.572,0.271,2.84651792
196706e-14,"6","Mrpl33"
"Nabp11",1.7289667266445e-18,0.857278354108764,0.536,0.217,2.915383694
46796e-14,"6","Nabp1"
"Ifi2041",2.22850276175072e-18,0.753169354337511,0.688,0.333,3.7577013
5686406e-14,"6","Ifi204"
"Ap1s2",2.51336338873882e-18,0.845730188297055,0.688,0.372,4.238033346
09139e-14,"6","Ap1s2"
"Arhgap9",2.65752334525476e-18,0.756911364665643,0.623,0.305,4.4811158
6476857e-14,"6","Arhgap9"
"Acot9",3.26012986224658e-18,0.520772076189062,0.478,0.179,5.497230973
72019e-14,"6","Acot9"
"Ppdpf",4.91032346632252e-18,0.453549822310207,0.406,0.138,8.279787428
91303e-14,"6","Ppdpf"
"Jdp21",5.11348240880495e-18,0.600636480714261,0.435,0.152,8.622354037
72691e-14,"6","Jdp2"
"Ehbp1l1",6.75685843423515e-18,0.681908438236905,0.754,0.437,1.1393414
6918073e-13,"6","Ehbp1l1"
"Rassf3",7.38146672667403e-18,0.74266389591147,0.601,0.288,1.244662919
45177e-13,"6","Rassf3"
"Mtpn",7.53337519118459e-18,0.632328921224103,0.717,0.413,1.2702777247
3755e-13,"6","Mtpn"
"Eif4ebp1",1.37109349132862e-17,0.571426060922665,0.616,0.279,2.311937
84507832e-13,"6","Eif4ebp1"
"Eif3h2",1.58477672150278e-17,0.679980256389098,0.92,0.785,2.672250507
79799e-13,"6","Eif3h"
"Parl",1.76641413385775e-17,0.754552939467985,0.558,0.26,2.97852751251
094e-13,"6","Parl"
"Lilrb4a1",2.26469290021085e-17,0.652619078518008,0.493,0.192,3.818725
16833554e-13,"6","Lilrb4a"
"Abracl1",2.44212958516532e-17,0.638048068616094,0.819,0.535,4.1179189
0650576e-13,"6","Abracl"

"Hpcal1",3.14609852833217e-17,0.585974699684648,0.616,0.287,5.30495133
84737e-13,"6","Hpcal1"
"Arid3a",3.38782763892979e-17,0.633735396702691,0.507,0.209,5.71255496
476341e-13,"6","Arid3a"
"Arhgap30",3.40446905856164e-17,0.742583916000878,0.783,0.536,5.740615
72654664e-13,"6","Arhgap30"
"Hspa82",5.69712997003634e-17,0.547420450062285,0.993,0.975,9.60650055
547527e-13,"6","Hspa8"
"Slfn51",6.80539857676489e-17,0.583694614657102,0.435,0.152,1.14752630
80141e-12,"6","Slfn5"
"Smchd11",6.84609199282273e-17,0.859144680443465,0.725,0.468,1.1543880
3182977e-12,"6","Smchd1"
"Lats2",7.32315665026761e-17,0.492355797256124,0.384,0.132,1.234830674
36812e-12,"6","Lats2"
"Lfng",7.70644128728513e-17,0.537072658963784,0.464,0.18,1.29946012986
202e-12,"6","Lfng"
"Mdfic1",7.99847286247483e-17,0.280412673765812,0.261,0.063,1.34870249
407051e-12,"6","Mdfic"
"B4galnt1",8.23161846761265e-17,0.724690167018827,0.659,0.372,1.388015
50600884e-12,"6","B4galnt1"
"Tnfrsf1a",8.51227065973919e-17,0.7008644564703,0.862,0.624,1.43533907
864522e-12,"6","Tnfrsf1a"
"Anxa11",8.96318912163331e-17,0.519814100522303,0.333,0.103,1.51137294
968981e-12,"6","Anxa11"
"Ets21",9.1281123246884e-17,0.804576583140749,0.652,0.357,1.5391823001
8896e-12,"6","Ets2"
"Rpl82",1.28471696926592e-16,0.438437805839001,0.978,0.976,2.166289753
57619e-12,"6","Rpl8"
"Mpc2",1.67302596938981e-16,0.677377712261585,0.71,0.425,2.82105638958
51e-12,"6","Mpc2"
"N4bp2l1",2.05594503173571e-16,0.525190252131005,0.507,0.218,3.4667345
1251275e-12,"6","N4bp2l1"
"5031439G07Rik",2.24188472374765e-16,0.712927860829713,0.616,0.338,3.7
8026602118329e-12,"6","5031439G07Rik"
"Polb",2.27160599563178e-16,0.660392907435278,0.529,0.253,3.8303820298
3431e-12,"6","Polb"
"Arhgef1",2.37152111596617e-16,0.707375530887315,0.688,0.403,3.9988589
0574216e-12,"6","Arhgef1"
"Eif3k",3.08368730687874e-16,0.572868640856544,0.899,0.706,5.199713536
85893e-12,"6","Eif3k"
"Fyn",3.28740277463414e-16,0.417276481829612,0.399,0.139,5.54321855858
809e-12,"6","Fyn"
"Ptp4a2",3.71895649471902e-16,0.622352573899477,0.891,0.74,6.270904441
39522e-12,"6","Ptp4a2"
"Foxp1",4.08064552989167e-16,0.814589408432268,0.601,0.328,6.880784492
50333e-12,"6","Foxp1"
"Eif4g3",4.74638945287145e-16,0.629978426840502,0.739,0.45,8.003361895
43184e-12,"6","Eif4g3"
"Fam174a1",5.14169966287072e-16,0.643022405870053,0.732,0.413,8.669933
97153261e-12,"6","Fam174a"

"Abcg3",5.32172982537587e-16,0.314988771132678,0.275,0.072,8.97350083154879e-12,"6","Abcg3"
"Vamp4",5.55072359479499e-16,0.549687230649348,0.565,0.276,9.35963012554332e-12,"6","Vamp4"
"Ppp2r5c",6.1450463241507e-16,0.738759481255997,0.717,0.422,1.03617771117829e-11,"6","Ppp2r5c"
"Psma21",7.09496109431e-16,0.557819176635136,0.87,0.633,1.19635233972255e-11,"6","Psma2"
"Mfsd14b",8.18077619520704e-16,0.558361576841023,0.442,0.174,1.37944248203581e-11,"6","Mfsd14b"
"Man2a11",8.22425590728143e-16,0.473422379257031,0.377,0.13,1.38677403108579e-11,"6","Man2a1"
"Txn11",9.85167913235153e-16,0.873726056078409,0.732,0.452,1.66119013529712e-11,"6","Txn1"
"Ptpn6",9.97381907507389e-16,0.623902206001202,0.928,0.77,1.68178537243896e-11,"6","Ptpn6"
"Klhl5",1.04860131906701e-15,0.547700616154641,0.486,0.202,1.76815154421078e-11,"6","Klhl5"
"Ndel11",1.28738292762297e-15,0.625738846517816,0.833,0.595,2.17078509255786e-11,"6","Ndel1"
"Tagln21",1.40849060261883e-15,0.774947364022952,0.761,0.456,2.37499685413588e-11,"6","Tagln2"
"Snrpb1",1.45177272226284e-15,0.65647736992761,0.928,0.756,2.4479791642796e-11,"6","Snrpb"
"Psme2",1.66871626284329e-15,0.795456761030211,0.797,0.607,2.81378936240635e-11,"6","Psme2"
"Gbp21",2.00429775802087e-15,1.21740749865752,0.297,0.092,3.37964687957479e-11,"6","Gbp2"
"Plcg2",2.43165797620695e-15,0.662853403336195,0.746,0.468,4.10026167948016e-11,"6","Plcg2"
"Pdlim5",2.51221522537684e-15,0.544324026245559,0.435,0.171,4.23609731303042e-11,"6","Pdlim5"
"Spop",2.64716109820067e-15,0.635097101829536,0.732,0.458,4.46364304378597e-11,"6","Spop"
"Ankrd44",2.86022851049954e-15,0.811477547929456,0.783,0.553,4.82291731440433e-11,"6","Ankrd44"
"Ywhag1",2.88049759313248e-15,0.477974655583316,0.674,0.358,4.85709504153999e-11,"6","Ywhag"
"Taf10",3.17861439380045e-15,0.634360664383729,0.761,0.492,5.35977959082631e-11,"6","Taf10"
"Il13ra1",3.76052147328867e-15,0.617019768546705,0.623,0.34,6.34099130825935e-11,"6","Il13ra1"
"Gnb21",4.10854487831463e-15,0.521245393058725,0.971,0.903,6.92782837381413e-11,"6","Gnb2"
"Samd9l1",5.108830805432e-15,0.470436941580207,0.254,0.066,8.61451050411943e-11,"6","Samd9l"
"Sipa1l3",5.11393734050021e-15,0.309126486126671,0.275,0.074,8.62312114355146e-11,"6","Sipa1l3"
"Atg2a",5.35383833745775e-15,0.4548299252341,0.377,0.134,9.02764220462126e-11,"6","Atg2a"

"Tmc6",6.05667761675507e-15,0.505583211089839,0.42,0.168,1.02127697973
724e-10,"6","Tmc6"
"Mbd2",6.26046826160029e-15,0.67294259710493,0.703,0.424,1.05564015827
104e-10,"6","Mbd2"
"Rnh1",8.33291004657425e-15,0.724057647150987,0.884,0.675,1.4050952920
5335e-10,"6","Rnh1"
"Notch2",8.44384196101104e-15,0.442750289561506,0.413,0.159,1.42380063
146568e-10,"6","Notch2"
"Gsap",8.56734175127975e-15,0.539699165520886,0.551,0.268,1.4446251661
0079e-10,"6","Gsap"
"Cyth3",8.89435229958835e-15,0.744908305653675,0.37,0.133,1.4997656847
5659e-10,"6","Cyth3"
"Tmem131",9.36616998880561e-15,0.533266528816351,0.536,0.247,1.5793235
835124e-10,"6","Tmem131"
"Pgk12",1.2502816798845e-14,0.410613206249062,0.855,0.56,2.10822496862
124e-10,"6","Pgk1"
"Nrros1",1.49690783850042e-14,0.713394152186406,0.768,0.496,2.52408599
727942e-10,"6","Nrros"
"Abcg1",1.61913894220488e-14,0.594989635243621,0.522,0.23,2.7301920843
4587e-10,"6","Abcg1"
"Capn1",1.73508676756569e-14,0.362306529076705,0.297,0.091,2.925703307
46926e-10,"6","Capn1"
"Crtc31",1.9557089666686e-14,0.460857851595104,0.428,0.167,3.297716459
5966e-10,"6","Crtc3"
"Myo1f",2.1801546063005e-14,0.704947488046329,0.804,0.59,3.67617669714
39e-10,"6","Myo1f"
"Rell1",2.33097499773542e-14,0.302997727507293,0.268,0.075,3.930490041
18146e-10,"6","Rell1"
"Ccdc71l",2.89730797574983e-14,0.437895577484329,0.391,0.149,4.8854407
0870937e-10,"6","Ccdc71l"
"Uqcrfs11",3.13512196972828e-14,0.537941075622429,0.812,0.542,5.286442
66535582e-10,"6","Uqcrfs1"
"Anp32a",3.44621924950564e-14,0.779518038704668,0.783,0.579,5.81101489
851641e-10,"6","Anp32a"
"Nt5c",3.5625817134136e-14,0.527375199101624,0.565,0.288,6.00722528515
801e-10,"6","Nt5c"
"Hipk1",4.09397772855243e-14,0.550608872042711,0.594,0.313,6.903265245
88511e-10,"6","Hipk1"
"Cd52",4.87416680936329e-14,0.913371734189231,0.935,0.823,8.2188200739
4838e-10,"6","Cd52"
"Dbnl",5.1704189697285e-14,0.613241907960318,0.754,0.508,8.71836046675
619e-10,"6","Dbnl"
"Me21",6.55752952422162e-14,0.501192448620099,0.37,0.142,1.10573062837
425e-09,"6","Me2"
"Polr2l",6.72044290393304e-14,0.416823284046927,0.261,0.074,1.13320108
246119e-09,"6","Polr2l"
"Crlf21",7.06110917196388e-14,0.501922969182865,0.768,0.45,1.190644228
57655e-09,"6","Crlf2"
"Mef2d1",7.31011903673709e-14,0.668126621466854,0.623,0.366,1.23263227
197461e-09,"6","Mef2d"

"Scand1",8.40382778953585e-14,0.680122472106578,0.754,0.507,1.41705344187153e-09,"6","Scand1"
"Rin3",8.47110658462202e-14,0.623444061549626,0.435,0.182,1.42839799229896e-09,"6","Rin3"
"Klf101",8.71199020078311e-14,0.490355306092395,0.5,0.22,1.46901578765605e-09,"6","Klf10"
"Gsdmd",8.80648405358237e-14,0.701163122463913,0.71,0.445,1.48494934111506e-09,"6","Gsdmd"
"Mxd1",1.05959847616143e-13,1.03971017188357,0.268,0.081,1.7866949505034e-09,"6","Mxd1"
"Dazap2",1.24158602679696e-13,0.58075905215554,0.855,0.659,2.09356235838503e-09,"6","Dazap2"
"Tmem38b",1.25263161025299e-13,0.539468000960603,0.319,0.111,2.11218742120859e-09,"6","Tmem38b"
"Tmcc1",1.32354327482634e-13,0.721688614505165,0.572,0.294,2.23175867001218e-09,"6","Tmcc1"
"Fam49b",1.39836077926531e-13,0.520155132775177,0.957,0.882,2.35791594599717e-09,"6","Fam49b"
"Pitpnm1",1.44133737318515e-13,0.455564925933997,0.341,0.122,2.4303830786648e-09,"6","Pitpnm1"
"Ifi2091",1.47937042050647e-13,0.483108819797583,0.297,0.096,2.49451440305802e-09,"6","Ifi209"
"Hmgb21",1.54242198806156e-13,0.338421555610925,0.819,0.54,2.60083195626941e-09,"6","Hmgb2"
"Btf32",1.87162449186352e-13,0.500864804288772,0.949,0.896,3.15593321818027e-09,"6","Btf3"
"Hcls1",2.05445730735542e-13,0.570458107154049,0.855,0.686,3.46422591166271e-09,"6","Hcls1"
"Rpsa2",2.3349468003119e-13,0.472949794224913,0.978,0.987,3.93718729468593e-09,"6","Rpsa"
"Fam117b",2.34899365517806e-13,0.46944500747853,0.565,0.284,3.96087310136125e-09,"6","Fam117b"
"Birc6",2.514838129823e-13,0.605839555411794,0.703,0.438,4.24052005450754e-09,"6","Birc6"
"Cmip",2.53825207229822e-13,0.64200783824114,0.659,0.394,4.28000064430925e-09,"6","Cmip"
"Fyb1",2.72777986258854e-13,0.592172720428442,0.935,0.844,4.5995824042968e-09,"6","Fyb"
"Api5",3.13639838944312e-13,0.402968513619463,0.558,0.274,5.28859496427899e-09,"6","Api5"
"Kras",3.13701468999673e-13,0.575293356086123,0.79,0.562,5.28963417027249e-09,"6","Kras"
"Tspan13",3.26212286159714e-13,0.608395278156289,0.58,0.308,5.5005915692251e-09,"6","Tspan13"
"Pqlc1",3.50114382379597e-13,0.342400119164142,0.326,0.113,5.90362871568476e-09,"6","Pqlc1"
"H2afy1",4.1089464086629e-13,0.606593777467177,0.884,0.748,6.92850543428738e-09,"6","H2afy"
"Stat5b",4.15923292955646e-13,0.443602592910223,0.341,0.119,7.0132985658181e-09,"6","Stat5b"

"Ppp1ca1",4.37977144703507e-13,0.522280027465073,0.906,0.795,7.3851706
1399053e-09,"6","Ppp1ca"
"Sptbn11",4.89394167630364e-13,0.472472650766656,0.384,0.151,8.2521644
5458319e-09,"6","Sptbn1"
"Mkrn11",5.33088690688198e-13,0.38792224349934,0.681,0.397,8.988941502
38439e-09,"6","Mkrn1"
"Klhl24",5.54121620412403e-13,0.599748023967174,0.587,0.305,9.34359876
339394e-09,"6","Klhl24"
"Psme1",6.32623986157111e-13,0.537796054940409,0.935,0.755,1.066730565
45812e-08,"6","Psme1"
"Pfn12",9.6840498200365e-13,0.434715231676802,0.993,0.943,1.6329244806
5455e-08,"6","Pfn1"
"Usp32",9.80363101751108e-13,0.596789886124811,0.312,0.109,1.653088262
17272e-08,"6","Usp32"
"Ibtk",9.83777104572392e-13,0.41489836383142,0.304,0.104,1.65884495372
997e-08,"6","Ibtk"
"Gnpnat1",1.06627178021236e-12,0.365885349080083,0.261,0.08,1.79794747
579408e-08,"6","Gnpnat1"
"Capns1",1.12660796511835e-12,0.481051973967668,0.862,0.676,1.89968635
078256e-08,"6","Capns1"
"Msr11",1.20666735174522e-12,0.585650157691165,0.304,0.103,2.034682488
5128e-08,"6","Msr1"
"Siva11",1.28233206873516e-12,0.301190823797589,0.37,0.14,2.1622683343
0122e-08,"6","Siva1"
"Rassf4",1.65801896488214e-12,0.575222555051457,0.761,0.503,2.79575157
858427e-08,"6","Rassf4"
"Atg31",1.8047511656017e-12,0.5642166625508,0.667,0.402,3.043171415437
59e-08,"6","Atg3"
"Tmed5",1.82606747482936e-12,0.539844351141016,0.884,0.718,3.079114976
05727e-08,"6","Tmed5"
"Eno11",1.98462908126035e-12,0.512038529657424,0.841,0.665,3.346481556
8212e-08,"6","Eno1"
"Mical1",2.00972850468478e-12,0.350911371688283,0.326,0.116,3.38880420
459947e-08,"6","Mical1"
"Lyst",2.08187085762588e-12,0.501612504680594,0.471,0.219,3.5104506401
2876e-08,"6","Lyst"
"Mta3",2.29015923826481e-12,0.386229302122958,0.312,0.11,3.86166650756
212e-08,"6","Mta3"
"Lsm14a",2.48147479715784e-12,0.505060251543098,0.659,0.371,4.18426280
296754e-08,"6","Lsm14a"
"Zfp106",2.94311344615461e-12,0.645802870157157,0.594,0.332,4.96267789
29059e-08,"6","Zfp106"
"Kat2b",2.95155495992915e-12,0.374788409600122,0.341,0.129,4.976911973
43254e-08,"6","Kat2b"
"Glipr11",2.97841603853461e-12,0.579254537186537,0.63,0.355,5.02220512
417707e-08,"6","Glipr1"
"Psmb10",3.10143859323854e-12,0.688476309372218,0.739,0.512,5.22964575
591883e-08,"6","Psmb10"
"Cript",3.13192728599973e-12,0.4871625255268,0.616,0.34,5.281055789652
74e-08,"6","Cript"

"Ppp2cb",3.1711027736466e-12,0.423611555085555,0.413,0.177,5.34711349692289e-08,"6","Ppp2cb"
"Map3k14",3.80186051393469e-12,0.478700898730211,0.486,0.239,6.41069719859668e-08,"6","Map3k14"
"Rtp41",4.03741906221609e-12,0.640857402706274,0.406,0.167,6.80789602270877e-08,"6","Rtp4"
"Jpt11",4.23405639993781e-12,0.460475746411411,0.877,0.714,7.13946590157514e-08,"6","Jpt1"
"Hck",4.43855636018688e-12,0.63980924369169,0.841,0.687,7.48429373454711e-08,"6","Hck"
"Kmt2e1",5.24109916167306e-12,0.578688078096802,0.812,0.571,8.83754140641311e-08,"6","Kmt2e"
"Ogt",5.26420719400011e-12,0.712939321181205,0.725,0.458,8.87650617052299e-08,"6","Ogt"
"Lmo4",5.47248709507967e-12,0.743370227594319,0.63,0.362,9.22770773972334e-08,"6","Lmo4"
"Cd741",5.96182235781236e-12,2.6554671913705,0.674,0.456,1.00528248597432e-07,"6","Cd74"
"Foxk1",6.11632548630771e-12,0.351779611633626,0.297,0.104,1.03133480350121e-07,"6","Foxk1"
"Cd481",7.15336543686357e-12,0.516229943396094,0.812,0.588,1.20620047996393e-07,"6","Cd48"
"Pptc7",8.2123061199478e-12,0.416168600302915,0.319,0.121,1.3847590579456e-07,"6","Pptc7"
"Rab32",8.28446270923104e-12,0.435948392847757,0.486,0.23,1.39692610203054e-07,"6","Rab32"
"Map3k5",8.86833994632798e-12,0.512244691456493,0.333,0.131,1.49537948174982e-07,"6","Map3k5"
"Krit1",9.03370917705465e-12,0.537675044191072,0.435,0.196,1.52326404143495e-07,"6","Krit1"
"Ncor1",9.51523681787816e-12,0.559207380882463,0.848,0.624,1.60445923223062e-07,"6","Ncor1"
"Cdk14",1.09302591135523e-11,0.547826506363902,0.413,0.178,1.84306029172718e-07,"6","Cdk14"
"Rplp02",1.19750345972305e-11,0.427756778120279,0.986,0.985,2.01923033378501e-07,"6","Rplp0"
"Mob1a",1.20345232767114e-11,0.606349317445419,0.725,0.504,2.02926131491907e-07,"6","Mob1a"
"Zzef1",1.20929328615123e-11,0.751672332337912,0.464,0.216,2.03911033910821e-07,"6","Zzef1"
"Lrp1",1.22933142311178e-11,0.590734171587379,0.79,0.621,2.07289864565108e-07,"6","Lrp1"
"Ppp1r12a",1.28882825377332e-11,0.576407176782583,0.775,0.567,2.17322220151257e-07,"6","Ppp1r12a"
"Psap",1.38868095220306e-11,0.424173597124807,0.986,0.96,2.3415938216048e-07,"6","Psap"
"Syf21",1.40534529488028e-11,0.504463335498085,0.739,0.49,2.36969323622712e-07,"6","Syf2"
"Slc12a6",1.60321938874973e-11,0.53915055896163,0.587,0.329,2.7033485333098e-07,"6","Slc12a6"

"Cap1",1.68033593597473e-11,0.551359100851928,0.862,0.684,2.8333824552
4058e-07,"6","Cap1"
"Atg7",1.69790622225385e-11,0.365566639834907,0.297,0.107,2.8630094719
6444e-07,"6","Atg7"
"Txnip1",1.71621846580389e-11,0.487473082207084,0.638,0.366,2.89388757
703851e-07,"6","Txnip"
"Tmpo1",1.78127123485463e-11,0.481960172510749,0.558,0.284,3.003579556
21188e-07,"6","Tmpo"
"Mthfd21",1.96949665402874e-11,0.313397633220946,0.29,0.101,3.32096525
802326e-07,"6","Mthfd2"
"Cdkn1a",2.02916447480291e-11,0.379464250163869,0.812,0.526,3.42157713
741266e-07,"6","Cdkn1a"
"Fis1",2.06463220389915e-11,0.469670722058683,0.797,0.576,3.4813828222
1475e-07,"6","Fis1"
"Mir142hg",2.09643947501483e-11,0.536191636326389,0.348,0.14,3.5350162
4277001e-07,"6","Mir142hg"
"Baz1a",2.11138597137281e-11,0.548401271937273,0.71,0.457,3.5602190249
2883e-07,"6","Baz1a"
"Stk4",2.20750732001964e-11,0.535769907892009,0.587,0.335,3.7222988430
1711e-07,"6","Stk4"
"D1Ertd622e",2.22159962335836e-11,0.472623862881356,0.536,0.284,3.7460
6128490687e-07,"6","D1Ertd622e"
"Mapkapk21",2.3095434687422e-11,0.497557862336039,0.899,0.713,3.894352
19699309e-07,"6","Mapkapk2"
"Baz2b",2.69393914886376e-11,0.567579958602662,0.616,0.346,4.542520192
81408e-07,"6","Baz2b"
"Elovl5",2.93624537939494e-11,0.339287207717797,0.275,0.096,4.95109695
873575e-07,"6","Elovl5"
"Baz2a",3.13548380050439e-11,0.425276716648987,0.478,0.229,5.287052784
4105e-07,"6","Baz2a"
"Rnf2131",3.24642612369038e-11,0.604964531734576,0.428,0.205,5.4741237
2976673e-07,"6","Rnf213"
"Pmaip1",3.57522273269398e-11,0.587764104274449,0.522,0.294,6.02854057
186859e-07,"6","Pmaip1"
"Clec2d1",3.98845106022707e-11,0.346480922651157,0.362,0.143,6.7253261
7775489e-07,"6","Clec2d"
"Tapbp",4.30752633085729e-11,0.569692185299297,0.812,0.624,7.263350899
09157e-07,"6","Tapbp"
"Cycs",4.38489444249962e-11,0.554996376867679,0.616,0.387,7.3938090089
4286e-07,"6","Cycs"
"Hnrnpa31",4.49472338498849e-11,0.471914915238709,0.913,0.77,7.5790025
717676e-07,"6","Hnrnpa3"
"Slc44a2",5.52367014766717e-11,0.648753480258508,0.551,0.315,9.3140126
0299638e-07,"6","Slc44a2"
"Acaa2",5.84522090873911e-11,0.477028711483759,0.522,0.272,9.856211496
31588e-07,"6","Acaa2"
"Ldlr1",6.28045704738692e-11,0.38948038432601,0.341,0.134,1.0590106673
3038e-06,"6","Ldlr"
"Anxa6",6.69281162807643e-11,0.514402360296132,0.507,0.269,1.128541896
72625e-06,"6","Anxa6"

"Jak2",6.83083504626444e-11,0.433886840930483,0.384,0.168,1.1518154055
0111e-06,"6","Jak2"
"Ybx12",6.88755283294358e-11,0.295223914633195,0.964,0.893,1.161379158
69095e-06,"6","Ybx1"
"Sfxn3",7.2902220921863e-11,0.313949200144692,0.326,0.126,1.2292772491
8445e-06,"6","Sfxn3"
"Usp25",7.40910087394985e-11,0.602004422423084,0.551,0.327,1.249322589
36542e-06,"6","Usp25"
"Samsn11",7.8671923165302e-11,0.674726375410827,0.79,0.597,1.326565968
41332e-06,"6","Samsn1"
"Pdcd6",8.4916533442729e-11,0.360055954331382,0.638,0.369,1.4318625869
113e-06,"6","Pdcd6"
"Rock1",9.37799206997033e-11,0.653095344715216,0.761,0.549,1.581317022
8384e-06,"6","Rock1"
"Sppl2a",1.00320739926219e-10,0.686748486682875,0.71,0.523,1.691608316
6359e-06,"6","Sppl2a"
"Usf3",1.18923215240384e-10,0.351948299601214,0.304,0.114,2.0052832553
8336e-06,"6","Usf3"
"Nop53",1.19474098202116e-10,0.487190904752647,0.746,0.541,2.014572243
88409e-06,"6","Nop53"
"Tab2",1.24591530725171e-10,0.595848570715402,0.696,0.492,2.1008623910
8783e-06,"6","Tab2"
"Uhrf2",1.33987818884761e-10,0.496398716101548,0.558,0.311,2.259302602
03484e-06,"6","Uhrf2"
"Gpsm3",1.38387101354046e-10,0.522598121525933,0.804,0.614,2.333483303
03192e-06,"6","Gpsm3"
"Per1",1.45867681176068e-10,0.572761567454166,0.558,0.313,2.4596208399
9086e-06,"6","Per1"
"Mxi11",1.86908222091875e-10,0.552760806424031,0.58,0.352,3.1516464409
132e-06,"6","Mxi1"
"H2afj",1.90325621578601e-10,0.583250440575634,0.543,0.316,3.209270631
05836e-06,"6","H2afj"
"Pak1ip11",1.93735841137046e-10,0.413071306147861,0.594,0.327,3.266773
75325287e-06,"6","Pak1ip1"
"Irf51",1.96710305919691e-10,0.505163514833775,0.877,0.704,3.316929178
41782e-06,"6","Irf5"
"Cpt1a",2.08772997148763e-10,0.40107739398104,0.355,0.149,3.5203302779
2244e-06,"6","Cpt1a"
"Eif3f1",2.20189842156874e-10,0.429785657533239,0.957,0.864,3.71284111
84492e-06,"6","Eif3f"
"Lyn",2.32617806221854e-10,0.556408523196538,0.935,0.819,3.92240144851
29e-06,"6","Lyn"
"Eps81",2.41857864804134e-10,0.373021066213009,0.304,0.116,4.078207316
32732e-06,"6","Eps8"
"Atp5c12",2.5963960026769e-10,0.419365185902822,0.906,0.769,4.37804293
971379e-06,"6","Atp5c1"
"Lilra51",2.60779000219091e-10,0.567651753222692,0.297,0.118,4.3972555
0169432e-06,"6","Lilra5"
"Mycbp21",2.75393515903537e-10,0.594359325735897,0.754,0.588,4.6436854
6516544e-06,"6","Mycbp2"

"Abr",2.90875783753557e-10,0.43767549403332,0.58,0.325,4.9047474656524
8e-06,"6","Abr"
"Med15",2.91757232358171e-10,0.483860552021627,0.413,0.191,4.919610452
02347e-06,"6","Med15"
"Sf3b61",2.94690912345907e-10,0.410532987480613,0.717,0.475,4.96907816
397669e-06,"6","Sf3b6"
"Stxbp2",3.12511234581287e-10,0.423376253825888,0.551,0.317,5.26956443
750966e-06,"6","Stxbp2"
"Nfe2l22",3.18130115752732e-10,0.568409297597424,0.877,0.716,5.3643100
1182256e-06,"6","Nfe2l2"
"Kctd10",3.53860598359307e-10,0.368573091587617,0.326,0.136,5.96679740
953463e-06,"6","Kctd10"
"Pip4k2a",3.60191137981003e-10,0.574512377996638,0.725,0.535,6.0735429
6863566e-06,"6","Pip4k2a"
"Grk2",3.77502751188927e-10,0.473341452417642,0.739,0.548,6.3654513905
4769e-06,"6","Grk2"
"Gnai2",3.99154942991342e-10,0.326241715013185,0.986,0.964,6.730550648
72002e-06,"6","Gnai2"
"Tes",4.00704816158717e-10,0.424134702388468,0.254,0.091,6.75668461006
828e-06,"6","Tes"
"Ago2",4.06189322799663e-10,0.510448635216234,0.507,0.285,6.8491643610
4792e-06,"6","Ago2"
"Fam96a1",4.18747655869307e-10,0.457239070505549,0.739,0.482,7.0609229
7326825e-06,"6","Fam96a"
"Cox17",4.26144921368343e-10,0.504800612955777,0.522,0.291,7.185655664
113e-06,"6","Cox17"
"Taok3",4.31447436420883e-10,0.55899225650047,0.739,0.495,7.2750666729
2893e-06,"6","Taok3"
"Dusp221",4.38727682533946e-10,0.387899988153592,0.399,0.186,7.3978261
828874e-06,"6","Dusp22"
"Psmb92",4.69010668193466e-10,0.688503841411447,0.572,0.365,7.90845788
707822e-06,"6","Psmb9"
"Nfatc3",4.69727676100183e-10,0.336482454117914,0.37,0.164,7.920548074
40128e-06,"6","Nfatc3"
"Tet21",4.72821508868519e-10,0.483526709977752,0.543,0.304,7.972716282
54097e-06,"6","Tet2"
"Kif5b",5.19527487118115e-10,0.495059144971797,0.877,0.702,8.760272487
78565e-06,"6","Kif5b"
"Cbl1",5.26534244633093e-10,0.599407130109867,0.71,0.489,8.87842043300
322e-06,"6","Cbl"
"Wdfy4",5.45204373346193e-10,0.438236511566435,0.428,0.208,9.193236143
36351e-06,"6","Wdfy4"
"Rilpl22",5.90019244528505e-10,0.441184737607097,0.754,0.477,9.9489045
0123965e-06,"6","Rilpl2"
"Cox5a1",5.92233743103415e-10,0.37251527061445,0.812,0.613,9.986245376
20979e-06,"6","Cox5a"
"Ist1",6.21490878030471e-10,0.407373584180712,0.543,0.293,1.0479579185
3498e-05,"6","Ist1"
"Cfap43",6.24836692970745e-10,0.326586955673455,0.297,0.114,1.05359963
168727e-05,"6","Cfap43"

"Hfe1",6.87558323085169e-10,0.614887070403775,0.688,0.461,1.1593608443
8621e-05,"6","Hfe"
"Cmpk11",6.92156975204264e-10,0.415080479462425,0.797,0.586,1.16711509
158943e-05,"6","Cmpk1"
"Ucp2",7.17408143780297e-10,0.411283243058264,0.942,0.898,1.2096936120
4234e-05,"6","Ucp2"
"Itch",7.29234583549507e-10,0.600183011694511,0.609,0.394,1.2296353547
8118e-05,"6","Itch"
"Scp2",7.51334530714403e-10,0.44453076447395,0.819,0.635,1.26690028569
063e-05,"6","Scp2"
"Ppp1r2",7.59495094218828e-10,0.41705302040364,0.688,0.469,1.280660627
87179e-05,"6","Ppp1r2"
"Lrrc8d",7.69396498916442e-10,0.478518196537618,0.471,0.248,1.29735637
64729e-05,"6","Lrrc8d"
"Phf11b2",8.0014055006258e-10,0.444356613503306,0.442,0.215,1.34919699
551552e-05,"6","Phf11b"
"Mia2",8.06448976225621e-10,0.49193829924239,0.775,0.561,1.35983426371
164e-05,"6","Mia2"
"Sh3bgrl31",8.23880156029809e-10,0.388727274872799,0.964,0.913,1.38922
671909746e-05,"6","Sh3bgrl3"
"H13",8.24094377868828e-10,0.36848588535595,0.659,0.407,1.389587939962
42e-05,"6","H13"
"Rsrp11",8.27216792413887e-10,0.456017314812442,0.899,0.731,1.39485295
53683e-05,"6","Rsrp1"
"Uba6",8.39789083134185e-10,0.314701063475001,0.29,0.108,1.41605235198
086e-05,"6","Uba6"
"Gbp31",8.4037202747349e-10,0.448033376994201,0.304,0.129,1.4170353127
258e-05,"6","Gbp3"
"Plagl2",8.95481964188224e-10,0.520896578456665,0.399,0.19,1.509961688
01418e-05,"6","Plagl2"
"Piezo1",9.48728820207919e-10,0.264342589410726,0.326,0.132,1.59974653
663459e-05,"6","Piezo1"
"Nmi",1.01264269614251e-09,0.396450171921675,0.391,0.185,1.70751811423
55e-05,"6","Nmi"
"Fcer1g1",1.01523684906104e-09,0.462599921807357,0.986,0.965,1.7118923
7488673e-05,"6","Fcer1g"
"Fam234a1",1.0238547532688e-09,0.314680155673474,0.384,0.171,1.7264238
8496184e-05,"6","Fam234a"
"Rpl71",1.09944408186443e-09,0.322925914879784,0.978,0.971,1.853882610
8398e-05,"6","Rpl7"
"Myl12a1",1.11006454460236e-09,0.453569314124656,0.928,0.782,1.8717908
3510849e-05,"6","Myl12a"
"Rps31",1.14083307448709e-09,0.314835328150362,0.986,0.974,1.923672730
20014e-05,"6","Rps3"
"Tspyl1",1.15137747746702e-09,0.33402743807567,0.377,0.171,1.941452702
50489e-05,"6","Tspyl1"
"Gsto1",1.1614740852625e-09,0.35061046112647,0.572,0.329,1.95847760256
964e-05,"6","Gsto1"
"Aip",1.18457745589234e-09,0.421733585226131,0.471,0.256,1.99743450612
567e-05,"6","Aip"

"Csf2rb1",1.29848467681487e-09,0.508570662184954,0.674,0.441,2.1895048
6204523e-05,"6","Csf2rb"
"Tcf7l2",1.39011600336795e-09,0.70289805132705,0.391,0.198,2.344013604
87903e-05,"6","Tcf7l2"
"Fam20c",1.40077425129551e-09,0.307475613206114,0.275,0.104,2.36198554
253449e-05,"6","Fam20c"
"Ythdf3",1.45814129470511e-09,0.469480733826745,0.543,0.3,2.4587178511
3176e-05,"6","Ythdf3"
"Oasl21",1.52719009241848e-09,0.514985734550475,0.275,0.104,2.57514793
383604e-05,"6","Oasl2"
"Ctnna1",1.57004935234413e-09,0.42365669073812,0.493,0.266,2.647417217
92267e-05,"6","Ctnna1"
"Uvrag1",1.62398963633945e-09,0.489477732405902,0.587,0.366,2.73837132
479558e-05,"6","Uvrag"
"Coro2a",1.68605044109213e-09,0.270163977116209,0.348,0.142,2.84301825
376955e-05,"6","Coro2a"
"Ppp1cb",1.73660728850711e-09,0.472927177187615,0.551,0.344,2.92826720
988069e-05,"6","Ppp1cb"
"Ugcg",1.77766989452194e-09,0.391417844927624,0.399,0.188,2.9975069761
4289e-05,"6","Ugcg"
"Kpna4",1.90363084122066e-09,0.584077116147464,0.717,0.526,3.209902324
46628e-05,"6","Kpna4"
"Tap11",2.16693693280931e-09,0.628625972794122,0.58,0.362,3.6538890561
0306e-05,"6","Tap1"
"Fbxo4",2.24916765398409e-09,0.477791509688133,0.355,0.163,3.792546498
14798e-05,"6","Fbxo4"
"Igf2bp3",2.26197395434047e-09,0.353598651254448,0.384,0.182,3.8141404
8180891e-05,"6","Igf2bp3"
"Stat21",2.42375822013864e-09,0.298526518409708,0.304,0.121,4.08694111
079778e-05,"6","Stat2"
"Lman21",2.44056951437101e-09,0.510630274214398,0.725,0.507,4.11528831
51324e-05,"6","Lman2"
"Lcp2",2.49614194093604e-09,0.566001186058783,0.768,0.573,4.2089945408
0635e-05,"6","Lcp2"
"Rps3a11",2.62617698603026e-09,0.262859195626871,0.993,0.983,4.4282596
3384423e-05,"6","Rps3a1"
"Cdc421",2.63595704695092e-09,0.290043081297497,0.978,0.956,4.44475077
256864e-05,"6","Cdc42"
"Kpna21",2.63946810798849e-09,0.271742786837684,0.304,0.124,4.45067112
36902e-05,"6","Kpna2"
"Coq10b1",2.80818291711714e-09,0.434888207949629,0.804,0.563,4.7351580
3484292e-05,"6","Coq10b"
"Gcnt21",3.02970452333097e-09,0.657349345638531,0.457,0.245,5.10868776
724068e-05,"6","Gcnt2"
"Atxn7l1",3.13419442686362e-09,0.395891185582902,0.333,0.148,5.2848786
4257744e-05,"6","Atxn7l1"
"Dock2",3.23449458454446e-09,0.532975384292602,0.79,0.628,5.4540047684
5887e-05,"6","Dock2"
"Sec24b",3.34791618361278e-09,0.315798135164822,0.406,0.198,5.64525626
880787e-05,"6","Sec24b"

"Lrrfip2",3.50282065812113e-09,0.394223805967986,0.42,0.214,5.90645619372385e-05,"6","Lrrfip2"
"Ap2a21",3.81759512886205e-09,0.434761901214296,0.457,0.243,6.43722890628719e-05,"6","Ap2a2"
"Dr1",3.93890932527347e-09,0.48541215111192,0.399,0.194,6.64178890427613e-05,"6","Dr1"
"Slc1a5",4.29302604130029e-09,0.299109167358068,0.268,0.102,7.23890051084055e-05,"6","Slc1a5"
"G6pdx",4.52497111460306e-09,0.393860482895946,0.377,0.184,7.63000629344368e-05,"6","G6pdx"
"Sp1101",4.83535234008937e-09,0.395778249472347,0.522,0.305,8.15337111585869e-05,"6","Sp110"
"Erp44",4.95670945384874e-09,0.390522999782178,0.601,0.369,8.35800348107974e-05,"6","Erp44"
"Rac1",5.01405062747657e-09,0.356395539506158,0.935,0.852,8.45469216805099e-05,"6","Rac1"
"Slc35b11",5.08234029907344e-09,0.301631362185172,0.522,0.278,8.56984221229764e-05,"6","Slc35b1"
"Pdcd10",5.32933052742647e-09,0.445109450601087,0.696,0.491,8.98631713534651e-05,"6","Pdcd10"
"Rbm32",5.39789856948507e-09,0.374962810159411,0.942,0.923,9.10193656786572e-05,"6","Rbm3"
"Rftn1",5.75273130555467e-09,0.276508835117767,0.341,0.151,9.70025552742629e-05,"6","Rftn1"
"Ptpn13",6.09961516930958e-09,0.560110879841746,0.884,0.793,0.000102851710984898,"6","Ptpn1"
"Grpel11",6.29346171559012e-09,0.306485978546936,0.558,0.322,0.000106120351448281,"6","Grpel1"
"Xbp11",6.33435915967605e-09,0.56282423723525,0.754,0.556,0.000106809964150458,"6","Xbp1"
"Mfsd14a",6.50588420120331e-09,0.442119776012128,0.507,0.294,0.00010970221940069,"6","Mfsd14a"
"Chfr",6.82478722497221e-09,0.369975889702102,0.312,0.136,0.000115079562187481,"6","Chfr"
"Tax1bp1",7.32645504358771e-09,0.466534705525596,0.804,0.604,0.000123538684944976,"6","Tax1bp1"
"Ptpn11",7.36527512129705e-09,0.488231639284236,0.507,0.289,0.000124193269095311,"6","Ptpn11"
"Lsm41",7.56919377805348e-09,0.312368463191572,0.652,0.398,0.000127631745485538,"6","Lsm4"
"Actr2",7.638136753766e-09,0.399492937431826,0.913,0.751,0.000128794261942002,"6","Actr2"
"Ywhaz1",8.28290959900986e-09,0.541381613556974,0.79,0.679,0.000139666421658504,"6","Ywhaz"
"Slc25a51",8.36445949271098e-09,0.329573813920876,0.949,0.893,0.000141041515966092,"6","Slc25a5"
"Camk2d",8.37708018200901e-09,0.717252823812806,0.703,0.553,0.000141254326029036,"6","Camk2d"
"Napg",8.57359795663993e-09,0.34085798113498,0.442,0.226,0.000144568008744862,"6","Napg"

"Crebbp",8.64174673243986e-09,0.454636439966688,0.652,0.434,0.000145717133402401,"6","Crebbp"
"Dpm1",8.68210303719625e-09,0.368191232634305,0.413,0.203,0.000146397621413203,"6","Dpm1"
"Eif3e",9.01692199058937e-09,0.385555260407902,0.819,0.653,0.000152043338605318,"6","Eif3e"
"Atp6v0e",9.20072824624864e-09,0.368080241839793,0.891,0.802,0.000155142679688245,"6","Atp6v0e"
"Tmem234",1.02306885260565e-08,0.403327763647842,0.797,0.62,0.000172509869926364,"6","Tmem234"
"Slc2a6",1.07749327937716e-08,0.272057022663077,0.326,0.142,0.000181686916768576,"6","Slc2a6"
"Brd4",1.15392921304263e-08,0.442132909762315,0.696,0.474,0.000194575543903249,"6","Brd4"
"Tiparp1",1.16735579926051e-08,0.416119578242277,0.587,0.342,0.000196839534871307,"6","Tiparp"
"Myo1c1",1.17124017846729e-08,0.426577706952491,0.5,0.28,0.000197494518893154,"6","Myo1c"
"Synj11",1.26524387828661e-08,0.459852898632187,0.551,0.349,0.000213345422756689,"6","Synj1"
"Pxn",1.4021176765284e-08,0.42759705282495,0.565,0.345,0.000236425082616219,"6","Pxn"
"Psmd81",1.40898926354806e-08,0.304574656897713,0.841,0.672,0.000237583769619475,"6","Psmd8"
"Csnk2b",1.5078356147591e-08,0.442433411128498,0.688,0.476,0.000254251241360679,"6","Csnk2b"
"Cop1",1.56961776419319e-08,0.45650568967813,0.514,0.302,0.000264668947398256,"6","Cop1"
"Pvr1",1.59489029448901e-08,0.315922994007687,0.399,0.193,0.000268930401456737,"6","Pvr"
"Phyh",1.59976296012622e-08,0.420432742459796,0.442,0.239,0.000269752030336483,"6","Phyh"
"Serp11",1.72545047743178e-08,0.380425325113527,0.855,0.712,0.000290945459504547,"6","Serp1"
"Dstyk",1.76505612600128e-08,0.384818633238156,0.283,0.116,0.000297623763966336,"6","Dstyk"
"Psmb51",1.87793451239862e-08,0.384557645771488,0.775,0.607,0.000316657317480656,"6","Psmb5"
"Cryzl1",1.90466306208666e-08,0.289399474791987,0.319,0.137,0.000321164285529052,"6","Cryzl1"
"Map3k3",1.99956481647314e-08,0.418923410430178,0.442,0.237,0.000337166619353701,"6","Map3k3"
"Aurkaip11",2.0877500962006e-08,0.278387078662585,0.645,0.398,0.000352036421221345,"6","Aurkaip1"
"Sptan1",2.16901170075783e-08,0.381849922144702,0.464,0.252,0.000365738752981785,"6","Sptan1"
"Cd302",2.19509652941436e-08,0.425351919012978,0.659,0.43,0.00037013717678985,"6","Cd302"
"Scarb1",2.26664494755772e-08,0.479022155493266,0.428,0.226,0.000382201671057183,"6","Scarb1"

"Far1",2.40705505062901e-08,0.419897886530196,0.471,0.27,0.00040587762
2637064,"6","Far1"
"Sri1",2.42978802762888e-08,0.333504730797187,0.71,0.495,0.00040971085
7218781,"6","Sri"
"Atf7ip",2.46610904070143e-08,0.404379214360473,0.457,0.248,0.00041583
5306443074,"6","Atf7ip"
"Nemf",2.47317428430897e-08,0.433857115428115,0.449,0.246,0.0004170266
47820178,"6","Nemf"
"Pcgf5",2.6414951996203e-08,0.343597772384976,0.333,0.158,0.0004454089
20559976,"6","Pcgf5"
"Mpp12",2.73424721670262e-08,0.390672110896269,0.514,0.294,0.000461048
765680396,"6","Mpp1"
"Tgfb1",2.75326352590479e-08,0.507298374588922,0.92,0.812,0.0004642552
95738065,"6","Tgfb1"
"Gpi11",2.77113651746608e-08,0.395488599095513,0.877,0.782,0.000467269
03957513,"6","Gpi1"
"Atp6v1e1",2.8392229418748e-08,0.424210064036269,0.761,0.592,0.0004787
49772458929,"6","Atp6v1e1"
"Sp1401",2.84209931402037e-08,0.372186207959387,0.435,0.238,0.00047923
4786330115,"6","Sp140"
"Frg1",2.89721650987925e-08,0.384213553712191,0.543,0.336,0.0004885286
47895839,"6","Frg1"
"Atp5l",3.0855560895136e-08,0.567440859613782,0.826,0.654,0.0005202864
67813783,"6","Atp5l"
"Ubl32",3.31336182710137e-08,0.487469431686494,0.826,0.686,0.000558699
071285832,"6","Ubl3"
"Rpl18",3.61686978683659e-08,0.405888834830776,0.964,0.944,0.000609876
583456386,"6","Rpl18"
"Flii",3.62951927547227e-08,0.360030575733347,0.609,0.398,0.0006120095
40230135,"6","Flii"
"Smim141",3.74933575946132e-08,0.372671496678593,0.652,0.453,0.0006322
12995760368,"6","Smim14"
"Rb1",3.75475205783882e-08,0.330109143336078,0.362,0.174,0.00063312629
1992781,"6","Rb1"
"Atg4d",3.8230477403265e-08,0.311009087661255,0.319,0.142,0.0006446423
09973854,"6","Atg4d"
"Map4k41",3.87806827713043e-08,0.444604782854136,0.761,0.585,0.0006539
19872889733,"6","Map4k4"
"Myo9b",3.91035392580175e-08,0.343135786451824,0.536,0.323,0.000659363
878968691,"6","Myo9b"
"Tle3",4.72244272188135e-08,0.500571343074689,0.399,0.213,0.0007962982
91763634,"6","Tle3"
"Sra1",4.73453333106877e-08,0.35257382811279,0.587,0.378,0.00079833701
0284816,"6","Sra1"
"Esyt1",4.86237303235468e-08,0.306932233492882,0.37,0.179,0.0008198933
40715646,"6","Esyt1"
"Nupr11",4.90893676439438e-08,0.309659525053646,0.493,0.275,0.00082774
491721218,"6","Nupr1"
"Sat1",5.14161267757038e-08,0.4617902863843,0.971,0.915,0.000866978729
691917,"6","Sat1"

"Atmin",5.14679404289994e-08,0.364542779577413,0.283,0.12,0.0008678524
11513789,"6","Atmin"
"Tfeb",5.22791590979285e-08,0.33980514347285,0.377,0.183,0.00088153118
070927,"6","Tfeb"
"Naga",5.31867594822394e-08,0.505182700998405,0.616,0.426,0.0008968351
3838952,"6","Naga"
"Phf20l11",5.46180426085097e-08,0.364681283031896,0.674,0.433,0.000920
969434464691,"6","Phf20l1"
"Stx8",5.46306793299042e-08,0.319225006304378,0.507,0.289,0.0009211825
14860845,"6","Stx8"
"Filip1l1",5.4772684013415e-08,0.533256708326258,0.536,0.327,0.0009235
76997834204,"6","Filip1l"
"Irak4",5.8518707156416e-08,0.296153953836142,0.384,0.19,0.00098674244
0071487,"6","Irak4"
"Pten",6.47454001122058e-08,0.527807797316927,0.739,0.528,0.0010917369
3669201,"6","Pten"
"Setx",6.97670638646483e-08,0.352524372870511,0.37,0.183,0.00117641223
08857,"6","Setx"
"Phip",7.02243471726948e-08,0.478818478953003,0.587,0.394,0.0011841229
4202598,"6","Phip"
"Ralgapa1",7.11369309077089e-08,0.300755116945367,0.283,0.12,0.0011995
1092896579,"6","Ralgapa1"
"Tmed10",7.16198231724952e-08,0.457706661019919,0.884,0.766,0.00120765
345833461,"6","Tmed10"
"Cs",7.34573029774295e-08,0.35455725712218,0.471,0.269,0.0012386370428
0542,"6","Cs"
"Nrd1",7.46768440345841e-08,0.421897925094564,0.572,0.365,0.0012592009
4411116,"6","Nrd1"
"Rpl91",7.56444974767367e-08,0.268142503381765,0.978,0.964,0.001275517
51645273,"6","Rpl9"
"Psmb3",7.57199530079872e-08,0.347607280417015,0.717,0.541,0.001276789
84762068,"6","Psmb3"
"Cyld",7.57322161959461e-08,0.40456711321036,0.442,0.249,0.00127699662
949604,"6","Cyld"
"Madd",7.61248192693444e-08,0.384873727067792,0.384,0.196,0.0012836167
0251969,"6","Madd"
"Ywhab1",7.85509852230825e-08,0.356787214291634,0.812,0.699,0.00132452
671283162,"6","Ywhab"
"Pcbp2",7.9846701293626e-08,0.378158147696233,0.841,0.703,0.0013463750
7721312,"6","Pcbp2"
"Fgfr1op2",8.4046850602556e-08,0.414623087602008,0.601,0.386,0.0014171
979948603,"6","Fgfr1op2"
"Litaf1",8.49189973212346e-08,0.418603512284313,0.87,0.729,0.001431904
13283066,"6","Litaf"
"Sf3b51",8.85426305792021e-08,0.3143515953471,0.594,0.377,0.0014930058
3682651,"6","Sf3b5"
"Soat1",8.93940043527269e-08,0.436301982022245,0.58,0.371,0.0015073617
0139568,"6","Soat1"
"Anxa7",9.56220996521296e-08,0.311946257964544,0.536,0.308,0.001612379
84433421,"6","Anxa7"

"Ccl91",9.58012807242784e-08,0.765330467509096,0.674,0.463,0.00161540119557278,"6","Ccl9"
"Rictor",9.6308062547941e-08,0.321575908792974,0.312,0.145,0.00162394655068338,"6","Rictor"
"Sod21",9.67875164541039e-08,0.460474318688429,0.696,0.491,0.0016320311024491,"6","Sod2"
"Fbxl5",9.70729214888516e-08,0.584468683390677,0.406,0.218,0.00163684360214502,"6","Fbxl5"
"Ube2b",9.73841978685233e-08,0.424040780020172,0.761,0.591,0.00164209234445904,"6","Ube2b"
"Tomm221",1.0140818341132e-07,0.3753199844923,0.732,0.55,0.00170994478868168,"6","Tomm22"
"Ppp1r15b1",1.02180272446949e-07,0.541787069640333,0.522,0.315,0.00172296375400046,"6","Ppp1r15b"
"Vasp",1.02276601726202e-07,0.598961916572314,0.826,0.717,0.00172458805830721,"6","Vasp"
"Slfn22",1.05258561163067e-07,0.408715854350705,0.87,0.68,0.00177486985833163,"6","Slfn2"
"Tpr1",1.12782408258034e-07,0.42950041942791,0.855,0.703,0.00190173696804697,"6","Tpr"
"Ak21",1.12950559266467e-07,0.490806071592139,0.609,0.414,0.00190457233035117,"6","Ak2"
"Gabpb2",1.13215044220948e-07,0.338233427901481,0.362,0.186,0.00190903207565363,"6","Gabpb2"
"Gmfg",1.14474863523219e-07,0.481180472289612,0.775,0.626,0.00193027514872851,"6","Gmfg"
"Ing3",1.16729018410337e-07,0.317174488533446,0.319,0.146,0.0019682847084351,"6","Ing3"
"Snf81",1.23880043371502e-07,0.314713512904068,0.587,0.38,0.00208886529133027,"6","Snf8"
"Ube2d31",1.24040616515584e-07,0.309912406772648,0.971,0.879,0.00209157287568578,"6","Ube2d3"
"March2",1.28090421298763e-07,0.274270425946958,0.529,0.307,0.00215986068393975,"6","March2"
"BC028528",1.33311522675708e-07,0.469325605102544,0.587,0.4,0.00224789889535779,"6","BC028528"
"Sept111",1.35411983147407e-07,0.292228789886177,0.268,0.112,0.00228331685983158,"6","Sept11"
"Bcl2a1d2",1.36415220158408e-07,0.588513371368634,0.565,0.387,0.00230023344231107,"6","Bcl2a1d"
"Cox4i11",1.40717445031024e-07,0.293915115756021,0.949,0.896,0.00237277755811313,"6","Cox4i1"
"Stim2",1.45061482400208e-07,0.31341612806312,0.261,0.11,0.0024460267162323,"6","Stim2"
"Ttc14",1.50578653300342e-07,0.358702033858316,0.587,0.373,0.00253905725195037,"6","Ttc14"
"Llph",1.51166415388895e-07,0.401984148224091,0.58,0.364,0.00254896809628754,"6","Llph"
"Nsd3",1.55808775408093e-07,0.607115610304144,0.638,0.455,0.00262724757093127,"6","Nsd3"

"Ugp2",1.64810667759251e-07,0.314488364657345,0.5,0.289,0.0027790374797565,"6","Ugp2"
"Fry",1.72242262034589e-07,0.278245323184965,0.319,0.148,0.00290434902242723,"6","Fry"
"Atp5h",1.81272571227565e-07,0.350961082214757,0.848,0.689,0.0030566180960392,"6","Atp5h"
"Rab1b",1.85782802261625e-07,0.400279347620935,0.478,0.283,0.00313266961173552,"6","Rab1b"
"Ncor2",2.01633476419721e-07,0.411679450872365,0.493,0.304,0.00339994367938933,"6","Ncor2"
"Cib1",2.07927970017409e-07,0.286432431201561,0.442,0.244,0.00350608143043355,"6","Cib1"
"Tor1a1",2.09883034731633e-07,0.295289819288949,0.572,0.357,0.0035390477316448,"6","Tor1a"
"Nsf",2.13927904722688e-07,0.398102062981523,0.493,0.289,0.00360725232943397,"6","Nsf"
"Adss",2.18891222470971e-07,0.288203373533929,0.428,0.234,0.00369094379330551,"6","Adss"
"Tspo1",2.19207735629985e-07,0.346786203626488,0.841,0.717,0.0036962808381928,"6","Tspo"
"Cept1",2.2220005148986e-07,0.35932798749617,0.623,0.427,0.00374673726822202,"6","Cept1"
"Cdkn2aipnl1",2.36969079494457e-07,0.2537439275804,0.275,0.121,0.00399577261843554,"6","Cdkn2aipnl"
"Psma42",2.49690961820873e-07,0.384975346468752,0.725,0.567,0.00421028899822356,"6","Psma4"
"Smc61",2.58647186409478e-07,0.620266952515698,0.543,0.379,0.00436130885723661,"6","Smc6"
"1110008F13Rik1",2.77809608707588e-07,0.338065875403576,0.804,0.629,0.00468442562202735,"6","1110008F13Rik"
"Cab39",2.99008572318525e-07,0.336510146072733,0.572,0.383,0.00504188254643497,"6","Cab39"
"Cnih4",3.02580075593673e-07,0.331421861510171,0.558,0.354,0.00510210523466052,"6","Cnih4"
"Eif1ax1",3.03355979810295e-07,0.332850758957691,0.486,0.286,0.00511518853156119,"6","Eif1ax"
"Cope",3.09711400679735e-07,0.395091060399953,0.696,0.534,0.00522235363826169,"6","Cope"
"Arap1",3.14824249577046e-07,0.356148790304743,0.507,0.313,0.00530856649636815,"6","Arap1"
"Dlst",3.33174090217394e-07,0.365404729051266,0.58,0.389,0.0056179815092457,"6","Dlst"
"Ncoa1",3.33421529469697e-07,0.348712352110075,0.326,0.158,0.00562215382991803,"6","Ncoa1"
"Casp1",3.34421593682389e-07,0.306361987770254,0.543,0.327,0.00563901691267245,"6","Casp1"
"Akap13",3.379491314836e-07,0.498743890694454,0.826,0.696,0.00569849825507646,"6","Akap13"
"Nrbp1",3.45243658739648e-07,0.273104534689179,0.616,0.386,0.00582149857366795,"6","Nrbp1"

"Uqcrc1",3.50307565760406e-07,0.339914216197203,0.696,0.498,0.00590688617385196,"6","Uqcrc1"
"Ramp1",3.53260123239977e-07,0.392624267000108,0.507,0.302,0.00595667219807249,"6","Ramp1"
"Zdhhc21",3.54263295558057e-07,0.270310269659481,0.261,0.113,0.00597358768969995,"6","Zdhhc21"
"Snrpc1",3.66085121380583e-07,0.386160495519908,0.551,0.352,0.00617292731671939,"6","Snrpc"
"Prkcb",3.80815229969369e-07,0.353398386654263,0.507,0.32,0.0064213064077435,"6","Prkcb"
"Celf2",3.91829072087266e-07,0.414949110232887,0.862,0.729,0.00660702181353547,"6","Celf2"
"Sephs2",3.95590385718715e-07,0.326859244367758,0.319,0.155,0.00667044508398898,"6","Sephs2"
"Preb",3.96575903815926e-07,0.364625387128299,0.42,0.228,0.00668706289014415,"6","Preb"
"Tmem131l",4.19701026027285e-07,0.308162462604871,0.348,0.168,0.00707699870087208,"6","Tmem131l"
"Slc38a11",4.31154028111604e-07,0.448157609260064,0.659,0.444,0.00727011922201787,"6","Slc38a1"
"Pfdn61",4.42577139750934e-07,0.286841025518651,0.42,0.236,0.00746273573048024,"6","Pfdn6"
"Etv6",4.61376046984853e-07,0.376398868969734,0.449,0.278,0.00777972290425859,"6","Etv6"
"Ldlrap1",4.98685664934695e-07,0.26245683722533,0.326,0.16,0.00840883768212883,"6","Ldlrap1"
"Tmem167",5.12663756610193e-07,0.386544785970733,0.572,0.381,0.00864453626396107,"6","Tmem167"
"Mrpl14",5.24718035333494e-07,0.374745284175134,0.384,0.218,0.00884779551179338,"6","Mrpl14"
"Rab6a",5.27795366630746e-07,0.279347553970172,0.5,0.309,0.00889968547212764,"6","Rab6a"
"Arrb1",5.29263066869706e-07,0.275151271969769,0.341,0.167,0.00892443383355699,"6","Arrb1"
"Lypla2",5.33347083957013e-07,0.391094365704748,0.326,0.162,0.00899329852968316,"6","Lypla2"
"Hdgf1",5.65609942126557e-07,0.301861204604894,0.674,0.44,0.009537314844138,"6","Hdgf"
"Chmp4b",5.74703925406135e-07,0.383138840360533,0.797,0.638,0.00969065759019825,"6","Chmp4b"
"Ripk2",5.88777951078026e-07,0.393104418236245,0.341,0.176,0.00992797381107768,"6","Ripk2"
"Itgb1",6.14908250459014e-07,0.489624989383002,0.804,0.72,0.0103685829192399,"6","Itgb1"
"Sdc3",6.2461915792036e-07,0.278074828025084,0.543,0.333,0.0105323282408531,"6","Sdc3"
"Atp6v1b2",6.30465394945262e-07,0.359455395692289,0.667,0.481,0.010630907489567,"6","Atp6v1b2"
"Ctdsp1",6.30581075977819e-07,0.382951493895323,0.42,0.235,0.010632858103138,"6","Ctdsp1"

"Crk1",6.55974740083649e-07,0.438677858322311,0.674,0.511,0.0110610460
672905,"6","Crk"
"Pnpla2",6.71749680322703e-07,0.391504193205122,0.464,0.278,0.01132704
31096014,"6","Pnpla2"
"Naip5",6.76612554508884e-07,0.293756353265209,0.297,0.143,0.011409040
8941288,"6","Naip5"
"Srsf11",6.82428954072588e-07,0.289282653516563,0.768,0.585,0.01150711
7023572,"6","Srsf11"
"Chp1",6.8970230284446e-07,0.261495071948449,0.326,0.16,0.011629760230
5633,"6","Chp1"
"Tubb62",6.94538832100197e-07,0.320682430626101,0.428,0.24,0.011711313
7868735,"6","Tubb6"
"Mdh21",7.08548652614713e-07,0.357092353347661,0.761,0.582,0.011947547
3803893,"6","Mdh2"
"Atp5f12",7.25771420537213e-07,0.291970301136888,0.899,0.811,0.0122379
576930985,"6","Atp5f1"
"Naip2",7.31552841185058e-07,0.33335091948255,0.464,0.282,0.0123354440
080624,"6","Naip2"
"Sub1",7.91805162704415e-07,0.396180747377584,0.899,0.786,0.0133514186
535218,"6","Sub1"
"Ube2a1",7.9251078605722e-07,0.317718785804583,0.594,0.405,0.013363316
8744968,"6","Ube2a"
"Ubtf",8.11107398296354e-07,0.3640189865182,0.58,0.374,0.0136768929500
731,"6","Ubtf"
"Fam46a2",8.25885920997831e-07,0.538195645463199,0.609,0.402,0.0139260
883998654,"6","Fam46a"
"Rnf115",8.42751462965092e-07,0.271902180212042,0.572,0.357,0.01421047
51685174,"6","Rnf115"
"Rchy1",8.45596530146082e-07,0.431078625884904,0.428,0.249,0.014258448
6913232,"6","Rchy1"
"Lrmp",8.55684801069727e-07,0.290488289578432,0.478,0.291,0.0144285571
156377,"6","Lrmp"
"Stxbp3",8.66391427994386e-07,0.430012701463733,0.478,0.294,0.01460909
22588413,"6","Stxbp3"
"Ddx41",8.81391096517257e-07,0.26706968922552,0.348,0.178,0.0148620166
69474,"6","Ddx41"
"Mapk1",8.97033997367005e-07,0.367915842964103,0.565,0.369,0.015125787
2636024,"6","Mapk1"
"Wdr3",9.04437640402837e-07,0.272105580104436,0.319,0.16,0.01525062749
24726,"6","Wdr3"
"Ctdsp2",9.18459169679336e-07,0.42185150606319,0.399,0.24,0.0154870585
19133,"6","Ctdsp2"
"Pik3cd1",9.27840929131693e-07,0.369696652302558,0.543,0.349,0.0156452
537470186,"6","Pik3cd"
"Psma11",9.44604569950476e-07,0.288813163505547,0.79,0.653,0.015927922
2585049,"6","Psma1"
"App1",9.69342906853209e-07,0.466392486739962,0.659,0.495,0.0163450600
953588,"6","App"
"Fes",9.7539695607704e-07,0.38116848675968,0.739,0.541,0.0164471434733
71,"6","Fes"

"Ube2j2",9.78495946759238e-07,0.352578410226376,0.543,0.342,0.0164993986542543,"6","Ube2j2"
"Phf3",9.8288206877909e-07,0.314978183506393,0.659,0.462,0.016573357443753,"6","Phf3"
"Arfgef1",9.84113619279696e-07,0.321793289440964,0.478,0.274,0.0165941238482942,"6","Arfgef1"
"Ifi351",9.91439986849429e-07,0.361513415847384,0.486,0.305,0.0167176610582551,"6","Ifi35"
"Sdhb1",1.01060057118286e-06,0.302937212161934,0.717,0.536,0.0170407468312853,"6","Sdhb"
"Casp3",1.01274989602582e-06,0.357744769733906,0.326,0.168,0.0170769887467874,"6","Casp3"
"Msl2",1.09469576409314e-06,0.279772843047489,0.391,0.215,0.0184587599741385,"6","Msl2"
"Rb1cc1",1.09762777104389e-06,0.495037085714959,0.572,0.38,0.0185081994753421,"6","Rb1cc1"
"Sin3b",1.16443335854538e-06,0.284916937404164,0.79,0.606,0.0196346752917922,"6","Sin3b"
"Tap2",1.32233399484781e-06,0.322375931820923,0.565,0.362,0.0222971958211238,"6","Tap2"
"Xrn2",1.35861637912373e-06,0.378819213823254,0.674,0.494,0.0229089893847843,"6","Xrn2"
"Gch11",1.39188749989048e-06,0.306321984137326,0.522,0.324,0.0234700070231533,"6","Gch1"
"Chuk",1.44822902282825e-06,0.292548878770461,0.399,0.218,0.02442003778293,"6","Chuk"
"Arl6ip51",1.58207783588936e-06,0.282341549025442,0.746,0.567,0.0266769964687664,"6","Arl6ip5"
"Rack12",1.64993668435076e-06,0.265637500828952,0.971,0.978,0.0278212323715225,"6","Rack1"
"Cep350",1.81507620148441e-06,0.441084116651354,0.507,0.322,0.0306058149094301,"6","Cep350"
"Rprd2",1.86103807351368e-06,0.322333431298786,0.319,0.16,0.0313808239955877,"6","Rprd2"
"Golga7",1.90212307660781e-06,0.289702367328026,0.493,0.304,0.032073599317761,"6","Golga7"
"Med8",1.99002696488583e-06,0.326009201557089,0.543,0.35,0.0335558346819049,"6","Med8"
"BC0055372",2.00730575875097e-06,0.349564148587782,0.783,0.612,0.0338471897040589,"6","BC005537"
"Dennd5a",2.15968603820403e-06,0.355434864210061,0.572,0.421,0.0364166259761964,"6","Dennd5a"
"Cisd1",2.3372086222823e-06,0.258569335536131,0.348,0.183,0.0394100117889241,"6","Cisd1"
"Mau2",2.35536818215038e-06,0.396899821510298,0.428,0.256,0.0397162182874197,"6","Mau2"
"Zdhhc9",2.36256054783646e-06,0.283292234435042,0.283,0.138,0.0398374959576184,"6","Zdhhc9"
"Ube2q1",2.37707490370789e-06,0.290463454923154,0.63,0.434,0.0400822370263224,"6","Ube2q1"

"Stk401",2.41238596170292e-06,0.454229407951902,0.5,0.314,0.0406776520
862346,"6","Stk40"
"Ppp2ca",2.45910278404457e-06,0.315684163342192,0.855,0.739,0.04146539
11445595,"6","Ppp2ca"
"Pgd",2.50893986193533e-06,0.425200104575078,0.688,0.519,0.04230574395
19535,"6","Pgd"
"Grb2",2.62433009859558e-06,0.275843366379221,0.877,0.739,0.0442514541
225187,"6","Grb2"
"Psma31",2.71374025277043e-06,0.320765320066619,0.804,0.688,0.04575908
8142215,"6","Psma3"
"Slc25a281",2.82176405975505e-06,0.411793297836221,0.348,0.194,0.04758
05855755896,"6","Slc25a28"
"Ubald21",2.8517561536552e-06,0.293294222004025,0.645,0.432,0.04808631
22629339,"6","Ubald2"
"Rnf146",2.881430772609e-06,0.304169658945191,0.319,0.166,0.0485866856
87733,"6","Rnf146"
"Mcmbp",2.88829355101523e-06,0.307193509613816,0.464,0.274,0.048702405
8572189,"6","Mcmbp"
"Adap1",2.90682927317076e-06,0.251064017175395,0.428,0.252,0.049014955
2042053,"6","Adap1"
"Lamtor5",2.93879444133948e-06,0.361383454711057,0.543,0.36,0.04955395
18698664,"6","Lamtor5"
"Tmod3",3.08706150257228e-06,0.355889836924463,0.616,0.435,0.052054031
0563737,"6","Tmod3"
"Otub1",3.17019160352166e-06,0.257839131450628,0.471,0.279,0.053455770
8185822,"6","Otub1"
"Prdx2",3.18135595067763e-06,0.284178171981804,0.804,0.624,0.053644024
0403262,"6","Prdx2"
"Atp5d1",3.18670378669789e-06,0.291750296422791,0.87,0.752,0.053734199
2512998,"6","Atp5d"
"March71",3.30928369196826e-06,0.45493975714806,0.703,0.526,0.05580114
16139689,"6","March7"
"Lbr2",3.39437783084027e-06,0.316916091461442,0.29,0.146,0.05723599898
36286,"6","Lbr"
"Stag2",3.47675541145626e-06,0.348566258813445,0.58,0.391,0.0586250497
479754,"6","Stag2"
"Tm6sf11",3.4896007405993e-06,0.344268503716454,0.797,0.639,0.05884164
76879854,"6","Tm6sf1"
"Chmp2b",3.59436341539899e-06,0.345938303535657,0.428,0.246,0.06060815
59104578,"6","Chmp2b"
"Trappc5",3.62111154594417e-06,0.252504644969376,0.275,0.132,0.0610591
828877105,"6","Trappc5"
"Cox6c",3.64165946052458e-06,0.412587709153379,0.761,0.605,0.061405661
8233654,"6","Cox6c"
"Plekhf2",3.77469127208593e-06,0.375729963584718,0.486,0.313,0.0636488
442299129,"6","Plekhf2"
"Cbfb1",3.79078704328041e-06,0.29929915152385,0.478,0.308,0.0639202511
237942,"6","Cbfb"
"Phka2",3.82104180866732e-06,0.269832960769904,0.29,0.141,0.0644304069
777484,"6","Phka2"

"Tia1",3.91596614441835e-06,0.286730015676354,0.319,0.164,0.0660310211271823,"6","Tia1"
"Akt11",4.16492636169094e-06,0.299370520406324,0.783,0.617,0.0702289883108326,"6","Akt1"
"Prpf40a",4.2550970144335e-06,0.347518004834172,0.775,0.629,0.0717494458573776,"6","Prpf40a"
"Kctd20",4.40790465539501e-06,0.375109984032342,0.362,0.208,0.0743260882992707,"6","Kctd20"
"Raly1",4.68631984835557e-06,0.310610025436424,0.768,0.617,0.0790207252829716,"6","Raly"
"Ggh",4.91097285279199e-06,0.303674538445576,0.616,0.432,0.0828088242437786,"6","Ggh"
"Slc20a1",4.95511336341455e-06,0.553599409347959,0.268,0.132,0.0835531215338961,"6","Slc20a1"
"H2-
M31",5.0080417251526e-06,0.265352218027764,0.478,0.287,0.0844455995695232,"6","H2-M3"
"Emd",5.04828441420496e-06,0.271374571211591,0.587,0.393,0.0851241717923241,"6","Emd"
"Wdr261",5.22930796304457e-06,0.457372208444562,0.819,0.699,0.0881765908728576,"6","Wdr26"
"Neat12",5.33089668765027e-06,0.268820685304551,0.986,0.748,0.0898895799471589,"6","Neat1"
"Ankrd13a",5.4415177804946e-06,0.328718773153033,0.594,0.4,0.0917548728146999,"6","Ankrd13a"
"Map7d1",5.52214549856521e-06,0.386214820542997,0.609,0.445,0.0931144173968066,"6","Map7d1"
"Crem1",5.73453717945207e-06,0.270950595103558,0.37,0.211,0.0966957659199208,"6","Crem"
"Irf11",5.85273782303975e-06,0.368489609895411,0.594,0.41,0.0986888651720963,"6","Irf1"
"Ccm2",5.98359576451346e-06,0.303120876513464,0.514,0.321,0.100895391781226,"6","Ccm2"
"Zfp207",6.21346400417538e-06,0.280095249623441,0.594,0.398,0.104771430038405,"6","Zfp207"
"Ccpg1",6.63717600517285e-06,0.278379635768366,0.297,0.15,0.111916061799225,"6","Ccpg1"
"Psma61",6.64829951716273e-06,0.256545717319682,0.652,0.472,0.112103626458398,"6","Psma6"
"Kansl3",6.6859849186001e-06,0.262900763097398,0.297,0.153,0.112739077697435,"6","Kansl3"
"Psmd131",6.70337784981271e-06,0.259482242434468,0.63,0.448,0.113032357303542,"6","Psmd13"
"Sec11a",6.70958382960257e-06,0.306632973127728,0.638,0.465,0.113137002534759,"6","Sec11a"
"Ebi31",6.77630762218684e-06,0.310480054509905,0.493,0.308,0.114262099125314,"6","Ebi3"
"Copb1",6.90947175445545e-06,0.347015070899706,0.667,0.488,0.116507512723628,"6","Copb1"
"Herc1",7.013680416099e-06,0.409154769949467,0.529,0.346,0.11826467917

6261,"6","Herc1"
"Asap1",7.40516537334312e-06,0.394977800149207,0.746,0.584,0.124865898
525312,"6","Asap1"
"Rnf166",7.40945911387903e-06,0.306028372192855,0.435,0.258,0.12493829
9578228,"6","Rnf166"
"Xiap",7.42653813688288e-06,0.36247997523297,0.594,0.425,0.12522628606
4119,"6","Xiap"
"Tnfaip22",7.63114151663852e-06,0.409787517113861,0.58,0.385,0.1286763
08253559,"6","Tnfaip2"
"Mff1",7.65183978812667e-06,0.276534180508883,0.558,0.394,0.1290253225
07392,"6","Mff"
"Eif4ebp2",7.70466845949701e-06,0.334289426120229,0.486,0.305,0.129916
119564039,"6","Eif4ebp2"
"Rap2a",7.70856470317296e-06,0.404626771803081,0.601,0.444,0.129981818
024902,"6","Rap2a"
"Ep3001",7.75639166170933e-06,0.32531584355064,0.623,0.449,0.130788276
199743,"6","Ep300"
"Slc11a1",7.7632081524848e-06,0.334329687310588,0.812,0.728,0.13090321
5867199,"6","Slc11a1"
"Fndc3a1",7.7855795061794e-06,0.406491386890568,0.478,0.302,0.13128044
1633197,"6","Fndc3a"
"Bach11",7.87380869173605e-06,0.348886395477937,0.746,0.568,0.13276816
2160053,"6","Bach1"
"Seh1l",7.8949966854055e-06,0.365659875187569,0.326,0.18,0.13312543410
9307,"6","Seh1l"
"Glyr1",7.94140058933517e-06,0.303862880995686,0.63,0.443,0.1339078967
3737,"6","Glyr1"
"Ubac2",7.95855237993047e-06,0.343029612962063,0.42,0.263,0.1341971102
30388,"6","Ubac2"
"Pigx",8.05984792163757e-06,0.310477975064453,0.478,0.294,0.1359051556
54653,"6","Pigx"
"Csk",8.09055875240816e-06,0.375807901956381,0.688,0.559,0.13642300168
3106,"6","Csk"
"Prr5l",8.10937539657311e-06,0.259876205873131,0.275,0.133,0.136740287
937016,"6","Prr5l"
"Ccdc12",8.19752115415372e-06,0.363481599631632,0.71,0.556,0.138226601
70134,"6","Ccdc12"
"Daxx",8.40024195165342e-06,0.279436491950278,0.297,0.149,0.1416448797
8878,"6","Daxx"
"Ogfrl1",8.45142781043736e-06,0.424708006083591,0.659,0.508,0.14250797
5739595,"6","Ogfrl1"
"Phf101",8.47902477523414e-06,0.254418684743974,0.406,0.238,0.14297331
5759998,"6","Phf10"
"Rhog",9.37123716516453e-06,0.376340015052279,0.775,0.645,0.1580178010
79004,"6","Rhog"
"Sharpin",1.05978330370189e-05,0.253661902331786,0.333,0.181,0.1787006
60670212,"6","Sharpin"
"Dctn3",1.06498065774677e-05,0.253091955998193,0.645,0.463,0.179577038
50926,"6","Dctn3"
"Cisd2",1.06609029344356e-05,0.291211577425354,0.696,0.517,0.179764145

280452,"6","Cisd2"
"Mrpl201",1.06990131755421e-05,0.259821388461882,0.536,0.352,0.180406760165991,"6","Mrpl20"
"Pou2f22",1.08003931842134e-05,0.559311639689891,0.841,0.766,0.182116229872206,"6","Pou2f2"
"Rps27a1",1.09476748280563e-05,0.315657932111022,1,0.956,0.184599692950686,"6","Rps27a"
"Lta4h",1.09497647924542e-05,0.3878469541968,0.348,0.193,0.184634933930363,"6","Lta4h"
"Tbc1d10b",1.13097469525305e-05,0.254708408453367,0.37,0.212,0.190704953113569,"6","Tbc1d10b"
"Vezf1",1.1524606932725e-05,0.331669770801338,0.399,0.236,0.194327922099609,"6","Vezf1"
"Dynll1",1.16637753840207e-05,0.301833105761022,0.804,0.632,0.196674580525357,"6","Dynll1"
"Magt1",1.17074659519186e-05,0.392022342866113,0.609,0.425,0.197411290881252,"6","Magt1"
"Mob3a",1.18832890588299e-05,0.258856712065079,0.326,0.171,0.200376020109989,"6","Mob3a"
"Ggta11",1.21606019419619e-05,0.274246769649365,0.37,0.213,0.205052069945361,"6","Ggta1"
"Stx4a",1.26934736691676e-05,0.292785683961077,0.514,0.331,0.214037353009504,"6","Stx4a"
"H2-
T221",1.30157239192519e-05,0.367302376536014,0.406,0.254,0.219471136726425,"6","H2-T22"
"Mbtd1",1.30892781337197e-05,0.283135847229535,0.514,0.326,0.220711407890782,"6","Mbtd1"
"Bin3",1.31308999426393e-05,0.347552431562608,0.362,0.21,0.221413234832783,"6","Bin3"
"Phactr21",1.32849543191609e-05,0.250890872497897,0.261,0.126,0.224010899729691,"6","Phactr2"
"Mrpl30",1.33165860641849e-05,0.314251349834699,0.522,0.35,0.224544274214286,"6","Mrpl30"
"Snap231",1.36164272279297e-05,0.327365127343886,0.565,0.398,0.22960019591735,"6","Snap23"
"Ptbp31",1.36848402500698e-05,0.333978288990664,0.826,0.694,0.230753776296677,"6","Ptbp3"
"Prkab2",1.40188613944901e-05,0.291555105163352,0.319,0.173,0.236386040833892,"6","Prkab2"
"Tcof1",1.45356405713803e-05,0.267632361984765,0.391,0.23,0.245099971314615,"6","Tcof1"
"Yy1",1.45378362399356e-05,0.307122086791888,0.754,0.575,0.245136994677793,"6","Yy1"
"Gpbp1",1.48417932240946e-05,0.282719589638579,0.609,0.45,0.250262317344683,"6","Gpbp1"
"Furin",1.49080396908752e-05,0.378407721871012,0.551,0.39,0.251379365267538,"6","Furin"
"Rnpep1",1.49124673350448e-05,0.339660839765069,0.652,0.464,0.251454024203525,"6","Rnpep"

"Sh3bp51",1.51355657102698e-05,0.282782881509544,0.362,0.204,0.2552159
0900657,"6","Sh3bp5"
"Dock11",1.56929720048885e-05,0.30178958905613,0.341,0.192,0.264614893
946429,"6","Dock11"
"Sys1",1.59678057058107e-05,0.298387573233649,0.652,0.488,0.2692491398
11381,"6","Sys1"
"Ccz11",1.63567347939783e-05,0.305446586827237,0.464,0.3,0.27580726209
6063,"6","Ccz1"
"Ssu72",1.66999234599623e-05,0.279061232587567,0.739,0.601,0.281594109
381884,"6","Ssu72"
"Clk3",1.70687760847998e-05,0.264376629689755,0.471,0.281,0.2878137023
41895,"6","Clk3"
"Nr1h2",1.76989985986595e-05,0.267546076499373,0.5,0.339,0.29844051437
0597,"6","Nr1h2"
"Psmd22",1.79432358470432e-05,0.251211812278737,0.609,0.447,0.30255884
2852843,"6","Psmd2"
"Gnb11",1.81048503635318e-05,0.348773303968559,0.855,0.747,0.305283986
829873,"6","Gnb1"
"Ggnbp21",1.89667414855161e-05,0.371696244943075,0.638,0.489,0.3198171
94928773,"6","Ggnbp2"
"Rbm42",1.90915159092798e-05,0.326244818551,0.594,0.415,0.321921141262
275,"6","Rbm42"
"Nol7",1.95652651048926e-05,0.271620481539526,0.674,0.511,0.3299095001
987,"6","Nol7"
"Cnppd1",1.99803791850935e-05,0.31037588875482,0.478,0.323,0.336909153
819047,"6","Cnppd1"
"Gm267401",2.07128558885129e-05,0.415831702662827,0.522,0.379,0.349260
175992104,"6","Gm26740"
"Cct71",2.08573286821501e-05,0.260891876512846,0.674,0.523,0.351696276
238415,"6","Cct7"
"Atf6",2.17708967876982e-05,0.331883935837635,0.457,0.282,0.3671008616
34167,"6","Atf6"
"BC005624",2.17814025551198e-05,0.325470516067585,0.486,0.315,0.367278
009884431,"6","BC005624"
"Zbtb7b",2.20497395136194e-05,0.299475262017549,0.254,0.131,0.37180270
7678651,"6","Zbtb7b"
"Prrc2c",2.30705443349632e-05,0.378784821424436,0.812,0.666,0.38901551
8576149,"6","Prrc2c"
"Zeb23",2.32016478238108e-05,0.392379024061521,0.906,0.785,0.391226185
605098,"6","Zeb2"
"Snrk",2.33723221039643e-05,0.331860907827858,0.399,0.249,0.3941040953
17046,"6","Snrk"
"Aplp2",2.47300671824948e-05,0.30045884958171,0.652,0.473,0.4169983928
31228,"6","Aplp2"
"Stk11",2.48725497631406e-05,0.286093764887345,0.457,0.287,0.419400934
106077,"6","Stk11"
"Gnai3",2.56358737360253e-05,0.285288849005586,0.536,0.349,0.432272102
936858,"6","Gnai3"
"Ccnt2",2.61186390893045e-05,0.289691039011386,0.377,0.212,0.440412492
323853,"6","Ccnt2"

"Sec61b",2.63287931268582e-05,0.4574724254403,0.688,0.565,0.4439561097
05083,"6","Sec61b"
"Tifab",2.65384044757432e-05,0.296077621632251,0.514,0.365,0.447490576
269982,"6","Tifab"
"Was",2.66497576769643e-05,0.310983167412028,0.551,0.395,0.44936821394
8972,"6","Was"
"Arhgap451",2.71104364801399e-05,0.319126164345732,0.79,0.607,0.457136
179928119,"6","Arhgap45"
"Tmem50b",2.85723296863774e-05,0.288866157978806,0.377,0.221,0.4817866
23171695,"6","Tmem50b"
"B2m",2.86269602514523e-05,0.263567986864269,0.993,0.972,0.48270780375
9988,"6","B2m"
"Cdc40",2.90752975961058e-05,0.332090131004189,0.457,0.305,0.490267668
065536,"6","Cdc40"
"Rala",2.97841963272474e-05,0.407832357978168,0.58,0.435,0.50222111847
0046,"6","Rala"
"Larp4b",3.01124762402878e-05,0.302592410610029,0.587,0.43,0.507756574
363734,"6","Larp4b"
"Oxr1",3.18125453276757e-05,0.313198259381599,0.384,0.229,0.5364231393
15268,"6","Oxr1"
"Sh3kbp1",3.25146910503464e-05,0.417544085199473,0.572,0.425,0.5482627
20490941,"6","Sh3kbp1"
"Epb41",3.51921026235896e-05,0.271769715571006,0.362,0.211,0.593409234
438967,"6","Epb41"
"Hacd41",3.53452955814093e-05,0.273986351471309,0.5,0.323,0.5959923740
93723,"6","Hacd4"
"Capza1",3.62178534775232e-05,0.408043114943852,0.638,0.499,0.61070544
5337995,"6","Capza1"
"C1d1",3.66925240796e-05,0.261193445088026,0.514,0.349,0.6187093410302
15,"6","C1d"
"S100a13",4.10553985369727e-05,0.307301963551684,0.399,0.255,0.6922761
30130434,"6","S100a13"
"Zc3h7a",4.20273483869114e-05,0.348314194712523,0.486,0.333,0.70866514
85001,"6","Zc3h7a"
"Phf8",4.28883000979904e-05,0.280045873886474,0.261,0.134,0.7231825162
52314,"6","Phf8"
"Ubr4",4.39720060719105e-05,0.256837347511179,0.399,0.235,0.7414559663
84555,"6","Ubr4"
"Bnip3l",4.62131938633952e-05,0.263665175705169,0.717,0.617,0.77924687
4924569,"6","Bnip3l"
"Sema4d1",4.64399642847774e-05,0.374869277758894,0.674,0.532,0.7830706
77769917,"6","Sema4d"
"Pxk",4.70603532548953e-05,0.299280709588819,0.428,0.267,0.79353167658
4045,"6","Pxk"
"Pnisr1",4.74974191662537e-05,0.266335414386596,0.725,0.575,0.80090148
1981369,"6","Pnisr"
"Fam168a",4.95317528393202e-05,0.250186616032488,0.464,0.283,0.8352044
16376618,"6","Fam168a"
"Cers5",4.95566679424235e-05,0.25741143778827,0.341,0.2,0.835624534845
146,"6","Cers5"

"Lman2l",5.04190363358097e-05,0.263870475151614,0.326,0.186,0.85016579
0694422,"6","Lman2l"
"Bbx1",5.12398210732861e-05,0.325424639066803,0.406,0.255,0.8640058629
3775,"6","Bbx"
"Srsf12",5.61507204369787e-05,0.261744169795926,0.457,0.297,0.94681344
8008334,"6","Srsf1"
"Kmt5a1",5.62991158772043e-05,0.358957496599819,0.413,0.268,0.94931569
1921418,"6","Kmt5a"
"Rbm8a",5.64686462071428e-05,0.297706485927001,0.558,0.388,0.952174312
344842,"6","Rbm8a"
"Trim12c",5.74964924899399e-05,0.317117198847062,0.312,0.179,0.9695058
56365367,"6","Trim12c"
"Wdr37",5.88164395341925e-05,0.32255232407312,0.326,0.189,0.9917628034
25555,"6","Wdr37"
"Tcp11l21",5.91347082377277e-05,0.265624967820922,0.297,0.164,0.997129
450304564,"6","Tcp11l2"
"Fam111a1",5.98602040709109e-05,0.405259377376976,0.5,0.339,1,"6","Fam
111a"
"Hnrnpd1",6.00375192360378e-05,0.29510885427488,0.783,0.638,1,"6","Hnr
npd"
"Eif61",6.08416479926578e-05,0.317264582712554,0.688,0.519,1,"6","Eif6
"
"Ogfr",6.26384333160384e-05,0.312959335109268,0.536,0.391,1,"6","Ogfr"
"Map3k1",6.57250719152572e-05,0.303841627365079,0.464,0.31,1,"6","Map3
k1"
"Nfatc1",6.68139076683371e-05,0.287187650639226,0.413,0.258,1,"6","Nfa
tc1"
"Vcpip1",6.7278466681659e-05,0.346887970883884,0.399,0.245,1,"6","Vcpi
p1"
"Pik3r5",6.73771041976289e-05,0.267525041262858,0.362,0.214,1,"6","Pik
3r5"
"Sbno11",7.00960949283381e-05,0.385635694753258,0.594,0.454,1,"6","Sbn
o1"
"Atg101",7.32246170146815e-05,0.275953948231774,0.435,0.279,1,"6","Atg
101"
"Tbrg1",7.36848457842507e-05,0.291848010055554,0.268,0.144,1,"6","Tbrg
1"
"Nsa21",7.53284455916089e-05,0.276595918901755,0.833,0.704,1,"6","Nsa2
"
"Dusp31",7.54979703068908e-05,0.343381070136873,0.623,0.456,1,"6","Dus
p3"
"Tbc1d23",7.95280477646977e-05,0.329257359719475,0.413,0.275,1,"6","Tb
c1d23"
"Adipor2",8.07267195710653e-05,0.350192645517544,0.478,0.34,1,"6","Adi
por2"
"Polr1d1",8.1081191669104e-05,0.253142487212879,0.797,0.679,1,"6","Pol
r1d"
"Prpf38b1",8.36626500587734e-05,0.335048079890875,0.652,0.512,1,"6","P
rpf38b"
"Tmed21",8.54720121590474e-05,0.256223167069455,0.855,0.729,1,"6","Tme

d2"
"Traf3ip3",9.86676116443494e-05,0.282347346672295,0.514,0.349,1,"6","T
raf3ip3"
"Trim30a1",9.93692565244376e-05,0.319844879309106,0.587,0.426,1,"6","T
rim30a"
"Cd82",9.9680478592312e-05,0.38728718107718,0.587,0.431,1,"6","Cd82"
"Csde1",0.000100418736873367,0.257594577542094,0.797,0.673,1,"6","Csde
1"
"Hnrnpul2",0.000104185894719176,0.292187354268694,0.536,0.375,1,"6","H
nrnpul2"
"Clec4a21",0.000110639725985929,0.358645520739208,0.486,0.324,1,"6","C
lec4a2"
"Ensa",0.000111307193905984,0.280772240658939,0.572,0.413,1,"6","Ensa"
"Mpp61",0.000113405317622598,0.261167909764499,0.464,0.3,1,"6","Mpp6"
"Mbnl2",0.000131694727645105,0.33274415402539,0.696,0.512,1,"6","Mbnl2
"
"Copz1",0.00013283505857088,0.325259228354009,0.464,0.307,1,"6","Copz1
"
"Atp6ap1",0.000135002331149653,0.269929732298059,0.819,0.688,1,"6","At
p6ap1"
"Pgap2",0.000135545366401493,0.259530807203728,0.377,0.227,1,"6","Pgap
2"
"Smg1",0.000143480032478973,0.256032793425552,0.522,0.364,1,"6","Smg1"
"Rbm51",0.000144890355547836,0.336383424074141,0.688,0.533,1,"6","Rbm5
"
"Snx1",0.000144919714571257,0.258565315042956,0.529,0.362,1,"6","Snx1"
"Trip12",0.000147527071742044,0.319806874265525,0.58,0.423,1,"6","Trip
12"
"Lamtor2",0.00014963448491589,0.310256505252825,0.536,0.39,1,"6","Lamt
or2"
"Cln8",0.000150300502046588,0.346034032925288,0.442,0.298,1,"6","Cln8"
"Ssr31",0.000158842310347315,0.330219903158673,0.572,0.432,1,"6","Ssr3
"
"Ndufb7",0.000160124194728479,0.340935292703377,0.536,0.418,1,"6","Ndu
fb7"
"Adipor1",0.000162012395420389,0.398944358444432,0.688,0.558,1,"6","Ad
ipor1"
"Slc16a10",0.000164539032006905,0.257817940963264,0.319,0.186,1,"6","S
lc16a10"
"Chd8",0.000177179143161457,0.269298170806458,0.428,0.283,1,"6","Chd8"
"Pet100",0.000182781896475989,0.369043625624847,0.355,0.233,1,"6","Pet
100"
"Vps13c",0.000183777723435038,0.265613807188636,0.362,0.222,1,"6","Vps
13c"
"Elf4",0.000193197008163323,0.270653777351745,0.391,0.25,1,"6","Elf4"
"Secisbp2",0.000199909052319734,0.267827792771706,0.399,0.258,1,"6","S
ecisbp2"
"Vps37b1",0.00020096100525074,0.44829913780828,0.558,0.428,1,"6","Vps3
7b"
"Srrm11",0.000201269674048605,0.252519797779806,0.804,0.596,1,"6","Srr

m1"
"1810037I17Rik1",0.000201398145945045,0.296006556949182,0.522,0.399,1,
"6","1810037I17Rik"
"Usp15",0.000202118705423994,0.301711101568915,0.486,0.336,1,"6","Usp1
5"
"Ppil2",0.000212580054548601,0.271159146274484,0.464,0.32,1,"6","Ppil2
"
"Crybg3",0.000233999145026333,0.266593528801294,0.312,0.181,1,"6","Cry
bg3"
"Ppp4c1",0.000236021640984969,0.328890162687962,0.63,0.491,1,"6","Ppp4
c"
"Apaf11",0.000240258610317258,0.300201993844842,0.261,0.144,1,"6","Apa
f1"
"Xpo6",0.000258765604644979,0.379484518148352,0.37,0.233,1,"6","Xpo6"
"Sort1",0.000268084658867804,0.292342409508085,0.326,0.199,1,"6","Sort
1"
"Itpkb2",0.000276477778503988,0.351493183726491,0.536,0.385,1,"6","Itp
kb"
"Pfkfb4",0.000278873742019404,0.252767358154971,0.297,0.178,1,"6","Pfk
fb4"
"Rab10",0.000283688452577308,0.346796406617524,0.645,0.506,1,"6","Rab1
0"
"Arnt",0.000288020992907265,0.305465195831041,0.326,0.196,1,"6","Arnt"
"Tor1aip11",0.000293222608965405,0.280842285595172,0.79,0.664,1,"6","T
or1aip1"
"Tbc1d8",0.000304770280697901,0.304096028225943,0.362,0.234,1,"6","Tbc
1d8"
"Sash3",0.000306620390859234,0.290126539853611,0.391,0.236,1,"6","Sash
3"
"Birc31",0.000312475000314924,0.374582886293873,0.652,0.518,1,"6","Bir
c3"
"Cnot6l",0.00031480833896123,0.323166861404537,0.543,0.402,1,"6","Cnot
6l"
"Thrap3",0.000318700760736008,0.283144979846667,0.717,0.566,1,"6","Thr
ap3"
"Atxn7l3b1",0.000332546847928095,0.3341937564107,0.558,0.411,1,"6","At
xn7l3b"
"Sqor",0.000338672195490787,0.314959089181984,0.507,0.374,1,"6","Sqor"
"Isy11",0.000341876169985386,0.375140400998189,0.304,0.177,1,"6","Isy1
"
"0610030E20Rik",0.000344213240473322,0.285701249660735,0.348,0.218,1,"
6","0610030E20Rik"
"Ttc19",0.000356815597104223,0.298279188971764,0.319,0.192,1,"6","Ttc1
9"
"N4bp11",0.0003587643918063,0.331292428431411,0.645,0.492,1,"6","N4bp1
"
"R3hdm1",0.000373199245288758,0.407257065191935,0.391,0.256,1,"6","R3h
dm1"
"Safb",0.000381053117133702,0.306715773696885,0.464,0.328,1,"6","Safb"
"Supt4a1",0.00038694662838346,0.25595076235101,0.572,0.436,1,"6","Supt

4a"
"Smad3",0.000388038169194922,0.255218484273836,0.275,0.163,1,"6","Smad
3"
"Ubr2",0.000392385537159568,0.266984719805856,0.391,0.263,1,"6","Ubr2"
"Dtx3l",0.000396139001745083,0.346690230664843,0.254,0.141,1,"6","Dtx3
l"
"Ndufa4",0.000398391046267873,0.304214079973767,0.645,0.563,1,"6","Ndu
fa4"
"Zfp2631",0.000437294893845806,0.282581330724506,0.304,0.191,1,"6","Zf
p263"
"Ddx3y1",0.00043970863055194,0.506464599031621,0.507,0.347,1,"6","Ddx3
y"
"Hnrnph2",0.000444175274909061,0.261741175908734,0.572,0.426,1,"6","Hn
rnph2"
"Rnf114",0.000454901964880573,0.26137990171741,0.406,0.272,1,"6","Rnf1
14"
"Slc8b1",0.000455126969919195,0.280161577834928,0.341,0.22,1,"6","Slc8
b1"
"Dock102",0.000500137349780034,0.280384005664686,0.761,0.631,1,"6","Do
ck10"
"Ptafr1",0.000518200820847699,0.412533903449967,0.558,0.449,1,"6","Pta
fr"
"Hcst",0.000523618438047752,0.383031445035133,0.341,0.223,1,"6","Hcst"
"Ogdh",0.000531629332679868,0.295619878738251,0.413,0.29,1,"6","Ogdh"
"Nr2c2",0.000565065584346095,0.287613797042271,0.312,0.19,1,"6","Nr2c2
"
"Tma7",0.000571716967419227,0.262451758098261,0.71,0.596,1,"6","Tma7"
"Psma51",0.00057914898557207,0.302008801458532,0.696,0.559,1,"6","Psma
5"
"Tbl1xr1",0.00057943492794249,0.275756669390013,0.449,0.313,1,"6","Tbl
1xr1"
"Rsu1",0.000597870493092936,0.253418452225427,0.717,0.603,1,"6","Rsu1"
"Coro1b",0.000611639775099288,0.281211686380335,0.659,0.555,1,"6","Cor
o1b"
"Pkn1",0.000613596953803918,0.253945023328927,0.696,0.563,1,"6","Pkn1"
"Hps3",0.000627499646431914,0.257878568954605,0.37,0.244,1,"6","Hps3"
"Dbi2",0.000640535605928932,0.254494113582599,0.594,0.462,1,"6","Dbi"
"Zdhhc3",0.000651940475658993,0.276588925143441,0.333,0.212,1,"6","Zdh
hc3"
"Wac1",0.000678099161491164,0.255529736426273,0.58,0.433,1,"6","Wac"
"Psme4",0.000749005767239853,0.266757276759316,0.377,0.249,1,"6","Psme
4"
"Tnrc18",0.000757384933143512,0.257215286450641,0.486,0.327,1,"6","Tnr
c18"
"Zcchc2",0.000845324194472391,0.27021745595956,0.261,0.149,1,"6","Zcch
c2"
"Ndufa61",0.000872433650664117,0.342336418415518,0.63,0.522,1,"6","Ndu
fa6"
"Cdc5l",0.000909076307277931,0.260643056499592,0.572,0.433,1,"6","Cdc5
l"

"Rbm6",0.00093405285717102,0.394886705105317,0.435,0.309,1,"6","Rbm6"
"Abi1",0.000947352018101143,0.265012984532444,0.594,0.484,1,"6","Abi1"
"H2-Q71",0.000951531358622724,0.468839380954824,0.514,0.402,1,"6","H2-Q7"
"Zmiz11",0.000985126869737405,0.250028413823487,0.681,0.557,1,"6","Zmiz1"
"2810474O19Rik1",0.00100526581723222,0.366178801717613,0.565,0.445,1,"6","2810474O19Rik"
"Cdc42se1",0.00104344337528951,0.2789206380261,0.601,0.469,1,"6","Cdc42se1"
"Nucb2",0.00106561164048266,0.314435004227911,0.348,0.23,1,"6","Nucb2"
"D8Ertd738e",0.00108886069069556,0.34951700605553,0.645,0.565,1,"6","D8Ertd738e"
"Mob1b",0.00117380973113906,0.262650676484921,0.319,0.197,1,"6","Mob1b"
"Arpp19",0.00117453885151994,0.253023577942305,0.558,0.443,1,"6","Arpp19"
"Snrnp200",0.00127767982314366,0.266724429958676,0.319,0.211,1,"6","Snrnp200"
"Usmg5",0.00133196220182453,0.334428419782623,0.464,0.362,1,"6","Usmg5"
"Tmem123",0.00145797656374209,0.254441175108205,0.406,0.283,1,"6","Tmem123"
"Pik3r6",0.00151467404077306,0.263878035163653,0.254,0.15,1,"6","Pik3r6"
"Trim12a",0.00187418643816654,0.275324806415611,0.355,0.24,1,"6","Trim12a"
"Bcl2l11",0.00187983920931987,0.279864678556662,0.37,0.248,1,"6","Bcl2l11"
"Usp7",0.00192663744139323,0.264997486585019,0.529,0.399,1,"6","Usp7"
"Ddx17",0.00202752800519659,0.261093801121141,0.601,0.493,1,"6","Ddx17"
"Slc6a61",0.00203678149645623,0.302821552074145,0.732,0.668,1,"6","Slc6a6"
"Vrk11",0.00208262002672123,0.254513726759272,0.478,0.36,1,"6","Vrk1"
"Isg151",0.00217396348147734,0.644763365062417,0.29,0.186,1,"6","Isg15"
"Ube3a",0.00253258159725099,0.252946353836555,0.572,0.424,1,"6","Ube3a"
"Tra2a2",0.00264803703241355,0.273790885094726,0.725,0.597,1,"6","Tra2a"
"Cox5b",0.00279897151831727,0.282585450396718,0.616,0.535,1,"6","Cox5b"
"Safb21",0.00283989297427912,0.268928024220193,0.565,0.473,1,"6","Safb2"
"Mob2",0.00308309615583755,0.290878624125321,0.268,0.171,1,"6","Mob2"
"Atp5e",0.00349460187985651,0.277813045176597,0.754,0.665,1,"6","Atp5e"
"Kpna1",0.00428525458762324,0.254222899301033,0.319,0.222,1,"6","Kpna1"

"Atp13a3",0.00433217014713173,0.253484231939356,0.493,0.379,1,"6","Atp
13a3"
"Pnpla8",0.00449885086084939,0.342636660175345,0.457,0.343,1,"6","Pnpl
a8"
"Pbxip11",0.00498324589682823,0.375394279878067,0.616,0.535,1,"6","Pbx
ip1"
"Fgl21",0.00512107802290715,0.508964061528623,0.304,0.205,1,"6","Fgl2"
"Map1lc3b",0.00525453847903535,0.2560352535162,0.768,0.683,1,"6","Map1
lc3b"
"Elob1",0.00661852145030704,0.281407219337361,0.652,0.573,1,"6","Elob"
"Eif3d1",0.00791486800369197,0.282668122526904,0.5,0.425,1,"6","Eif3d"
"Fcho21",0.00862160971202536,0.258478781796112,0.623,0.507,1,"6","Fcho
2"
"Myo18a",0.00907349581950289,0.306103882545955,0.333,0.238,1,"6","Myo1
8a"
"Hexb2",1.9454361642108e-18,0.806920511200872,1,0.955,3.28039446009225
e-14,"7","Hexb"
"Lpcat22",1.06969234992904e-13,0.852557053181005,0.946,0.859,1.8037152
4045034e-09,"7","Lpcat2"
"Trem21",2.19519766974159e-11,0.630216666364088,0.968,0.934,3.70154231
071827e-07,"7","Trem2"
"Selplg3",5.85233893701077e-11,0.860654901465054,0.946,0.852,9.8682139
1558755e-07,"7","Selplg"
"Itgb53",2.06192163007323e-10,0.771221154369599,0.946,0.866,3.47681225
262947e-06,"7","Itgb5"
"Gpr342",3.13167248560372e-10,0.829960509589987,0.871,0.819,5.28062614
522499e-06,"7","Gpr34"
"C1qa1",3.97919934169135e-10,0.487845799663938,0.989,0.946,6.709725929
95995e-06,"7","C1qa"
"Vsir2",2.20551843488579e-09,0.922363232890568,0.806,0.825,3.718945184
90442e-05,"7","Vsir"
"Lgmn",2.92043750349456e-09,0.552426036306438,1,0.959,4.92444171839253
e-05,"7","Lgmn"
"Serpine23",5.14251851913568e-09,0.692949035647684,0.828,0.74,8.671314
72696658e-05,"7","Serpine2"
"Cd9",1.13611410516658e-08,0.596709455345506,0.978,0.891,0.00019157156
0413189,"7","Cd9"
"P2ry123",1.32873755334089e-08,0.703797112141074,0.882,0.804,0.0002240
51726244341,"7","P2ry12"
"Cst32",1.60040041134338e-08,0.754465878825016,0.989,0.993,0.000269859
517360721,"7","Cst3"
"Cd813",2.58828909169618e-08,0.487602941835462,0.978,0.885,0.000436437
30664181,"7","Cd81"
"Ctsl",3.75796349330312e-08,0.684568582794135,0.989,0.938,0.0006336678
04240773,"7","Ctsl"
"Laptm5",5.19093850961473e-08,0.435817420112656,0.957,0.954,0.00087529
6051491236,"7","Laptm5"
"Csf1r3",5.84269085835383e-08,0.483875551736972,0.946,0.946,0.00098519
4532535623,"7","Csf1r"
"Ctss2",7.53805349510338e-08,0.486415433110405,1,0.983,0.0012710665803

4433,"7","Ctss"
"Ifngr11",1.45931337777844e-07,0.686307151156546,0.86,0.853,0.00246069
421761001,"7","Ifngr1"
"Tmem1193",1.67472816588386e-07,0.713432469097357,0.806,0.715,0.002823
92663331337,"7","Tmem119"
"Ly862",1.70866973501429e-07,0.588883526160997,0.978,0.954,0.002881158
90718109,"7","Ly86"
"Unc93b11",5.19692516508697e-07,0.528807703453659,0.925,0.918,0.008763
05521336966,"7","Unc93b1"
"Ctsd1",5.45144768713138e-07,0.507322075499333,0.989,0.971,0.009192231
09004093,"7","Ctsd"
"Cfh",8.15242472931403e-07,0.667307919201555,0.817,0.787,0.01374661857
85693,"7","Cfh"
"Mpeg1",9.46465388839354e-07,0.580742208237377,0.871,0.866,0.015959299
3866092,"7","Mpeg1"
"Plxdc23",1.56232077310288e-06,0.734306044920695,0.806,0.776,0.0263438
528760607,"7","Plxdc2"
"Son1",1.94633503455245e-06,0.79165420347721,0.785,0.785,0.03281910135
26235,"7","Son"
"Sirpa1",2.38885556179271e-06,0.734690169275987,0.892,0.859,0.04028088
24829486,"7","Sirpa"
"Malat12",2.57420029111961e-06,0.928114431810174,0.774,0.832,0.0434061
653088588,"7","Malat1"
"Ctsz1",2.65513233876041e-06,0.515326408378755,0.989,0.974,0.044770841
4961781,"7","Ctsz"
"Pld4",2.8532297997757e-06,0.57902173873778,0.957,0.905,0.048111160883
8179,"7","Pld4"
"Tgfbr13",3.20554178529307e-06,0.818305867479424,0.763,0.752,0.0540518
455836118,"7","Tgfbr1"
"Slc16a62",4.93656184444876e-06,0.803277806003231,0.645,0.574,0.083240
305821095,"7","Slc16a6"
"C1qc1",7.14653017039255e-06,0.370059616567949,1,0.945,0.1205047917331
59,"7","C1qc"
"Sparc3",7.50120004875349e-06,0.470497187635276,0.957,0.836,0.12648523
5222081,"7","Sparc"
"C1qb1",8.30862087155997e-06,0.328689173115013,0.978,0.947,0.140099965
136244,"7","C1qb"
"Sgk12",1.01386276546417e-05,1.08944313620867,0.677,0.554,0.1709575395
12568,"7","Sgk1"
"Mef2c2",1.26617872735028e-05,0.627820567128957,0.796,0.818,0.21350305
7005804,"7","Mef2c"
"P2ry132",1.5687179772739e-05,0.978584179832723,0.656,0.647,0.26451722
5327925,"7","P2ry13"
"Mafb2",1.85560176307783e-05,0.706169203161637,0.806,0.796,0.312891569
290184,"7","Mafb"
"Olfml33",2.53709329201147e-05,0.513152753856375,0.839,0.814,0.4278046
70898974,"7","Olfml3"
"Rrbp13",2.84533033981967e-05,0.593160177153081,0.817,0.859,0.47977960
1900393,"7","Rrbp1"
"Pla2g151",3.71181682550102e-05,0.814638850460939,0.624,0.601,0.625886

553115982,"7","Pla2g15"
"Gm170561",3.95852563446031e-05,0.255808620217554,0.183,0.46,0.667486592482697,"7","Gm17056"
"Cd53",6.00739727011043e-05,0.437308032085175,0.839,0.9,1,"7","Cd53"
"Lair13",6.74448083626014e-05,0.593087415427423,0.785,0.817,1,"7","Lair1"
"Atp6v0b",8.74198189022405e-05,0.428858037037255,0.86,0.895,1,"7","Atp6v0b"
"Ctsf2",9.35905297590841e-05,0.675071459538132,0.688,0.666,1,"7","Ctsf"
"Itm2c",0.000164603005974177,0.461650601804068,0.882,0.874,1,"7","Itm2c"
"Ccr52",0.000183506090723417,0.882156963914622,0.667,0.624,1,"7","Ccr5"
"Fcgr31",0.000297342217439499,0.314355236794707,0.903,0.929,1,"7","Fcgr3"
"Apoe1",0.000312964679163483,0.4270409416642,1,0.99,1,"7","Apoe"
"Cx3cr13",0.00035387586139351,0.685586403014862,0.882,0.857,1,"7","Cx3cr1"
"Itgam3",0.000856150077148075,0.507896001145983,0.774,0.81,1,"7","Itgam"
"Slco2b11",0.000948358893516396,0.641937462185792,0.71,0.728,1,"7","Slco2b1"
"Rbm391",0.0010354236517945,0.745352485980379,0.785,0.808,1,"7","Rbm39"
"Adap2os2",0.00107760833204135,0.749769085710661,0.419,0.331,1,"7","Adap2os"
"Hpgds1",0.00130089408882425,0.585666896356576,0.731,0.778,1,"7","Hpgds"
"Fcgr11",0.00149826952526205,0.537889064651802,0.785,0.827,1,"7","Fcgr1"
"Rabgef12",0.00177547649752213,0.25246446613587,0.204,0.43,1,"7","Rabgef1"
"Etv31",0.00182493835026969,0.370197785691868,0.183,0.375,1,"7","Etv3"
"Tmem86a",0.00194435248972197,0.662557517498329,0.613,0.639,1,"7","Tmem86a"
"Abhd12",0.00215285073772439,0.381093894671949,0.86,0.891,1,"7","Abhd12"
"Serinc32",0.00224143181665007,0.269385564945217,0.935,0.934,1,"7","Serinc3"
"Dusp81",0.00286044151035938,0.25196269489043,0.183,0.384,1,"7","Dusp8"
"Tmbim6",0.00292186463120773,0.379989398436043,0.882,0.915,1,"7","Tmbim6"
"Qk3",0.00322503272979584,0.368027488696519,0.785,0.843,1,"7","Qk"
"Bhlhe41",0.00324847746220567,0.814003311903476,0.441,0.382,1,"7","Bhlhe41"
"Sec62",0.00333842077665591,0.696981644663691,0.559,0.611,1,"7","Sec62"
"Man2b11",0.00365608867034164,0.473865978595533,0.871,0.909,1,"7","Man

2b1"
"Pros1",0.00370852174175871,0.62943941301857,0.559,0.549,1,"7","Pros1"
"Mertk2",0.00392950106082475,0.809508780504963,0.581,0.603,1,"7","Mert
k"
"Slc29a32",0.00403176508883448,0.517590083506233,0.624,0.651,1,"7","Sl
c29a3"
"Lag33",0.00417420855156941,0.664855798931735,0.699,0.707,1,"7","Lag3"
"Tmem59",0.00436027191267114,0.477346748500766,0.796,0.841,1,"7","Tmem
59"
"Pou2f23",0.00504959191149078,0.64296397134506,0.731,0.773,1,"7","Pou2
f2"
"Sp1102",0.00535516478864876,0.303574679752644,0.161,0.33,1,"7","Sp110
"
"Asah11",0.00574615003975792,0.493612646211236,0.753,0.859,1,"7","Asah
1"
"Tgfbr22",0.00743562552260503,0.547538719310643,0.624,0.67,1,"7","Tgfb
r2"
"Golm13",0.00879613819974234,0.427722921937474,0.645,0.675,1,"7","Golm
1"
"Serpine1",5.25715955590059e-22,1.86105583668732,0.346,0.062,8.8646224
4315957e-18,"8","Serpine1"
"Csf11",2.62749941941826e-20,1.50607867795025,0.605,0.198,4.4304895210
2307e-16,"8","Csf1"
"Vat1",6.13373696310678e-18,0.430374289298349,0.469,0.125,1.0342707267
1907e-13,"8","Vat1"
"Ctsd2",1.09109366390745e-17,0.849583667936206,1,0.971,1.8398021360807
5e-13,"8","Ctsd"
"AI504432",2.71412036664474e-17,0.667203312519147,0.407,0.105,4.576549
76223636e-13,"8","AI504432"
"Cpd1",4.29079078298028e-17,0.846326223483675,0.827,0.427,7.2351314182
6134e-13,"8","Cpd"
"Ctsz2",1.42918073937978e-15,0.622537117883891,1,0.973,2.4098845627421
8e-11,"8","Ctsz"
"Gas2l31",2.87447625374552e-15,0.393325187783405,0.321,0.073,4.8469418
590657e-11,"8","Gas2l3"
"Cxcl162",6.30697048291927e-15,0.765879663330421,0.889,0.532,1.0634813
6282985e-10,"8","Cxcl16"
"Ctsb1",1.80174314107272e-14,0.72196624493542,1,0.974,3.03809928447683
e-10,"8","Ctsb"
"Gla",2.3641345173153e-14,0.585590130013578,0.556,0.197,3.986403623097
06e-10,"8","Gla"
"Id22",3.99739597385607e-14,1.0749388457617,0.901,0.516,6.740409091116
11e-10,"8","Id2"
"Fabp5",6.86659422268954e-14,0.625671882836072,0.593,0.241,1.157845117
82991e-09,"8","Fabp5"
"Cd631",1.33183224855959e-13,0.718531821970904,1,0.882,2.2457355375211
8e-09,"8","Cd63"
"Lilrb4a2",1.53797847948879e-13,0.833576466440818,0.531,0.199,2.593339
31211399e-09,"8","Lilrb4a"
"Pld3",1.64225482043538e-13,0.682604798849896,0.877,0.512,2.7691700782

1814e-09,"8","Pld3"
"Maff2",2.24757472862138e-13,0.611815944203107,0.901,0.491,3.789860507
40138e-09,"8","Maff"
"Cd91",3.81335656129553e-13,0.772960346935132,1,0.891,6.43008183365652
e-09,"8","Cd9"
"Slc7a11",8.24493063596678e-13,1.74691720148288,0.42,0.142,1.390260203
83672e-08,"8","Slc7a11"
"Cst72",1.1199575625753e-12,0.591493351471197,0.556,0.22,1.88847244201
447e-08,"8","Cst7"
"Fam46c",1.85665119031952e-12,0.757761547715853,0.716,0.354,3.13068523
711678e-08,"8","Fam46c"
"Apbb2",2.72958408799937e-12,0.604319797170604,0.506,0.201,4.602624689
18453e-08,"8","Apbb2"
"Tlr2",6.39269995556774e-12,1.02916427192956,0.901,0.701,1.07793706650
783e-07,"8","Tlr2"
"Rab7b1",7.07160149656804e-12,0.672321788616428,0.494,0.188,1.19241344
43513e-07,"8","Rab7b"
"Ccdc86",1.07661659286649e-11,0.500769360087658,0.667,0.314,1.81539089
889148e-07,"8","Ccdc86"
"Pmp221",1.66081885626146e-11,0.909521185400807,0.84,0.552,2.800472755
42807e-07,"8","Pmp22"
"Ctsa",2.06114521952345e-11,0.648949393559331,0.988,0.911,3.4755030691
6044e-07,"8","Ctsa"
"Grn1",5.75342628189488e-11,0.484612874859139,1,0.939,9.70142739653114
e-07,"8","Grn"
"Nfkbia2",1.13547059997095e-10,0.648858249358845,1,0.853,1.91463052567
102e-06,"8","Nfkbia"
"Ccl42",1.18509563404363e-10,0.912490758840106,0.975,0.786,1.998308258
12437e-06,"8","Ccl4"
"Tnf2",1.42865840004731e-10,0.938768910981947,0.938,0.699,2.4090037941
5978e-06,"8","Tnf"
"Scd2",1.63121462063724e-10,0.415677135777447,0.42,0.153,2.75055409331
851e-06,"8","Scd2"
"Il1a3",2.1288962977157e-10,0.765696904165096,1,0.753,3.58974493720821
e-06,"8","Il1a"
"Axl1",3.04570721919441e-10,0.482539336919507,0.741,0.395,5.1356715130
0561e-06,"8","Axl"
"Cd142",3.68746141044752e-10,0.990707520917765,0.951,0.836,6.217797430
2966e-06,"8","Cd14"
"Ccl33",4.1296964382853e-10,0.864746197478828,0.975,0.787,6.9634941342
3667e-06,"8","Ccl3"
"Timp21",4.46144445751105e-10,0.722501229264985,0.951,0.82,7.522887644
25513e-06,"8","Timp2"
"Fnip2",6.00150479257428e-10,0.375740268041683,0.358,0.12,1.0119737381
2388e-05,"8","Fnip2"
"Cd832",7.73931338044862e-10,0.625361518642554,0.988,0.832,1.305003022
21125e-05,"8","Cd83"
"Slc3a21",1.08816471523134e-09,0.536284647250659,0.988,0.791,1.8348633
4282308e-05,"8","Slc3a2"
"Fem1b",1.73327885458464e-09,0.631080657802062,0.481,0.215,2.922654804

60062e-05,"8","Fem1b"
"Cd68",2.26045051666127e-09,0.54076371073837,1,0.899,3.81157166119423e-05,"8","Cd68"
"Cstb",2.28196310608353e-09,0.577020114247672,0.765,0.47,3.84784618947806e-05,"8","Cstb"
"Arl5c2",2.30116884811725e-09,0.912141464095897,0.815,0.543,3.8802309116953e-05,"8","Arl5c"
"Atp6v1a1",3.59823052100533e-09,0.492617600963159,0.728,0.411,6.06733630451918e-05,"8","Atp6v1a"
"Gadd45b2",3.69272650143151e-09,0.797873585170537,0.938,0.668,6.22667542671381e-05,"8","Gadd45b"
"Sgk13",4.76942368662953e-09,1.27712608389391,0.84,0.547,8.04220222039472e-05,"8","Sgk1"
"Sqstm11",6.29321361599075e-09,0.76801870336935,0.963,0.746,0.000106116167992836,"8","Sqstm1"
"Slc23a2",1.44042031418092e-08,0.34449417243828,0.444,0.187,0.000242883673377186,"8","Slc23a2"
"Renbp",1.73512955605498e-08,0.419571400496832,0.728,0.411,0.00029257754574199,"8","Renbp"
"Ccrl23",1.89254592190967e-08,0.725414657831643,0.926,0.671,0.000319121093352408,"8","Ccrl2"
"Gm26609",2.11194865604787e-08,0.274875330626405,0.259,0.08,0.000356116782382791,"8","Gm26609"
"Mir155hg",2.5618383333989e-08,0.465219314341838,0.296,0.101,0.000431977179777723,"8","Mir155hg"
"Abca11",2.98421179364782e-08,0.520342358906538,0.765,0.474,0.000503197792644896,"8","Abca1"
"Dot1l2",3.08623869667216e-08,0.405357560201625,0.691,0.383,0.00052040156903286,"8","Dot1l"
"Slc16a32",3.61664749876368e-08,0.594362412445823,0.679,0.401,0.000609839101241532,"8","Slc16a3"
"Lpl1",3.64670899768413e-08,1.11672623786401,0.691,0.442,0.000614908071189498,"8","Lpl"
"Pfkfb31",3.79912975970756e-08,0.60806938889327,0.704,0.427,0.000640609260081888,"8","Pfkfb3"
"Smim31",4.24524072683339e-08,0.428747169626853,0.691,0.387,0.000715832491358646,"8","Smim3"
"Ell21",7.35386198681663e-08,0.668083519730559,0.654,0.372,0.00124000820821702,"8","Ell2"
"Plek2",7.64246441565078e-08,0.649101287345176,1,0.835,0.00128867234976704,"8","Plek"
"Clic42",8.35862822410137e-08,0.535741799381141,0.778,0.472,0.00140943189114797,"8","Clic4"
"Extl3",9.27821938637626e-08,0.320479485088506,0.58,0.299,0.00156449335293077,"8","Extl3"
"Bsg1",9.33246837391287e-08,0.637039163171821,0.975,0.852,0.00157364081720919,"8","Bsg"
"Nceh1",1.16087841860888e-07,0.370682113982485,0.556,0.281,0.00195747318945829,"8","Nceh1"
"Dtnbp12",1.29293144191863e-07,0.367018337852602,0.728,0.44,0.00218014

099736319,"8","Dtnbp1"
"Dpp7",1.44648719612914e-07,0.265074917753165,0.42,0.183,0.00243906671011296,"8","Dpp7"
"Abcg11",1.46687794762422e-07,0.268236349328418,0.506,0.24,0.00247344959528396,"8","Abcg1"
"Itga51",1.64705252766173e-07,0.437574507573632,0.481,0.23,0.00277725997214322,"8","Itga5"
"Sdc31",1.69630806181803e-07,0.413601963655683,0.605,0.337,0.00286031465383756,"8","Sdc3"
"Slc11a2",1.75861264302515e-07,0.368825828397147,0.407,0.174,0.00296537263866902,"8","Slc11a2"
"C3ar12",1.82387207689212e-07,0.507682341663725,0.963,0.728,0.00307541309605548,"8","C3ar1"
"Got11",1.84585783672951e-07,0.383693600739094,0.543,0.273,0.0031124854842933,"8","Got1"
"Cadm11",2.00594626923389e-07,0.431006665405576,0.778,0.524,0.00338242659918219,"8","Cadm1"
"Slc2a11",2.02266889666243e-07,0.876541358362366,0.519,0.276,0.00341062429355219,"8","Slc2a1"
"Traf6",2.07666074763431e-07,0.27936054225405,0.259,0.088,0.00350166535266098,"8","Traf6"
"Prr5l1",2.15928294264356e-07,0.294674884171035,0.346,0.135,0.00364098289788557,"8","Prr5l"
"Sel1l",2.31735105870437e-07,0.338699461445541,0.519,0.259,0.0039075173551873,"8","Sel1l"
"Tnfaip32",2.3302258906158e-07,0.499014940445349,0.975,0.706,0.00392922689675637,"8","Tnfaip3"
"Plaur1",2.49716627502083e-07,0.472068598895927,0.802,0.487,0.00421072177294012,"8","Plaur"
"Tmbim11",2.70233349190305e-07,0.442742838979929,0.519,0.259,0.00455667473404693,"8","Tmbim1"
"Plekhm21",2.7063199037726e-07,0.390696250875575,0.543,0.275,0.00456339662174136,"8","Plekhm2"
"Txnrd11",2.78863579496221e-07,0.604384580089603,0.79,0.518,0.00470219767746529,"8","Txnrd1"
"Hilpda",2.86985570350718e-07,0.490807912695887,0.284,0.106,0.0048391506872538,"8","Hilpda"
"Sdc43",4.11229728001851e-07,1.20471300306086,0.63,0.4,0.00693415567356722,"8","Sdc4"
"Cd721",4.62378286465402e-07,0.583903390519176,0.617,0.355,0.00779662266637961,"8","Cd72"
"Ldha2",5.20560929467263e-07,0.585674339888892,0.951,0.798,0.00877769839267699,"8","Ldha"
"Gpr842",5.73243088691727e-07,0.681397408226954,0.901,0.729,0.00966602496151991,"8","Gpr84"
"S1pr1",6.04964062495405e-07,0.30409152914469,0.395,0.17,0.0102009040217975,"8","S1pr1"
"Lgals3bp1",6.0862684254635e-07,0.489360973069805,0.84,0.547,0.0102626658190166,"8","Lgals3bp"
"Pdgfa",6.67827043722798e-07,0.494642761270454,0.494,0.244,0.011260899

6112538,"8","Pdgfa"
"Arhgap24",6.75018912889554e-07,0.33509506927849,0.358,0.151,0.0113821689091437,"8","Arhgap24"
"Nfe2l23",6.93149707774104e-07,0.559230035291324,0.926,0.718,0.0116878903724869,"8","Nfe2l2"
"Ptgs22",7.15646932149091e-07,1.12377737381416,0.346,0.147,0.012067238569898,"8","Ptgs2"
"Gcnt22",8.1323423551346e-07,0.398006004901328,0.519,0.249,0.013712755679228,"8","Gcnt2"
"Aldoa1",9.1209365928766e-07,0.563911129170586,0.938,0.823,0.0153797232829085,"8","Aldoa"
"Naglu",9.37980782268307e-07,0.501760747521872,0.716,0.451,0.0158162319506082,"8","Naglu"
"Abhd121",9.42635750813608e-07,0.366779712716904,0.988,0.885,0.0158947240302191,"8","Abhd12"
"Zfand52",1.00923966674728e-06,0.518981244641891,0.975,0.767,0.0170177992606927,"8","Zfand5"
"Syngr1",1.05149850472345e-06,0.404539681124842,0.926,0.697,0.0177303677866468,"8","Syngr1"
"Rasgef1b2",1.12186033412301e-06,0.555471061873402,0.691,0.413,0.0189168089539822,"8","Rasgef1b"
"Irgm11",1.20194788590592e-06,0.381963083434695,0.333,0.143,0.0202672452521457,"8","Irgm1"
"Myo1e",1.36120359732637e-06,0.399362269707048,0.42,0.209,0.0229526150581172,"8","Myo1e"
"Mpeg11",1.49770060964256e-06,0.38134488706866,0.975,0.861,0.0252542276797928,"8","Mpeg1"
"Gna131",1.51095162324678e-06,0.577816667816631,0.827,0.561,0.0254776662711871,"8","Gna13"
"Aplp21",1.55054445879862e-06,0.503624170347449,0.728,0.475,0.0261452806642624,"8","Aplp2"
"Eno12",1.72498485398984e-06,0.514121029690191,0.877,0.669,0.0290866946079767,"8","Eno1"
"Eid31",1.72837076730112e-06,0.478218839581335,0.481,0.251,0.0291437878782315,"8","Eid3"
"Epb41l3",2.10196749825343e-06,0.356859565005795,0.457,0.235,0.0354433759555493,"8","Epb41l3"
"Cd843",2.33725075091556e-06,0.366887304777617,0.938,0.654,0.0394107221619382,"8","Cd84"
"Slc15a31",2.36800150307952e-06,0.432349668636402,0.901,0.69,0.0399292413449269,"8","Slc15a3"
"March5",2.38585439048991e-06,0.389214078538434,0.691,0.435,0.0402302767324408,"8","March5"
"Dusp12",2.59041822565749e-06,0.443935126668152,0.975,0.789,0.0436796321210366,"8","Dusp1"
"Cndp2",2.64169615534412e-06,0.298613998265143,0.827,0.603,0.0445442805714125,"8","Cndp2"
"Ptafr2",2.69382879283949e-06,0.358894803801038,0.753,0.444,0.0454233411048595,"8","Ptafr"
"Rab202",2.72872486582811e-06,0.44345652572356,0.753,0.507,0.046011758

6875936,"8","Rab20"
"Slc43a21",2.76976687620119e-06,0.336042714419351,0.605,0.345,0.046703
8090665045,"8","Slc43a2"
"Zeb24",2.93104766978236e-06,0.497767164651988,1,0.785,0.0494233258078
701,"8","Zeb2"
"Mfsd12",3.35104417251126e-06,0.347862535613593,0.519,0.273,0.05650530
68368848,"8","Mfsd12"
"Ifih11",3.42767312614376e-06,0.535685607057583,0.42,0.212,0.057797424
253036,"8","Ifih1"
"Chst2",3.5820960710635e-06,0.35762065635789,0.259,0.099,0.06040130395
02728,"8","Chst2"
"Nfkbiz4",3.77059996653269e-06,0.487198696708009,0.988,0.777,0.0635798
566356742,"8","Nfkbiz"
"Creg1",3.81794213744544e-06,0.430567012282118,0.975,0.819,0.064378140
321605,"8","Creg1"
"Ftl11",3.83424146509782e-06,0.268688801920891,1,0.997,0.0646529795844
794,"8","Ftl1"
"Mif",4.12720047698931e-06,0.48147855531739,0.654,0.445,0.069592854442
9937,"8","Mif"
"Fam162a1",4.51951511506651e-06,0.262997805446006,0.407,0.201,0.076208
0638702515,"8","Fam162a"
"Ifnar11",4.55962753578632e-06,0.353182378526226,0.605,0.335,0.0768844
39508429,"8","Ifnar1"
"Calr",4.56760385278431e-06,0.403444956483713,1,0.905,0.07701893616564
91,"8","Calr"
"Maged1",4.61737695510312e-06,0.314800630385356,0.432,0.209,0.07785821
02169487,"8","Maged1"
"Trem22",5.02848614331375e-06,0.322997669405432,1,0.932,0.084790333348
5564,"8","Trem2"
"Tgif13",5.20338814164449e-06,0.421124854564106,0.951,0.707,0.08773953
08444093,"8","Tgif1"
"Dnmt3a1",5.2044049420748e-06,0.281806695528768,0.753,0.438,0.08775667
61332653,"8","Dnmt3a"
"Osm2",5.32559652671161e-06,0.536161309266825,0.741,0.442,0.0898002086
334111,"8","Osm"
"Tpi12",6.03415764106334e-06,0.666031141611937,0.79,0.589,0.1017479661
4361,"8","Tpi1"
"Tpp11",6.09546082682536e-06,0.377833976071106,0.877,0.654,0.102781660
461929,"8","Tpp1"
"Serpine24",6.22509483054039e-06,0.424280824015583,0.975,0.734,0.10496
7549032572,"8","Serpine2"
"Pvr2",6.84324903044835e-06,0.255066416829362,0.42,0.198,0.11539086515
142,"8","Pvr"
"Socs33",7.66287874741583e-06,0.587800702205008,0.975,0.766,0.12921146
1438926,"8","Socs3"
"Slc38a10",7.6644754381833e-06,0.361818862751002,0.691,0.447,0.1292383
84838647,"8","Slc38a10"
"Mgat5",8.10297646398426e-06,0.287134987255681,0.494,0.268,0.136632389
135703,"8","Mgat5"
"Itgav2",8.13416523843653e-06,0.351433368184105,0.654,0.426,0.13715829

4250517,"8","Itgav"
"Fth11",8.25036369174811e-06,0.300247707958174,1,0.994,0.139117632570257,"8","Fth1"
"Gde11",8.3250077402343e-06,0.364367950686908,0.753,0.529,0.140376280515831,"8","Gde1"
"Nes1",8.75035176028767e-06,0.506093451083547,0.58,0.337,0.147548431381971,"8","Nes"
"Dusp23",8.89509640485335e-06,0.554086575784329,0.802,0.563,0.149989115578637,"8","Dusp2"
"Gnl31",8.9763375432466e-06,0.292008142298508,0.58,0.333,0.151359003654224,"8","Gnl3"
"Bnip3",9.00206237402271e-06,0.673656493240999,0.481,0.278,0.151792775750771,"8","Bnip3"
"Npnt",9.13202660528992e-06,0.362476317471585,0.494,0.277,0.153984232618399,"8","Npnt"
"Fam20c1",9.22013636647208e-06,0.622721261969999,0.259,0.11,0.155469939411452,"8","Fam20c"
"Cxcl103",9.51165446228077e-06,0.962753813994012,0.58,0.356,0.160385517542978,"8","Cxcl10"
"P4ha1",9.89753081335421e-06,0.316464858073979,0.667,0.416,0.166892164574779,"8","P4ha1"
"Gusb1",1.14333924739119e-05,0.364767675090438,0.938,0.798,0.192789863895103,"8","Gusb"
"Hif1a1",1.36107455968342e-05,0.398609500573983,0.704,0.49,0.229504392253818,"8","Hif1a"
"Tmem86a1",1.42478232016719e-05,0.302161616230852,0.864,0.627,0.240246794826591,"8","Tmem86a"
"Lrp11",1.6066108795298e-05,0.333760443025266,0.815,0.626,0.270906726506314,"8","Lrp1"
"Igf11",1.69470853235224e-05,0.63044990200608,0.444,0.249,0.285761752725235,"8","Igf1"
"M6pr1",1.74162964500093e-05,0.341589564939951,0.84,0.632,0.293673590740057,"8","M6pr"
"Nfkbib1",1.7773431985695e-05,0.385497382641593,0.815,0.543,0.299695610142789,"8","Nfkbib"
"Tnfaip23",1.82963614406265e-05,0.507232246817018,0.642,0.389,0.308513246611844,"8","Tnfaip2"
"AU0202061",1.83997362316665e-05,0.296839760721162,0.654,0.411,0.31025635233836,"8","AU020206"
"Gapdh2",1.88103069351256e-05,0.516447198319561,1,0.954,0.317179395540088,"8","Gapdh"
"Frrs1",1.88849531671461e-05,0.320750807470979,0.691,0.43,0.318438080304417,"8","Frrs1"
"Basp11",1.97264420531942e-05,0.437741696612303,1,0.884,0.332627265900961,"8","Basp1"
"Bcl2a1b2",2.12350272014027e-05,0.405706304931506,0.988,0.858,0.358065028670052,"8","Bcl2a1b"
"Neat13",2.25592052548388e-05,0.488332990295815,1,0.755,0.380393319007092,"8","Neat1"
"Abhd17c1",2.3329516556775e-05,0.312000564782513,0.444,0.235,0.3933823

08180339,"8","Abhd17c"
"Pgk13",2.65446773138738e-05,0.596364414896071,0.778,0.573,0.447596348
86654,"8","Pgk1"
"Ctsl1",2.79621337362298e-05,0.343941337708912,1,0.938,0.4714974990603
07,"8","Ctsl"
"Bcl2a1d3",2.92593310651438e-05,0.25122226176618,0.667,0.388,0.4933708
40420455,"8","Bcl2a1d"
"Soat11",3.00235080852033e-05,0.307107781338584,0.605,0.377,0.50625639
3332698,"8","Soat1"
"Rhoc1",3.05793972114344e-05,0.411060402543914,0.642,0.437,0.515629795
779208,"8","Rhoc"
"Lgals32",3.14147689225787e-05,0.546166466512748,0.444,0.235,0.5297158
33572522,"8","Lgals3"
"Arl4c1",3.1875798081746e-05,0.369321308194688,0.864,0.684,0.537489707
254402,"8","Arl4c"
"Egln1",3.28378672731933e-05,0.443257635233344,0.58,0.372,0.5537121179
60585,"8","Egln1"
"Fcrls2",3.29995053703742e-05,0.409231738790126,0.988,0.882,0.55643765
955525,"8","Fcrls"
"Phlda12",3.3311067946122e-05,0.631944414423819,0.593,0.368,0.56169122
7707509,"8","Phlda1"
"Vps352",3.62769286727991e-05,0.27725577527691,0.765,0.534,0.611701571
280738,"8","Vps35"
"Cflar2",3.67442206677072e-05,0.341642042675718,0.901,0.655,0.61958104
8898879,"8","Cflar"
"Mylip3",3.97456427519376e-05,0.628383846493096,0.877,0.637,0.67019102
8083171,"8","Mylip"
"Lilr4b2",4.02617374021363e-05,0.568307740813368,0.296,0.135,0.6788934
16074823,"8","Lilr4b"
"Gm269172",4.0430534727219e-05,0.409433392322612,0.457,0.265,0.6817396
76570367,"8","Gm26917"
"Pdgfb3",4.29930159194524e-05,0.561028226113601,0.802,0.528,0.72494823
4433807,"8","Pdgfb"
"Nek6",4.34317197899085e-05,0.261643605692058,0.543,0.315,0.7323456590
97438,"8","Nek6"
"Pdia61",4.36709550680944e-05,0.345349929909464,0.889,0.707,0.73637964
4358208,"8","Pdia6"
"Parp9",4.51306551583876e-05,0.271490132224002,0.519,0.287,0.760993107
280731,"8","Parp9"
"Ero1l",4.58833798015123e-05,0.345515069917711,0.296,0.139,0.773685550
2131,"8","Ero1l"
"Pfkl",4.71149084683783e-05,0.51247799285488,0.568,0.363,0.79445158659
3795,"8","Pfkl"
"C1qbp1",4.73775939991453e-05,0.263688872192802,0.642,0.437,0.79888099
0013589,"8","C1qbp"
"Pitpnc11",4.76587272450962e-05,0.296649349502053,0.543,0.316,0.803621
458806812,"8","Pitpnc1"
"Hsp90b12",4.79031276689018e-05,0.375686590207169,0.988,0.856,0.807742
538753021,"8","Hsp90b1"
"C1qb2",5.01032028998443e-05,0.254303210168827,1,0.946,0.8448402072971

74,"8","C1qb"
"C5ar11",5.23773443049061e-05,0.46612395755668,0.889,0.715,0.883186779
669327,"8","C5ar1"
"Gem2",5.58293043804422e-05,0.545036239905186,0.654,0.434,0.9413937304
63017,"8","Gem"
"Grhpr",5.95647798146369e-05,0.349258476222112,0.296,0.138,1,"8","Grhp
r"
"Pgam12",6.09565209807506e-05,0.578099924044462,0.864,0.678,1,"8","Pga
m1"
"Hexa2",6.23654263569197e-05,0.311608552418504,0.988,0.904,1,"8","Hexa
"
"Capg1",6.5688557306573e-05,0.437521164354075,0.568,0.36,1,"8","Capg"
"Slc37a2",6.75614767064923e-05,0.32736694873536,0.531,0.31,1,"8","Slc3
7a2"
"Tns3",6.86696086456449e-05,0.304052331111661,0.642,0.423,1,"8","Tns3"
"Il1b4",6.9924063045342e-05,0.732990880109898,0.728,0.56,1,"8","Il1b"
"Nampt1",7.12122397379222e-05,0.316061391253748,0.432,0.234,1,"8","Nam
pt"
"Hspa51",7.96252614819166e-05,0.369913149410302,1,0.888,1,"8","Hspa5"
"Slc6a62",7.97098028890691e-05,0.285534646938645,0.901,0.663,1,"8","Sl
c6a6"
"Rcan12",8.74079524800293e-05,0.319020973089994,0.753,0.516,1,"8","Rca
n1"
"Lman1",8.77321711679881e-05,0.283888660573011,0.58,0.351,1,"8","Lman1
"
"Gch12",9.12970672679653e-05,0.61364763628744,0.543,0.33,1,"8","Gch1"
"Cdkn1a1",0.000105554583390223,0.447963940154357,0.753,0.538,1,"8","Cd
kn1a"
"Arl8a",0.000123490206107949,0.278036011974231,0.728,0.506,1,"8","Arl8
a"
"Il1rn1",0.000125203564498564,0.660760429714026,0.259,0.118,1,"8","Il1
rn"
"Nfkbid2",0.000133234461848108,0.490007718586446,0.901,0.695,1,"8","Nf
kbid"
"Sdf2l11",0.000137552110644457,0.319426020681471,0.765,0.531,1,"8","Sd
f2l1"
"Rhbdf2",0.00014614847559588,0.269730722762675,0.358,0.187,1,"8","Rhbd
f2"
"Glipr12",0.000161043164635591,0.428412242535401,0.568,0.367,1,"8","Gl
ipr1"
"Osbpl8",0.000166774162702751,0.370971503801953,0.481,0.282,1,"8","Osb
pl8"
"Lmna3",0.000168402111561091,0.385851571384845,0.457,0.253,1,"8","Lmna
"
"Scarb2",0.000169819900886106,0.266346394378492,0.815,0.625,1,"8","Sca
rb2"
"Tmed31",0.000172589308234919,0.26229368460394,0.765,0.577,1,"8","Tmed
3"
"Adamts12",0.000184841584906719,0.486518969387208,0.346,0.176,1,"8","A
damts1"

"Slc11a11",0.000186130929267801,0.426038223065878,0.901,0.726,1,"8","S
lc11a1"
"Ranbp21",0.000198641999443683,0.365751709847319,0.716,0.506,1,"8","Ra
nbp2"
"Atp6v1c1",0.000199441023314657,0.307179744473596,0.679,0.462,1,"8","A
tp6v1c1"
"Slc35f6",0.000203612316126779,0.301334152338852,0.407,0.24,1,"8","Slc
35f6"
"Pkm1",0.000210158262235317,0.656647765560145,0.926,0.851,1,"8","Pkm"
"Hmox11",0.000215073408310126,0.870676868026738,0.914,0.728,1,"8","Hmo
x1"
"Otud11",0.000226832568199923,0.511661407338755,0.321,0.165,1,"8","Otu
d1"
"Nfkb11",0.000260648847560808,0.310537336159065,0.926,0.717,1,"8","Nfk
b1"
"Trim351",0.000268234255398268,0.334590974315257,0.457,0.277,1,"8","Tr
im35"
"Peli11",0.000297447248169888,0.324186348933969,0.84,0.57,1,"8","Peli1
"
"Nrbf2",0.000317584715780952,0.271376301681131,0.617,0.411,1,"8","Nrbf
2"
"Gns",0.0003359526858011,0.280327647687064,0.877,0.67,1,"8","Gns"
"Ier33",0.000357822323927164,0.350951627997369,0.975,0.799,1,"8","Ier3
"
"Eprs",0.000391342631233679,0.267295591269282,0.704,0.486,1,"8","Eprs"
"Lipa1",0.000416067068803052,0.422941368916624,0.704,0.485,1,"8","Lipa
"
"Atp6v1d",0.000424564692219618,0.250536848614898,0.691,0.458,1,"8","At
p6v1d"
"Sgms11",0.000510720035287369,0.28573355389941,0.284,0.145,1,"8","Sgms
1"
"Atf31",0.000574838199719392,0.533739657357037,0.901,0.749,1,"8","Atf3
"
"Gm170562",0.000577250347319345,0.438405581668122,0.617,0.439,1,"8","G
m17056"
"Cpeb41",0.00062159677319559,0.270684767855153,0.543,0.365,1,"8","Cpeb
4"
"Srgn3",0.000716818554720277,0.316784582380774,1,0.849,1,"8","Srgn"
"Kdm6b2",0.000765925026381722,0.310173423283332,0.938,0.741,1,"8","Kdm
6b"
"Plekho22",0.000773369247908902,0.277621325014966,0.889,0.629,1,"8","P
lekho2"
"Smad72",0.000849439781433894,0.301548235911237,0.704,0.507,1,"8","Sma
d7"
"Manf",0.000850826059867377,0.265162086577854,0.802,0.623,1,"8","Manf"
"Mthfsl2",0.000854441686156956,0.290789206316584,0.506,0.317,1,"8","Mt
hfsl"
"Cd862",0.000861237344918507,0.277598723235573,0.901,0.708,1,"8","Cd86
"
"Por",0.000874593613789685,0.335803876278411,0.444,0.276,1,"8","Por"

"Stt3b1",0.000888076151241897,0.260166946303707,0.481,0.313,1,"8","Stt
3b"
"Macf13",0.000969986519421167,0.294523386098186,0.914,0.718,1,"8","Mac
f1"
"Bhlhe411",0.000995886378261877,0.266222578157518,0.568,0.377,1,"8","B
hlhe41"
"Bcl2a1a2",0.00101090927192442,0.288754181296057,0.568,0.389,1,"8","Bc
l2a1a"
"Cln5",0.00103458900497723,0.279767639631968,0.568,0.375,1,"8","Cln5"
"Gclc1",0.0011005349616207‌5,0.744113553502478,0.469,0.291,1,"8","Gclc"
"Nktr1",0.00112029101863797,0.360767540896527,0.815,0.635,1,"8","Nktr"
"Psmb61",0.00117689796192572,0.255858121229014,0.765,0.645,1,"8","Psmb
6"
"Tmem882",0.0013003304418266,0.318526421565624,0.605,0.428,1,"8","Tmem
88"
"Skil3",0.00130107692321838,0.315201450668972,0.988,0.792,1,"8","Skil"
"Nr4a13",0.00139677244842887,0.256769885415026,0.84,0.588,1,"8","Nr4a1
"
"Rgs12",0.00141962644788095,0.752248216276395,0.494,0.339,1,"8","Rgs1"
"Prdx1",0.00149080845466177,0.288924647189902,0.963,0.86,1,"8","Prdx1"
"Cdk61",0.00151836811805975,0.295887759820958,0.481,0.322,1,"8","Cdk6"
"Lrpap11",0.00154287007753603,0.299493423794928,0.728,0.558,1,"8","Lrp
ap1"
"Clcn4",0.00163123641933342,0.253087539023356,0.741,0.498,1,"8","Clcn4
"
"Zfp364",0.00167341727750601,0.294022273164763,1,0.839,1,"8","Zfp36"
"Chka2",0.00198985699394839,0.373962043848439,0.494,0.342,1,"8","Chka"
"Arid5b1",0.00212789687290785,0.261461340021973,0.481,0.315,1,"8","Ari
d5b"
"Slfn23",0.00221175569826575,0.37938884403024,0.914,0.684,1,"8","Slfn2
"
"Snx29",0.00236715135176648,0.362102237345791,0.469,0.309,1,"8","Snx29
"
"Srxn12",0.00238436127979465,0.544054710398532,0.494,0.33,1,"8","Srxn1
"
"Itpkb3",0.00243530742574905,0.327503742316815,0.568,0.389,1,"8","Itpk
b"
"Mt12",0.00254270466262235,0.459564240972542,0.79,0.592,1,"8","Mt1"
"Gm201862",0.00255572390013384,0.64303446797814,0.321,0.191,1,"8","Gm2
0186"
"Sod22",0.00256268637321851,0.259445070696191,0.691,0.498,1,"8","Sod2"
"Hivep31",0.00268431560780176,0.386630993374198,0.605,0.442,1,"8","Hiv
ep3"
"Alas1",0.00277485051218521,0.40718642898156,0.531,0.352,1,"8","Alas1"
"Lpp",0.00283963861405833,0.27840406101656,0.432,0.275,1,"8","Lpp"
"Tor3a1",0.00297005197873937,0.330997066340936,0.481,0.317,1,"8","Tor3
a"
"Pdia41",0.00307613806831163,0.273307817944158,0.531,0.38,1,"8","Pdia4
"
"Slc48a1",0.00333803405853207,0.315619974153164,0.457,0.3,1,"8","Slc48

a1"
"Sema4d2",0.00357431014206248,0.263103540600951,0.716,0.535,1,"8","Sem
a4d"
"Ifi2042",0.00359245068070015,0.531972144793251,0.506,0.353,1,"8","Ifi
204"
"Tet22",0.00381934130009656,0.258837661559148,0.469,0.315,1,"8","Tet2"
"Hvcn1",0.00386336184410977,0.251636903721636,0.654,0.456,1,"8","Hvcn1
"
"Parp141",0.00393369618828825,0.509624546230354,0.481,0.328,1,"8","Par
p14"
"Gm12840",0.00411191030055388,0.385374099973313,0.272,0.153,1,"8","Gm1
2840"
"Sh3bp2",0.00420679684301159,0.277360485235776,0.296,0.175,1,"8","Sh3b
p2"
"Irf12",0.0048058973299376,0.396253265294755,0.568,0.417,1,"8","Irf1"
"Isg152",0.00501681847326226,0.445942247219087,0.309,0.189,1,"8","Isg1
5"
"Anxa21",0.00544225246143386,0.258664501050172,0.42,0.273,1,"8","Anxa2
"
"Gclm",0.00561008940049357,0.38353732255405,0.481,0.319,1,"8","Gclm"
"Gpi12",0.00634088967897395,0.262813020354249,0.938,0.782,1,"8","Gpi1"
"Zfp7041",0.00706920986808395,0.255167613623151,0.259,0.151,1,"8","Zfp
704"
"Glrx1",0.00708157669595794,0.38624287337183,0.568,0.396,1,"8","Glrx"
"Ncf12",0.00767000823406299,0.318604317583757,0.951,0.788,1,"8","Ncf1"
"Mbtd11",0.00879048375110943,0.256647413605862,0.481,0.334,1,"8","Mbtd
1"
"Lgals33",7.84146066382859e-51,3.19567204393885,0.908,0.22,1.322227097
13478e-46,"9","Lgals3"
"Fabp51",4.09267007313611e-49,3.29517437360644,0.892,0.234,6.901060277
32211e-45,"9","Fabp5"
"Gclm1",8.28942544410027e-44,3.39479277583433,0.892,0.306,1.3977629183
8419e-39,"9","Gclm"
"Ftl12",2.29226861599197e-42,3.44934850696986,1,0.997,3.86522334028566
e-38,"9","Ftl1"
"Fth12",6.70718077566146e-42,3.37792508427493,1,0.994,1.13096482239204
e-37,"9","Fth1"
"Prdx11",2.14460332715565e-40,4.03019185813474,1,0.859,3.6162301302498
5e-36,"9","Prdx1"
"Slc48a11",6.60337081000759e-38,3.01241465227708,0.831,0.288,1.1134603
8598348e-33,"9","Slc48a1"
"Hmox12",1.23624766031941e-36,3.87454376051574,0.985,0.727,2.084560804
83059e-32,"9","Hmox1"
"Igf12",4.34966041632675e-35,2.46027761615853,0.785,0.238,7.3343973940
1017e-31,"9","Igf1"
"Aldoa2",5.91747263348201e-35,2.4481021498687,0.985,0.823,9.9780423545
7737e-31,"9","Aldoa"
"Ftl1-
ps1",2.15639462728846e-34,2.71160850946016,0.846,0.346,3.6361126205338
1e-30,"9","Ftl1-ps1"

"Gapdh3",2.31526377385976e-33,2.10183290832947,1,0.955,3.9039977754823
3e-29,"9","Gapdh"
"Esd",2.84701074404767e-32,3.00774179310812,0.938,0.611,4.800629516613
18e-28,"9","Esd"
"Taldo11",9.62849563654247e-31,2.39179746345584,0.938,0.79,1.623556934
23379e-26,"9","Taldo1"
"Pkm2",1.62724944321379e-29,2.34815159990995,0.969,0.85,2.743868011147
1e-25,"9","Pkm"
"Cd632",1.07938136724044e-27,1.621531415499,0.985,0.883,1.820052861440
83e-23,"9","Cd63"
"Ctsd3",2.15567423058234e-27,1.58026638759535,1,0.971,3.63489788760794
e-23,"9","Ctsd"
"Creg11",2.32254052013101e-27,2.33926709961158,0.938,0.822,3.916267825
0449e-23,"9","Creg1"
"Pgam13",1.93654743106193e-23,2.04106243418634,0.892,0.679,3.265406278
25662e-19,"9","Pgam1"
"Pgk14",2.49705490523539e-23,2.04847392968202,0.877,0.571,4.2105339812
0792e-19,"9","Pgk1"
"Eno13",1.39180626914595e-22,2.10330402320646,0.892,0.67,2.34686373103
39e-18,"9","Eno1"
"Gsr1",4.35531718012964e-21,1.61308552965048,0.677,0.244,7.34393582913
46e-17,"9","Gsr"
"Blvrb1",5.11733130435745e-21,1.95167346214639,0.815,0.523,8.628844045
40754e-17,"9","Blvrb"
"Cyb5a",1.14037936644948e-20,1.99649387387308,0.815,0.596,1.9229076877
0711e-16,"9","Cyb5a"
"Cd681",1.77006285326672e-19,1.26435150850157,0.938,0.903,2.9846799831
7835e-15,"9","Cd68"
"Lpl2",3.16842812612866e-19,2.04735147310524,0.8,0.44,5.34260350627814
e-15,"9","Lpl"
"Aldh22",1.35956114710436e-18,1.69244749378434,0.785,0.51,2.2924920062
4736e-14,"9","Aldh2"
"Akr1a13",2.57132576799852e-18,1.22160606568942,0.892,0.86,4.335769509
9991e-14,"9","Akr1a1"
"Ctsb2",6.7119359757899e-18,1.18111785608383,1,0.974,1.13176664423769e
-13,"9","Ctsb"
"Anxa52",1.12909674972678e-17,1.82056111835571,0.754,0.436,1.903882939
3893e-13,"9","Anxa5"
"Vat11",1.51900362533975e-16,1.66231435363824,0.446,0.129,2.5613439130
4788e-12,"9","Vat1"
"Ctsz3",9.47073832159446e-16,0.889970062015908,0.985,0.974,1.596955895
78726e-11,"9","Ctsz"
"Tpi13",1.0500157102545e-15,2.00245884526231,0.8,0.59,1.77053649063114
e-11,"9","Tpi1"
"Mif1",3.90209125832787e-15,1.72256412451916,0.708,0.445,6.57970627979
246e-11,"9","Mif"
"Cstb1",2.97402594142548e-14,1.76457884843178,0.723,0.475,5.0148025424
3165e-10,"9","Cstb"
"Ass11",3.02337529527874e-14,1.20627945913687,0.262,0.049,5.0980154228
9901e-10,"9","Ass1"

"Gabarap1",4.85033152981063e-14,0.792746738004736,0.923,0.927,8.178629
02556669e-10,"9","Gabarap"
"Capg2",6.42125932655858e-14,1.74237086280547,0.662,0.358,1.0827527476
4431e-09,"9","Capg"
"Vim1",1.02163125355463e-12,1.64813381587745,0.631,0.29,1.722674619743
82e-08,"9","Vim"
"Cyba1",1.92678315597616e-12,0.756675299440121,0.985,0.955,3.248941757
60701e-08,"9","Cyba"
"Rplp03",2.65703903203645e-12,0.576744444120835,1,0.984,4.480299215819
86e-08,"9","Rplp0"
"Ldha3",8.33002963692975e-12,1.51753419206758,0.815,0.805,1.4046095973
791e-07,"9","Ldha"
"Gpi13",1.87257277786222e-11,1.12725790606092,0.8,0.788,3.157532218031
28e-07,"9","Gpi1"
"Cfl12",5.92846489194241e-11,0.630650359969024,0.985,0.98,9.9965775007
9329e-07,"9","Cfl1"
"Rps210",5.96185209191623e-11,0.686387059084981,0.969,0.988,1.00528749
973891e-06,"9","Rps2"
"Oaz12",6.19035260862673e-11,0.680875205279512,0.908,0.97,1.0438172568
6664e-06,"9","Oaz1"
"Gstm11",1.13833610459436e-10,1.18825538623395,0.308,0.087,1.919462339
56701e-06,"9","Gstm1"
"Bsg2",1.30325609359874e-10,1.22683804722488,0.831,0.858,2.19755042502
62e-06,"9","Bsg"
"Eif5a2",2.41616870127099e-10,0.948235646285513,0.877,0.873,4.07414366
408314e-06,"9","Eif5a"
"Psma72",1.08002356514566e-09,1.14325031171581,0.738,0.662,1.821135735
54861e-05,"9","Psma7"
"Psmb52",1.08825690679823e-09,1.3945184289785,0.708,0.616,1.8350187962
4317e-05,"9","Psmb5"
"Prdx61",1.26252148297736e-09,2.51073916913418,0.569,0.343,2.128863724
59642e-05,"9","Prdx6"
"Anxa22",1.35238013676645e-09,1.08558278077495,0.554,0.27,2.2803833866
1559e-05,"9","Anxa2"
"Slc40a11",1.82817425507125e-09,1.52270710448327,0.538,0.295,3.0826674
2890114e-05,"9","Slc40a1"
"Gnas1",4.93901660519654e-09,0.60338242748132,0.969,0.97,8.32816979968
241e-05,"9","Gnas"
"Plin22",6.16545003156404e-09,1.24937408067464,0.677,0.472,0.000103961
818432233,"9","Plin2"
"Psmb62",1.42823274352144e-08,1.2264066376359,0.708,0.648,0.0002408286
05212585,"9","Psmb6"
"Lipa2",2.35683118002144e-08,1.33486586159869,0.615,0.491,0.0003974088
73575216,"9","Lipa"
"Psma32",2.47041717768163e-08,0.988652533020107,0.723,0.696,0.00041656
1744500676,"9","Psma3"
"Rbm33",2.68508272439218e-08,0.622931469755294,0.923,0.924,0.000452758
64898701,"9","Rbm3"
"Cst73",3.70683221245081e-08,1.02739216142209,0.462,0.226,0.0006250460
47663456,"9","Cst7"

"Gpx41",6.48088167345207e-08,0.97396044269914,0.769,0.823,0.0010928062
6777749,"9","Gpx4"
"Syngr11",1.30661470363168e-07,1.15253603372063,0.723,0.707,0.00220321
371326373,"9","Syngr1"
"Spp1",1.33496465148016e-07,2.3764748711785,0.323,0.122,0.002251017395
32585,"9","Spp1"
"Lyz22",1.68262592229556e-07,0.755434271864922,0.646,0.373,0.002837243
83017477,"9","Lyz2"
"Sh3bgrl32",1.98955929481514e-07,0.550725357397288,0.908,0.917,0.00335
479488291729,"9","Sh3bgrl3"
"Calr1",3.25273481133917e-07,0.863497960637198,0.908,0.909,0.005484761
43888012,"9","Calr"
"Gde12",3.73017444548887e-07,1.24312893541738,0.631,0.535,0.0062898201
4998333,"9","Gde1"
"Aprt1",4.70653077936678e-07,1.20098665472871,0.615,0.532,0.0079361522
0016826,"9","Aprt"
"Atp6v1g12",4.90344476217169e-07,0.830841872942249,0.785,0.841,0.00826
81885579739,"9","Atp6v1g1"
"Dap",8.13637399325746e-07,1.33740719875779,0.538,0.381,0.013719553827
4307,"9","Dap"
"Cd92",1.02403696170728e-06,0.565937159534782,0.954,0.893,0.0172673112
483082,"9","Cd9"
"Chchd2",1.46845939164045e-06,0.707818006498185,0.815,0.859,0.02476116
22618412,"9","Chchd2"
"Nme11",1.81689366180741e-06,0.801986229368576,0.6,0.481,0.03063646092
53966,"9","Nme1"
"Arpc1b3",2.21229234491403e-06,0.509544182153155,0.923,0.977,0.0373036
735199404,"9","Arpc1b"
"Tspo2",2.82733818458344e-06,0.922733575051439,0.723,0.726,0.047674576
468446,"9","Tspo"
"Lgals13",3.18278917082523e-06,1.08914615508165,0.385,0.188,0.05366819
0998455,"9","Lgals1"
"Mpc11",4.27987488007096e-06,0.926905293387252,0.754,0.79,0.0721672502
277565,"9","Mpc1"
"Tmed22",4.85366032787277e-06,0.85120510775963,0.692,0.74,0.0818424204
485906,"9","Tmed2"
"Bag12",5.24704056696599e-06,0.822906675699031,0.708,0.69,0.0884755980
401805,"9","Bag1"
"Sdf2l12",5.35451461659424e-06,1.13244129052588,0.6,0.539,0.0902878254
65012,"9","Sdf2l1"
"Cdc422",5.83171324031652e-06,0.54315731412193,0.923,0.959,0.098334348
6582171,"9","Cdc42"
"Cndp21",6.53364973576825e-06,0.911025796455892,0.662,0.611,0.11017040
1844524,"9","Cndp2"
"Atp6v0e1",6.95023221131788e-06,0.857926504625237,0.738,0.811,0.117194
815547242,"9","Atp6v0e"
"Hsp90ab11",8.16813921643235e-06,0.456889416561732,0.938,0.963,0.13773
1163467482,"9","Hsp90ab1"
"Ran1",8.72951051852333e-06,0.863381823930893,0.708,0.724,0.1471970063
6334,"9","Ran"

"Ybx13",9.59266114851999e-06,0.607689591515788,0.846,0.9,0.16175145228
6344,"9","Ybx1"
"Rpsa3",9.96881582691878e-06,0.384002111628807,0.969,0.987,0.168094172
473504,"9","Rpsa"
"Eef1g1",1.15615875272774e-05,0.794604236764145,0.723,0.801,0.19495148
8884952,"9","Eef1g"
"Ppia2",1.18648857591029e-05,0.496936874376273,0.908,0.971,0.200065703
669993,"9","Ppia"
"Bnip31",1.50460600970542e-05,1.19607443901732,0.446,0.281,0.253706665
356528,"9","Bnip3"
"Lamp12",1.66890800293576e-05,0.562583933715292,0.923,0.96,0.281411267
455028,"9","Lamp1"
"Calm11",1.86503582173608e-05,0.453493857843928,0.877,0.919,0.31448234
0261138,"9","Calm1"
"Psmd82",1.95276289841052e-05,1.17544435333646,0.677,0.685,0.329274879
929982,"9","Psmd8"
"Cope1",1.9656884080807e-05,1.00152312950898,0.585,0.544,0.33145437937
0567,"9","Cope"
"Psmb21",2.45496084863597e-05,0.853786795713031,0.646,0.636,0.41395549
8296997,"9","Psmb2"
"Prdx51",2.8791479414807e-05,0.70722685409355,0.754,0.837,0.4854819258
92476,"9","Prdx5"
"Ostf11",3.14829812359292e-05,0.853038107956297,0.723,0.836,0.53086602
9600239,"9","Ostf1"
"Rnh11",3.35341897217526e-05,1.31311535323536,0.677,0.69,0.56545350708
8192,"9","Rnh1"
"Gla1",3.63919633379388e-05,1.15439473351227,0.369,0.207,0.61364128580
4325,"9","Gla"
"Lamtor11",3.88884789050284e-05,0.798890578636324,0.708,0.719,0.655737
531296589,"9","Lamtor1"
"Psma22",7.88197057414035e-05,0.759170225928566,0.646,0.651,1,"9","Psm
a2"
"Ube2m1",8.77403268302173e-05,1.06242716884104,0.585,0.548,1,"9","Ube2
m"
"Selenof2",9.13353647979236e-05,0.596033061003129,0.831,0.859,1,"9","S
elenof"
"Atp5b1",9.24240316598104e-05,0.623691034779399,0.785,0.86,1,"9","Atp5
b"
"Srxn13",0.00012789943025946,0.956882473718319,0.477,0.332,1,"9","Srxn
1"
"Pld31",0.000137809646173552,0.960394220515643,0.569,0.527,1,"9","Pld3
"
"Gsn1",0.000170207073715588,0.706985357478961,0.338,0.178,1,"9","Gsn"
"Tkt2",0.000190240963320079,0.782527523738122,0.692,0.761,1,"9","Tkt"
"Ugp21",0.000191862119660497,1.10821659788764,0.431,0.3,1,"9","Ugp2"
"Cox4i12",0.000218133963131683,0.441193064837888,0.846,0.902,1,"9","Co
x4i1"
"Sdcbp1",0.000220902029649079,0.64936824251041,0.831,0.873,1,"9","Sdcb
p"
"Snx32",0.000245128159716211,0.659434188353909,0.738,0.762,1,"9","Snx3

"
"Arl1",0.000252947403934394,1.06779522003742,0.554,0.517,1,"9","Arl1"
"Rps32",0.000340063222919156,0.352307174726362,0.969,0.975,1,"9","Rps3
"
"Rhoa3",0.000359927305646882,0.39908717792668,0.892,0.937,1,"9","Rhoa"
"Pgd1",0.000396019521301208,1.219486687918,0.554,0.531,1,"9","Pgd"
"Pfn13",0.000450821254090751,0.379784897403386,0.892,0.949,1,"9","Pfn1
"
"Rhoc2",0.000573394923369529,1.07892691388639,0.508,0.444,1,"9","Rhoc"
"Bax1",0.000599951617556791,0.993768543897737,0.585,0.57,1,"9","Bax"
"Ap3s1",0.000664360767336195,0.688190418740526,0.446,0.334,1,"9","Ap3s
1"
"Bnip3l1",0.000687793896734565,0.922808651565828,0.615,0.625,1,"9","Bn
ip3l"
"Cmas",0.000714022356426966,1.0330276120495,0.415,0.313,1,"9","Cmas"
"Lilr4b3",0.000731745094393233,0.810108726028354,0.262,0.137,1,"9","Li
lr4b"
"Npc21",0.000774641739902567,0.378357894055561,0.908,0.964,1,"9","Npc2
"
"Ucp21",0.000870254232630089,0.50017466412875,0.785,0.906,1,"9","Ucp2"
"Anxa4",0.000916800939635686,0.893288417767438,0.308,0.183,1,"9","Anxa
4"
"Prelid12",0.00106745018225033,0.766703595389763,0.615,0.602,1,"9","Pr
elid1"
"Arhgdia2",0.00128960147277396,0.601360595834636,0.754,0.885,1,"9","Ar
hgdia"
"Rnf71",0.00141846969266474,0.791375803716377,0.615,0.654,1,"9","Rnf7"
"Vdac11",0.00142075770379872,0.902446795167279,0.554,0.555,1,"9","Vdac
1"
"Taf101",0.00142183106945675,0.766784427388564,0.538,0.511,1,"9","Taf1
0"
"Prdx21",0.00153301827359603,0.839401992829329,0.6,0.639,1,"9","Prdx2"
"Tbcb1",0.0015725474490226,0.934332773806342,0.508,0.467,1,"9","Tbcb"
"Psmb41",0.00169322247418401,0.843285135686038,0.615,0.656,1,"9","Psmb
4"
"Uap1l1",0.0021425107449829,0.829425139721509,0.431,0.346,1,"9","Uap1l
1"
"Txn12",0.00222306136953476,0.949465007703646,0.508,0.472,1,"9","Txn1"
"Ppib1",0.00224263832500039,0.497296703962841,0.862,0.873,1,"9","Ppib"
"Pebp11",0.0023105402570453,0.961553733431468,0.615,0.694,1,"9","Pebp1
"
"Ninj11",0.00255584391880258,0.882278101803244,0.615,0.646,1,"9","Ninj
1"
"Slc25a41",0.00300606125466195,0.746227088493171,0.631,0.679,1,"9","Sl
c25a4"
"Tmed101",0.00319093986309355,0.661507163963108,0.708,0.777,1,"9","Tme
d10"
"Arhgdib3",0.0037900585686903,0.449008421026972,0.738,0.873,1,"9","Arh
gdib"
"Tmem14c1",0.00393264130904804,0.47663863122301,0.646,0.715,1,"9","Tme

m14c"
"Cox5a2",0.00403963087653685,0.659568396749922,0.6,0.629,1,"9","Cox5a"
"Hspd11",0.00408931694279042,0.75515294767196,0.523,0.504,1,"9","Hspd1
"
"Ranbp11",0.00502828020241509,0.741503280958087,0.554,0.558,1,"9","Ran
bp1"
"Gltp1",0.0051534886695859,1.07264871740709,0.615,0.682,1,"9","Gltp"
"Mrpl41",0.0058524169469146,0.256139809276246,0.123,0.319,1,"9","Mrpl4
"
"Slc11a12",0.00589646009014063,0.823564599747244,0.631,0.738,1,"9","Sl
c11a1"
"Ube2k1",0.00617618304354995,0.320442368246252,0.231,0.498,1,"9","Ube2
k"
"Psmb31",0.0062299279745545,0.860346189877739,0.538,0.554,1,"9","Psmb3
"
"Sod23",0.00670171637231137,0.984505145168908,0.508,0.506,1,"9","Sod2"
"Gpr137b",0.00687958941129599,0.94591804574713,0.4,0.338,1,"9","Gpr137
b"
"Arpc32",0.00690471103312093,0.287575547845555,0.815,0.913,1,"9","Arpc
3"
"Rex1bd1",0.00720101441324105,0.757240055203712,0.385,0.304,1,"9","Rex
1bd"
"Cyth21",0.00785844843818669,0.26198402638446,0.169,0.374,1,"9","Cyth2
"
"Ufm1",0.00841436773695863,0.852377904180052,0.369,0.288,1,"9","Ufm1"
"Fxyd51",0.00874407800391412,0.45389803939947,0.492,0.4,1,"9","Fxyd5"
"Mrfap11",0.00926359421629525,0.417438449389085,0.723,0.813,1,"9","Mrf
ap1"
"Pf41",8.2102450423916e-66,3.08863607584829,0.883,0.121,1.384411519048
07e-61,"10","Pf4"
"Mrc11",9.1395840741679e-58,2.47576202208957,0.833,0.118,1.54111666658
619e-53,"10","Mrc1"
"Ms4a71",1.77227480820302e-53,2.51555084250377,0.8,0.119,2.98840978159
194e-49,"10","Ms4a7"
"F13a11",1.98289846732278e-46,2.38142476332944,0.683,0.097,3.343563395
59968e-42,"10","F13a1"
"Clec4n1",2.70077867742983e-35,2.27708631462894,0.683,0.137,4.55405300
588218e-31,"10","Clec4n"
"Igfbp42",6.16044245066485e-32,2.59431717534554,0.817,0.281,1.03877380
603111e-27,"10","Igfbp4"
"Dab21",1.46844624710011e-31,2.00183271883461,0.767,0.198,2.4760940618
602e-27,"10","Dab2"
"Ifi27l2a2",6.77189945353803e-31,2.61771668251697,0.833,0.297,1.141877
68585558e-26,"10","Ifi27l2a"
"Folr21",2.00297042686697e-30,2.11745981776494,0.517,0.089,3.377408733
78308e-26,"10","Folr2"
"Tpt11",1.29601234186514e-27,1.0415058467892,1,0.99,2.185336010853e-23
,"10","Tpt1"
"Ifitm32",3.35779496136928e-25,1.92829159558077,0.833,0.287,5.66191386
386088e-21,"10","Ifitm3"

"Snx22",5.32631143303431e-21,2.16767622967926,0.867,0.586,8.9812263383
8246e-17,"10","Snx2"
"Clta3",6.35355999008519e-21,1.40560719663106,0.983,0.959,1.0713372855
2816e-16,"10","Clta"
"Snx33",6.9616678245258e-19,1.72083276120457,0.9,0.757,1.1738764285715
4e-14,"10","Snx3"
"Ms4a6c2",1.42300676126393e-18,1.76729156099271,0.767,0.36,2.399474000
84323e-14,"10","Ms4a6c"
"Fcgrt1",1.60016037669559e-18,2.25136688204625,0.85,0.616,2.6981904271
841e-14,"10","Fcgrt"
"Lyve11",5.0486302714616e-18,1.75606121922813,0.35,0.062,8.51300036373
855e-14,"10","Lyve1"
"Pla2g72",1.15151053914803e-17,1.74518694049089,0.567,0.178,1.94167707
11114e-13,"10","Pla2g7"
"Tmsb4x2",1.34880759156403e-16,0.834652365961802,1,0.997,2.27435936089
527e-12,"10","Tmsb4x"
"Ybx14",7.16053341224018e-16,1.05179356366895,0.933,0.897,1.2074091439
7194e-11,"10","Ybx1"
"H3f3a2",7.98069030441807e-16,0.876857511053887,0.983,0.966,1.34570399
913097e-11,"10","H3f3a"
"Tmem176b1",9.38438409930249e-16,1.24181337696688,0.967,0.861,1.582394
84682439e-11,"10","Tmem176b"
"Cd1631",1.04149773705257e-15,1.59509452849078,0.317,0.061,1.756173484
21805e-11,"10","Cd163"
"Irf72",2.21092784745502e-15,1.97934008700746,0.55,0.202,3.72806653637
865e-11,"10","Irf7"
"Bst21",7.51248176538447e-15,1.99885614516811,0.8,0.596,1.266754675279
13e-10,"10","Bst2"
"Selenop1",1.33760919310595e-14,1.11101272936211,0.95,0.931,2.25547662
141525e-10,"10","Selenop"
"Aoah1",1.69598572652275e-14,1.4536319963478,0.367,0.091,2.85977113206
266e-10,"10","Aoah"
"Cfp2",1.13286532640704e-13,1.46992781763417,0.4,0.11,1.91023751338756
e-09,"10","Cfp"
"Gas61",2.60329324421897e-13,1.76824651967217,0.8,0.603,4.389673068402
03e-09,"10","Gas6"
"Blvrb2",3.95014559672782e-13,1.78889929808673,0.75,0.526,6.6607355052
0244e-09,"10","Blvrb"
"Ms4a6b2",4.92301484954103e-13,1.79712225981979,0.65,0.35,8.3011876392
9608e-09,"10","Ms4a6b"
"Pltp1",1.22822399574368e-12,1.09843175894345,0.317,0.076,2.0710313016
2299e-08,"10","Pltp"
"C21",1.91281978149546e-12,1.28876018839172,0.283,0.062,3.225396715557
64e-08,"10","C2"
"Ppia3",2.77495457527179e-12,0.751224179090253,0.95,0.969,4.6791284048
2329e-08,"10","Ppia"
"Rps3a12",3.31771640230647e-12,0.643610715220444,1,0.983,5.59433339756
917e-08,"10","Rps3a1"
"Gabarap2",3.60739442500472e-12,1.10450550299961,0.933,0.927,6.0827884
7944295e-08,"10","Gabarap"

"Smagp1",4.09133068332917e-12,1.88994682841613,0.583,0.3,6.89880179822
965e-08,"10","Smagp"
"Serp12",6.29828578514223e-12,1.58906772582916,0.817,0.719,1.062016949
09068e-07,"10","Serp1"
"Arpc33",7.21894325791051e-12,1.13541970443087,0.883,0.911,1.217258212
14887e-07,"10","Arpc3"
"Tgfbi2",8.55047544761923e-12,1.40862440855727,0.583,0.273,1.441781169
97756e-07,"10","Tgfbi"
"Akr1a14",1.65647705115258e-11,1.16868030205903,0.867,0.861,2.79315160
365348e-07,"10","Akr1a1"
"Serpinb6a2",3.21865486275107e-11,1.95157752696069,0.45,0.174,5.427295
82957086e-07,"10","Serpinb6a"
"Ubb1",3.25131056975777e-11,0.672869476379588,0.983,0.978,5.4823598827
2555e-07,"10","Ubb"
"Rpsa4",3.81403610526714e-11,0.665282048758244,0.983,0.987,6.431227680
70145e-07,"10","Rpsa"
"Ccr11",4.20228877076736e-11,1.15693528366273,0.533,0.205,7.0858993252
6792e-07,"10","Ccr1"
"Ifi2112",9.31221722584738e-11,1.14237410358476,0.333,0.094,1.57022606
862238e-06,"10","Ifi211"
"Snx61",1.08102541070311e-10,1.6899898305089,0.717,0.579,1.82282504752
758e-06,"10","Snx6"
"Rps212",1.944149100588e-10,0.673980786138417,0.983,0.988,3.2782242134
1149e-06,"10","Rps2"
"Pfn14",2.07774925600655e-10,0.726926135955624,0.967,0.946,3.503500795
47825e-06,"10","Pfn1"
"Lgals14",2.95523434652743e-10,1.59349354246905,0.467,0.186,4.98311615
511455e-06,"10","Lgals1"
"Fcna1",3.11446496770911e-10,1.28905990688696,0.25,0.058,5.25161082855
11e-06,"10","Fcna"
"Ap2s12",6.77927322165138e-10,1.36815620673572,0.667,0.475,1.143121050
63486e-05,"10","Ap2s1"
"Apoe2",1.16380650611243e-09,0.818789035549593,0.983,0.991,1.962410530
60678e-05,"10","Apoe"
"Mndal2",2.03115618663282e-09,1.14752766835813,0.35,0.115,3.4249355619
0025e-05,"10","Mndal"
"Mpp13",2.31241361583616e-09,1.45521711804396,0.55,0.302,3.89919183902
293e-05,"10","Mpp1"
"Trf1",6.36714618168952e-09,1.0229009837426,0.883,0.917,0.000107362818
915649,"10","Trf"
"Rbm34",7.69384908108807e-09,0.868835828382508,0.883,0.925,0.000129733
683205307,"10","Rbm3"
"Rcbtb21",1.08044051140124e-08,1.53711649553124,0.683,0.584,0.00018218
3879032477,"10","Rcbtb2"
"Hmgb11",1.17960777266788e-08,0.872795073760751,0.85,0.825,0.000198905
462627257,"10","Hmgb1"
"Cela1",1.37870212758038e-08,1.19148971258094,0.25,0.069,0.00023247675
2752604,"10","Cela1"
"Ifit31",1.58250347971207e-08,1.37077168585731,0.3,0.092,0.00026684173
6749049,"10","Ifit3"

"Ly6e2",1.84803301603516e-08,0.983994940678924,0.95,0.9,0.000311615327
163849,"10","Ly6e"
"Rps4x1",2.01246980974352e-08,0.518697559061184,0.95,0.985,0.000339342
659318953,"10","Rps4x"
"Rps33",2.27295294802852e-08,0.525627908711884,0.983,0.975,0.000383265
326096569,"10","Rps3"
"Rpl72",2.67388777107337e-08,0.547300032848712,0.983,0.971,0.000450870
955958391,"10","Rpl7"
"Arhgdib4",5.41262083472485e-08,0.917531187537458,0.867,0.868,0.000912
676125151305,"10","Arhgdib"
"Clec4a22",6.9381010174246e-08,1.53512034314313,0.533,0.329,0.00116990
259355814,"10","Clec4a2"
"Ctsc3",1.13760424604936e-07,1.08430378727424,0.833,0.891,0.0019182282
7968844,"10","Ctsc"
"Rpl62",1.20772726931808e-07,0.549142601463695,0.95,0.97,0.00203646972
152414,"10","Rpl6"
"Lyz23",2.09793104521636e-07,1.2387704386365,0.6,0.375,0.0035375313284
4383,"10","Lyz2"
"Sumo21",2.19675977482825e-07,0.967390992303904,0.767,0.781,0.00370417
633231539,"10","Sumo2"
"Fcgr32",2.24312452542822e-07,0.708382041253909,0.933,0.928,0.00378235
657477706,"10","Fcgr3"
"Hgsnat1",2.41936359291815e-07,1.93411560451426,0.467,0.267,0.00407953
089037859,"10","Hgsnat"
"Rpl191",2.50163403784841e-07,0.561084879172506,0.967,0.984,0.00421825
531461999,"10","Rpl19"
"Ms4a6d1",2.90701142093709e-07,1.65120592040761,0.667,0.58,0.004901802
65798412,"10","Ms4a6d"
"Psmb82",3.13712653841033e-07,1.10082990281366,0.767,0.771,0.005289822
7690675,"10","Psmb8"
"Rplp04",3.34012040612569e-07,0.453556845853401,0.967,0.986,0.00563211
102880914,"10","Rplp0"
"Btf33",4.10387929578692e-07,0.809377335342932,0.867,0.901,0.006919961
2685559,"10","Btf3"
"Aes1",5.24047510600492e-07,1.18244601716698,0.683,0.636,0.00883648912
37455,"10","Aes"
"Eif5a3",5.81363955867742e-07,0.781596765127046,0.817,0.875,0.00980295
902384186,"10","Eif5a"
"Rpl310",6.36019010485732e-07,0.520806650284266,0.967,0.973,0.01072455
25548104,"10","Rpl3"
"Cdc423",7.64350895316297e-07,0.48356239219227,0.967,0.957,0.012888484
7968234,"10","Cdc42"
"Atp6v1g13",8.7049820678971e-07,0.744612467784228,0.783,0.841,0.014678
3407628881,"10","Atp6v1g1"
"Chchd21",9.37875768647046e-07,0.712926344148123,0.833,0.859,0.0158144
612109265,"10","Chchd2"
"Arpc1b4",1.09814036232028e-06,0.616786685333993,0.95,0.976,0.01851684
27894445,"10","Arpc1b"
"Arf52",1.72253304073497e-06,0.953998386322387,0.733,0.76,0.0290453521
32873,"10","Arf5"

"Rps52",1.83111543280833e-06,0.52968119745861,0.983,0.966,0.0308762684
280141,"10","Rps5"
"Oaz13",2.1889883568689e-06,0.600585553729186,0.933,0.969,0.0369107216
735235,"10","Oaz1"
"Cd633",2.23171625398581e-06,0.658242507837274,0.933,0.885,0.037631199
4747087,"10","Cd63"
"Naca2",2.58815114479526e-06,0.676394770941734,0.85,0.922,0.0436414046
035377,"10","Naca"
"Psmb93",2.83892824610748e-06,1.44057556395064,0.517,0.375,0.047870008
0858643,"10","Psmb9"
"Ucp22",2.97644625187469e-06,0.818710613079425,0.833,0.904,0.050188836
699111,"10","Ucp2"
"Cpq1",3.89339172800316e-06,1.00574704196191,0.317,0.139,0.06565037131
75893,"10","Cpq"
"Msr12",4.78552644807888e-06,1.06754646670411,0.283,0.113,0.0806935469
675061,"10","Msr1"
"Prdx52",6.19226891768457e-06,0.948327687562546,0.8,0.835,0.1044140384
89997,"10","Prdx5"
"Hmgn21",7.13878058302617e-06,1.42016386914128,0.567,0.447,0.120374118
190987,"10","Hmgn2"
"Npl2",8.40552626074539e-06,1.34109350470568,0.55,0.417,0.141733983808
689,"10","Npl"
"Ifitm22",8.5296297583749e-06,0.803049672077718,0.433,0.223,0.14382661
6985717,"10","Ifitm2"
"Plin23",9.02796767354154e-06,1.12930436246395,0.6,0.475,0.15222959091
1257,"10","Plin2"
"Fcgr2b1",1.02426610005025e-05,1.49368264411968,0.683,0.688,0.17271174
9790472,"10","Fcgr2b"
"Rpl83",1.0666903781434e-05,0.459539509560345,0.933,0.977,0.1798653315
62539,"10","Rpl8"
"Itm2b1",1.37899489328941e-05,0.506386196305903,1,0.99,0.2325261189064
6,"10","Itm2b"
"Ap2m11",1.38269619175466e-05,1.27398818268255,0.633,0.621,0.233150231
853672,"10","Ap2m1"
"Snx51",1.57109248785295e-05,0.924353047809492,0.733,0.819,0.264917615
301765,"10","Snx5"
"Cfl13",1.72526582024674e-05,0.527167879927665,0.983,0.98,0.2909143226
10005,"10","Cfl1"
"Atp5g2",1.75226851054477e-05,0.756584462888458,0.75,0.805,0.295467516
24806,"10","Atp5g2"
"Mrfap12",1.86103783691552e-05,0.975555354139356,0.733,0.813,0.3138082
00060695,"10","Mrfap1"
"Gpx42",1.91702308041691e-05,0.829310830620406,0.75,0.824,0.3232484318
19899,"10","Gpx4"
"Rpl131",2.41780233116252e-05,0.429954736131107,0.983,0.987,0.40768982
9080624,"10","Rpl13"
"Aldh23",2.66078336328898e-05,1.24163857092104,0.6,0.517,0.44866129071
7788,"10","Aldh2"
"Rack13",2.73417293543922e-05,0.507257464572084,0.933,0.979,0.46103624
0373761,"10","Rack1"

"Stab11",2.87683471640019e-05,1.30198283924525,0.583,0.482,0.485091869
8794,"10","Stab1"
"Ptma1",2.98204940344988e-05,0.637229749011472,0.967,0.963,0.502833170
409718,"10","Ptma"
"Stmn11",3.77327999455742e-05,1.37224987093155,0.483,0.308,0.636250472
682271,"10","Stmn1"
"Eef1a12",4.03642358090338e-05,0.302371880817877,1,0.993,0.68062174421
1927,"10","Eef1a1"
"Ube2d2a1",4.0393245278822e-05,0.919938339975127,0.667,0.688,0.6811109
01891496,"10","Ube2d2a"
"Gnb22",4.05955026762518e-05,0.557036217666396,0.833,0.911,0.684521366
126958,"10","Gnb2"
"Rpl7a1",4.1631078069239e-05,0.558953755051887,0.883,0.952,0.701983238
403507,"10","Rpl7a"
"Rpl151",4.78509491401179e-05,0.477360823163571,0.95,0.965,0.806862704
400668,"10","Rpl15"
"Npc22",4.87769584070343e-05,0.409978070806747,0.95,0.962,0.8224770726
59412,"10","Npc2"
"Phf11b3",5.9995071536839e-05,1.21902520310758,0.383,0.226,1,"10","Phf
11b"
"Rpl92",6.34241674331866e-05,0.446025016652458,0.917,0.966,1,"10","Rpl
9"
"Cox4i13",7.76086112171785e-05,0.500043398638003,0.783,0.904,1,"10","C
ox4i1"
"Vamp8",8.4319457004251e-05,0.872842618507415,0.667,0.7,1,"10","Vamp8"
"Actr32",0.000101054404897726,0.67907463844432,0.85,0.891,1,"10","Actr
3"
"Bex31",0.000118764455893159,0.997077097681249,0.267,0.125,1,"10","Bex
3"
"Slc25a52",0.00012571998083618,0.57796951461438,0.833,0.9,1,"10","Slc2
5a5"
"Rpl102",0.000144957688484613,0.423507329897619,0.917,0.966,1,"10","Rp
l10"
"Pfdn5",0.000149750942858126,0.632824734979275,0.75,0.814,1,"10","Pfdn
5"
"Ube2l61",0.000156177252070805,1.01929074143596,0.267,0.126,1,"10","Ub
e2l6"
"Fcer1g2",0.000184351848628599,0.503959921632427,0.933,0.967,1,"10","F
cer1g"
"Rpl141",0.000193572284752917,0.489258868957282,0.85,0.949,1,"10","Rpl
14"
"Ppp1ca2",0.000206844513047483,0.876803912445004,0.717,0.806,1,"10","P
pp1ca"
"Calm21",0.000294916818117159,0.670028014116571,0.817,0.882,1,"10","Ca
lm2"
"Tmem106a1",0.000329070774382492,1.31305187409943,0.333,0.2,1,"10","Tm
em106a"
"Rnase41",0.000344972675181064,0.714310852992638,0.717,0.77,1,"10","Rn
ase4"
"Snrpb2",0.000352961050299468,0.800068980644912,0.683,0.771,1,"10","Sn

rpb"
"H2afz1",0.000353175522758572,0.427125690284105,0.817,0.882,1,"10","H2afz"
"Rnf1301",0.000365363934496578,0.844991661608128,0.7,0.803,1,"10","Rnf130"
"Rps62",0.000382611225186122,0.415491521223993,0.95,0.966,1,"10","Rps6"
"Clec4a12",0.00041063339883775,0.810870051417252,0.283,0.139,1,"10","Clec4a1"
"Hras1",0.000445573194100284,1.16610782580106,0.417,0.306,1,"10","Hras"
"H2-K12",0.000456056098129133,0.572463051545106,0.85,0.914,1,"10","H2-K1"
"Pnp1",0.000464884274332805,0.85912715954761,0.717,0.822,1,"10","Pnp"
"Hprt1",0.000472377326476692,0.865037561199963,0.65,0.645,1,"10","Hprt"
"Gatm2",0.000541591344457274,0.766946523739492,0.683,0.719,1,"10","Gatm"
"Atp5o.11",0.000552371789508802,1.08299313661024,0.55,0.51,1,"10","Atp5o.1"
"Use11",0.000557420763717334,1.20908942317322,0.567,0.559,1,"10","Use1"
"Ccl71",0.000578613022115773,1.15327329561881,0.417,0.244,1,"10","Ccl7"
"Pdia62",0.000594703385968721,0.83319528313757,0.667,0.717,1,"10","Pdia6"
"Eif5b",0.000622597436693237,0.31215454424826,0.25,0.564,1,"10","Eif5b"
"Atp5d2",0.000636897922948553,0.760498207264153,0.65,0.765,1,"10","Atp5d"
"Rpl51",0.000643358124221631,0.355803275350515,0.933,0.963,1,"10","Rpl5"
"Rpl122",0.000671885341728382,0.541500850534575,0.883,0.94,1,"10","Rpl12"
"Ubxn1",0.000714513463996092,0.833521305544824,0.633,0.678,1,"10","Ubxn1"
"Psma43",0.00071560763510585,0.938150429474408,0.583,0.578,1,"10","Psma4"
"Etfb1",0.000735319582292045,1.01651864300915,0.483,0.415,1,"10","Etfb"
"Capza22",0.000737358256755525,0.598438554891949,0.85,0.935,1,"10","Capza2"
"Selenof3",0.000776512205928579,0.667004183361016,0.767,0.861,1,"10","Selenof"
"Scp21",0.000811141754653242,1.00452381879841,0.617,0.649,1,"10","Scp2"
"Hfe2",0.000849118843930495,0.866553288697172,0.517,0.477,1,"10","Hfe"
"Rps82",0.000887166561250713,0.39510988710621,0.9,0.973,1,"10","Rps8"
"Arpc23",0.000915590981323638,0.393785900586674,0.933,0.961,1,"10","Arpc2"

"Lamp13",0.000922334158617538,0.53550769273052,0.917,0.96,1,"10","Lamp1"
"Laptm4a1",0.000975686726486847,0.783375934983,0.667,0.802,1,"10","Laptm4a"
"Gbp71",0.000987222853771411,1.14334872610898,0.383,0.257,1,"10","Gbp7"
"Ninj12",0.00101500767058964,0.989191531780488,0.633,0.645,1,"10","Ninj1"
"Rpl43",0.00104046250439702,0.444933434552297,0.9,0.928,1,"10","Rpl4"
"Plscr3",0.00118770089921897,1.15256623165635,0.35,0.238,1,"10","Plscr3"
"Rab5c1",0.00120298839895481,1.00483001979213,0.65,0.725,1,"10","Rab5c"
"Rrp11",0.00130009810608531,0.286576832208139,0.233,0.547,1,"10","Rrp1"
"Hmgn11",0.00136064885264141,0.903294385230912,0.617,0.647,1,"10","Hmgn1"
"Atp5h1",0.0013896941805485,0.932707531003051,0.617,0.703,1,"10","Atp5h"
"Ran2",0.00147448216012137,0.69902000779162,0.667,0.725,1,"10","Ran"
"Slc25a32",0.0015784561386583,0.780025484208308,0.833,0.913,1,"10","Slc25a3"
"Tagln22",0.00163911072984626,0.726431435883213,0.567,0.475,1,"10","Tagln2"
"Psme11",0.00167263561925459,0.789820729012845,0.65,0.773,1,"10","Psme1"
"0610012G03Rik",0.00170703419938616,1.14462895714692,0.45,0.393,1,"10","0610012G03Rik"
"Unc1191",0.00172023374400054,0.793513707414883,0.25,0.129,1,"10","Unc119"
"Lamtor12",0.00172441623534574,0.865744512029659,0.633,0.722,1,"10","Lamtor1"
"Isg153",0.00185748935474861,1.08038256938471,0.317,0.19,1,"10","Isg15"
"Atp5f13",0.00193518396593851,0.505094470162276,0.733,0.821,1,"10","Atp5f1"
"1110008F13Rik2",0.00196003319538356,0.75283677764841,0.617,0.643,1,"10","1110008F13Rik"
"Sem11",0.002022763669159,0.92154331596366,0.6,0.622,1,"10","Sem1"
"Hint11",0.00204906801335083,0.752884697072893,0.65,0.738,1,"10","Hint1"
"Clic11",0.00235500850515797,0.480158628492064,0.85,0.944,1,"10","Clic1"
"Ehd41",0.00242370609621116,0.909979879256939,0.633,0.703,1,"10","Ehd4"
"H2-D12",0.00254791543292057,0.403802573793388,0.883,0.95,1,"10","H2-D1"
"Txndc17",0.00268386581426168,1.04925146454432,0.517,0.5,1,"10","Txndc17"
"Cyba2",0.0027126188881628,0.493980499065316,0.883,0.959,1,"10","Cyba"

"Atp5j1",0.00279205132383956,0.888348025345115,0.583,0.639,1,"10","Atp
5j"
"Atp5c13",0.00281087269492474,0.721917232897753,0.683,0.782,1,"10","At
p5c1"
"Igf13",0.00301242409578206,0.721723561519273,0.383,0.253,1,"10","Igf1
"
"Sh3bgrl33",0.00304799010788104,0.397888967162885,0.85,0.919,1,"10","S
h3bgrl3"
"Nedd8",0.00306043963315853,0.901214300845036,0.55,0.596,1,"10","Nedd8
"
"Nr1h21",0.00319579812941562,0.287666873506402,0.133,0.358,1,"10","Nr1
h2"
"Atp5a11",0.00329144021805368,0.631197778317153,0.683,0.812,1,"10","At
p5a1"
"Pnpla81",0.00373720811178887,0.302708284782025,0.133,0.358,1,"10","Pn
pla8"
"Psmb42",0.00390942885543252,0.828705989481913,0.6,0.657,1,"10","Psmb4
"
"Calm12",0.0044058307168817,0.690699363767698,0.8,0.922,1,"10","Calm1"
"Rac22",0.00441984806170578,0.756524621964958,0.65,0.766,1,"10","Rac2"
"Eef1b22",0.00454657570654974,0.417166069699808,0.867,0.927,1,"10","Ee
f1b2"
"Anp32b1",0.00458164392809165,0.905308057001947,0.583,0.604,1,"10","An
p32b"
"Tmem176a1",0.00459425748515954,0.979876013610692,0.733,0.792,1,"10","
Tmem176a"
"Necap21",0.00470981952253671,0.953371331351244,0.583,0.65,1,"10","Nec
ap2"
"Capzb3",0.00531807190594868,0.522270570875573,0.75,0.888,1,"10","Capz
b"
"Hexa3",0.00563080793728973,0.598980577336742,0.767,0.912,1,"10","Hexa
"
"Glmp1",0.00607135338661671,0.26033683613507,0.317,0.659,1,"10","Glmp"
"Ndufb81",0.00649263225787728,0.923875119072374,0.517,0.515,1,"10","Nd
ufb8"
"Tm2d21",0.00673222220947413,0.905142794653418,0.517,0.536,1,"10","Tm2
d2"
"Tm9sf2",0.00709981373243551,0.253592667556871,0.15,0.368,1,"10","Tm9s
f2"
"Ehd11",0.00710182045916833,0.877992800255496,0.55,0.555,1,"10","Ehd1"
"Tmem14c2",0.00719110579887908,0.622772223701328,0.633,0.715,1,"10","T
mem14c"
"Abhd17a",0.0080979265822115,0.312100745799564,0.233,0.496,1,"10","Abh
d17a"
"Psma73",0.00844818378878001,0.75462451998353,0.617,0.666,1,"10","Psma
7"
"Rps72",0.00852437960059774,0.423452079486686,0.9,0.946,1,"10","Rps7"
"Ncoa41",0.00932601026007406,1.15462728752369,0.367,0.293,1,"10","Ncoa
4"
"Ensa1",0.00988651921240831,0.319940719366167,0.183,0.433,1,"10","Ensa

"
