# Supplementary material for: Single-cell transcriptome analyses reveal microglia types associated with proliferative retinopathy
Source: JCI Insight. 2022 Dec 8;7(23):e160940. doi: 10.1172/jci.insight.160940 (PMC9746914; doi:10.1172/jci.insight.160940)
Supplement: Supplemental table 2 [file jciinsight-7-160940-s223.pdf]

```
"","p_val","avg_log2FC","pct.1","pct.2","p_val_adj","cluster","gene"
"1",5.31946386757398e-105,1.25549729116785,0.998,0.771,8.9696799735032
4e-101,"0","Ier5"
"2",1.98607106193287e-99,1.40466588793594,0.995,0.763,3.34891302463121
e-95,"0","Rhob"
"3",1.67046564594163e-91,1.09584813841618,0.998,0.876,2.81673917218677
e-87,"0","Jund"
"4",1.28548335297366e-83,1.11734178469083,0.824,0.396,2.16758202978419
e-79,"0","Sertad1"
"5",1.31122732220766e-83,1.19482265632404,0.963,0.66,2.21099151070656e
-79,"0","Jmjd1c"
"6",6.4041287355696e-81,1.14427944800629,0.998,0.796,1.07986418739175e
-76,"0","Btg2"
"7",2.62663392186684e-70,1.14827473258941,0.983,0.704,4.42903011905186
e-66,"0","Ifrd1"
"8",6.1706411611102e-66,1.61624694743704,0.745,0.357,1.0404935125864e-
61,"0","Rrad"
"9",4.15660263768674e-62,1.32123052068005,0.956,0.699,7.00886336766739
e-58,"0","Atf3"
"10",5.66775348576552e-43,1.31138410104506,0.887,0.602,9.5569659276978
2e-39,"0","Klf2"
"11",2.65533840644532e-60,0.912572990494412,1,0.827,4.47743162094809e-
56,"1","Nfkbia"
"12",7.85138426136222e-54,1.0852089612253,0.988,0.679,1.3239004141509e
-49,"1","Gpr84"
"13",5.67678882474295e-50,0.94064134490969,0.96,0.615,9.57220131628156
e-46,"1","Gadd45b"
"14",8.2166155735808e-49,1.3160250767422,0.839,0.49,1.3854857180172e-4
4,"1","Arl5c"
"15",6.34377166380242e-39,0.907106964869106,0.963,0.652,1.069686777950
36e-34,"1","Tnf"
"16",9.5266117599547e-39,0.774087894735045,0.974,0.659,1.6063772749635
6e-34,"1","Tnfaip3"
"17",1.63721561815449e-38,0.835362330942275,0.991,0.835,2.760672975332
1e-34,"1","Bcl2a1b"
"18",3.23536869434388e-38,0.797857586676466,0.977,0.692,5.455478692402
65e-34,"1","Icam1"
"19",7.6166654736479e-36,0.767885964170282,0.991,0.711,1.2843221321665
1e-31,"1","Il1a"
"20",4.98099455880313e-31,1.01221532613403,0.804,0.521,8.3989530250538
5e-27,"1","Dusp2"
"21",2.39376797928054e-82,2.09482689417885,0.972,0.469,4.0363715666628
4e-78,"2","Rcan1"
"22",1.10186314003393e-70,1.49717046135594,0.762,0.225,1.8579616267252
e-66,"2","Nr4a3"
"23",3.5705158460793e-69,1.48149640207329,0.953,0.497,6.02060381965891
e-65,"2","Egr2"
"24",2.71338966684212e-59,1.77621712756011,0.808,0.339,4.5753176562291
9e-55,"2","Egr3"
"25",1.13857363725586e-51,1.1906118740961,0.893,0.463,1.91986286714083
```

e-47,"2","Tagap"
"26",3.31460974860731e-51,1.42352452951594,0.977,0.675,5.5890949581016
4e-47,"2","Tnf"
"27",8.34598704416096e-48,1.32386059478909,0.991,0.783,1.4073003353864
2e-43,"2","Ier3"
"28",5.1416777879501e-47,1.1768305584011,1,0.768,8.66989708604146e-43,
"2","Ccl4"
"29",2.41596181913929e-42,1.42662660583123,0.953,0.647,4.0737948194326
7e-38,"2","Ccrl2"
"30",2.24972611815972e-35,1.19929667219587,0.584,0.22,3.79348818044091
e-31,"2","Gm26522"
"31",8.15814659173486e-20,1.54351927882762,0.9,0.961,1.37562667829833e
-15,"3","Gapdh"
"32",4.54512006314614e-12,1.48162005982841,0.781,0.861,7.6639814504770
3e-08,"3","Pkm"
"33",4.21982948718024e-08,1.04455174423925,0.725,0.838,0.0007115476481
28332,"3","Aldoa"
"34",3.25141595199378e-06,1.06031451425678,0.719,0.87,0.05482537578251
92,"3","Bsg"
"35",4.24487457003608e-06,1.42801217824454,0.625,0.692,0.0715770749999
484,"3","Pgam1"
"36",2.09855695079534e-05,1.59794676103045,0.556,0.601,0.3538586730431
11,"3","Tpi1"
"37",0.000122170943350126,1.16410030325,0.6,0.685,1,"3","Eno1"
"38",0.00013008159044962,1.3450318053563,0.656,0.819,1,"3","Ldha"
"39",0.000254098724834588,1.35786708765928,0.556,0.584,1,"3","Pgk1"
"40",0.00473393372459398,1.37655395133588,0.312,0.238,1,"3","Lgals3"
"41",1.73264901812027e-229,2.92982924339247,0.677,0.01,2.9215927743544
e-225,"4","Bmp2"
"42",1.39255762636649e-215,2.77830160703549,0.962,0.065,2.348130669579
18e-211,"4","Ms4a7"
"43",1.12424140819354e-212,3.30777371502049,0.949,0.066,1.895695862495
95e-208,"4","Mrc1"
"44",3.86946527839643e-177,3.15746926850581,0.899,0.076,6.524692352432
06e-173,"4","Pf4"
"45",4.65037474582776e-155,2.48354318181875,0.753,0.057,7.841461896414
77e-151,"4","F13a1"
"46",3.61978400768153e-153,2.5201334156914,0.589,0.024,6.1036797937526
e-149,"4","Lyve1"
"47",1.60873450541076e-148,2.67346359403476,0.962,0.147,2.712648123023
63e-144,"4","Ccr1"
"48",1.04913155446814e-137,2.80160873634128,0.937,0.15,1.7690456271441
9e-133,"4","Dab2"
"49",7.28725935322498e-93,3.36652731766054,0.861,0.201,1.2287776721408
e-88,"4","Cxcl2"
"50",1.5222504549251e-86,3.77976265959142,0.816,0.197,2.56681871709471
e-82,"4","Ccl7"
"51",6.12110048440855e-214,2.64170335895549,0.701,0.018,1.032139963680
97e-209,"5","Top2a"
"52",1.46588884359813e-201,2.35255871283608,0.707,0.023,2.471781768075

17e-197,"5","Pclaf"
"53",1.2083099975193e-182,2.70848344581882,0.667,0.024,2.0374523178170
4e-178,"5","Ube2c"
"54",4.32138690942269e-178,2.47947787986873,0.667,0.026,7.286722606668
54e-174,"5","Birc5"
"55",4.07150956830901e-129,2.47350580176935,0.612,0.043,6.865379434082
65e-125,"5","Mki67"
"56",6.62341650563327e-120,2.45536356860287,0.762,0.099,1.116840491179
88e-115,"5","Cdk1"
"57",2.28746459275021e-111,3.26619188418487,0.966,0.258,3.857122796295
41e-107,"5","Stmn1"
"58",1.04192530278348e-78,2.28547551330531,0.741,0.165,1.7568944455535
1e-74,"5","H2afx"
"59",2.20779996585582e-71,2.53263963791838,0.966,0.526,3.7227923024260
8e-67,"5","Hmgb2"
"60",2.53629252752027e-66,2.45572618164224,0.993,0.87,4.27669645990467
e-62,"5","H2afz"
"61",0,4.35831376940728,0.92,0.012,0,"6","Plac8"
"62",0,3.24470745877453,0.949,0.01,0,"6","Napsa"
"63",9.44115097066059e-266,3.26743190444711,0.79,0.012,1.5919668766727
9e-261,"6","S100a6"
"64",1.70805140037052e-236,2.92163365367226,0.942,0.049,2.880116271304
77e-232,"6","Cytip"
"65",2.39741037702316e-136,2.8825297407532,1,0.186,4.04251337773645e-1
32,"6","Iqgap1"
"66",1.2825564823845e-125,3.01288239968217,0.949,0.173,2.1626467405967
4e-121,"6","Ifitm2"
"67",1.12343649548201e-98,3.42399394790801,0.609,0.065,1.8943386186817
7e-94,"6","Thbs1"
"68",2.87573767669178e-97,2.94477565778679,0.775,0.129,4.8490688704376
8e-93,"6","Crip1"
"69",3.56063204528895e-96,3.23868599676906,0.957,0.253,6.0039377547662
3e-92,"6","Ifitm3"
"70",3.2890429510109e-20,3.09765376790362,0.652,0.361,5.54598422399458
e-16,"6","Lyz2"
"71",1.06969234992904e-13,0.852557053181005,0.946,0.859,1.803715240450
34e-09,"7","Lpcat2"
"72",5.85233893701077e-11,0.860654901465054,0.946,0.852,9.868213915587
55e-07,"7","Selplg"
"73",3.13167248560372e-10,0.829960509589987,0.871,0.819,5.280626145224
99e-06,"7","Gpr34"
"74",2.20551843488579e-09,0.922363232890568,0.806,0.825,3.718945184904
42e-05,"7","Vsir"
"75",2.57420029111961e-06,0.928114431810174,0.774,0.832,0.043406165308
8588,"7","Malat1"
"76",3.20554178529307e-06,0.818305867479424,0.763,0.752,0.054051845583
6118,"7","Tgfbr1"
"77",1.01386276546417e-05,1.08944313620867,0.677,0.554,0.1709575395125
68,"7","Sgk1"
"78",1.5687179772739e-05,0.978584179832723,0.656,0.647,0.2645172253279

25,"7","P2ry13"
"79",3.71181682550102e-05,0.814638850460939,0.624,0.601,0.625886553115
982,"7","Pla2g15"
"80",0.000183506090723417,0.882156963914622,0.667,0.624,1,"7","Ccr5"
"81",5.25715955590059e-22,1.86105583668732,0.346,0.062,8.8646224431595
7e-18,"8","Serpine1"
"82",2.62749941941826e-20,1.50607867795025,0.605,0.198,4.4304895210230
7e-16,"8","Csf1"
"83",3.99739597385607e-14,1.0749388457617,0.901,0.516,6.74040909111611
e-10,"8","Id2"
"84",8.24493063596678e-13,1.74691720148288,0.42,0.142,1.39026020383672
e-08,"8","Slc7a11"
"85",6.39269995556774e-12,1.02916427192956,0.901,0.701,1.0779370665078
3e-07,"8","Tlr2"
"86",3.68746141044752e-10,0.990707520917765,0.951,0.836,6.217797430296
6e-06,"8","Cd14"
"87",4.76942368662953e-09,1.27712608389391,0.84,0.547,8.04220222039472
e-05,"8","Sgk1"
"88",3.64670899768413e-08,1.11672623786401,0.691,0.442,0.0006149080711
89498,"8","Lpl"
"89",4.11229728001851e-07,1.20471300306086,0.63,0.4,0.0069341556735672
2,"8","Sdc4"
"90",7.15646932149091e-07,1.12377737381416,0.346,0.147,0.0120672385698
98,"8","Ptgs2"
"91",7.84146066382859e-51,3.19567204393885,0.908,0.22,1.32222709713478
e-46,"9","Lgals3"
"92",4.09267007313611e-49,3.29517437360644,0.892,0.234,6.9010602773221
1e-45,"9","Fabp5"
"93",8.28942544410027e-44,3.39479277583433,0.892,0.306,1.3977629183841
9e-39,"9","Gclm"
"94",2.29226861599197e-42,3.44934850696986,1,0.997,3.86522334028566e-3
8,"9","Ftl1"
"95",6.70718077566146e-42,3.37792508427493,1,0.994,1.13096482239204e-3
7,"9","Fth1"
"96",2.14460332715565e-40,4.03019185813474,1,0.859,3.61623013024985e-3
6,"9","Prdx1"
"97",6.60337081000759e-38,3.01241465227708,0.831,0.288,1.1134603859834
8e-33,"9","Slc48a1"
"98",1.23624766031941e-36,3.87454376051574,0.985,0.727,2.0845608048305
9e-32,"9","Hmox1"
"99",2.15639462728846e-34,2.71160850946016,0.846,0.346,3.6361126205338
1e-30,"9","Ftl1-ps1"
"100",2.84701074404767e-32,3.00774179310812,0.938,0.611,4.800629516613
18e-28,"9","Esd"
"101",8.2102450423916e-66,3.08863607584829,0.883,0.121,1.3844115190480
7e-61,"10","Pf4"
"102",9.1395840741679e-58,2.47576202208957,0.833,0.118,1.5411166665861
9e-53,"10","Mrc1"
"103",1.77227480820302e-53,2.51555084250377,0.8,0.119,2.98840978159194
e-49,"10","Ms4a7"

"104",1.98289846732278e-46,2.38142476332944,0.683,0.097,3.34356339559968e-42,"10","F13a1"
"105",2.70077867742983e-35,2.27708631462894,0.683,0.137,4.55405300588218e-31,"10","Clec4n"
"106",6.16044245066485e-32,2.59431717534554,0.817,0.281,1.03877380603111e-27,"10","Igfbp4"
"107",6.77189945353803e-31,2.61771668251697,0.833,0.297,1.14187768585558e-26,"10","Ifi27l2a"
"108",2.00297042686697e-30,2.11745981776494,0.517,0.089,3.37740873378308e-26,"10","Folr2"
"109",5.32631143303431e-21,2.16767622967926,0.867,0.586,8.98122633838246e-17,"10","Snx2"
"110",1.60016037669559e-18,2.25136688204625,0.85,0.616,2.6981904271841e-14,"10","Fcgrt"
